# Analysis of the Antigenic Properties of Membrane Proteins of *Mycobacterium tuberculosis*

**DOI:** 10.1038/s41598-019-39402-z

**Published:** 2019-02-28

**Authors:** Haifeng Li, Liguo Liu, Wei-jia Zhang, Xiaobing Zhang, Jianhua Zheng, Li Li, Xiuyun Zhu, Qianting Yang, Mingxia Zhang, Haiying Liu, Xinchun Chen, Qi Jin

**Affiliations:** 1 NHC Key Laboratory of Systems Biology of Pathogens, Institute of Pathogen Biology, Chinese Academy of Medical Sciences & Peking Union Medical College, Beijing, China; 2 Institute of Deep-sea Science and Engineering, Chinese Academy of Sciences, Sanya, Hainan China; 3 Guangdong Key Laboratory for Emerging Infectious Diseases, Shenzhen Key Laboratory of Infection & Immunity, Shenzhen Third People’s Hospital, Shenzhen, China; 4 Department of Pathogen Biology, Shenzhen University School of Medicine, Shenzhen, China

## Abstract

Tuberculosis (TB) is a continuing major threat to global health and a leading cause of death, particularly in developing countries. In this study, we aimed to identify a specific and sensitive diagnostic biomarker and develop a vaccine to prevent this disease. We investigated membrane proteins to reveal biomarkers in serum and peripheral blood mononuclear cells (PBMCs) obtained from TB patients. We employed Western blotting to evaluate serological immunoglobulin G levels, and Enzyme Linked Immunospot (ELISpot) to assess the antigen-specific cellular interferon-γ secretion from PBMCs after membrane protein stimulation. A total of 219 membrane proteins were identified, 52 exhibited at a higher levels than the 38-kDa prositive control. Of these 18 exhibited reacted ratios above 1, especially Rv1111c (427–981), with a ratios at 3.38. Accuracy and sensitivity were markedly higher for the top two antigen candidates, Rv0232 and Rv1115, after two rounds of ELISpot tests than ESAT-6 in the commercial kit (42.15 and 43.62%, respectively). These two proteins were administered to mice to detect whether they acted as effective antigens *in vivo*. These data provide a comprehensive view of the membranes involved in humoural and cellular immune responses that may be used as biomarkers for TB and candidates for a vaccine.

## Introduction

Despite nearly 140 years of biomedical research since the discovery of the tubercle bacillus, tuberculosis (TB) continues to be a major threat to global health and is a leading cause of death, particularly in the developing countries. As TB ranking alongside HIV/AIDS in worldwide mortality^1^, most countries are responding to the WHO End Tuberculosis (TB) Strategy by 2035. As a country with a high TB burden, China has developed new tools, especially in TB diagnosis, to reduce the epidemic. Currently, the gold standards for obtaining an aetiological diagnosis of TB are sputum-positive smear and culture. However, these methods are restricted because they are time-consuming to perform and these pathogenic bacteria are difficult to trace. Therefore TB diagnosis remains a challenge, as more than 50% of patients lack aetiological evidence. In addition, due to the presence of intracellular bacteria^2^, vaccine failure, and drug resistance, no single method accurately diagnoses all clinical TB cases. The antigen-specific IFN-γ release assay, which is based on host immune response detection, is a reliable auxiliary tool that can be used in the clinic and is recommended by the WHO for detect *Mycobacterium tuberculosis (M.tb)* infection^3,4^.

Nonetheless, newer, more rapid and accurate diagnostic methods are required because current methods are not superior to the tuberculin skin tests or able to differentiate latent *M. tb* infection from active TB. Additionally, the rate of missed diagnoses is high, as only 60% to 80% of active pulmonary TB is diagnosed by the present established methods. Thus, identification of novel biomarkers for both the host and the pathogen is key to increasing the accuracy of TB diagnosis^5^. Recently, scientists are focusing on deciphering each *M.tb* gene or protein function^6^. Among more than 4,000 *M.tb* open reading frame (ORF), secreted proteins (e.g., ESAT-6, CFP-10, and Ag85A/B) are thought to stimulate an antigen specific immune response^7,8^ and are well-studied *M.tb* antigens used for diagnosis and vaccine development. However, there are still limits to the use of these proteins in the clinical setting because they cannot used to differential diagnosis between TB infection and TB and only 70% of positive clinical TB patients are identified by these proteins^9^. Previously, we comprehensively examined the functions of 1,250 proteins (representing approximately one-third of *M.tb* proteins)^10^. To obtain more detailed and reliable information, we focused on membrane proteins in the present study^11^. Although *M.tb* membrane proteins have long been considered to be immunogens, they have not been systematically studied. Membrane proteins are those that interact with or components of biological membranes and include integral membrane proteins that are permanently anchored or are components of the membrane, as well as peripheral membrane proteins, which are only temporarily attached to the lipid bilayer or to other integral proteins. Certain membrane proteins play vital roles in many cellular processes, for example membrane receptor proteins relay signals between the internal and external environments, transport proteins move molecules and ions across the membrane, and cell adhesion molecules, such as proteins involved in the immune response, allow cells to identify and interact each other^12^.

To obtain a better understanding of these proteins, we expressed and purified all *M.tb* membrane proteins and examined via three rounds of serological immunity to determine their usefulness as potential serological diagnosis biomarkers. In addition, two rounds of cell-mediated immunity tests were carried out to evaluate the proteins for their suitability as screening biomarkers. Further analysis of membrane protein antigens generated by the cellular antigenic response in TB patients may accelerate antigen biomarker research and improve TB diagnosis and vaccine development.

## Results

### Bioinformatics analysis of target proteins

**Figure 1:**
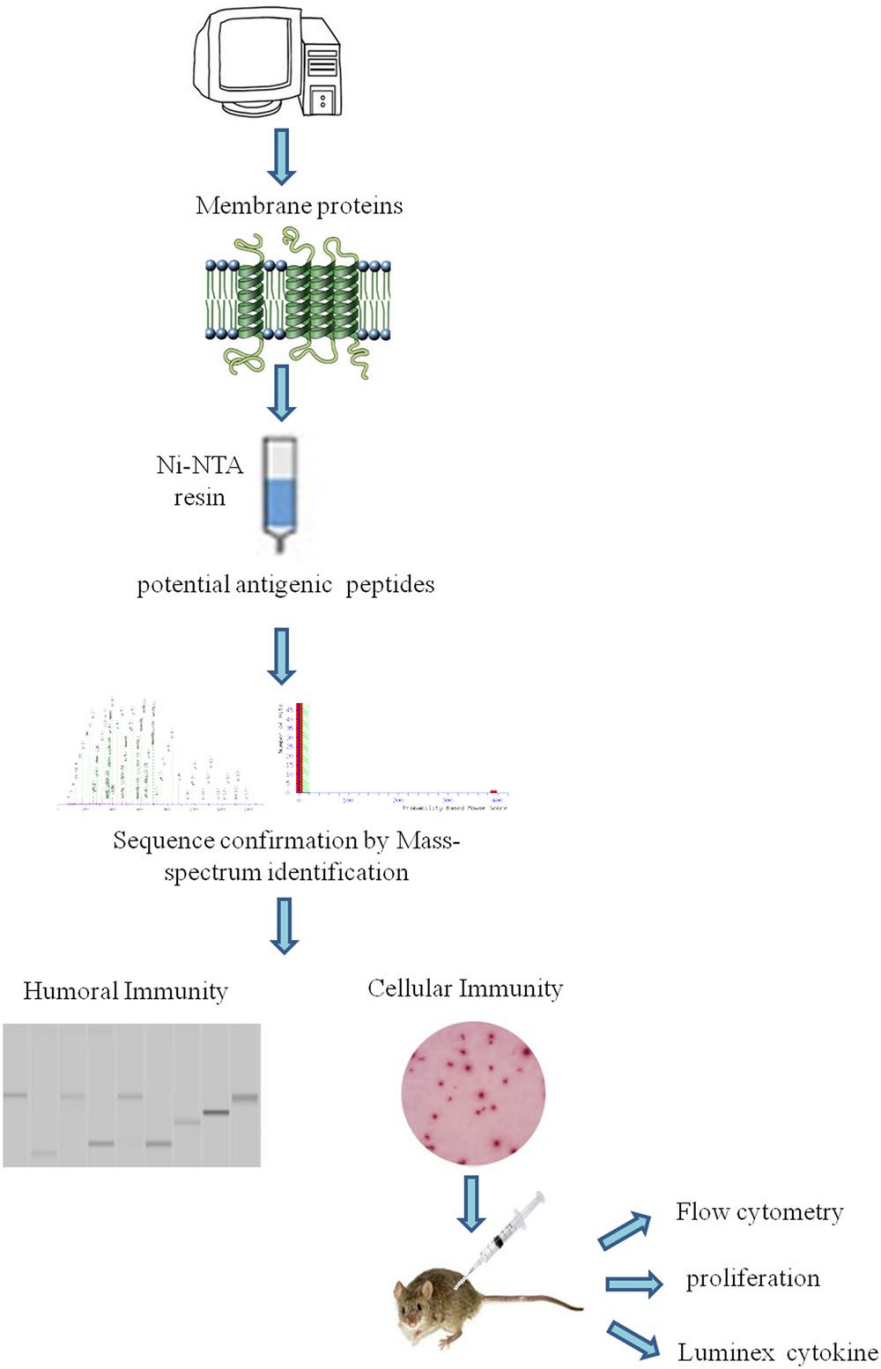
Flow chart of the *M. tuberculosis* H37Rv membrane proteomic antigenicity detection. The sequence of H37Rv was downloaded and analysed by PSORT and TMHMM version 2. Target proteins contain transmembrane α-helices and membrane subcellular localization. Gateway technology was used for target protein expression. The purified proteins were detect by ELISpot and Western blotting. Positive proteins were used as antigens to immune mice and detect T-cell proliferation and cytokines.

ORFs of membrane proteins of *M.tb* H37Rv was identified and predicted by PSORTb 3.0 (http://psort.hgc.jp/form2.html) and TMHMM 2 (Fig. 1). According to PSORT, 992 *M.tb* H37Rv proteins are localized to cytoplasmic membrane proteins. In total, 676 of the proteins contain hydrophobic domains and more than 100 amino acids; whereas 428 proteins did not have trans-membrane domains (Tm helix = 0) and were considered negative result, and 248 of the ORFs identified were regarded as membrane proteins. A total of 248 membrane proteins were cloned and purified. Finally, 219 membrane proteins were purified for functional analysis(Supplement Table S1). The serology and cytology of purified proteins from clinical pulmonary tuberculosis patients were screened and identified separately.

### Membrane protein cloning expression and purification

A total of 248 membrane proteins were cloned, expressed, purified and quantified. Of these, 219 were eligible for functional analyses (Supplemental Table S1) such as serology and cytology analyses using clinically obtained pulmonary tuberculosis patient serum and PBMCs.

### Identification of *M. tb* serological antigens by Western blotting

**Figure 2:**
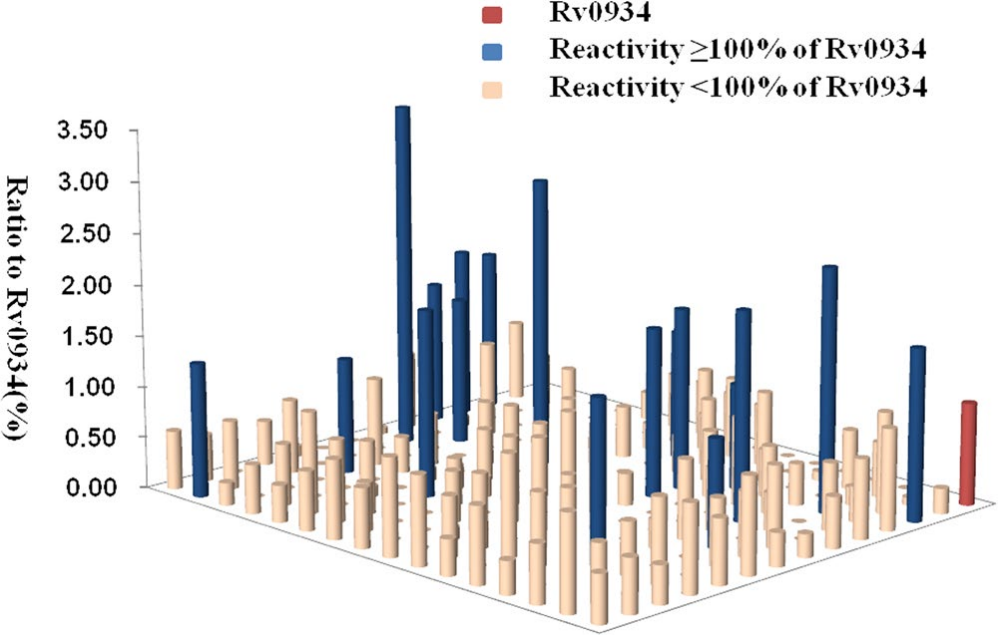
Serum IgG levels of 219 membrane proteins in 90 pulmonary TB patients. The heights and colors of the columns indicate the reactivity of each protein, as evaluated based on the ratio of reactivity between the tested protein and commercial Rv0934 protein. The red column represents the commercial Rv0934 protein, for which ratio was set to 1. The yellow columns represent proteins for which a response by serum obtained from active TB patients was obtained but with a reactivity ratio below 1. The blue columns represent proteins with a ratio above 1.

Although humoural immunity remains an auxiliary means of diagnosing TB, we expected the results of Western blotting to provide us with useful *M.tb*-specific 6 antigens for serum IgG, as based on our previous method. Transmembrane proteins play important roles in the transport of substances and immune protection. To assess the antigenic properties of the induced humoral response in patients, Western Blotting was performed to identify serological antigens in pulmonary TB patients (Table 1) and healthy controls (Table 2). To avoid the influence of Bacillus Clamette- Guérin (BCG) vaccination, the healthy controls included individuals with or without a BCG vaccine scar. Additionally, a positive protein response to active pulmonary TB patient serum was used to screen control candidate serum (data not show). All serum samples were pretreated to remove the *E. coli* background as reported previously^10^, and bovine albumin (BSA) and commercial Rv0934 were used as the negative and positive control. To evaluate the antigenicity of each *M.tb* protein, the serum response intensity ratio of expression level of the target proteins to that of Rv0934 was calculated; if the ratio was equal to or greater than 1, the protein was confirmed in second and third rounds. After the first round of testing, we obtained positive responses for 161 of the 219 membrane protein candidates (approximately 74%). After the second round, 52 membrane proteins had a ratio greater than that of Rv0934 (Fig. 2, Supplemental Tables S1, Figs S2–S4); the yellow bars indicate positive proteins, and the ratio ranged from 0.02% to 338%. After three rounds, 18 of the remaining membrane proteins had ratios above 1, (Fig. 2, dark blue bars). The ratio of Rv1111c (427–981) was particularly high (a mean ratio at 3.38).

**Table 1:** Characterization of patients by a three-round test of antigenic proteins via Western blotting: out of 219 membrane proteins, 52 had ratios above 1 after the first round, 12 had ratios above 1 after the second round, and 10 had ratios were above 1 after the third round.

Characteristics	First round	Second round	Third round
Total number	30	30	30
Age, mean (range, years)	40 (21–60)	43 (23–66)	36 (17–53)
Male/Female	19/11	20/10	22/8
Bacteriological test positive/negative	30/0	30/0	30/0
ELISPOT positive, Mean (range)	30 141.5 (41–299)	30 139.9 (40–292)	30 98.3 (42–290)

**Table 2:** Characterization of control candidates for test of antigenic proteins by Western blotting.

Characteristics	Control with BCG scar	Control without BCG scar	Active TB
Total number	60	60	12
Age, mean range, years)	39 (37–42)	39 (37–42)	36 (17–53)
Male/Female	35/25	30/30	22/8
Bacteriological test positive/negative	—	—	30/0
ELISPOT positive, Mean (range)	—	—	30 98.3 (42–290)
BCG scar	no	yes	yes
(IGRA) TB Ag	negative	negative	—

### Identification of *M. tb* antigenic stimulating IFN-γ secretion

**Figure 3:**
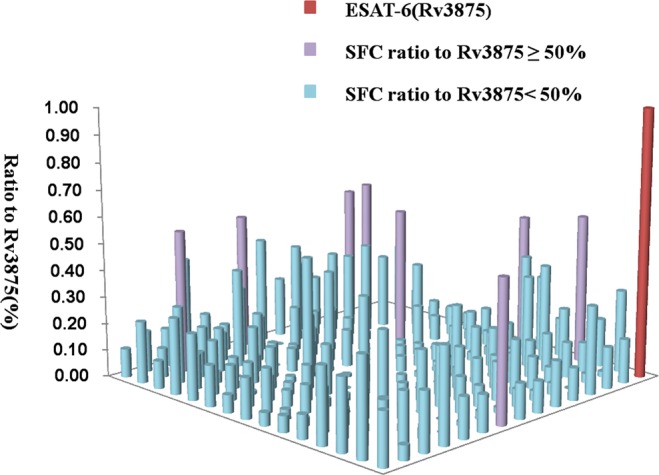
TB antigen-specific IFN-γ-producing SFC reaction ratio of purified membrane proteins. The heights and colors of the columns indicate the reactivity of each protein, as evaluated based on the ratio of the reactivity between the tested protein and commercial containing kit Rv3875. The red column represents the commercial kit Rv3875, for which the ratio set to 1. The light blue columns represent proteins with a ratio below 50%. The purple columns represent the proteins with a ratio higher than 50%.

**Figure 4:**
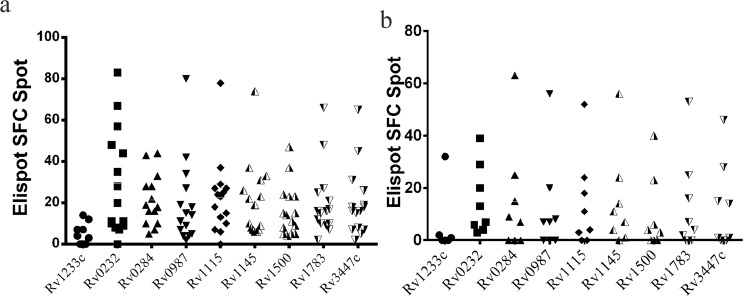
Second-round screen of proteins with a reactivity above 40% using PBMCs from pulmonary TB patients (n = 15). The bar indicates the means plus SEM. (**a**) The SFC of membrane proteins for ESAT-6-positive patients. (**b**) The SFC of membrane proteins for ESAT-6-negative patients.

TB is a disease mediated by cellular response, and membrane proteins often play a vital role in the interaction between bacteria and host. Hence, 219 *M.tb* membrane proteins were tested by enzyme-linked immunospot (ELISpot) using the commercial antigen ESAT-6 as a positive control; purified ESAT-6 protein was included in parallel experiments. The spot forming cell (SFC), the two ESAT-6 proteins included with the kit, and Gateway expression system used in the present study were approximate, indicated sufficient reactivity by proteins in the expression system. An assay result was considered positive only when the ESAT-6 protein expressed from the Gateway expression system led to stimulation greater than the cut-off recommended by the manufacturer. Proteins were tested using PBMCs obtained from three patients and the average ratio was calculated (the SFC of the target protein compared to the SFC of ESAT-6 in the kit). When the ratio was >20%, the test was continued for the next patient. If the ratio was <20%, detection was terminated. We used the ratio of SFCs of proteins of interest to those of ESAT-6 to evaluate cellular immune reactivity (Tables 3 and 4) and found that 189 of the 219 membrane proteins were positive (Fig. 3, light blue bars; each column represents the SFC ratio of an individual protein;Supplemental Table S1). The SFC ratio of the reference protein (the commercial ESAT-6 protein) is shown as a red column (Fig. 3). There were 8 membrane proteins with ratios above 50% (Fig. 3, purple bars). To further examine the 8 proteins, which exhibited more than half of the reactivity of ESAT-6, a second round screening was performed in parallel tests using 15 active TB^10^ ESAT-6 positive patients, 8 patients with an ESAT-6 stimulation response that was negative, and 5 healthy candidates (Table 3). The best accuracy was obtained for 2 of the 8 proteins(varying from 42.15 to 43.62%): Rv0232 and Rv1115. Strikingly, positive Rv0232 and Rv1115 stimulation response were observed using PBMCs obtained from 8 ESAT-6 negative patients (Fig. 4). Moreover, all of the SFC of proteins were below 15 in the 5 healthy candidates (Supplement Table S5).

**Table 3:** Characterization of patients according to the identification of proteins by ELISpot, which identified 219 membrane proteins.

Characteristics	E pulmonary TB patients
Total number	31
Age, mean (range, years)	34 (18–57)
Male/Female	18/13
Bacteriological test positive/negative/other	15/6/10
Outpatient/Hospitalized	6/25
Tuberculosis/Other	25/6
ELISPOT, Mean (range)	121.1 (31–232)

**Table 4:** Characterization of patients for the second-round identification of protein candidates by ELISpot; which yielded 8 membrane proteins.

Characteristics	E^+^ pulmonary TB patients	E^−^ pulmonary TB patients	Non-tuberculosis patients
Total Number	15	8	5
Age, mean (range, years)	35.0 (17–51)	41 (22–61)	28 (21–35)
Male/Female	12/3	4/4	3/2
Bacteriological test positive/negative	14/1	8/0	0/5
PCR positive/negative/other	13/1/1	3/1/4	5/0
ELISpot, mean (range)	93.7 (11–268)	20.5 (1–167)	3.7 (0–23)

### *In vivo* evaluation of antigenic properties in mice

**Figure 5:**
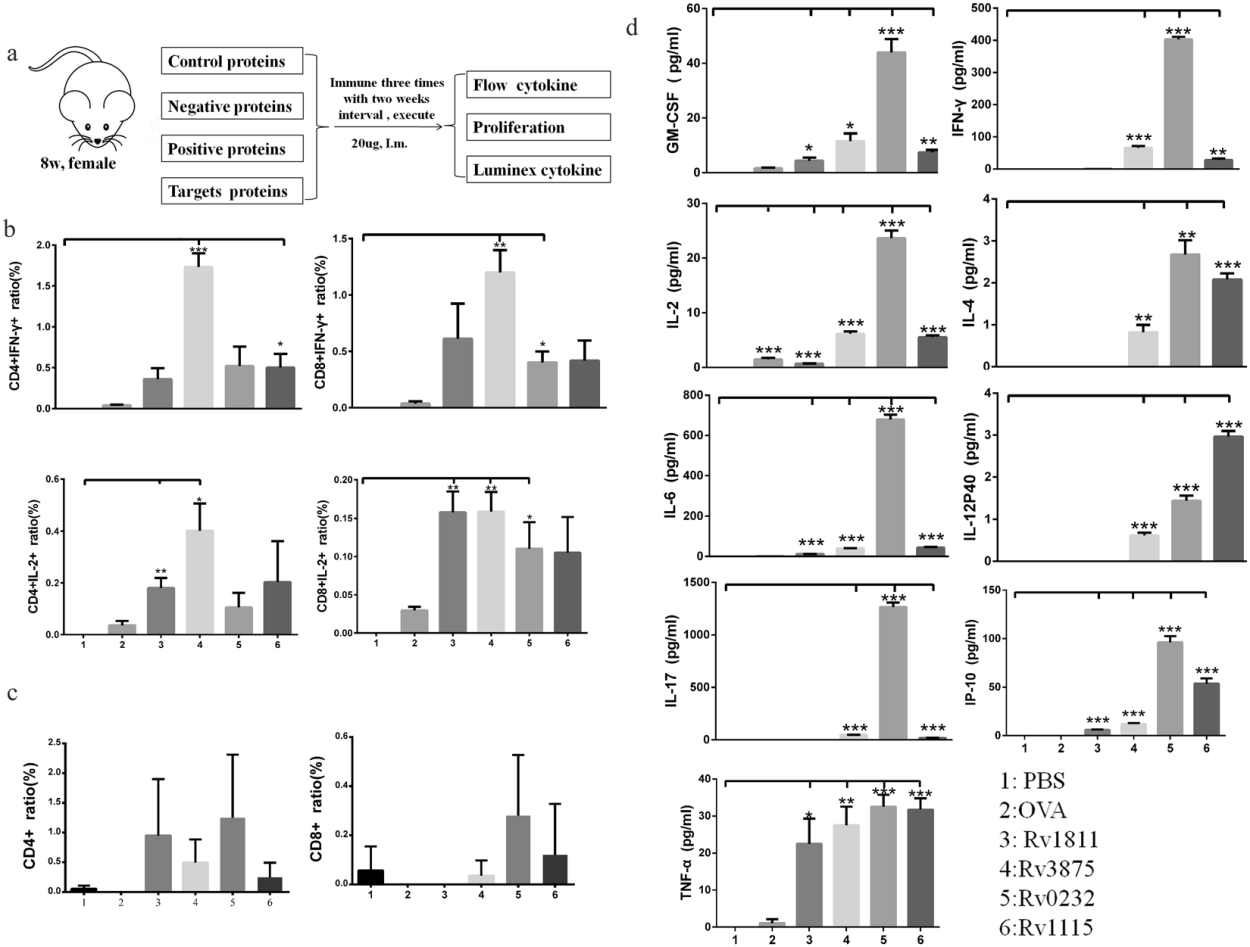
CD4^+^ and CD8^+^ T-cell intracellular IFN-γ and IL-2 detection, CD4^+^ and CD8^+^ T-cell proliferation and cytokine screening *in vivo*. (**a**) C57BL/6 mouse immunization procedure. (**b**) CD4^+^ and CD8^+^ T-cell intracellular IFN-γ and IL-2 detection. Mouse splenocytes obtained from different mouse immunization groups (n = 8 from each immunization group) were separated into duplicate samples: one sample for CD4^+^ and CD8^+^ T-cell intracellular IFN-γ and IL-2 detection and another sample was used for CD4^+^ and CD8^+^ T cell proliferation. Each antigen stimulation were triplicate to avid system error. (**c**) CD4^+^ and CD8^+^ T-cell proliferation after overnight antigen stimulation, as determined by FACS analysis. The plotted numbers reflect the percentage of CD4^+^ and CD8^+^ T-cells compared to those observed for controls. (**d**) Cytokine and chemokine tests. Cytokine and chemokine research plays a significant role in improving our understanding of the immune system and its multi-faceted response to most antigens.

Our studies aimed at identifying *M.tb* antigenic proteins that stimulate cell-specific IFN-γ secretion; as two membrane proteins (Rv0232 and Rv1115) showed better antigenicity, we evaluated the antigenicity of these two proteins *in vivo*. C57BL/6 mice were divided into six groups, Rv0232c, Rv1115, buffer control, OVA (blank control), Rv1811 (INF-γ negative control), and Rv3875 (INF-γ positive control), and immunized three times with different antigens or controls at two-week intervals (Fig. 5a). Mice were sacrificed 7–10 days after the third immunization, the spleen and lymph gland cells were collected, and lymphocytes were prepared for T-cell IFN-γ and IL-2 cytokine secretion assays as these two immune factors are related to infection and disease.

### Intracellular IFN-γ and IL-2 secretion from CD4 and CD8 cells

Interferon-γ (IFN-γ) release assays rely on the fact that T-lymphocytes release IFN-γ when exposed to specific antigens, including in TB, and interleukin-2 (IL-2) plays essential roles in key functions of the immune system. Thus, we chose IFN-γ and IL-2 as parameters to evaluate the antigenicity of expressed membrane protein. Significantly higher levels of IFN-γ expression in spleen CD4^+^ T-cells were found for cells obtained from mice immunized with Rv3875(positive control) compared to phosphate buffer saline(PBS, control samples, P = 0.0005), Rv1115 significantly enhanced IFN-γ secretion in spleen CD4^+^ T-cells. Additionally, CD8^+^ T cells from mice immunized with the Rv3875 and Rv0232 showed markedly higher levels of of cellular IFN-γ expression (P = 0.0038, P = 0.0134, respectively). However, neither of these proteins induced a significant increase in IL-2 secretion by CD4^+^ T-cells. In mice injected with Rv3875 and Rv1811, expression in IL-2 secretion by CD4^+^ T-cells (P = 0.0186, and P = 0.0100, respectively), and Rv3875 enhanced secretion of cytokines (P = 0.0036). Rv1811 and Rv0232 also significantly increased IL-2 expression in CD8^+^ cells (P = 0.0043, and P = 0.0337, respectively; Fig. 5b, Supplement Table S6, Fig. S7). Rv1811 significantly increasing IL-2 expression in CD4^+^ T-cells and CD8^+^ T-cells.

### Cell proliferation and cytokine assays

To evaluate proliferation, carboxyfluorescein succinimidyl ester (CFSE)-labelled lymphocytes were stimulated with Rv0232 and Rv1115 to promote CD4^+^/CD8^+^ T-cell proliferation. FACS analysis showed that Rv0232 dispayed a insignificant ability to activate CD4^+^ T-cell proliferation (Fig. 5c, Supplement Table S8, Fig. S9). We further examined levels of cytokine secretion from mice lymphocytes following stimulation with antigen candidates or controls by using a LUMINEX system to examine levels in the supernatants of spleen cells treated with or without antigen stimulation (Fig. 5d, Supplement Table S10). Most of the proteins tested, including Rv3875, Rv0232, Rv1115, and Rv1811, resulted in significantly higher levels of cellular cytokine secretion than were observed in the blank controls. Rv3875, Rv0232, and Rv1115 caused substantially higher levels of secretion of a wide range of inflammatory cytokines and chemokines, such as GM-CSF, INF-γ, IL-12P40, IL-17, IL-2, IP10, IL-4, IL-6, and TNF-α, compared to the group that received PBS injection. Rv1811 significantly induced secretion of IL-2, IL-6, IP10, and TNF-α. Interestingly, the ability of these proteins to induce antigenic cellular responses were variable. According to the cytokine secretion panel, every candidate protein was able to stimulate significant IFN-γ secretion.

## Discussion

More than 100 years have passed since its initial iderntification, and *M.tb* remains one of the most successful human pathogens. There is currently no single method of rapid method of diagnosis TB, especially when the sputum smear is negative. Accordingly, there is an urgent need to identify more accurate and sensitive *M.tb*-related biomarkers. Vaccines are an effective method of preventing disease. BCG is currently the only prophylactic TB vaccine avaiable, and evidence of its protection against *M.tb* infection remains controversial, particularly in adults^13,14^. To achieve the goal of eliminating TB, novel antigens must be identified, and new vaccination strategies must be developed. Unfortunately, the most promising candidate vaccine, (MVA85A) did not confer significant protection against TB or *M.tb* infection in a phase 2b trial^15^. Moreover, there are numerous unsolved problems related to the development of a new TB vaccine. *M.tb* membrane-associated proteins of are potent activators of human T-cell-responses^15,16^. To identify new antigens, we examined all membrane proteins in *M.tb in vitro* and *in vivo*.

Humoral antigenic was still a auxiliary means of diagnostics TB. However, Western blotting revealed a visible and specific band that could be quantified according to the grey value. In the present study, this approach used to evaluate the antigenicity of membrane proteins by comparing humoural responses with that of the commercial *M.tb* 38-kDa antigens (Rv0934). The antigenic and immunological properties of 18 proteins were confirmed in three rounds of screening, and the ratio of the results for target proteins to those for the positive control Rv0934 control remaining above 1. especially for Rv1111c (427–981), which had a mean ratio of 3.38. These data show that these proteins have distinct humoural antigenic properties, and suggest that they may represent potential biomarkers or vaccine candidates. Data obtained from humoral immune response experiments performed using serum from subjects without TB infection with or without BCG scars showed that a weakly positive antibody response. Although WHO has halted the use of humoural immunity to diagnose TB^17–19^, serological results suggest an active response to TB serum by 18 of the evaluated membrane proteins, with satisfactory performance. Thus, combinations of these membrane proteins may be used to obtain the sensitivity and specificity desired for diagnosis because the response intensity was much higher than that for other proteins. In the future, we will perform work to explore this issue.

The most significant result of this study is the emergence of membrane proteins as potent stimulators of human T-cell INF-γ and IL-2 secretion, proliferative responses, and multi-cytokine production. T-cells have been explored in numerous research efforts because of their importance in protecting against TB via secretion of high levels of IFN-γ, IL-2, and other cytokines. *M.tb*-secreted antigen target-6 (ESAT-6), Antigen 85 (Ag85), and culture filtrate protein-10 (CFP-10) elicit strong T cell responses in both mice and humans. Our antigen-specific IFN-γ secretion assay identified 8 membrane proteins (Rv0232, Rv1115, Rv0284, Rv1145, Rv0987, Rv1783, Rv3447c, and Rv1500) with marked stability and a significant response. Rv1115 and Rv0232 reacted with PBMCs from active patients, with a reaction ratio above 50% *in vitro*, and enhanced cytokines secretion *in vivo*.

To further evaluate protein immunogenicity, two candidate proteins were used as antigens to immunized mice. The two proteins were detected by two rounds of ELISpot,with ratio above 50% in the first round (supplement Table S1). In the second round, all proteins tested showed a suitable average intensity of reactivity and specificity (supplement Table S5). The C57BL/6 mouse is most commonly used as an *in vivo* model of *M. tuberculosis* infection, and purified Rv1115 significantly increase IFN-γ expression in spleen CD4^+^ T-cells *in vivo* (P = 0.0381), CD8^+^ T-cells from mice immunized with Rv0232 also had significantly higher levels of cellular IFN-γ expression in (P = 0.0134) than were observed in controls. These results show that Rv0232 and Rv1115 can enhance the Th1 immune response and may serve as candidates for the clinical diagnosis or vaccination against *M.tb*^20,21^.

IL-2 promotes T-cell replication and is essential for cellular immunity and granuloma formation. Here we presented candidate proteins with a higher capacity to stimulate IL-2 production. With the exception for the positive control, Rv3875 (P = 0.0186), which enhances cytokines secretion, we found that target proteins such as Rv1811 (P = 0.0100) induced higher expression levels of IL-2 in CD4^+^ T-cells. In addition, Rv1811 and Rv0232 also enhanced IL-2 expression in CD8^+^ cells (P = 0.0043, P = 0.0337, respectively). However, based on ELISpot, Rv1811 did not elicit IFN-γexpression when used as an antigen, indicating that this protein may serve as a biomarker for IL-2 detection. Future studies will be performed to explore this hypothesis. The finding that the initial protective immune response to *M.tb* following infection or vaccination can be subsequently lost is central to understanding the natural history of *M.tb* infection, and for exploring the rational design of TB vaccines.

In the antigen-specific proliferation test, Rv0232 and Rv1115 also increased T-cell proliferation; though the increase were not significant. TB is a cellular immune response-mediated disease, and antigens that specifically activate IFN-γ and CD8^+^ T-cells mediate the cytotoxic response. Our results are consistent with this observation, as the two proteins stimulated T-cell proliferation at different levels.

The interferon-γ release assay is a well-established tool for the diagnosing of TB and is based on the detection of T-cells sensitized to the *M.tb* antigens ESAT-6 and CFP-10. However, positive reations are not observed for ESAT6 and CFP-10 in clinical TB patients, with negative results for at least 20% of patients. Noetheless, the percentage of ESAT-6-responsive patients in studies conducted in low endemic countries (Denmark, USA, Germany, and Kuwait) ranged from 60% to 80%, yet reactivity is absent in healthy individuals^22–25^. Thus, it is likely that ESAT-6 contains multiple broadly recognized T-cell epitopes and may have the potential to induce responses in genetically diverse populations. The ESAT-6 and CPF10antigens have been combined to increase the sensitivity of detection, with relevance ratios of 73% and 84%^1^, respectively. Accordingly, researchers have focused on identifying novel *M.tb* antigens. The aim of the present study was to identify new candidates through comparison with ESAT-6 and CFP-10, as the two currently available commercial antigens, to replace them or overcome their insufficiency. Interestingly, Rv0232 and Rv1115 were identified, with positive responses by serum from patients with negative responses to the commercial antigens ESAT6 and CFP10 (Supplement Table S5).

In summary, efforts aimed at establishing a systemic *M.tb* membrane protein biomarker screen may result in a TB biobank that could be used *in vitro* and *in vivo* to detect both humoural and cellular reactivity. The data obtained in the present study may also impact TB diagnosis and vaccine development. This is particularly true because cell-mediated immunity reaction experiments as well as the *in vivo* experiments showed that Rv0232 and Rv1115 exhibit strong potential as biomarkers that may be useful for the diagnosis of TB, and as potential antigens for use in TB vaccine development.

## Methods

### Bacterial strains and plasmids

H37Rv genomic chromosomes were donated by Professor Zongde Zhang (Beijing Tuberculosis & Thoracic Tumor Research Institute, China). *E. coli* DH5α cells were used for plasmid construction, and the *E. coli* BL21 (DE3) (Novagen, Darmstadt, Germany) strain was used for protein expression. For membrane protein expression, the Gateway expression system^26,27^ (Invitrogen, Camarillo, American) was employed according the manufacturer’s instructions.

### Bioinformatics software analysis of the target membrane proteins

In total, approximately 4,000 protein sequences of the *M.tb* H37Rv genome were obtained from http://ftp.ncbi.nih.gov/genbank/genomes/Bacteria/ *Mycobacterium tuberculosis* H37Rv^28^. The primary sequences of the *M.tb* H37Rv ORFs that were used to identify membrane proteins were determined using publicly available algorithms: PSORT was used to predict subcellular localization (http://psort.hgc.jp/form.html) and TMHMM version 2 (http://www.cbs.dtu.dk/services/TMHMM/) was used to identify transmembrane α-helices (TMH). To avoid missing immunogenicity areas for easy exprission, the amino acids sequences were segmented into 90–900 lengths with overlap of 8 amino acids of each neighbouring segment.

### DNA cloning

All of the coding sequences for H37Rv were obtained from NCBI (NC_000962.3)^29^. Gateway Recombination Cloning Technology (Invitrogen) was used to amplify the membrane proteins according to the manufacturer’s instructions. Briefly, the target genes were amplified via attB PCR and constructed into the entry vector (pDONR221) and expression clone (pDEST17) using Gateway® BP and LR enzyme cloning.

### Protein Expression and Purification

The expression clone were transformed into *E. coli* BL21 (DE3) cells^26^ and expression was induced using standard methods. The next day, cells were collected by centrifugation and lysed using Bugbuster according to the manufacturer’s instructions. Purified proteins were examined by SDS-PAGE and quantified using a Thermo Scientific Pierce BCA Protein Assay Kit. Protein sequences were confirmed by mass-spectrometry(Thermo Scientific,Rockford, Germany) as previously described^23^. The protein concentration was 0.5 μg/μL, and Western Blotting and ELISPOT analyses were performed.

### Subject Inclusion Criteria

TB patients and healthy controls (no TB) were recruited from Shenzhen Third People’s Hospital. TB was diagnosed with a positive sputum smear and/or PCR result or when one or more positive results were obtained. The patient’ ages ranged from 18 and 60 years old. The candidates were free from other infectious diseases such as acquired immunodeficiency syndrome, syphilis, and hepatitis B. none of the patients (n = 156, Tables 1–3) were not using immunomodulatory drugs. Control donor samples were defined as (n = 125) (i) had no history of TB, no contact with TB patients, and no clinical symptoms of TB (n = 5); (ii) no BCG scar health sera (n = 60); or (iii) had BCG scar health sera and were from professor Gao Lei’s epidemiological study on tuberculosis sectioning (n = 60). All control donors were negative according to the PPD and IGRA tests^30^. PBMC cells and serum were obtained from TB patients or healthy controls after written informed consent was provided by all participants (No. 2012-007). This study was approved by and performed under the guidelines of the Ethical Committee of the Shenzhen-Hong Kong Institute of Infectious Disease, Shenzhen Third People’s Hospital. Written informed consent was directly obtained from each participant.

### Western Blotting

In the present study, we used Simple Western system (Santa Clara, CA, USA)^31^ to quantitate the absolute response to membrane proteins by patent serum according to the manufacturer’s instructions. First, a 0.2 μg was mixed with master mix (Protein Simple) to achieve a final concentration of 1× sample buffer in the presence of fluorescent molecular weight markers and 40 mM dithiothreitol (DTT), the sample was denatured at 99 °C for 5 minutes. Target proteins were immune-probed with primary antibodies (TB patient plasma**)**, followed by HRP-conjugated secondary antibodies. All antibodies were diluted using an antibody diluent (Protein Simple) at a 1:100 or 1:200 ratio. The 38-kDa Rv0934 protein (IMMUNO, Massachusetts, USA) as a positive control^32^. Digital image was analyzed with Compass software (Protein Simple), and the quantified data of the detected proteins are reported as the molecular weight and signal/peak intensity. Serum of 30 active TB patients were a group and mix used to detect. To advance to the second round screening, a peak area ratio of greater than 1 was required for a given protein. After the second round of screening, proteins with a ratio greater than 1 were tested in a third round of screening. If any test result was negative or zero, then the last result was considered negative, and the mean and variance were calculated twice or three times. All the membrane proteins were also verified in tested using five control candidates’ serum mixture.

### Endotoxin removal

Purified recombinant proteins were concentrated via a 0.22 µm 3 kDa column (Millipore, Merck KGaA, Darmstadt, Germany) and centrifuged at 10,000 g at 4 °C. The proteins were diluted in PBS to a final concentration of 800 ng/μL and each protein was detected using a Pierce LAL Chromogenic Endotoxin Quantitation Kit (GenScript, Piscataway, USA). The pharmacopoeia pharmacy intravenous or endotoxin biological product doses did not exceed a maximum amount of more than 5 EU per kilogram body weight. Additionally, 1 EU of the standard endotoxin EC-5 (100 pg) derived from *E. coli* O111: B4 had an activity of 120 pg. A total of <5.0 EU/mg of endotoxin was typically observed and subsequently used for animal immunization^33,34^.

### ELISpot

A cellular antigen-specific IFN-γ ELISPOT Assay (Dakewe Biotech Company, Shenzhen, China) was performed as previously described^35^. Briefly, the plates were seeded with 2.5 × 10^5^ of freshly separated PBMCs per well. Each individual sample was tested in parallel with a positive control (phytohemagglutinin), a negative control (blank and protein purification buffer), the ESAT-6 antigen from the *M. tb* infection detection ELISpot kit, and purified ESAT-6 protein obtained from our system. The final concentration of proteins was 10 μg/mL. After conventional incubation, the plates were developed following the manufacturer’s instructions. SFCs were counted using a BIOREADER® 4000 PRO-X (Biosys, Karben, Germany). The SFC number in the buffer control was subtracted from the number in all of the test wells. Assays were considered positive using the criteria for *M. tb* infection in the established kit.

### Mouse immunization

The C57BL/6 mice (6–8 weeks old) used in this study were raised under specific, pathogen-free conditions in our own breeding facilities. Eight mice from each group were administered intramuscular (i.m.) immunization without adjuvant at two points in their legs. The amount of protein administered per mouse was 20 μg^36,37^ or an equal volume (50 μL) of phosphate-buffered saline (PBS). Two boosters were subsequently administered two weeks apart^38^. All mice experimental procedures were performed in accordance with the Regulations for the Administration of Affairs Concerning Experimental Animals approved by the State Council of People’s Republic of China. The animal experiments were approved by the Animal Research Ethics Committee of Shenzhen Third People’s Hospital (No. 2012-A003).

### Intracellular IFN–γ, IL-2 assay

One week after the last immunization, the mice were sacrificed, and spleens and lymph glands were collected. Cells (5 × 10^5^ cells in 100 μL/well) were cultured in triplicate in 96-well plates in at 37 °C in 5% CO_2_ with or without 10 µg/ml of antigens. Phytohemagglutinin (PHA), 10 μg/ml (Sigma, Merck KGaA, Darmstadt, Germany) was used as the positive control, and PBS and ovalbumin (OVA) stimulation served as the negative controls. Each antigen or fraction was used at a final concentration of 10 µg/mL. Antigen-stimulated cells were cultured overnight. For the intracellular FACS assay, monensin (2 μL/well, eBioscience, Rockford, Germany), was used as a Golgi inhibitor and 1 µL of BD GolgiPlug was added to 1 mL of cell culture. The cells were collected within 12 hours of culture. For the FACS assay, cells were blocked using anti-CD16/anti-CD32 (BD Pharmingen, Sa Diego CA) antibodies; surface staining and intracellular staining were performed using anti-CD4 (2 μL/tube), anti-CD8 (2 μL/tube), FITC-conjugated anti-IFN-γ, and PE-conjugated anti-IL-2 antibodies. The results were analyzed by Flowjo7.6 software.

### Cell proliferation and Luminex analysis

Splenocytes were stained with CFSE (Cell Trace^TM^ CFSE Cell Proliferation Kit, Invitrogen, Camarillo, American) before antigen stimulation and incubated with or without antigens (10 μg/mL) in U-bottom 96-well plates. Cells treated with PBS or untreated cells and cells treated with a PHA cocktail (1 μg/mL) were used as negative and positive controls, respectively. After 72 hours of incubation the cells were stained with PE-anti-CD4 and APC-anti-CD8 antibodies (BD Pharmingen, San Diego, CA) and detected by FACS analysis in which SSC and FSC were used to gate lymphocytes. Proliferating CD4^+^ or CD8^+^ cells were identified based ona low intensity of CFSE compared to negative controls. Cells culture supernatant were collected and stored at −80 °C for multi-cytokine examination (GM-CSF, IFN-γ, IL-2, IL-4, IL-6, IL-12p40, IL-17, IP-10, and TNF-α) using a Luminex 200 (Millipore, KGaA, Darmstadt, Germany).

### Statistical analysis

Statistical analyses were performed using GraphPad Prism 6 (GraphPad software), SPSS, and xPONENT 3.1 software. The unparied Student’s *t*- test was used for statistical analysis. A P-value < 0.05 was considered statistically significant.

## Supplementary information

Supplementary Material
