# Supplementary material for: Analysis of the Antigenic Properties of Membrane Proteins of Mycobacterium tuberculosis
Source: Sci Rep. 2019 Feb 28;9:3042. doi: 10.1038/s41598-019-39402-z (PMC6395656; doi:10.1038/s41598-019-39402-z)
Supplement: Supplementary file 1 — Supplementary Material [file 41598_2019_39402_MOESM1_ESM.pdf]

Supplementary Information

Analysis of the Antigenic Properties of Membrane Proteins of *Mycobacterium tuberculosis*

Haifeng LI[1], Liguo Liu[1], Wei-jia Zhang[2], Xiaobing Zhang[1], Jianhua Zheng[1], Li Li[1], Xiuyun Zhu[3], Qianting Yang[3], Mingxia Zhang[3]， Haiying Liu[1] *, Xinchun Chen[4]*, Qi Jin[1]*

Contents:

Supplemental table S1,S2,S3,S5,S6,S8,S10

Supplemental fig S4,S7,S9

Membrane proteins were identified by Serological test and Celluar antigen test.

Locus tag	Product Length	Protein size	Position F	Position R	Serological test					Celluar antigen test				
					First	Second	Third	Average	StDev	First	Second	Third	Average	StDev
Rv0563,175-429	255	12.7	175	429	0.36	-	-	0.36	-	10.64%	-	-	10.64%	-
Rv0103c,1-315	279	13.5	1	279	0.58	-	-	0.58	-	33.33%	12.12%	0.40%	15.29%	0.17
Rv1508A,1-333	288	13.9	13	300	0.00	-	-	0.00	-	36.07%	3.77%	3.02%	14.28%	0.19
Rv0290,13-384	372	16.9	13	384	0.60	-	-	0.60	-	78.72%	5.88%	32.38%	39.00%	0.37
Rv1363c,385-783	396	17.8	1	396	1.35	0.00	-	0.00	0.95	32.79%	13.07%	1.89%	15.91%	0.16
Rv0969,715-1122	375	17.1	4	378	0.00	-	-	0.00	-	26.23%	3.52%	0.00%	9.92%	0.14
Rv1145,106-546	363	16.6	40	402	0.47	-	-	0.47	-	27.74%	86.90%	36.36%	50.34%	0.32
Rv1635c,1222-1668	447	19.7	1237	1662	0.00	-	-	0.00	-	-	-	-	-	-
Rv0402c,118-573	399	17.9	10	408	0.00	-	-	0.00	-	-	-	-	-	-
Rv0507,124-630	504	21.8	1	504	0.53	-	-	0.53	-	40.00%	34.43%	6.53%	26.99%	0.18
Rv1557,1-561	561	23.9	1	561	0.59	-	-	0.59	-	27.87%	23.45%	6.53%	19.28%	0.11
Rv0007,16-591	576	24.4	16	591	0.00	-	-	0.00	-	-	-	-	-	-
Rv0987,142-783	609	25.6	13	621	0.00	-	-	0.00	-	51.61%	55.74%	46.09%	51.15%	0.05
Rv1500,1-672	561	23.9	40	600	0.00	-	-	0.00	-	59.02%	41.30%	57.04%	52.45%	0.10
Rv0111,1282-2055	747	30.7	10	756	0.56	-	-	0.56	-	34.43%	7.55%	8.04%	16.67%	0.15
Rv1469,961-1785	777	31.8	13	789	0.57	-	-	0.57	-	54.10%	10.87%	4.02%	23.00%	0.27
Rv1239c,1-924	921	37.1	1	921	2.32	0.30	-	1.31	1.43	21.74%	0.00%	-	10.87%	0.15
Rv1783,256-1305	1026	40.9	13	1038	0.22	-	-	0.22	-	59.26%	54.10%	46.09%	53.15%	0.07
Rv0507,1192-2280	1083	43.0	4	1086	0.48	-	-	0.48	-	37.70%	3.02%	3.77%	14.83%	0.20
Rv0064,922-2937	1950	74.8	1	1950	5.15	0.50	-	2.83	3.29	10.71%	-	-	10.71%	-
Rv1459c,298-600	297	14.2	4	300	0.00	-	-	0.00	-	50.82%	6.00%	21.74%	26.19%	0.23
Rv0210,1150-1476	279	13.5	16	294	0.00	-	-	0.00	-	13.04%	-	-	13.04%	-
Rv0093c,13-381	369	16.8	13	381	0.58	-	-	0.58	-	0.00%	0.00%	0.00%	0.00%	0.00
Rv0713,170-645	297	13.5	22	318	0.00	-	-	0.00	-	63.83%	10.08%	37.14%	37.02%	0.27
Rv0226c,1159-1608	429	19.0	1	429	0.54	-	-	0.54	-	21.31%	2.51%	3.77%	9.20%	0.11
Rv1522c,127-639	459	20.1	10	468	0.18	-	-	0.18	-	8.54%	-	-	8.54%	-

Rv0987,1513-2166	653	27.3	4	653	0.35			0.35		26.23%	3.52%	5.66%	11.80%	0.13
Rv0206c,2158-2832	576	24.4	4	579	0.00	-	-	0.00	-	21.74%	0.00%	-	10.87%	0.15
Rv0528,538-1320	765	31.4	1	765	0.33	-	-	0.33	-	22.67%	4.52%	0.00%	8.06%	0.12
Rv1069c,640-1743	1104	43.8	640	1743	1.10	0.00	-	0.00	0.78	57.45%	3.36%	19.05%	26.62%	0.28
Rv1522c,1210-2475	1260	49.5	4	1263	1.27	0.00	-	0.00	0.90	21.31%	4.02%	5.66%	10.33%	0.10
Rv0284,1465-2805	1194	47.1	7	1200	0.67	-	-	0.67	-	76.19%	0.81%	8.48%	28.49%	0.41
Rv0876c,1-333	297	14.2	31	327	0.00	-	-	0.00	-	9.43%	-	-	9.43%	-
Rv0908,244-642	384	17.4	1	384	1.37	0.00	-	0.00	0.97	16.39%	-	-	16.39%	-
Rv0076c,1-291	288	13.9	1	288	0.53	-	-	0.53	-	-	-	-	-	-
Rv1795,1-405	387	17.5	1	387	0.71	-	-	0.71	-	18.03%	-	-	18.03%	-
Rv0412c,784-1227	393	17.7	31	423	0.00	-	-	0.00	-	55.74%	19.57%	4.52%	26.61%	0.26
Rv1431,1180-1767	549	23.4	13	561	1.09	0.00	-	0.00	0.77	39.34%	8.54%	1.89%	16.59%	0.20
Rv0051,244-540	297	14.2	244	540	0.13	-	-	0.13	-	59.57%	12.61%	20.00%	30.73%	0.25
Rv1811,373-702	258	12.8	412	669	0.83	-	-	0.83	-	22.16%	12.61%	0.00%	11.59%	0.11
Rv0982,142-483	321	15.1	151	471	1.13	0.31	-	0.72	0.58	38.92%	10.08%	45.71%	31.57%	0.19
Rv0019c,85-465	369	16.8	91	459	0.99	-	-	0.99	-	82.98%	0.00%	19.05%	34.01%	0.43
Rv0103c,688-1050	363	16.6	688	1050	0.71	-	-	0.71	-	44.68%	11.76%	26.67%	27.70%	0.16
Rv1030,313-714	387	17.5	322	708	0.37	-	-	0.37	-	13.33%	-	-	13.33%	-
Rv1739c,1231-1680	432	19.1	1	432	0.00	-	-	0.00	-	9.84%	-	-	9.84%	-
Rv1746,991-1422	432	19.1	991	1422	0.00	-	-	0.00	-	48.94%	1.68%	34.29%	28.30%	0.24
Rv0676c,160-609	432	19.1	1	432	0.44	-	-	0.44	-	29.51%	5.03%	3.77%	12.77%	0.15
Rv0450c,139-621	459	20.1	4	462	0.72	-	-	0.72	-	4.02%	-	-	4.02%	-
Rv0235c,970-1437	468	20.5	970	1437	0.00	-	-	-	-	-	-	-	-	-
Rv1522c,2893-3438	525	22.6	1	525	0.00	-	-	0.00	-	6.03%	-	-	6.03%	-
Rv1814,325-900	576	23.7	340	897	0.11	-	-	0.11	-	-	-	-	-	-
Rv0996,79-705	558	23.8	31	588	0.00	-	-	0.00	-	6.06%	-	-	6.06%	-
Rv0394c,88-717	579	24.5	1	579	0.00	-	-	0.00	-	26.23%	5.03%	0.00%	10.42%	0.14
Rv0845,619-1263	645	27.0	619	1263	0.50	-	-	0.50	-	65.96%	5.04%	43.81%	38.27%	0.31
Rv1319c,835-1605	723	29.8	34	756	1.10	0.00	-	0.00	0.78	24.59%	5.03%	1.89%	10.50%	0.12
Rv0931c,1207-1892	720	29.7	31	750	1.90	1.50	1.36	1.59	0.28	3.02%	-	-	3.02%	-

Rv0292,238-924	687	28.5	238	924	0.00	-	-	0.00	-	61.70%	2.52%	44.76%	36.33%	0.30
Rv0103c,1258-2115	807	32.9	4	810	0.22	-	-	0.22	-	-	-	-	-	-
Rv1348,1714-2577	819	33.3	4	822	0.96	0.00	-	0.00	0.68	22.58%	0.81%	9.52%	9.30%	0.11
Rv0969,1273-2151	858	34.8	1	858	1.32	0.40	-	0.86	0.65	4.85%	-	-	4.85%	-
Rv1320c,832-1701	816	33.2	34	849	0.52	-	-	0.52	-	40.98%	21.38%	11.56%	24.64%	0.15
Rv1348,31-813	783	32.0	31	813	0.83	-	-	0.83	-	49.52%	3.57%	6.72%	19.94%	0.26
Rv1349,793-1737	930	37.4	805	1734	0.11	-	-	0.11	-	22.62%	0.81%	11.52%	11.65%	0.11
Rv0014c,7-954	948	38.1	7	954	0.96	-	-	0.96	-	87.23%	2.52%	20.00%	36.59%	0.45
Rv1743,43-987	945	38.0	43	987	1.45	0.82	-	1.13	0.45	38.10%	0.00%	0.84%	12.98%	0.22
Rv1639c,427-1434	1008	40.3	427	1434	0.00	-	-	0.00	-	8.70%	-	-	8.70%	-
Rv0593,157-1206	1011	40.4	40	1050	0.00	-	-	0.00	-	9.43%	-	-	9.43%	-
Rv0450c,1213-2253	1041	41.5	1213	2253	3.19	1.03	1.26	1.83	1.19	80.85%	6.72%	15.24%	34.27%	0.41
Rv0284,2728-3990	1164	46.0	31	1194	0.46	-	-	0.46	-	60.87%	4.67%	-	32.77%	0.40
Rv1307,79-1317	1239	48.7	79	1317	2.53	0.00	-	0.00	1.79	-	-	-	-	-
Rv1183,1141-2412	1161	45.9	40	1200	1.06	0.60	-	0.83	0.33	5.53%	-	-	5.53%	-
Rv0284,202-1542	1341	52.5	202	1542	1.48	0.00	-	0.00	1.05	57.14%	60.00%	35.76%	50.97%	0.13
Rv0425c,1354-2715	1347	52.7	1	1347	0.90	-	-	0.90	-	54.35%	27.87%	1.89%	28.03%	0.26
Rv0236c,1270-2664	1356	53.0	19	1374	2.42	0.00	-	0.00	1.71	29.51%	5.03%	0.00%	11.51%	0.16
Rv0236c,2601-3882	3621	50.3	262	3882	0.00	-	-	0.00	-	21.57%	0.67%	0.00%	6.74%	0.12
Rv2163c,1-279	249	12.4	25	273	0.63	-	-	0.63	-	15.22%	-	-	15.22%	-
Rv2772c,184-471	255	12.7	10	264	0.00	-	-	0.00	-	13.04%	-	-	13.04%	-
Rv2843,253-543	285	13.8	1	285	0.00	-	-	0.00	-	1.21%	-	-	1.21%	-
Rv3252c,472-762	285	13.8	472	756	0.00	-	-	0.00	-	15.48%	-	-	15.48%	-
Rv2536,397-690	270	13.2	400	669	0.00	-	-	0.00	-	2.38%	-	-	2.38%	-
Rv3694c,1-303	285	13.8	13	297	0.00	-	-	0.00	-	52.17%	13.11%	5.66%	23.65%	0.25
Rv2209,1225-1536	306	14.5	4	309	0.00	-	-	0.00	-	26.09%	0.00%	0.00%	8.70%	0.15
Rv2025c,676-996	279	13.5	16	294	0.00	-	-	0.00	-	34.78%	6.56%	0.00%	13.78%	0.18
Rv3005c,1-336	300	14.3	7	306	0.00	-	-	0.00	-	0.00%	0.00%	0.00%	0.00%	0.00
Rv2874,19-348	330	15.4	19	348	0.20	-	-	0.20	-	-	-	-	-	-
Rv3448,1-357	348	16.1	4	351	0.98	-	-	0.98	-	3.77%	-	-	3.77%	-

Rv0638,1-375	336	15.6	1	336	0.00	-	-	0.00	-	15.22%	-	-	15.22%	-
Rv3843c,1-381	357	16.4	1	357	0.28	-	-	0.28	-	28.26%	0.00%	0.00%	9.42%	0.16
Rv2287,1237-1626	381	17.3	4	384	0.00	-	-	0.00	-	30.43%	1.33%	1.89%	11.22%	0.17
Rv3870,277-672	393	17.7	1	393	0.41	-	-	0.41	-	6.52%	-	-	6.52%	-
Rv3887c,1-402	390	17.6	1	390	1.15	0.00	-	0.00	0.82	6.64%	-	-	6.64%	-
Rv3166c,550-957	360	16.5	19	378	0.95	-	-	0.95	-	47.83%	0.67%	0.00%	16.16%	0.27
Rv1997,313-738	411	18.4	1	411	0.00	-	-	0.00	-	26.09%	2.00%	0.00%	9.36%	0.15
Rv2942,199-627	426	18.9	1	426	0.65	-	-	0.65	-	4.42%	-	-	4.42%	-
Rv3743c,385-774	390	17.6	385	774	0.00	-	-	0.00	-	70.64%	22.69%	52.38%	48.57%	0.24
Rv1992c,679-1131	426	18.9	19	444	0.00	-	-	0.00	-	78.26%	17.39%	13.79%	36.48%	0.36
Rv3764c,1-456	423	18.8	34	456	1.22	0.00	-	0.00	0.86	19.57%	-	-	19.57%	-
Rv3823c,199-657	453	19.9	1	453	0.51	-	-	0.51	-	5.45%	-	-	5.45%	-
Rv3435c,130-537	408	18.3	130	537	0.46	-	-	0.46	-	34.04%	0.00%	31.43%	21.82%	0.19
Rv2219,301-720	420	18.7	301	720	0.51	-	-	0.51	-	74.47%	2.52%	28.57%	35.19%	0.36
Rv3738c,472-945	453	19.9	4	456	0.00	-	-	0.00	-	15.15%	-	-	15.15%	-
Rv2625c,688-1179	468	20.5	4	471	0.72	-	-	0.72	-	15.48%	-	-	15.48%	-
Rv3737,1-492	429	19.0	16	444	0.30	-	-	0.30	-	16.36%	-	-	16.36%	-
Rv3794,103-612	483	21.0	10	492	0.77	-	-	0.77	-	25.00%	5.75%	8.48%	13.08%	0.10
Rv2743c,298-810	480	20.9	1	480	0.87	-	-	0.87	-	10.30%	-	-	10.30%	-
Rv3000,1-525	516	22.2	4	519	0.00	-	-	0.00	-	15.15%	-	-	15.15%	-
Rv3695,400-930	459	20.1	22	480	0.00	-	-	0.00	-	16.36%	-	-	16.36%	-
Rv3792,1396-1929	507	21.9	19	525	0.33	-	-	0.33	-	6.06%	-	-	6.06%	-
Rv3104c,382-924	456	20.0	394	849	1.65	0.45	-	1.05	0.85	13.10%	-	-	13.10%	-
Rv3793,169-672	504	21.8	169	672	0.63	-	-	0.63	-	42.55%	0.84%	12.38%	18.59%	0.22
Rv0286,1054-1539	486	21.1	1054	1539	0.06	-	-	0.06	-	-	-	-	-	-
Rv1111c,427-981	549	23.4	4	552	4.50	4.00	1.64	3.38	1.53	6.67%	-	-	6.67%	-
Rv2195,703-1287	585	24.8	703	1287	0.87	-	-	0.87	-	40.00%	10.81%	0.00%	16.94%	0.21
Rv2869c,358-978	600	25.3	10	609	0.00	-	-	0.00	-	5.75%	-	-	5.75%	-
Rv2903c,262-882	621	26.1	1	621	0.63	-	-	0.63	-	6.19%	-	-	6.19%	-
Rv3239c,2524-3144	621	26.1	1	621	0.00	-	-	0.00	-	6.19%	-	-	6.19%	-

Rv2194,163-768	606	25.5	163	768	1.39	0.00	-	0.00	0.98	31.91%	1.68%	0.00%	11.20%	0.18
Rv3200c,388-1059	672	27.9	388	1059	0.76	-	-	0.76	-	14.89%	-	-	14.89%	-
Rv3805c,1168-1881	657	27.4	10	666	0.33	-	-	0.33	-	5.31%	-	-	5.31%	-
Rv3365c,193-963	747	30.7	1	747	0.61	-	-	0.61	-	2.65%	-	-	2.65%	-
Rv3645,841-1647	798	32.6	4	801	0.31	-	-	0.31	-	4.85%	-	-	4.85%	-
Rv3743c,940-1791	786	32.1	40	825	0.57	-	-	0.57	-	3.64%	-	-	3.64%	-
Rv2601,712-1569	834	33.9	16	849	0.00	-	-	0.00	-	10.30%	-	-	10.30%	-
Rv3428c,262-1140	855	34.7	25	879	0.23	-	-	0.23	-	3.10%	-	-	3.10%	-
Rv2284,286-1293	924	37.2	31	954	0.00	-	-	0.00	-	2.42%	-	-	2.42%	-
Rv2874,1090-2067	978	39.2	1090	2067	0.80	-	-	0.80	-	59.57%	5.88%	43.81%	36.42%	0.28
Rv3239c,1402-2454	1041	41.5	1	1041	1.09	0.40	-	0.75	0.49	1.21%	-	-	1.21%	-
Rv2942,1222-2277	1005	40.2	22	1026	1.69	0.00	-	0.00	1.20	3.03%	-	-	3.03%	-
Rv2587c,97-1152	1035	41.3	16	1050	1.87	1.10	1.35	1.44	0.39	1.82%	-	-	1.82%	-
Rv2339,1216-2307	1092	43.3	1	1092	4.50	0.00	-	0.00	3.18	3.64%	-	-	3.64%	-
Rv3793,2167-3282	1083	43.0	1	1083	0.47	-	-	0.47	-	3.10%	-	-	3.10%	-
Rv3835,199-1347	1095	43.5	22	1116	0.00	-	-	0.00	-	3.03%	-	-	3.03%	-
Rv1854c,1-1152	1098	43.6	1	1098	1.14	0.00	-	0.00	0.80	3.03%	-	-	3.03%	-
Rv2921c,82-1266	1158	45.8	94	1251	1.01	0.50	-	0.75	0.36	1.21%	-	-	1.21%	-
Rv3447c,235-1470	1212	47.7	250	1461	1.50	0.39	-	0.94	0.79	40.61%	52.00%	59.82%	50.14%	0.10
Rv0169,103-1362	1236	48.6	124	1359	3.59	1.30	1.23	2.04	1.34	6.06%	-	-	6.06%	-
Rv3894c,2887-4188	1284	50.4	7	1290	2.64	1.12	1.18	1.65	0.86	3.03%	-	-	3.03%	-
Rv3479,1378-2739	1362	53.2	1378	2739	0.00	-	-	-	-	27.57%	5.88%	51.43%	28.29%	0.23
Rv3554,640-2055	1308	51.3	661	1968	1.05	0.00	-	0.00	0.74	1.77%	-	-	1.77%	-
Rv2326c,652-2091	1371	53.6	40	1410	0.50	-	-	0.50	-	23.21%	12.39%	21.69%	19.10%	0.06
Rv3479,1-1455	1440	56.1	16	1455	0.46	0.29	-	0.37	0.12	65.06%	3.98%	9.52%	26.19%	0.34
Rv3870,796-2187	1392	54.3	796	2187	1.78	0.00	-	0.00	1.26	70.21%	1.68%	46.67%	39.52%	0.35
Rv3365c,1033-2628	1524	59.2	10	1533	1.60	1.20	1.38	1.39	0.20	59.04%	6.64%	1.19%	22.29%	0.32
Rv0151c,1-1659	1584	61.4	43	1626	0.10	-	-	0.10	-	-	-	-	-	-
Rv2748c,910-2547	1638	63.4	1819	3456	0.92	-	-	0.92	-	44.68%	9.24%	27.62%	27.18%	0.18
Rv3610c,430-2241	1812	69.7	430	2241	4.09	0.00	-	0.00	2.90	61.70%	2.52%	17.14%	27.12%	0.31

Rv3193c,919-2976	2052	78.5	4	2055	0.38	-	-	0.38	-	31.33%	3.10%	3.57%	12.66%	0.16
Rv0083,1558-1857	297	14.2	1	297	0.00	-	-	0.00	-	37.35%	2.65%	2.98%	14.33%	0.20
Rv0092,1255-2082	828	33.7	1255	2082	0.16	-	-	0.16	-	-	-	-	-	-
Rv0176,574-966	393	17.7	574	966	1.35	0.00	-	0.00	0.96	48.94%	4.20%	32.38%	28.51%	0.23
Rv0200,292-687	396	17.8	1	396	1.06	0.00	-	0.00	0.75	24.10%	1.33%	1.19%	8.87%	0.13
Rv0206c,103-555	453	19.9	1	453	0.66	-	-	0.66	-	45.78%	0.88%	2.38%	16.35%	0.26
Rv0283,283-1614	1323	51.8	10	1332	1.89	1.91	1.51	1.77	0.23	25.22%	3.10%	1.19%	9.84%	0.13
Rv0312,952-1332	381	17.3	1	381	0.65	-	-	0.65	-	9.57%	-	-	9.57%	-
Rv0338c,694-2646	1938	74.4	4	1941	0.86	-	-	0.86	-	-	-	-	-	-
Rv0362,1-870	870	35.2	1	870	1.49	0.00	-	0.00	1.05	20.00%	2.38%	0.88%	7.76%	0.11
Rv0412c,175-612	435	19.3	4	438	1.22	0.00	-	0.00	0.86	6.09%	-	-	6.09%	-
Rv0431,127-492	366	16.7	1	366	0.66	-	-	0.66		10.43%	-	-	10.43%	-
Rv0497,1-681	675	28.1	7	681	1.92	2.03	1.02	1.66	0.55	7.83%	-	-	7.83%	-
Rv0676c,1234-2310	1077	42.8	1234	2310	1.79	0.00	-	0.00	1.27	43.81%	4.76%	14.29%	20.95%	0.20
Rv0686,211-795	585	24.8	211	795	0.00	-	-	0.00	-	38.10%	14.89%	2.52%	18.50%	0.18
Rv0816c,70-420	342	15.8	10	351	0.47	-	-	0.47	-	8.70%	-	-	8.70%	-
Rv0901,91-525	435	19.3	91	525	0.78	-	-	0.78	-	87.23%	0.84%	14.29%	34.12%	0.46
Rv0969,1-420	420	18.7	4	414	0.11	-	-	0.11	-	-	-	-	-	-
Rv0982,553-1512	954	38.3	7	960	0.51	-	-	0.51	-	39.13%	1.19%	2.21%	14.18%	0.22
Rv1115,94-696	597	25.2	4	600	0.00	-	-	0.00	-	54.55%	44.16%	67.39%	55.37%	0.12
Rv1132,1165-1728	564	24.0	1	564	0.37	-	-	0.37	-	26.51%	0.00%	5.36%	10.62%	0.14
Rv1318c,835-1623	780	31.9	10	789	0.41	-	-	0.41	-	40.96%	0.83%	4.76%	15.52%	0.22
Rv1565c,1297-2187	873	35.3	19	891	0.81	-	-	0.81	-	22.89%	5.95%	1.24%	10.03%	0.11
Rv1621c,811-1581	762	31.2	10	771	1.43	1.30	0.35	1.03	0.59	28.92%	10.71%	0.00%	13.21%	0.15
Rv1635c,1-309	309	14.6	1	309	0.47	-	-	0.47	-	30.95%	36.14%	2.07%	23.05%	0.18
Rv1736c,1-1257	1230	48.4	28	1257	0.99	-	-	0.99	-	57.83%	8.93%	0.41%	22.39%	0.31
Rv1743,1078-1698	621	26.1	1	621	3.10	1.84	2.19	2.38	0.65	49.40%	10.12%	0.00%	19.84%	0.26
Rv1797,199-1218	1020	40.7	1	1020	0.35	-	-	0.35	-	33.73%	6.55%	0.00%	13.43%	0.18
Rv0062,214-1140	912	36.7	13	924	2.26	0.88	-	1.57	0.97	30.12%	7.14%	0.00%	12.42%	0.16
Rv1625c,607-1329	720	29.7	4	723	1.43	0.00	-	0.00	1.01	28.92%	4.17%	0.00%	11.03%	0.16

Rv1819c,1063-1917	849	34.4	7	855	0.57	-	-	0.57	-	32.53%	6.55%	2.48%	13.85%	0.16
Rv1845c,373-843	471	20.6	373	843	0.28	-	-	0.28	-	41.90%	0.00%	1.19%	14.37%	0.24
Rv1877,1504-2061	558	23.8	1	558	0.00	-	-	0.00	-	57.83%	7.14%	0.83%	21.93%	0.31
Rv1973,79-480	402	18.0	69	402	0.00	-	-	-	-	-	-	-	-	-
Rv1997,919-2100	1182	46.6	1	1182	0.26	-	-	0.26	-	56.63%	3.57%	2.27%	20.82%	0.31
Rv2154c,1216-1572	315	14.9	43	357	1.85	1.67	0.25	1.26	0.87	34.94%	5.36%	0.83%	13.71%	0.19
Rv2195,1-288	288	13.9	1	288	0.51	-	-	0.51	-	34.94%	6.55%	0.41%	13.97%	0.18
Rv2200c,430-1077	648	27.1	430	1077	0.99	-	-	0.99	-	22.69%	1.19%	6.34%	10.07%	0.11
Rv2236c,217-639	414	18.5	7	420	0.77	-	-	0.77	-	45.78%	4.76%	0.00%	16.85%	0.25
Rv2262c,643-1080	411	18.4	28	438	0.88	-	-	0.88	-	12.05%	-	-	12.05%	-
Rv2306A,316-591	276	13.4	316	591	0.00	-	-	0.00	-	13.25%	-	-	13.25%	-
Rv2339,151-600	450	19.8	151	600	2.55	0.82	-	1.68	1.23	7.80%	-	-	7.80%	-
Rv2345,85-483	399	17.9	85	483	0.80	-	-	0.80	-	14.29%	-	-	14.29%	-
Rv2443,1108-1473	354	16.3	13	366	0.99	-	-	0.99	-	59.04%	1.79%	0.41%	20.41%	0.33
Rv2643,1072-1494	423	18.8	1072	1494	0.78	-	-	0.78	-	11.90%	-	-	11.90%	-
Rv2673,25-333	309	14.6	25	333	0.51	-	-	0.51	-	5.88%	-	-	5.88%	-
Rv2690c,1498-1971	474	20.7	1498	1971	0.44	-	-	0.44	-	13.10%	-	-	13.10%	-
Rv2748c,7-444	438	19.4	7	444	0.00	-	-	0.00	-	10.92%	-	-	10.92%	-
Rv3252c,910-1248	339	15.7	1	339	0.00	-	-	0.00	-	50.60%	2.38%	0.83%	17.94%	0.28
Rv3764c,571-1425	855	34.7	571	1425	0.00	-	-	0.00	-	2.42%	-	-	2.42%	-
Rv3779,1558-1995	438	19.4	1558	1995	0.48	-	-	0.48	-	3.57%	-	-	3.57%	-
Rv3869,196-1434	1239	48.7	196	1434	1.38	0.22	-	0.80	0.82	39.76%	16.67%	2.07%	19.50%	0.19
Rv3877,13-426	414	18.5	13	426	0.79	-	-	0.79	-	3.57%	-	-	3.57%	-
Rv3894c,265-1653	1389	54.2	19	1389	0.58	-	-	0.58	-	-	-	-	-	-
Rv3921c,790-1098	309	14.6	790	1098	1.03	0.17	-	0.60	0.61	1.46%	-	-	1.46%	-
Rv0008c,52-303	252	12.5	52	303	0.00	-	-	0.00	-	3.00%	-	-	3.00%	-
Rv0392c,181-1413	1233	48.5	181	1413	0.09	-	-	0.09	-	2.00%	-	-	2.00%	-
Rv0758,100-1443	1344	52.6	100	1443	0.25	-	-	0.25	-	6.50%	-	-	6.50%	-
Rv0774c,64-795	732	30.1	64	795	0.54	-	-	0.54	-	3.60%	-	-	3.60%	-
Rv1004c,94-885	792	32.3	94	885	0.08	-	-	0.08	-	4.20%	-	-	4.20%	-

All membrane proteins identified by MS/MS analysis: The nanoACQUITY was controlled by MassLynx version 4.1. Samples of 6 μL were loaded on a C18 precolumn (Symmetry C18, 5 μm, 180 μm X 20 mm) at 10 μL/min in 5% acetonitrile, 0.05% FA for 5 min, followed by separation using a C-18 analytical column (Waters ACQUITY UPLC BEH130 C18, 1.7 μm, 100 μm X 100 mm) at a constant temperature (35°C). A linear gradient of 5 to 35% solvent B over a 40 min period (solvent A, 0.1% formic acid (FA), 3% acetonitrile; solvent B, 97% acetonitrile in 0.1% FA) at a flow rate of 400 nL/min was applied. The peptides were analyzed in the positive ion mode by electrospray ionization (the spray voltage = 2.0 kV). MS data were acquired by a survey scan (m/z 300-1,800, R=60,000 at m/z 400) in the Orbitrap using the lock masses (m/z=445.120025) for internal recalibration in real time (21). The ten most intense peaks for fragmentation in the LTQ were selected with a normalized collision energy value of 35%, dynamic exclusion duration of 30 sec, and ion selection threshold of 500 counts. Precursors with unmatched and single charge states were discarded during data-dependent acquisition. Raw data were acquired using the Xcalibur software v2.2.6 (Thermo). Searching database: 3,988 entries of H37Rv genome and 247 known contaminant protein sequences.Database searching program: MASCOT version 2.2.The following search parameters were applied: max missed cleavage: 1; fixed modification: Carbamidomethylation (C); variable modification: Oxidation (M);Carbamyl (N-term), Deamidated (NQ); Precursor ion mass tolerance: ±50 ppm; fragment mass tolerance: ±0.6 Da. High peptide confidence (p<0.01) and the false positive rate (FPR) was controlled at under 1%, missed cleavages permitted: 2

locus tag	Accession	Description	Score	Coverage	Proteins	Unique Peptide	Peptides	PSMs	AAs	MW [kDa]	calc. pI	Sequence	PSMs	Proteins	Protein Gr
Rv0563,1	15607703	heat shock	956.6155	24.48	1	5	5	28	286	30.66212	9.891113				
												AVLGHEI	6	1	1
												NAAVccT	2	1	1
												AmHAQP	10	1	1
												ELATSAH	4	1	1
												LYISDTA	2	1	1
												AMHAQP	4	1	1
Rv0103c,	15607245	PROBABI	1709.681	7.71	1	5	5	35	752	77.46085	7.29834				
												LDVLGm	7	1	1
												VATIDAV	2	1	1
												LDVLGM	5	1	1
												IRLDVLG	5	1	1
												ASVNFAT	5	1	1
												IRLDVLG	6	1	1
												aSVNFAT	2	1	1
												VATIDAV	2	1	1
												AAPVVGI	1	1	1
Rv1508A	57116880	hypothetic	1484.56	58.33	1	7	7	40	120	13.34061	8.938965	GYVAAG	3	1	1
												ELYGLLA	13	1	1
												NYRELYC	17	1	1
												cADADAI	1	1	1

												HGETFmT	1	1	1
												GADRcAI	3	1	1
												HGETFMT	1	1	1
												HGETFmT	1	1	1
Rv0290,1	15607431	PROBABI	710.3699	6.14	1	5	5	27	472	47.91373	8.880371	LTEMALI	2	1	1
												LTEmALP	14	1	1
												VAILADS	3	1	1
												EILPAVQ	2	1	1
												LTEMALI	1	1	1
												LTEmALP	3	1	1
												VAILADS	2	1	1
Rv1363c,	15608503	POSSIBLI	1781.495	30.27	1	7	7	60	261	28.29089	4.843262	VVTEGTV	6	1	1
												QGVINMT	1	1	1
												VIDSSTGI	5	1	1
												AAAFAA	2	1	1
												VIDSSTGI	43	1	1
												VTNSAG	1	1	1
												vIDSSTGI	1	1	1
												DDFQQR	1	1	1
Rv0969,7	15608109	PROBABI	3537.418	12.73	1	7	7	54	770	80.05293	9.085449	VAGATVI	20	1	1
												ATAVGA	17	1	1
												AAVDESm	3	1	1
												AAVDESM	3	1	1
												IPVDGEV	2	1	1
												ATAVGA	1	1	1
												EATLLVI	4	1	1
												VRPGEKI	3	1	1
												vRPGEKII	1	1	1
Rv1145,1	15608285	PROBABI	4646.512	37.29	1	10	10	95	303	32.52512	8.265137	MLIVVTA	21	1	1
												mLIVVTA	3	1	1
												AGGSAmI	14	1	1
												AGGSAM	27	1	1
												GGENDA	5	1	1
												EVGTDIV	15	1	1
												SGLIVVN	1	1	1
												GGENDA	2	1	1

												SPLVYNV	2	1	1
												AIEVLTD	1	1	1
												eVGTDIV	1	1	1
												EVGTDIV	1	1	1
												aGGSAMI	1	1	1
												RSPLVYN	1	1	1
Rv1635c,	15608773	Probable c	741.8994	14.75	1	9	9	19	556	59.99709	9.817871	SLPDHQV	4	1	1
												SLPDHQV	4	1	1
												DKSLPDH	2	1	1
												SLIDVER	1	1	1
												cPTLWTL	1	1	1
												SLIDVER	1	1	1
												ALLATRF	1	1	1
												WQFHYS	2	1	1
												TPVYRFP	2	1	1
												FPSYLGF	1	1	1
Rv0402c,	15607453	CONSERV	91.7189	4.19	1	1	1	2	620	64.10392	5.262207	RPSPLPTI	2	1	1
Rv0507,1	15607648	PROBABI	4180.997	14.05	1	9	9	91	968	106.1333	7.151855	HVAHIQI	7	1	1
												AYVTGPA	1	1	1
												VGQVFGI	15	1	1
												AHSVSMS	2	1	1
												AHSVSmS	2	1	1
												AAYVVV	45	1	1
												aAYVVVY	1	1	1
												HMVDTT	3	1	1
												AHSVSmS	1	1	1
												AHSVSMS	1	1	1
												hmVDTTF	1	1	1
												FYSDLmF	3	1	1
												HmVDTTI	1	1	1
												FYSDLMI	5	1	1
												hVAHIQD	1	1	1
												fYSDLMR	1	1	1
												fYSDLmR	1	1	1
Rv1557,1	15608695	PROBABI	1014.575	38.79	1	7	7	23	397	42.39468	6.41748	GTPmAGA	1	1	1

												SVFDTID	3	1	1
												ELALANY	1	1	1
												mIISHEGI	4	1	1
												LAAIQPQ	5	1	1
												MIISHEGI	6	1	1
												LFLSADC	1	1	1
												NDDSFYI	2	1	1
Rv0007,1	15607149	POSSIBLI	611.682	27.96	1	9	9	20	304	31.05394	8.865723	FISGASAI	7	1	1
												AATGPGI	1	1	1
												GDGPNAI	1	1	1
												APQRNPA	2	1	1
												GDSAAGS	1	1	1
												IPDAGDP	4	1	1
												NPAPARF	1	1	1
												APQRNPA	2	1	1
												NPAPARF	1	1	1
Rv0987,1	15608127	PROBABI	4376.6	17.78	1	14	14	90	855	93.54572	9.83252	LSDLIIAD	2	1	1
												LVSGPAC	21	1	1
												AMQFMC	8	1	1
												AMQFmG	6	1	1
												HGYDFSA	19	1	1
												LSDLIIAD	2	1	1
												AmQFmG	5	1	1
												LSHIDLII	8	1	1
												lVSGPAG	1	1	1
												AFDLSAA	1	1	1
												GAPTTVT	1	1	1
												hGYDFSA	3	1	1
												RHGYDFS	2	1	1
												LVSGPAC	4	1	1
												aMQFMG	1	1	1
												LPAGVRN	2	1	1
												YGLAVGI	1	1	1
												AFDLSAA	1	1	1
												LSHIDLII	1	1	1
												IQQRLPA	1	1	1

Rv1500,1	15608638	PROBABI	871.7435	21.35	1	8	8	27	342	38.07082	8.616699	LISTGAD	2	1	1
												aSALLcDI	2	1	1
												ASALLcD	6	1	1
												NFGPKIH	2	1	1
												LmTADY	3	1	1
												LMTADY	6	1	1
												LMTADY	2	1	1
												GTTTYTF	1	1	1
												LmTADY	1	1	1
												RPGGWL	1	1	1
												SLVQHQI	1	1	1
Rv0111,1	15607253	POSSIBLI	4115.276	27.15	1	15	15	102	685	74.3256	10.63818	DQPDVAI	16	1	1
												ALSIVGS	18	1	1
												WTHIGDI	21	1	1
												SDGVHL	2	1	1
												EIGLPPG	8	1	1
												WSAQVN	1	1	1
												DPNRPFT	6	1	1
												FIDHTVI	6	1	1
												YIGQTLE	5	1	1
												DPNRPFT	6	1	1
												VmVTTV	2	1	1
												aLSIVGST	1	1	1
												GTPYRYI	1	1	1
												WSAQVN	2	1	1
												AEcDGWI	2	1	1
												WETVDR	2	1	1
												VMVTTV	1	1	1
												LYPEDQF	2	1	1
Rv1469,9	15608607	PROBABI	2478.672	19.63	1	11	11	49	657	67.84259	6.63916	LLQLAA	23	1	1
												NLQAGG	4	1	1
												LADTSIV	2	1	1
												NLQAGG	2	1	1
												RLLQLAA	2	1	1
												AVPGcG	3	1	1
												LASVAPL	8	1	1

											NAGITDV	1	1	1
											AALLPEQ	1	1	1
											RGIAIPPA	1	1	1
											AALLPEQ	1	1	1
											RGIAIPPA	1	1	1
Rv1239c,	15608379	POSSIBLE	1161.515	37.98	1	14	14	29	366	41.47412	5.757324 DVADHQ	4	1	1
											DVADHQ	1	1	1
											VGMQQN	1	1	1
											TGEIMIFV	1	1	1
											TGEImIFV	1	1	1
											TVNYVPI	2	1	1
											MDADPE	3	1	1
											cVNPLST	3	1	1
											VGmQQN	2	1	1
											YDETLFL	1	1	1
											HGEHGG	4	1	1
											YLRDVAI	1	1	1
											YTYAAA	1	1	1
											LPGKYTY	1	1	1
											RcVNPLS	1	1	1
											DFVVTVI	1	1	1
											KLDIEPIY	1	1	1
Rv1783,2	15608921	PROBABI	1869.017	32.18	1	13	13	39	435	48.5543	5.414551 EFAAEQA	13	1	1
											ETAQESA	1	1	1
											WFHPAPI	4	1	1
											ETAQESA	1	1	1
											TGVIEAL	1	1	1
											mVSLLVI	1	1	1
											AQFMLm	2	1	1
											AQFMLm	1	1	1
											AQFMLM	1	1	1
											AQFmLml	1	1	1
											LAEAYEI	5	1	1
											MVSLLVI	1	1	1
											DLNFGVV	1	1	1
											WLPHFGI	1	1	1

												YQSVVY	1	1	1
												EQVLGL	1	1	1
												MVYTSV	1	1	1
												EQVLGL	1	1	1
Rv0507,1	15607648	PROBABI	4377.495	29.44	1	24	24	117	968	106.1333	7.151855	DRIDDIL	1	1	1
												IDDILVQ	3	1	1
												DRIDDIL	1	1	1
												DRIDDIL	1	1	1
												VFDAAQ	4	1	1
												GTPLQG	2	1	1
												IDDILVQ	5	1	1
												MYALM	2	1	1
												FVIALEG	11	1	1
												SIFDMFD	1	1	1
												AQmPPm	3	1	1
												FmPASM	2	1	1
												MYALmG	2	1	1
												mYALmG	2	1	1
												LNPEVLL	4	1	1
												VFDAAQ	2	1	1
												LNPEVLL	3	1	1
												IDDILVQ	2	1	1
												mYALmG	6	1	1
												FMPASM	1	1	1
												AQmPPM	8	1	1
												NPVDmL	1	1	1
												KFmPASm	2	1	1
												AQMPPM	3	1	1
												NPVDML	1	1	1
												DHLADF	14	1	1
												DMMLIW	2	1	1
												DMDILIT	2	1	1
												AQMPPM	2	1	1
												LNPEVLL	1	1	1
												FmPASm	2	1	1
												AQMPPm	4	1	1

												LNPEVLL	1	1	1
												HcFDVPL	2	1	1
												AITRPLG	1	1	1
												DMDILIT	2	1	1
												EAIKGTP	1	1	1
												mYALmG	2	1	1
												NIYHSPG	2	1	1
												TTYDLR	2	1	1
												DmDILITI	1	1	1
												LNPEVLL	1	1	1
												LEYLVK	1	1	1
Rv0064,9	15607206	hypothetic	8078.089	45.45	1	36	36	199	979	107.3021	7.649902	YTYTGA	3	1	1
												VGYAPTI	16	1	1
												ATVDAY	1	1	1
												ISTSPSSS	10	1	1
												VYYGEV	17	1	1
												NFFSFAE	4	1	1
												IDGELQD	12	1	1
												IVWIVDA	21	1	1
												LASAmV	4	1	1
												SSLEGPV	9	1	1
												IHYGNLL	13	1	1
												EFLSAYI	3	1	1
												LASAMV	7	1	1
												GPDVPPA	4	1	1
												GGDAAS	6	1	1
												IGGDWV	8	1	1
												VAPWLT	2	1	1
												DVPVDV	3	1	1
												HTVYTH	1	1	1
												LLDPHIL	9	1	1
												YPEDLFE	10	1	1
												YRIDGEL	6	1	1
												SLTGNQT	1	1	1
												TTQPPAA	1	1	1
												SYPGIGT	3	1	1

											LTVLELP	2	1	1
											TTQPPAA	3	1	1
											EAYRIGG	2	1	1
											ATVDAY	2	1	1
											sSLEGPV	1	1	1
											iSTSPSSS	2	1	1
											TFTQQQ	1	1	1
											NFFSFAE	2	1	1
											QVIPVEQ	3	1	1
											LTVLELP	1	1	1
											VASERTI	2	1	1
											YHVDEPI	1	1	1
											NIEATRE	1	1	1
											SYPGIGT	1	1	1
											VLVSVRI	1	1	1
Rv1459c,	15608597	POSSIBLI	870.2783	12.69	1	6	6	23	591	62.65324	11.04834	VGPASGI	3	1	1
											DVYSYL	5	1	1
											ETPAPYC	8	1	1
											DVYSYL	2	1	1
											TLLLWm	2	1	1
											DGLDPYI	3	1	1
Rv0210,1	15607351	hypothetic	3344.439	12.8	1	6	6	65	492	52.31436	9.480957	VADQVSI	7	1	1
											VVAVETI	14	1	1
											VVAVETI	16	1	1
											SVVEQLV	3	1	1
											VADQVSI	2	1	1
											WTGEVT	23	1	1
Rv0093c,	15607235	PROBABI	2007.11	29.08	1	10	10	48	282	29.61237	9.158691	AWFTQV	8	1	1
											RHcATID	6	1	1
											GVPAAm	5	1	1
											aWFTQV	1	1	1
											HcATIDG	4	1	1
											AWFTQV	4	1	1
											GVPAAM	3	1	1
											RHcATID	3	1	1
											VDEHLGI	2	1	1

											LAESRPV	5	1	1
											HcATIDG	2	1	1
											GVPAAM	1	1	1
											RLAESRP	3	1	1
											LDGERAI	1	1	1
Rv0713,1	15608452	CONSERV	1785.744	29.93	1	8	8	55	147	16.58383	11.06299	VGYEIAL	8	1	1
											DIPAVGG	19	1	1
											GGEAAFY	4	1	1
											RVGYEIA	8	1	1
											GVEIISR	2	1	1
											YRGGEA	4	1	1
											RGVEIISR	6	1	1
											GGEAAFY	4	1	1
Rv0226c,	15607367	PROBABI	7436.671	22.92	1	11	11	199	576	59.10175	9.92041	ADVLTTC	48	1	1
											AVQELLI	13	1	1
											AVQELLI	18	1	1
											LAAAHRI	80	1	1
											VAPVHYI	6	1	1
											FSWSGSA	16	1	1
											DDELALY	4	1	1
											TVAVLPA	3	1	1
											RFSWSGS	6	1	1
											FSWSGSA	1	1	1
											TLGRLAA	2	1	1
											TVAVLPA	1	1	1
											TVAVLPA	1	1	1
Rv1522c,	15608660	PROBABI	342.6972	7.85	1	5	5	10	1146	122.3529	8.265137	EQAPLPP	3	1	1
											EQAPLPP	1	1	1
											IETSALLI	1	1	1
											ADTQDKI	2	1	1
											KLIENLR	1	1	1
											AWNLPIT	2	1	1
Rv0987,1	15608127	PROBABI	752.0943	10.53	1	7	7	22	855	93.54572	9.83252	EVELmAV	1	1	1
											EVELMAY	1	1	1
											EVELmAV	1	1	1
											EDIVIQST	2	1	1

												WIDGNKI	2	1	1
												YSSVFAP	9	1	1
												GVmLSEI	2	1	1
												GVMLSEI	1	1	1
												EVELMA	1	1	1
												SDVYVSI	2	1	1
Rv0206c,	15607347	POSSIBLI	517.4488	12.39	1	7	7	15	944	100.8407	9.45166	STDAAGI	2	1	1
												LLGDDcV	1	1	1
												mQIGSST	2	1	1
												AmPLAAI	1	1	1
												SVQSPAS	3	1	1
												SSPASSPI	3	1	1
												MQIGSST	1	1	1
												IGLGEIHI	2	1	1
Rv0528,5	15607668	PROBABI	4440.046	38.56	1	16	16	113	529	57.0959	9.026855	rKHQIAIQ	1	1	1
												FPALNAP	17	1	1
												IDQGPAA	8	1	1
												VNNFQAI	12	1	1
												KHQIAIQ	5	1	1
												HQIAIQG	4	1	1
												AGNTVD	5	1	1
												VYLQGH	12	1	1
												IDPPAGS	8	1	1
												TSTVQW	16	1	1
												GDTGLD	12	1	1
												AGQQVR	1	1	1
												LQVNHPI	2	1	1
												RKHQIAI	1	1	1
												tSTVQWI	2	1	1
												VGGDRV	1	1	1
												gDTGLDS	1	1	1
												fPALNAP	1	1	1
												IDPPAGS	2	1	1
												FPALNAP	1	1	1
												lQVNHPL	1	1	1
Rv1069c,	15608209	hypotheti	3050.983	36.97	1	14	14	78	587	64.30089	6.976074	TYAGLN	6	1	1

											HAGEALI	4	1	1
											TYAGLNS	20	1	1
											DAGSPQV	5	1	1
											SGGPGSL	6	1	1
											WIPVVTF	2	1	1
											VVYLQHI	5	1	1
											LVVFGES	2	1	1
											sGGPGSL	1	1	1
											TDGALFS	2	1	1
											LVVFGES	1	1	1
											WIPVVTF	4	1	1
											GYDVLPC	5	1	1
											YVATVAI	3	1	1
											HAGEALI	1	1	1
											ARDLQRI	1	1	1
											DLQRPDA	5	1	1
											LLFREPD	2	1	1
											tYAGLNS	2	1	1
											lVVFGESI	1	1	1
Rv1522c, 15608660	PROBABI	5345.077	19.9	1	14	14	90	1146	122.3529	8.265137	AQLAAIV	21	1	1
											ALAVTLC	39	1	1
											TPGALAI	1	1	1
											LDEATTL	2	1	1
											YFVQSAI	2	1	1
											TPGALAI	1	1	1
											GAQIFLS	4	1	1
											VADSARI	6	1	1
											KGAQIFL	1	1	1
											EIAELPNI	1	1	1
											EIAELPNI	1	1	1
											YFVQSAI	1	1	1
											QLENASC	4	1	1
											LAQmQQ	1	1	1
											TVSTLDC	2	1	1
											VADSARI	1	1	1
											DADKPSr	2	1	1

Rv0284,1	15607425	POSSIBLE	1123.897	16.32	1	19	19	35	1330	145.08	6.480957	DFPQVVA	1	1	1
												ALQDLAI	3	1	1
												IHILFASQ	2	1	1
												LIATIGEC	2	1	1
												STYVDGI	2	1	1
												KLIATIGE	2	1	1
												APQLWLI	6	1	1
												AVVVQSY	1	1	1
												LFTAAAV	1	1	1
												DPLVFDA	3	1	1
												SSAGNMT	1	1	1
												LIVIYADI	1	1	1
												RDPLVFD	2	1	1
												FADTLRC	2	1	1
												VASPSVS	1	1	1
												IGLKVAS	1	1	1
												GVGFLVI	2	1	1
												WPLGEID	1	1	1
												WPLGEID	1	1	1
Rv0876c,	15608016	POSSIBLE	137.6809	5.84	1	4	4	6	548	57.90082	10.85791	SRRPPPM	1	1	1
												YLPPLGE	3	1	1
												MYLLVH	1	1	1
												RPPPmPS	1	1	1
Rv0908,2	15608048	PROBABI	779.89	4.02	1	2	2	11	797	84.92037	6.873535	DAGDTV	4	1	1
												DAGDTV	6	1	1
												LAIIGQAI	1	1	1
Rv0076c,	15607218	PROBABI	445.2042	50.39	1	8	8	13	129	14.63993	10.81396	LQEmLLC	3	1	1
												FALIVTQ	1	1	1
												LQEMLLC	1	1	1
												VGVEAPI	2	1	1
												RLQEmLI	1	1	1
												KLSHQPP	1	1	1
												RLSQHFA	2	1	1
												GQILGRR	1	1	1
												LSHQPPV	1	1	1
Rv1795,1	15608932	hypothetic	2888.506	14.51	1	11	11	72	503	53.40082	9.041504	ELGEAPL	5	1	1

												ATQSLTE	8	1	1
												RSQVIEH	6	1	1
												SQVIEHIS	15	1	1
												WALcLVI	13	1	1
												LRELGEA	9	1	1
												ATQSLTE	7	1	1
												GRWALcl	2	1	1
												wALcLVD	2	1	1
												FIADTERI	1	1	1
												FIADTER	3	1	1
												SQVIEHIS	1	1	1
Rv0412c,	15607553	POSSIBLI	1547.523	12.76	1	6	6	33	439	47.08688	9.715332	HAVDKPI	5	1	1
												TITNLAIT	3	1	1
												ADETLSL	7	1	1
												INSDISVC	15	1	1
												RHAVDK	2	1	1
												QANDRIN	1	1	1
Rv1431,1	15608569	hypothetic	2556.992	24.45	1	14	14	60	589	65.28037	8.104004	IREGEIVA	3	1	1
												YYAGNW	13	1	1
												TGLFEAC	9	1	1
												EGEIVAG	13	1	1
												TGLFEAC	2	1	1
												GDAEATı	1	1	1
												LYDGATA	1	1	1
												cNFADGD	2	1	1
												ALNGLLI	2	1	1
												GDAEATI	1	1	1
												VIILEGQI	2	1	1
												ALDDEAI	4	1	1
												ALDDEAI	2	1	1
												SSALVVN	1	1	1
												RcNFADC	3	1	1
												VIILEGQI	1	1	1
Rv0051,2	15607193	PROBABI	1579.578	16.61	1	7	7	42	560	61.20137	9.715332	AYYQLcY	7	1	1
												YmEYPVI	4	1	1
												YmEYPVI	6	1	1

												AAcLQST	1	1	1
												SSWIETD	1	1	1
												YDGQIAV	14	1	1
												VANWDN	8	1	1
												AYYQLcY	1	1	1
Rv1811,3	15608948	POSSIBLI	1447.819	31.2	1	8	8	45	234	24.79114	8.338379	DNAVEDI	8	1	1
												TGPAGDI	4	1	1
												LVDRDNA	21	1	1
												TGPAGDI	4	1	1
												TSSNDITI	1	1	1
												TGPAGDI	2	1	1
												AHIVQRT	1	1	1
												AETYVRA	1	1	1
												VIcRPKAI	2	1	1
												TGPAGDI	1	1	1
Rv0982,1	15608122	PROBABI	1979.261	7.74	1	6	6	51	504	54.37377	9.671387	AQLLIAS	19	1	1
												TTADQRV	3	1	1
												AVIRGEL	3	1	1
												GELFMSR	15	1	1
												GELFmSR	6	1	1
												AVIRGEL	2	1	1
												RTTADQR	1	1	1
												gELFMSR	1	1	1
												GELFMSR	1	1	1
Rv0019c,	15607161	hypothetic	4426.373	41.29	1	10	10	85	155	17.14133	10.79932	ITLSEQPV	20	1	1
												YLVVTEC	24	1	1
												ADDSTLV	23	1	1
												ITLSEQPV	1	1	1
												YLVVTEC	1	1	1
												HAARYLY	1	1	1
												ADDSTLV	2	1	1
												VPIGTPVI	9	1	1
												AKVTTAY	2	1	1
												VTTAVRY	2	1	1
Rv0103c,	15607245	PROBABI	4737.638	12.1	1	4	4	82	752	77.46085	7.29834	AYPAASV	9	1	1
												AYPAASV	5	1	1

											LVIEATA	35	1	1
											LVIEATA	29	1	1
											FVTRPGE	1	1	1
											NVAVLLI	3	1	1
Rv1030,3	15608170	potassium-	643.3318	7.19	1	6	6	16	709	74.58691	6.404785	SAVTGGT	4	1	1
											TAAGNIE	2	1	1
											MIALVEC	2	1	1
											mIALVEG	3	1	1
											QGQTFID	1	1	1
											ESGGDRS	2	1	1
											RTAAGNI	2	1	1
Rv1739c,	15608877	PROBABI	609.5505	10.89	1	6	6	18	560	59.32907	9.539551	RVAHPHI	2	1	1
											YDAPLcF	4	1	1
											VAHPHDS	2	1	1
											VAHPHDS	2	1	1
											VPGLVVY	4	1	1
											RGIVFAm	1	1	1
											RVPGLVV	1	1	1
											RGIVFAM	1	1	1
											vPGLVVY	1	1	1
Rv1746,9	15608884	ANCHORI	102.2993	3.78	1	2	2	3	476	50.63667	5.98584	VcMQQTC	1	1	1
											VcmQQTC	1	1	1
											REcREEIF	1	1	1
Rv0676c,	15607816	PROBABI	185.63	8.61	1	3	3	3	964	104.7172	6.036621	LQADTTF	1	1	1
											LAGNQGI	1	1	1
											VFEEGDS	1	1	1
Rv0450c,	15607591	PROBABI	458.1688	7.86	1	5	5	14	967	105.1666	7.122559	AYVTGPS	3	1	1
											DAPSFEA	3	1	1
											YYDGLV.	4	1	1
											SIVESTPA	2	1	1
											EGDSDSF	1	1	1
											DAPSFEA	1	1	1
Rv0235c,	15607376	PROBABI	29.88593	3.53	1	2	2	2	482	54.80171	9.92041	AQLYQYT	1	1	1
											HNPFPQS	1	1	1
Rv1522c,:	15608660	PROBABI	799.4531	5.06	1	5	5	29	1146	122.3529	8.265137	TVTVPAI	9	1	1
											TVTVPAI	10	1	1

												EPVDHLI	1	1	1
												ANWWPS	7	1	1
												DPSTPPTI	1	1	1
												ANWWPS	1	1	1
Rv1814,3	15608951	MEMBRA	81.75874	11.67	1	2	2	3	300	34.8601	9.671387	LIWATHC	2	1	1
												WFEFVFN	1	1	1
Rv0996,7	15608136	PROBABI	33.04	6.15	1	1	1	1	358	39.4949	4.805176	WGYLPPI	1	1	1
Rv0394c,:	15607535	POSSIBLI	1277.759	35.15	1	5	5	24	239	25.26322	5.922363	IDKNPEL	4	1	1
												EIcESVGC	13	1	1
												LLAQAA	1	1	1
												LLAQAA	4	1	1
												iDKNPEL	1	1	1
												SSPDQDE	1	1	1
Rv0845,6	15607985	POSSIBLI	15133.54	48	1	30	30	345	425	45.75468	9.202637	AELLAQT	35	1	1
												AELLAQT	29	1	1
												ARVDAA	24	1	1
												aLAGLQS	1	1	1
												cVLDVAI	2	1	1
												LGITDEK	2	1	1
												cVLDVAI	2	1	1
												QELIELD	13	1	1
												SVAGLSA	18	1	1
												VDAAGG	20	1	1
												sGIDPIVF	2	1	1
												IAEAIHDC	34	1	1
												RIAEAIHI	18	1	1
												SGIDPIVF	18	1	1
												QATFELH	6	1	1
												SGIKISTD	2	1	1
												RLGEGHI	16	1	1
												LGITDEK	2	1	1
												ALAGLQS	20	1	1
												QATFELH	1	1	1
												LRQATFE	3	1	1
												ISTDIDYI	6	1	1

										SVAGLSA	12	1	1
										SVAGLSA	7	1	1
										ELLSNVV	16	1	1
										QELIELD	1	1	1
										LGEGHIG	18	1	1
										aELLAQT	1	1	1
										IAEAIHD	3	1	1
										rIAEAIHD	1	1	1
										LGEGHIG	2	1	1
										RLGEGHI	1	1	1
										HSGATTA	1	1	1
										RIAEAIHI	3	1	1
										SVAGLSA	1	1	1
										IAEAIHD	1	1	1
										lGITDEKc	1	1	1
										ELLSNVV	1	1	1
										ALAGLQS	1	1	1
Rv1319c,	15608459	POSSIBLE	8436.585	42.06	1	24	24	230	535	58.06118	9.759277	LLASSDT	40	1	1
										GDLVVFI	22	1	1
										FAGDAAI	5	1	1
										gDLVVFD	1	1	1
										HAAVVFY	8	1	1
										LANEMPI	13	1	1
										LANEmPI	2	1	1
										FFAIVVN	16	1	1
										LcELAKS	2	1	1
										AHWSLG	39	1	1
										VEQGDLI	14	1	1
										LDRPEDA	17	1	1
										GFNAMV	1	1	1
										FFAIVVN	6	1	1
										FAGDAAI	9	1	1
										ERPQLGC	11	1	1
										lLASSDTI	3	1	1
										EVAAAAI	1	1	1
										FEYTVVC	6	1	1

												VEQGDLI	1	1	1
												QRFEYTV	2	1	1
												RVEQGDI	1	1	1
												VRDLFGI	6	1	1
												HVGREV.	1	1	1
												EVAAAAI	1	1	1
												GHEQPTI	1	1	1
												AHWSLG	1	1	1
Rv0931c,	15608071	TRANSMI	4076.949	19.88	1	5	5	97	664	69.50249	5.858887	LSPSGVA	42	1	1
												LSPSGVA	43	1	1
												LLAGSTT	6	1	1
												VVTLAA(	2	1	1
												TQTVLPI	3	1	1
												VVKLAA(	1	1	1
Rv0292,2	15607433	PROBABI	1678.92	58.31	1	16	16	56	331	35.91004	10.34521	YTVAAA(	4	1	1
												IPLQETA(	11	1	1
												TWVGLT	2	1	1
												EIGWEA(	9	1	1
												HTDSDY	5	1	1
												VSANAEI	8	1	1
												LADHLRI	1	1	1
												SARIPLQI	2	1	1
												TTVLVW	2	1	1
												SRPAQET	2	1	1
												RLADHLI	2	1	1
												GMRHTD	2	1	1
												GmRHTD	1	1	1
												TDWRPG	2	1	1
												IPLQETA(	1	1	1
												YLDRYGI	1	1	1
												YLDRYGI	1	1	1
Rv0103c,	15607245	PROBABI	8781.041	24.2	2	16	16	206	752	77.46085	7.29834	LREEGHT	27	1	1
												GTDVAL(	20	2	1
												LREEGHT	25	1	1
												TGTLTV(	4	1	1
												AALTIAD	12	1	1

												SIDTVVF	1	1	1
												DSAAAA	4	1	1
												GETVVFV	23	1	1
												AAADAV	9	1	1
												TGTLTVC	3	1	1
												LDGESRC	18	1	1
												TTPcHDA	14	1	1
												AAADAV	9	1	1
												AALTIAD	7	1	1
												EEGHTVA	1	1	1
												TILLTGD	13	1	1
												GAQLGIF	1	2	1
												EEGHTVA	2	1	1
												SIDTVVF	6	1	1
												tTPcHDA	2	1	1
												lREEGHT	1	1	1
												AAADAV	1	1	1
												SIDTVVF	1	1	1
												AAADAV	1	1	1
												tILLTGDN	1	1	1
Rv1348,1	15608488	PROBABI	10256.69	25.03	1	24	24	273	859	92.90826	7.415527	LPDGYDT	32	1	1
												VLRLPDC	21	1	1
												HLQVTLI	41	1	1
												SLAADEI	26	1	1
												ADQIVVI	30	1	1
												GTHEELI	28	1	1
												IVERGTH	5	1	1
												TGLLAAI	2	1	1
												LHTITRA	11	1	1
												HLQVTLI	3	1	1
												DRTVLVI	18	1	1
												VGFVLQI	11	1	1
												gTHEELL	2	1	1
												sLAADEL	1	1	1
												STLATLL	5	1	1
												VGGQDIF	3	1	1

											tVLVIAHI	1	1	1
											AILGDTP	3	1	1
											GTHEELI	2	1	1
											TVLVIAH	17	1	1
											FHDVERC	1	1	1
											LTRDRTV	2	1	1
											ADQIVVI	2	1	1
											GAIRVGC	1	1	1
											LPDGYDT	1	1	1
											vLRLPDG	1	1	1
											aDQIVVL	1	1	1
											QRLTIAR	1	1	1
											EAQIHDR	1	1	1
Rv0969,1	15608109	PROBABI	5195.527	22.73	1	17	17	149	770	80.05293	9.085449	TAVFVGC	2	1	1
											LAAAVES	44	1	1
											GLAIPAA	16	1	1
											TAVFVGC	9	1	1
											LVDEQHI	6	1	1
											LHAmGLC	7	1	1
											LHAMGL	19	1	1
											GAELGIL	3	1	1
											VTDVIAG	11	1	1
											TAAAIAK	1	1	1
											LHAmGLC	6	1	1
											DDAADV	1	1	1
											KLVDEQI	5	1	1
											RKLVDEC	1	1	1
											lAAAVES	3	1	1
											mRVTDVI	5	1	1
											MRVTDV	3	1	1
											VLAEVLF	1	1	1
											IDTVVFD	1	1	1
											RQPDQVI	3	1	1
											vTDVIAG	1	1	1
											RQPDQVI	1	1	1
Rv1320c,:	15608460	POSSIBLI	6200.911	32.28	1	13	16	190	567	61.92565	7.840332	LASEMPI	32	1	1

												VEQGDLS	25	1	1
												FQGDASI	7	1	1
												WSLGETV	17	1	1
												LcELAKS	2	1	1
												FFTVIVDI	20	1	1
												FEYTVIG	26	1	1
												LLASSQT	16	1	1
												LASEmPE	5	1	1
												wSLGETV	1	1	1
												LASEmPE	1	1	1
												LLASSQT	2	1	1
												EVAAAAI	1	2	2
												vEQGDLS	1	1	1
												LSHPEDA	16	1	1
												lSHPEDA	2	1	1
												HVGREVA	2	2	2
												ERPKLGC	2	1	1
												VRDLFGF	5	2	2
												LASEMPE	6	1	1
												WSLGETV	1	1	1
Rv1348,3	15608488	PROBABI	305.9357	9.9	1		5	5	11	859	92.90826	7.415527 LPDGYDT	2	1	1
												HLQVTLI	2	1	1
												GTHEELI	2	1	1
												VLRLPDC	1	1	1
												VGFVLQI	4	1	1
Rv1349,7	15608489	PROBABI	2332.353	34.54	1		14	14	53	579	60.91662	6.265137 VLIDGTD	10	1	1
												LPDGANT	7	1	1
												VVEDGSI	4	1	1
												DNVFAAI	5	1	1
												STILALIA	2	1	1
												AQQAVcS	3	1	1
												YLEPFTA	4	1	1
												IGSVLTA	4	1	1
												VLFVDDC	5	1	1
												VDELIAR	1	1	1
												IGSVLTA	3	1	1

												FSQFWR	2	1	1
												VLFVDD	1	1	1
												AAPVLL	1	1	1
												HADRVL	1	1	1
Rv0014c,	15607156	TRANSM	634.1614	21.25	1		11	11	23	626	66.46905	5.452637 TSLLSSA	3	1	1
												YELGEIL	2	1	1
												HEGLSAI	2	1	1
												YELGEIL	2	1	1
												YQTAAE	1	1	1
												EDPIPPS	4	1	1
												VMDFGI	1	1	1
												DIVHTEG	2	1	1
												DPSFYLR	2	1	1
												DIVHTEG	1	1	1
												DVAVKV	1	1	1
												ADLARD	1	1	1
												AIADSGN	1	1	1
Rv1743,4	15608881	PROBABI	2872.374	28.45	1		14	14	87	566	60.47443	6.062012 GGmGDV	8	1	1
												YVTcGDI	8	1	1
												QIGSALD	9	1	1
												LQEPHV	10	1	1
												GGMGDV	7	1	1
												LQEPHV	13	1	1
												FSESHAT	5	1	1
												LTQLGN	2	1	1
												LmSETLS	1	1	1
												gGMGDV	1	1	1
												QGPLAP	2	1	1
												LMSETLS	1	1	1
												LTQLGN	2	1	1
												LVGRGG	1	1	1
												RQGPLA	2	1	1
												GMAKNP	1	1	1
												YVTcGDI	1	1	1
												GGMGDV	5	1	1
												GGmGDV	4	1	1

												LPVPSTH	2	1	1
												LVGRGGI	2	1	1
Rv1639c,·	15608777	hypothetic	193.3166	20.45	1	4	4	6	489	51.75927	8.147949	DAAANP(	2	1	1
												LFGGNEI	2	1	1
												HGSYTGI	1	1	1
												SENPPPP(	1	1	1
Rv0593,1	15607733	POSSIBLI	6751.881	58.96	1	20	20	208	402	43.14769	6.430176	MTIYVQN	18	1	1
												AIDSTNQ	21	1	1
												LATFTAE	14	1	1
												VADVWV	18	1	1
												NWIATLT	9	1	1
												YTPNPNI	14	1	1
												MTIYVQn	14	1	1
												LGLTAPF	6	1	1
												VLTDVPF	19	1	1
												DPDTmIP	5	1	1
												YTPNPNI	14	1	1
												mTIYVQn	5	1	1
												ALEQAW	11	1	1
												DPDTMIP	7	1	1
												ASPYVV(	1	1	1
												VRVADV	2	1	1
												ALEQAW	3	1	1
												IGQTSLL(	2	1	1
												FSEVANF	2	1	1
												ALAATRI	7	1	1
												ALEQAW	8	1	1
												LATFTAE	1	1	1
												aIDSTNQI	1	1	1
												NSSAYPT	3	1	1
												VADVWV	1	1	1
												aLAATRP	1	1	1
												fSEVANR	1	1	1
Rv0450c,	15607591	PROBABI	3141.864	22.75	1	20	20	137	967	105.1666	7.122559	MVGDTE	2	1	1
												QADEMA	2	1	1
												mVGDTEI	4	1	1

												MHSLMA	15	1	1
												mHSLMA	2	1	1
												mHSLmAI	22	1	1
												LSEQIGV	2	1	1
												MHSLMA	7	1	1
												SIFDALD	2	1	1
												MKPEILM	2	1	1
												AMKSFLS	1	1	1
												mVGDTEI	2	1	1
												VQAITRP	2	1	1
												LmPQmV	7	1	1
												SFLSSDG	3	1	1
												mHSLmAI	7	1	1
												DHVADFI	6	1	1
												EmDRLM	2	1	1
												QADEmA	1	1	1
												VQAITRP	4	1	1
												LMPQMV	6	1	1
												TSYNDRI	1	1	1
												DYLPDFI	5	1	1
												MVGDTE	1	1	1
												LMPQmV	10	1	1
												mKPEILM	1	1	1
												LMPQMV	4	1	1
												VQAITRP	2	1	1
												HcYGIPIc	1	1	1
												EMDRLM	1	1	1
												LmPQmV	2	1	1
												EITEELRI	1	1	1
												EMDRLm	1	1	1
												mKPEILm	1	1	1
												NPADFLV	1	1	1
												LSEQIGV	1	1	1
												FIILHR	3	1	1
Rv0284,2	15607425	POSSIBLI	5759.189	14.59	1	19	19	180	1330	145.08	6.480957	RFNDLQA	2	1	1
												IIPAMLGI	14	1	1

												IIPAmLGI	13	1	1
												GLTmAAI	11	1	1
												GPDQLVI	13	1	1
												FNDLQAT	2	1	1
												ATGSAHI	5	1	1
												LLPTNLA	15	1	1
												FNDLQAT	3	1	1
												RPPAGMS	8	1	1
												ATGSAHI	12	1	1
												LHLVDEI	18	1	1
												RPPAGmS	6	1	1
												VAFTVLI	3	1	1
												RPADAVI	11	1	1
												SGKTTLL	2	1	1
												YPGMAA	11	1	1
												RFNDLQA	2	1	1
												DNTDQFI	3	1	1
												GLTMAA	15	1	1
												DNTDQFI	2	1	1
												RLHLVDI	3	1	1
												VAFTVLI	2	1	1
												ATGSAHI	2	1	1
												LLPTNLA	1	1	1
												YPGmAAI	1	1	1
Rv1307,7	15608447	PROBABI	1520.628	48.88	1	14	14	29	446	48.77604	5.57959	ADSTVNI	1	1	1
												IAEQLEA	8	1	1
												AGQVDET	3	1	1
												QQLADA	2	1	1
												NHVADQ	2	1	1
												GQAVEEA	4	1	1
												QALLELA	1	1	1
												VGAPTLI	1	1	1
												LAILLGD	1	1	1
												WSANSDI	1	1	1
												YLTVPAI	1	1	1
												RGEIVAQ	2	1	1

												ALTSLVD	1	1	1
												LLDREAV	1	1	1
Rv1183,1	15608323	PROBABI	648.2798	6.59	1	5	5	12	1002	106.3477	8.645996	ATYQAG	7	1	1
												QVADGV	1	1	1
												LASGANI	1	1	1
												sPQTVSA	1	1	1
												SPQTVSA	1	1	1
												VSQIPGV	1	1	1
Rv0284,2	15607425	POSSIBLI	2508.583	20.53	1	21	21	65	1330	145.08	6.480957	VPIGVTG	1	1	1
												VPIGVTG	1	1	1
												VNDTADI	10	1	1
												GASFTTL	12	1	1
												LDVPALV	4	1	1
												ASALWSI	5	1	1
												HLLIVVD	3	1	1
												DEELRVF	1	1	1
												VSPITVL	1	1	1
												IIPAMLGI	2	1	1
												SIGDVPT	3	1	1
												WDSNPTI	5	1	1
												AGVTVV	2	1	1
												WQTGGW	3	1	1
												AWIAQA	1	1	1
												HTVPLAT	4	1	1
												GASFTTL	1	1	1
												DPHDPDI	2	1	1
												TEEVDAI	1	1	1
												EQYSDPE	1	1	1
												SLLDTQR	1	1	1
												SIGDVPT	1	1	1
Rv0425c,	15607566	POSSIBLI	6558.966	16.18	1	22	22	200	1539	159.6747	8.147949	GLGPGPV	17	1	1
												GNEISGG	17	1	1
												AAELVSC	29	1	1
												ALAGGLI	2	1	1
												QRGNEIS	3	1	1
												GNEISGG	14	1	1

										RAAELVS	13	1	1
										GAQITQA	4	1	1
										VLAGADI	1	1	1
										KVLAGAI	2	1	1
										AEELRPG	22	1	1
										RGAQITQ	2	1	1
										QVEPTPG	2	1	1
										gNEISGG	1	1	1
										LLAQQIPI	24	1	1
										THEVVPA	5	1	1
										cMLYAGT	2	1	1
										TVAVLSS	5	1	1
										ALAGGLI	2	1	1
										TGADLVS	7	1	1
										QRGNEIS	2	1	1
										LLAQQIPI	3	1	1
										cmLYAGT	2	1	1
										VLHAIPA	15	1	1
										APPSPYPI	1	1	1
										vLHAIPA	1	1	1
										RcMLYAG	1	1	1
										gLGPGPV	1	1	1
Rv0236c, 15607377	PROBABI	11816.99	23.36	1	24	24	307	1400	146.1895	9.583496	FTAATI	31	1	1
										RLQGQPI	13	1	1
										VSAPANI	34	1	1
										LFAAGRI	40	1	1
										ILIETVNG	4	1	1
										QGISYVL	17	1	1
										HTYNRVI	6	1	1
										RLQGQPI	3	1	1
										VDGGPEV	21	1	1
										LQGQPPL	13	1	1
										VPDYPVI	24	1	1
										AAGLPVI	7	1	1
										LQGQPPL	19	1	1
										ALDSVQI	2	1	1

												YPAIEIYI	20	1	1
												FDEAGKI	7	1	1
												LAEFGAI	7	1	1
												NDLDPET	2	1	1
												ETDYGRV	4	1	1
												THHAATI	2	1	1
												DSIPLTPI	8	1	1
												VLVVPGA	14	1	1
												VLVVPGA	3	1	1
												ILIETVNC	1	1	1
												SARPILLI	3	1	1
												SIAGSPGI	2	1	1
Rv0236c,	15607377	PROBABI	11621.78	23.79	1	26	26	334	1400	146.1895	9.583496	FTAAATI	29	1	1
												LFAAGRI	37	1	1
												RLQGQPI	13	1	1
												QGISYVL	19	1	1
												RLQGQPI	9	1	1
												ILIETVNC	6	1	1
												VPDYPVI	21	1	1
												HTYNRVI	9	1	1
												VDGGPEV	21	1	1
												VSAPANI	32	1	1
												LQGQPPL	19	1	1
												AAGLPVI	12	1	1
												LQGQPPL	19	1	1
												vSAPANP	2	1	1
												YPAIEIYI	20	1	1
												FDEAGKI	9	1	1
												VLVVPGA	13	1	1
												DSIPLTPI	11	1	1
												ALDSVQI	1	1	1
												SARPILLI	5	1	1
												SIAGSPGI	3	1	1
												VDHHSSA	1	1	1
												NDLDPET	2	1	1
												ETDYGRV	4	1	1

												THHAATI	2	1	1
												LAEFGAF	9	1	1
												VLVVPGA	3	1	1
												lFAAGRPS	1	1	1
												VDHHSSA	1	1	1
												SIAGSPGI	1	1	1
Rv2163c,	15609300	Probable p	169.1863	4.57	1	5	5	9	679	72.49229	9.627441	TRQVVDY	1	1	1
												GASFVFR	2	1	1
												RPPGAQE	2	1	1
												QVVDVG	3	1	1
												GLRRPPG	1	1	1
Rv2772c,	15609909	PROBABI	3829.91	36.94	1	8	8	76	157	17.31525	10.4917	LIAEDGL	35	1	1
												DAADALI	19	1	1
												dAADALF	1	1	1
												TELEDDA	1	1	1
												IQRDAAD	4	1	1
												TELEDDA	8	1	1
												AGFAYQI	1	1	1
												AYDYAG	4	1	1
												lIAEDGLI	1	1	1
												LIAEDGL	2	1	1
Rv2843,2	15609980	PROBABI	142.6856	13.81	1	3	3	4	181	17.75643	9.598145	ALATEIA	1	1	1
												LVATTSC	2	1	1
												TSAGEAS	1	1	1
Rv3252c,	15610388	PROBABI	145.7411	8.65	1	4	4	5	416	46.98435	9.129395	FGETLWI	1	1	1
												LGVSPWI	1	1	1
												VSTPEDP	1	1	1
												RLGVSPV	2	1	1
Rv2536,3	15609673	PROBABI	651.6309	24.78	1	5	5	16	230	24.61083	5.033691	ADDSPTC	4	1	1
												AADTDVI	5	1	1
												ADDSPTC	4	1	1
												TTESDTP	1	1	1
												EAPTEVII	1	1	1
												AADTDVI	1	1	1
Rv3694c,	15610830	POSSIBLI	1790.816	21.82	1	7	7	55	330	35.44869	6.536621	HSLSGAE	11	1	1
												SASSDQL	2	1	1

											SASSDQL	6	1	1
											SAVTGAI	10	1	1
											FWTVSFF	11	1	1
											sAVTGAI	1	1	1
											VSTHLSm	4	1	1
											VSTHLSM	8	1	1
											TFIRFWT	1	1	1
											SASSDQL	1	1	1
Rv2209,1	15609346	Probable c	620.0448	16.21	1	6	6	23	512	53.54479	11.16553	IGVTADT	11	1	1
											EGAPLDA	2	1	1
											STPPDHL	2	1	1
											RPAPPLN	2	1	1
											HVWDSR	5	1	1
											RPAPGKF	1	1	1
Rv2025c,(	15609162	POSSIBLE	4947.5	19.88	1	5	5	135	332	35.18466	6.712402	LHADAEI	60	1	1
											LLDGVDI	33	1	1
											RLLDGVI	13	1	1
											LLDGVDI	16	1	1
											IAHDAEH	4	1	1
											RLLDGVI	7	1	1
											lLDGVDP	1	1	1
											LLDGVDI	1	1	1
Rv3005c,	15610142	hypothetic	124.19	22.22	1	2	2	2	279	28.84186	5.186035	QVPAGSA	1	1	1
											YAGDFGS	1	1	1
Rv2874,1	15610011	POSSIBLE	636.345	6.62	1	3	3	19	695	74.12224	9.656738	cGYHSHL	2	1	1
											SLATPPT	15	1	1
											ScQLVAV	2	1	1
Rv3448,1	15610584	PROBABI	2090.866	22.91	1	10	10	62	467	46.70099	8.309082	YQLSALC	27	1	1

												cDDVAEA	9	1	1
												DGAVLVI	3	1	1
												RVTVHAG	6	1	1
												VTVHAGI	4	1	1
												RVTVHAG	3	1	1
												DGAVLVI	2	1	1
												GASPATA	1	1	1
												VTVHAGI	3	1	1
												dGAVLVI	2	1	1
												YQLSALC	2	1	1
Rv0638,1	57116765	translocase	174.1102	23.6	1	2	2	3	161	16.94589	9.656738	SVNPIAFY	1	1	1
												AAGADA	2	1	1
Rv3843c,	15610979	PROBABI	1743.096	21.64	1	8	8	62	342	37.36097	11.16553	WGLTDH	2	1	1
												GmLLAPI	11	1	1
												GmLLAPI	5	1	1
												GMLLAPI	9	1	1
												gmLLAPL	1	1	1
												WIAVRPC	9	1	1
												gMLLAPI	1	1	1
												cRGmLLA	2	1	1
												TPARPQV	12	1	1
												cRGmLLA	1	1	1
												TPPRLPPC	4	1	1
												TPARPQV	2	1	1
												cRGMLLA	1	1	1
												GmLLAPI	1	1	1
												GMLLAPI	1	1	1
Rv2287,1	15609424	Probable c	9438.576	17.53	1	11	11	182	542	57.45551	7.649902	AVLVmA	28	1	1
												AVLVMA	40	1	1
												mPEDVAI	7	1	1
												MPEDVAI	22	1	1
												NQNLIDD	21	1	1
												AVLVMA	28	1	1
												mPEDVAI	2	1	1
												VRLGVLC	1	1	1
												SAQAALI	6	1	1

												WARmPE	1	1	1
												LGVLQH	2	1	1
												AVLVmA	20	1	1
												AVLVMA	1	1	1
												QAVTTL	1	1	1
												QAVTTL	1	1	1
												lGVLQH	1	1	1
Rv3870,2	15611006	POSSIBL	3322.005	10.84	1	7	7	97	747	80.86182	8.001465	VTSSATS	23	1	1
												IGIGDQP	31	1	1
												TRVTSSA	5	1	1
												QWSRPA	31	1	1
												VPEINAD	2	1	1
												THGLIHD	3	1	1
												QWSRPA	2	1	1
Rv3887c,	15611023	PROBABI	483.5605	12.97	1	5	5	11	509	53.24371	8.543457	VAGESFE	3	1	1
												VAGESFE	2	1	1
												KGFDGV	2	1	1
												SLDELGV	2	1	1
												cAVNIcY	1	1	1
												GFDGVA	1	1	1
Rv3166c,	15610302	hypothetic	13247.62	32.92	1	14	14	377	319	33.37494	7.210449	ALHGAS	33	1	1
												AAEIGLA	21	1	1
												AAEIGLA	23	1	1
												EAIIAcY	21	1	1
												AAEIGLA	49	1	1
												EAIIAcY	29	1	1
												AAEIGLA	42	1	1
												ELSHVPG	40	1	1
												AAEIGLA	6	1	1
												ISGDRIES	55	1	1
												AAEIGLA	7	1	1
												FSPHVM	20	1	1
												IESPAPS	2	1	1
												FSPHVM	4	1	1
												FSPHVm	6	1	1
												eLSHVPG	1	1	1

												EAIIAcYV	2	1	1
												FSPHVmN	5	1	1
												SESLARA	2	1	1
												eAIIAcYV	1	1	1
												ISGDRIES	2	1	1
												iSGDRIES	3	1	1
												FSPHVmN	1	1	1
												IESPAPSA	1	1	1
												EAIIAcYV	1	1	1
Rv1997,3	15609134	PROBABI	1363.724	8.62	1	5	5	27	905	94.9729	5.60498	NIAYSGT	23	1	1
												QTGLSVN	1	1	1
												QTGLSVN	1	1	1
												SMVHTH	1	1	1
												QTGLSVN	1	1	1
Rv2942,1	15610079	CONSERV	4010.032	11.52	1	9	9	107	920	95.0624	8.851074	GEAGTTC	21	1	1
												HVGSVLI	25	1	1
												SATAMV	11	1	1
												ADTRHVC	2	1	1
												SATAmVV	8	1	1
												SATAmVV	2	1	1
												IAQAFPG	21	1	1
												QLPPSEG	8	1	1
												sATAmVV	1	1	1
												SATAMV	2	1	1
												hVGSVLD	1	1	1
												GEAGTTC	3	1	1
												SVLRQLP	2	1	1
Rv3743c,1	15610879	PROBABI	2225.613	15.45	1	10	10	66	660	68.60201	8.851074	VGAGGG	21	1	1
												IATLVEQ	5	1	1
												GLmGLAI	10	1	1
												GLMGLA	4	1	1
												TQLFIEK	1	1	1
												IGDIVLVI	20	1	1
												GLmGLAI	1	1	1
												ISADATV	1	1	1
												LARDSVV	1	1	1

												TQLFIEKY	1	1	1
												IATLVEQ	1	1	1
Rv1992c,(	15609129	PROBABI	3659.382	8.43	1	7	7	94	771	79.26038	5.808105	ALLSLVP	26	1	1
												IVHIVEAI	49	1	1
												LATDGIIF	9	1	1
												IVHIVEAI	3	1	1
												GLRALLS	2	1	1
												iVHIVEAI	1	1	1
												EGTETIV.	1	1	1
												aLLSLVPI	2	1	1
												LATDGIIF	1	1	1
Rv3764c,	15610900	POSSIBLI	7958.398	23.37	1	13	13	165	475	50.31343	7.459473	HLVAQLI	48	1	1
												TPLTLDL	21	1	1
												FLDAPGC	34	1	1
												RHLVAQI	3	1	1
												FLDAPGC	34	1	1
												tPLTLDLI	1	1	1
												IAGSRTPI	5	1	1
												FLDAPGC	1	1	1
												HETHNYI	1	1	1
												YRVLAAI	2	1	1
												AALTSTC	2	1	1
												AALTSTC	3	1	1
												FLDAPGC	2	1	1
												HETHNYI	3	1	1
												SQLERIA(	2	1	1
												SVLmYPE	1	1	1
												fLDAPGQ	2	1	1
Rv3823c,	15610959	PROBABI	1602.858	9.46	1	8	8	49	1089	115.9252	9.246582	EAGLQSV	3	1	1
												HPVAILPS	12	1	1
												DTRDVVI	2	1	1
												DVVMLQ	7	1	1
												DVVmLQ	11	1	1
												SYKELVI	2	1	1
												SYKELVI	1	1	1
												QmTAAFI	1	1	1

												VADIVEH	7	1	1
												DTRDVV	2	1	1
												QMTAAF	1	1	1
Rv3435c,	15610571	PROBABI	3752.087	27.11	1	7	7	94	284	30.60332	8.221191	LPGWNV	23	1	1
												VSVTFVI	25	1	1
												TWSSGSI	11	1	1
												SGPITVQ	24	1	1
												VSVTFVI	6	1	1
												sGPITVQI	2	1	1
												GAAHAPI	1	1	1
												SGPITVQ	1	1	1
												vSVTFVD	1	1	1
Rv2219,3	15609356	PROBABI	3569.129	23.2	1	5	5	74	250	26.84683	11.60498	KAEGQTC	2	1	1
												VTPGVA	23	1	1
												AEGQTG	4	1	1
												VIGRPGV	43	1	1
												vTPGVAA	1	1	1
												LPANITV	1	1	1
Rv3738c,	57117150	PPE FAMI	2364.924	19.68	1	6	6	50	315	31.41589	4.55127	LVSAVAI	38	1	1
												GAGVLG	4	1	1
												ESAAVRI	3	1	1
												ESAAVRI	1	1	1
												LVSAVAI	2	1	1
												TADVAPY	2	1	1
Rv2625c,	15609762	PROBABI	9548.269	32.82	1	16	16	237	393	41.4544	9.949707	SRALVTE	6	1	1
												ISTQQLF	25	1	1
												ALVTEGS	38	1	1
												VADAMT	20	1	1
												VADAmT	21	1	1
												DGSITGL	19	1	1
												HSAYPVA	50	1	1
												STTSVGD	21	1	1
												sTTSVGD	1	1	1
												vADAMT	1	1	1
												HSAYPVA	2	1	1
												RSTTSVG	7	1	1

												YVLGERI	2	1	1
												LIDVYR	16	1	1
												QLRDVAI	1	1	1
												DVAPSRF	1	1	1
												YVLGERI	2	1	1
												QLRDVAI	1	1	1
												hSAYPVA	2	1	1
												VADAmT	1	1	1
Rv3737,1	15610873	PROBABI	2525.934	9.26	1	11	11	92	529	54.7413	8.763184	TSGGIDD	16	1	1
												tSGGIDDI	2	1	1
												SRTSGGII	10	1	1
												LAELDRI	37	1	1
												RDPLPVA	7	1	1
												KVLDLTI	2	1	1
												DPLPVAC	6	1	1
												RSRTSGC	2	1	1
												VLDLTIR	2	1	1
												STDYSRL	1	1	1
												GRRDPLF	5	1	1
												lAELDRL	1	1	1
												STDYSRL	1	1	1
Rv3794,1	15610930	INTEGRA	3426.895	2.74	1	4	4	80	1094	115.6515	9.568848	DSVAAV	2	1	1
												ANQDTV	45	1	1
												ANQDTV	27	1	1
												VGAQPGI	4	1	1
												dSVAAVA	1	1	1
												aNQDTVV	1	1	1
Rv2743c,	15609880	POSSIBLI	4813.257	55.56	1	14	14	180	270	28.94345	11.3999	DLTAAAI	11	1	1
												GFFSLLG	14	1	1
												SYLVPTII	25	1	1
												DLTAAAI	7	1	1
												GFFSLLG	12	1	1
												YREELAC	21	1	1
												DLTAAAI	4	1	1
												GAmLPAI	3	1	1
												AVQcSAA	1	1	1

												GAmLPAI	7	1	1
												GAMLPA	6	1	1
												AVQcSAA	1	1	1
												LPPPGSA	10	1	1
												LPPPGSA	7	1	1
												RLPPPGS	5	1	1
												gFFSLLG	1	1	1
												GAmLPAI	7	1	1
												qYNEmV	19	1	1
												GAMLPA	2	1	1
												RLPPPGS	6	1	1
												WLRSEPI	6	1	1
												SEPLPVR	1	1	1
												yREELAG	1	1	1
												SEPLPVR	2	1	1
												sYLVPTIN	1	1	1
Rv3000,1	15610137	POSSIBLI	3672.829	63.47	1		19	19	100	219	23.38503	10.85791 VGLVAQ	27	1	1
												GASGSGI	1	1	1
												VWLDGV	4	1	1
												RVGLVA	18	1	1
												vSVVRDE	1	1	1
												VSVVRDI	14	1	1
												RRVGLV	2	1	1
												rVGLVAC	1	1	1
												mAVHGF	1	1	1
												VTELLAI	9	1	1
												VGRPDLI	5	1	1
												EAFLPHC	6	1	1
												LcLGQSA	2	1	1
												LIDPTSGI	1	1	1
												VGRPDLI	1	1	1
												lIDPTSGK	1	1	1
												AVHGFLI	1	1	1
												QVSAHFI	1	1	1
												TALIPAID	1	1	1
												EAFLPHC	1	1	1

												DEATVLI	1	1	1
												DEATVLI	1	1	1
Rv3695,4	15610831	POSSIBLI	1155.068	31.29	1	6	6	38	310	33.14675	8.865723	APQLDPA	10	1	1
												IAGDVVA	3	1	1
												LRPTLPP.	5	1	1
												LGPPPVm	1	1	1
												IAPPPPPC	12	1	1
												LRPTLPP.	4	1	1
												APQLDPA	1	1	1
												LGPPPVM	1	1	1
												APQLDPA	1	1	1
Rv3792,1	15610928	PROBABI	3832.03	22.24	1	19	19	139	643	69.47095	9.83252	FVVEDIG	15	1	1
												ATQIDSW	2	1	1
												TVVLTAI	1	1	1
												LAQDVYI	28	1	1
												TALFADI	14	1	1
												HGAHNSY	3	1	1
												tALFADPI	1	1	1
												YYPAIDA	4	1	1
												GDRRPPC	3	1	1
												LAQDVYI	25	1	1
												TALFADI	2	1	1
												RPPGSEK	1	1	1
												YTVDLR	25	1	1
												LPWQPPT	2	1	1
												HGAHNSY	1	1	1
												YTVDLRT	2	1	1
												RYTVDLI	5	1	1
												RYTVDLI	1	1	1
												lAQDVYP	1	1	1
												LPWQPPT	1	1	1
												TVVLTAI	1	1	1
												YYPAIDA	1	1	1
Rv3104c,:	15610241	POSSIBLI	1631.782	36.69	1	7	7	40	308	33.35996	6.087402	HDSLLGI	3	1	1
												HDSLLGI	8	1	1
												AVVDIPV	12	1	1

												IEVDTVT	11	1	1
												QYGFGDI	1	1	1
												VNEVLHG	2	1	1
												TLPGKQI	2	1	1
												SSEGEVF	1	1	1
Rv3793,1	15610929	INTEGRA	2259.748	10.51	1		8	8	58	1094	117.416	9.803223	NVPLVTA	12	1	1
												ANDDLVI	19	1	1
												VAAEFVG	13	1	1
												VAAEFVG	5	1	1
												LTFTAHA	5	1	1
												TVLLSTV	1	1	1
												SGYDFRF	2	1	1
												AVDRGLI	1	1	1
Rv1111c,-	57116830	hypothetic	3959.32	35.47	1		20	20	237	327	36.994	10.59424	LSSLmTG	1	1	1
												LSSLMTC	1	1	1
												KLSSLMT	2	1	1
												SYQPSEP	34	1	1
												kLSSLMT	2	1	1
												FDSYRSY	2	1	1
												RARPSEP	6	1	1
												KLSSLMT	1	1	1
												KLSSLmT	3	1	1
												YEPTYEP	30	1	1
												RDNYREI	5	1	1
												ARPSEPT	67	1	1
												HARPPSE	37	1	1
												SRHARPP	4	1	1
												LSSLmTG	1	1	1
												FDSYRSY	2	1	1
												SYQPSEP	24	1	1
												HARPPSE	4	1	1
												sYQPSEPS	1	1	1
												RPRPQNI	1	1	1
												DNYREEG	1	1	1
												aRPSEPTN	2	1	1
												YEPTYEP	2	1	1

												RRPRPQN	3	1	1
												YQPYARY	1	1	1
Rv2195,7	15609332	Probable F	2691.611	34.73	1	13	13	75	429	46.89304	6.150879	YQGETIY	26	1	1
												KAVLWT	2	1	1
												VcSHLGc	2	1	1
												ATGTEDC	8	1	1
												KGQESFN	2	1	1
												ESDGDGT	1	1	1
												LQEIAMC	2	1	1
												LQEIAmG	2	1	1
												AVLWTS	4	1	1
												NPVMLIR	8	1	1
												yQGETIY	1	1	1
												LQEIAMC	2	1	1
												MRPEDm	1	1	1
												LQEIAMC	2	1	1
												NPVmLIR	2	1	1
												LQEIAmG	1	1	1
												MRPEDM	2	1	1
												mRPEDmI	2	1	1
												IKPSDLG	2	1	1
												ILcPcHQS	3	1	1
Rv2869c,I	15610006	PROBABI	1931.277	26.98	1	9	9	33	404	42.80678	8.543457	VGDTPVS	1	1	1
												DGTAIVT	21	1	1
												AVIGETG	1	1	1
												VGDTPVS	2	1	1
												LEQcTGP	1	1	1
												AVIGETG	1	1	1
												WIPNGQC	2	1	1
												SHGSVPT	2	1	1
												sGDVVVI	1	1	1
												KSHGSVI	1	1	1
Rv2903c,I	15610040	PROBABI	2645.387	42.52	1	14	14	99	294	31.86001	5.947754	VIAVGGC	20	1	1
												FGSPQPG	19	1	1
												VWVMGI	6	1	1
												IMVDKLS	2	1	1

												ImVDKLS	3	1	1
												VWVmGI	8	1	1
												GPPSWN\	1	1	1
												WVQNAL	1	1	1
												RVIAVGC	2	1	1
												VWVMGI	5	1	1
												LIVWPPS	16	1	1
												VWVmGI	8	1	1
												SHNVAVI	1	1	1
												FGSPQPG	1	1	1
												AHcPLLcT	1	1	1
												LIVWPPS	4	1	1
												WVQNAL	1	1	1
Rv3239c,1	15610375	PROBABI	5559.788	15.94	1	14	14	111	1048	110.1304	9.437012	ASPDGPL	25	1	1
												LHVDGG	26	1	1
												TLTLLEA	9	1	1
												GADAALS	19	1	1
												tLTLLEA/	1	1	1
												HNPLSDY	15	1	1
												TLTLLEA	2	1	1
												RTLTLLE	4	1	1
												gADAALS	1	1	1
												LVDVIGc	1	1	1
												EAMPHIm	1	1	1
												EAMPHIM	1	1	1
												QDGSPK\	1	1	1
												cVSVDLL	2	1	1
												VPGIGDT	1	1	1
												LVDVIGc	1	1	1
												GADAALS	1	1	1
Rv2194,1	15609331	Probable	1081.212	8.57	1	2	2	28	280	29.12004	9.715332	NPDGSIA	27	1	1
												mPAmRGI	1	1	1
Rv3200c,1	15610336	POSSIBLE	7695.659	54.08	1	18	18	166	355	38.10869	8.147949	QSGADT\	29	1	1
												DDTAVL\	24	1	1
												IGAPEVD	4	1	1
												EVEQAE\	5	1	1

										AAAAGL	8	1	1
										LAGTQH/	41	1	1
										LAGTQH/	10	1	1
										VRNHTV	2	1	1
										TAVAAM	2	1	1
										TAVAAm	1	1	1
										DIVLGVV	21	1	1
										HLRDIVL	4	1	1
										LLGIATT	2	1	1
										DIVLGVV	6	1	1
										EAENQHI	1	1	1
										AAAAGL	1	1	1
										HLRDIVL	2	1	1
										NHTVVIC	1	1	1
										IGAPEVD	2	1	1
Rv3805c, 15610941	PROBABI	2618.111	19.14	1	10	10	70	627	68.66528	9.803223 VIDQIGL	14	1	1
										FYAQATC	12	1	1
										VTYSGIV	15	1	1
										FLSNVLH	15	1	1
										RFYAQA	7	1	1
										NLFPDW	1	1	1
										APITLHR	2	1	1
										WYPGIPC	1	1	1
										vTYSGIVI	1	1	1
										VTYSGIV	1	1	1
										YRIDRVP	1	1	1
Rv3365c, 15610501	hypothetic	7100.454	21.8	1	19	19	142	876	93.38388	6.932129 LADTDVI	17	1	1
										VEAAMA	5	1	1
										MAIMSDI	6	1	1
										VEAAmA	6	1	1
										SAmVTL/	2	1	1
										DIAEQVI	5	1	1
										mAIMSDI	13	1	1
										GQMTMC	6	1	1
										DRVTAY	11	1	1
										VTAYAPI	22	1	1

												mAImSDP	5	1	1
												VLADSIG	5	1	1
												SAmVTLA	1	1	1
												GQMTmQ	3	1	1
												DRVTAYA	2	1	1
												VTAYAPI	7	1	1
												VDSEQIR	3	1	1
												GADLAEI	7	1	1
												GQmTmQ	6	1	1
												SAMVTLA	2	1	1
												VDSEQIR	1	1	1
												NLQQQM	2	1	1
												AEmIPAIT	1	1	1
												KYELQTI	1	1	1
												VLADSIG	1	1	1
												TQVQGLS	1	1	1
												NLQQQm	1	1	1
Rv3645,8	15610781	PROBABI	2892.425	26.23	1		10	10	83	549	58.72248	6.315918	ELHDELI	23	1	1
												FEYTVIG	11	1	1
												FQGDAAI	2	1	1
												AIAGHIG	5	1	1
												VVVETVA	10	1	1
												WALSEVC	10	1	1
												GNYNAH	5	1	1
												GNYNAH	1	1	1
												GNYNAH	4	1	1
												GTELGGC	1	1	1
												YVGEDV	3	1	1
												LRDLFGF	7	1	1
												gNYNAHr	1	1	1
Rv3743c,:	15610879	PROBABI	7601.59	25	1		18	18	180	660	68.60201	8.851074	SELTLQT	17	1	1
												LTVVGDC	22	1	1
												LPTVGEF	5	1	1
												GTPELAC	17	1	1
												LAAAAEY	3	1	1
												LTVVGDC	20	1	1

											LGVQVGI	24	1	1
											QLQAGGI	2	1	1
											ATADRLC	10	1	1
											RFTDDEL	26	1	1
											IAFDKTG	2	1	1
											AGLLPDI	3	1	1
											IRLPTVGI	6	1	1
											LTGAKPV	2	1	1
											FTDDELL	15	1	1
											AGLLPDI	1	1	1
											VAAVRQ	1	1	1
											LAAAAEY	2	1	1
											aGLLPDD	1	1	1
											FTDDELL	1	1	1
Rv2601,7	57117003	spermidine	11727.65	50.48	1	20	20	316	523	54.64911	5.554199 SVLVLGC	22	1	1
											LYSTEFY	21	1	1
											YTESLVY	19	1	1
											LYLDGGI	16	1	1
											GAAVPPA	9	1	1
											DVNAGSI	14	1	1
											VHVVIDI	8	1	1
											HSAYQEI	17	1	1
											ALAPGGI	22	1	1
											LTDIAPTI	31	1	1
											sVLVLGC	1	1	1
											VHVVIDI	18	1	1
											TLDPSTL	2	1	1
											FLDQQVI	31	1	1
											ALAPGGI	8	1	1
											TTLRDVN	3	1	1
											QQLYADI	14	1	1
											DEYRYTI	6	1	1
											GAAVPPA	22	1	1
											ELLRQPC	4	1	1
											TLDPSTL	2	1	1
											SAGYAVT	22	1	1

												HSAYQEI	2	1	1
												DPDTPVI	1	1	1
												lTDIAPTP	1	1	1
Rv3428c,	15610564	POSSIBL	5433.881	58.05	1	26	26	194	410	45.46523	8.968262	HTAELVC	12	1	1
												SYAELAS	27	1	1
												HTAELVC	3	1	1
												TAGDVV	22	1	1
												mFEAVEC	14	1	1
												EFDSLAQ	12	1	1
												GREFDSL	1	1	1
												SAQGVLC	16	1	1
												TGVDKPI	5	1	1
												AFELTSW	2	1	1
												sYAELAS	1	1	1
												MFEAVEC	10	1	1
												ALEGAQI	11	1	1
												DNAIHHI	1	1	1
												STDFSHY	6	1	1
												VERPMTY	4	1	1
												AIEVGDP	14	1	1
												EFDSLAQ	1	1	1
												VERPmTY	10	1	1
												ALYSVPV	1	1	1
												sAQGVLC	1	1	1
												YLRALEC	1	1	1
												LVcDNLR	2	1	1
												SYAELAS	2	1	1
												TPTWcR	2	1	1
												HGcDRLE	2	1	1
												ALEGAQI	3	1	1
												LEAAcAR	1	1	1
												DSFWKG	2	1	1
												IAFHMR	1	1	1
												DKHGcDI	2	1	1
												tGVDKPD	1	1	1
												sTDFSHY	1	1	1

Rv2284,2	15609421	Probable e	5138.128	59.16	1	19	19	179	431	46.65226	9.319824	TANmQSY	23	1	1
												DYAHDG	13	1	1
												FQPGFEE	16	1	1
												NDSLVPV	12	1	1
												QVSKQPV	1	1	1
												IYRDYAH	8	1	1
												VQAAVPI	22	1	1
												TANMQSY	11	1	1
												GQAHPLr	9	1	1
												RPDLDLT	2	1	1
												TASAGLV	6	1	1
												GQAHPLN	2	1	1
												RPDLDLT	4	1	1
												LQDAmHT	8	1	1
												QPVVYAI	4	1	1
												NTWPDH	2	1	1
												LQDAMH	2	1	1
												LQDAMH	9	1	1
												NTWPDH	1	1	1
												AHISEYG	6	1	1
												ANHLDIV	6	1	1
												RTASAGI	1	1	1
												LQDAmHT	8	1	1
												nDSLVPV	1	1	1
												tANmQSY	1	1	1
												RPAGGGT	1	1	1
Rv2874,1	15610011	POSSIBLE	2130.011	30.79	1	15	15	51	695	74.12224	9.656738	WALDYQ	1	1	1
												LPQPSST	3	1	1
												FGEGDYI	3	1	1
												YWPAEY	13	1	1
												LASETLE	3	1	1
												HIKFGEG	3	1	1
												AALTPET	1	1	1
												DGKPATI	3	1	1
												NRYWPA	11	1	1
												GAANLGI	1	1	1

											AIPHVVG	2	1	1
											VVNYGG	1	1	1
											hIKFGEGI	3	1	1
											DSGLAVI	1	1	1
											QLLNDAI	1	1	1
											GRWALD	1	1	1
Rv3239c,	15610375	PROBABI	9897.346	21.56	1	26	26	263	1048	110.1304	9.437012	SIGLVLG	20	1	1
											GEVLGEI	20	1	1
											VQVLQD	3	1	1
											sIGLVLG	2	1	1
											LREAPPP	9	1	1
											AQFDEIA	2	1	1
											GADLVL	16	1	1
											VADKVV	12	1	1
											IVLVAGD	23	1	1
											GFAHLGV	31	1	1
											NPVQmPA	10	1	1
											ELGRGEV	14	1	1
											RQWEELI	6	1	1
											VQVLQD	8	1	1
											NPVQMP	2	1	1
											QWEELIT	10	1	1
											TNRNPV	16	1	1
											TNRNPV	3	1	1
											iVLVAGD	1	1	1
											SVHVVHT	4	1	1
											ARGADL	2	1	1
											GEVLGEI	2	1	1
											gEVLGEL	1	1	1
											gFAHLGV	1	1	1
											VVLHAA	2	1	1
											GADLVL	3	1	1
											sVHVVHY	1	1	1
											RILENVR	8	1	1
											ILENVRP	15	1	1
											VADRIVL	4	1	1

												tNRNPVQ	1	1	1
												VADKVV	2	1	1
												EAPPPAT	4	1	1
												VVLHAA	2	1	1
												vADKVVI	2	1	1
												vADKVVI	1	1	1
Rv2942,1	15610079	CONSERV	9204.968	29.89	1		24	24	189	920	95.0624	8.851074	NLSADFA	16	1	1
												LFLYSDG	29	1	1
												ISAISTQT	15	1	1
												SIIEQMSC	6	1	1
												ISAISTQT	13	1	1
												SIIEQmSC	2	1	1
												VIEPYDIV	2	1	1
												LADQLGC	4	1	1
												QGGAQV	4	1	1
												ESMFSSD	9	1	1
												DPAALIA	2	1	1
												KVESAAV	2	1	1
												KVIEPYD	1	1	1
												sIIEQMSC	1	1	1
												QAQQYLI	5	1	1
												QAQQYLI	6	1	1
												KVIEPYD	2	1	1
												DLRDPAA	27	1	1
												ESmFSSD	7	1	1
												AQQLEIA	4	1	1
												VIEPYDIV	1	1	1
												HVRESmI	1	1	1
												LVEVPGV	13	1	1
												HVRESMI	3	1	1
												QGGAQV	1	1	1
												LETTIQD	1	1	1
												SALAQVI	1	1	1
												DLRDPAA	1	1	1
												sALAQVF	1	1	1
												KDLADPS	3	1	1

												kVIEPYDI	1	1	1
												MVAQMF	1	1	1
												dLRDPAA	2	1	1
												DLADPSY	2	1	1
Rv2587c,!	15609724	protein exp	7823.234	35.78	1		13	13	168	573	60.22969	8.79248 TQISGGD	25	1	1
												QSTNQYI	26	1	1
												EALAQA(	28	1	1
												TAYLLAI	3	1	1
												QSTNQYI	34	1	1
												QSTNQYr	5	1	1
												LGIDLQG	12	1	1
												TPDGSAI	1	1	1
												qSTNQYn	3	1	1
												TAYLLAI	2	1	1
												GIGYVVI	18	1	1
												tQISGGDI	1	1	1
												LPLVTcST	2	1	1
												NLGQTAI	1	1	1
												QLANVLI	2	1	1
												VTLTART	2	1	1
												GPAANIW	1	1	1
												qSTNQYM	1	1	1
												VNGLGV!	1	1	1
Rv2339,1	15609476	PROBABI	5524.82	25.88	1		19	19	136	962	104.5453	7.166504 SLFDTLD	21	1	1
												IYIGGTA/	10	1	1
												MNPEML	5	1	1
												mNPEMLI	4	1	1
												MNPEML	4	1	1
												mNPEmLI	1	1	1
												FVISHESI	8	1	1
												GYAAAD	2	1	1
												DHLADID	21	1	1
												NPAGML'	2	1	1
												FVISHESI	12	1	1
												NPAGmL'	1	1	1
												MEALTP(	2	1	1

											ATTSELR	2	1	1
											TMMLTM.	4	1	1
											SYFYWEI	4	1	1
											STISGVQ.	1	1	1
											EIKATTSI	2	1	1
											VQAITRP	2	1	1
											EVLHVSC	5	1	1
											TMmLTM	4	1	1
											YYLRAGT	1	1	1
											sLFDTLD(	1	1	1
											TmMLTm	2	1	1
											IAKEVLH	1	1	1
											VQAITRP	6	1	1
											tmMLTMI	1	1	1
											VQAITRP	5	1	1
											VQAITRP	1	1	1
											AGTPVNI	1	1	1
Rv3793,2	15610929	INTEGRA	1657.753	18.74	1	21	21	60	1094	117.416	9.803223	FGAEANS	1	1	1
											APLLVVT	2	1	1
											LQWATD	2	1	1
											LTPYYPD	5	1	1
											APLSAIPS	7	1	1
											DRAPLLV	4	1	1
											LQWATD	2	1	1
											ATTVASY	2	1	1
											LVADDQ.	5	1	1
											LVADDQ.	2	1	1
											FGAEANS	1	1	1
											LPYNLDF	2	1	1
											SNLQALA	1	1	1
											NLRAPLS	4	1	1
											ILPDRFG.	1	1	1
											ARLPYNI	3	1	1
											AGVQVP.	2	1	1
											DWGALQ	2	1	1
											AGVQVP.	2	1	1

												TPVLGSV	4	1	1
												SGWYRL	2	1	1
												ATTVASY	1	1	1
												ATTVASY	1	1	1
												LPTNEQR	1	1	1
												iLPDRFGA	1	1	1
Rv3835,1	15610971	PROBABI	1954.762	40.31	1	16	16	69	449	47.01374	5.186035	IQQISEEC	8	1	1
												TFPGMEY	7	1	1
												mTDAYLA	2	1	1
												mLcGLQS	2	1	1
												FEVAESII	1	1	1
												RMLcGLC	1	1	1
												MTDAYL	2	1	1
												TFPGmEY	8	1	1
												FEVAESII	1	1	1
												VASGDcL	1	1	1
												FPDALPS	1	1	1
												RmLcGLQ	1	1	1
												FPDALPS	2	1	1
												MTDAYL	1	1	1
												TTTLTLIY	13	1	1
												FTISMLW	1	1	1
												INASFNR	1	1	1
												mTDAYLA	1	1	1
												GALLING	2	1	1
												FTISmLW	1	1	1
												IQQISEEC	8	1	1
												VASGDcL	1	1	1
												VASGDcL	1	1	1
												VASGDcL	1	1	1
												tFPGmEY	1	1	1
Rv1854c,	15608991	PROBABI	2053.73	39.09	1	13	13	52	463	49.58833	9.26123	VVIIGSGI	3	1	1
												TTHHLFC	10	1	1
												ILSAFEQA	19	1	1
												VWSAGV	4	1	1
												ADVDIKI	2	1	1

												AELAGAI	1	1	1
												SIDDALE	4	1	1
												VILLDAA	2	1	1
												VILLDAA	1	1	1
												GRILSAFI	2	1	1
												RIESAcKV	1	1	1
												mSPQQEP	1	1	1
												LGRDLAI	1	1	1
												VQVLPDI	1	1	1
Rv2921c,	15610058	PROBABI	5855.423	46.21	1	19	19	135	422	44.00458	5.36377	SGGYTAS	36	1	1
												GPEGADI	1	1	1
												AAAADQ	14	1	1
												IDTSGLP	11	1	1
												GPEGADI	2	1	1
												GIAAGAI	2	1	1
												TIADVHL	10	1	1
												SGGYTAS	1	1	1
												DVLINEL	6	1	1
												VVLGAA	29	1	1
												VQQELGV	1	1	1
												VFAEVVI	1	1	1
												GVVDRS	1	1	1
												RVVLGA	6	1	1
												RTIADVH	1	1	1
												AVLRDVI	7	1	1
												VGAAVV	1	1	1
												DVLINEL	1	1	1
												ISLSPRPE	1	1	1
												AVLRDVI	2	1	1
												AVLRDVI	1	1	1
Rv3447c,	15610583	PROBABI	165.9533	3.56	1	4	4	4	1236	131.8132	7.400879	VDYLGY	1	1	1
												LVVGQLI	1	1	1
												TIALGMN	1	1	1
												IRVGVGS	1	1	1
Rv0169,1	57116701	MCE-FAN	1584.158	36.12	1	8	8	34	454	47.75684	5.351074	HDIQQLA	14	1	1
												GVADLV	7	1	1

												DLWPAPY	1	1	1
												TINAQQA	3	1	1
												GGPGGAI	4	1	1
												VDTISEV	1	1	1
												TQLTmLS	1	1	1
												DLWPAPY	2	1	1
												ITPKDVII	1	1	1
Rv3894c,1	15611030	POSSIBLE	1493.705	15.54	1	19	19	48	1396	153.6044	6.442871	LADPGES	2	1	1
												TTALVAI	2	1	1
												TTALVAI	2	1	1
												GPEYGVI	2	1	1
												IGVPALA	2	1	1
												LADPGES	4	1	1
												QSLLQNV	3	1	1
												TLNRPGF	4	1	1
												VAQLYm	1	1	1
												VAQLYM	1	1	1
												LRESPGL	1	1	1
												qSLLQNV	1	1	1
												ESPGLmII	3	1	1
												GLSQEEL	1	1	1
												NSANWA	1	1	1
												NSANWA	1	1	1
												IISLSSR	1	1	1
												tLNRPGFC	1	1	1
												IADVAGV	1	1	1
												FSPQQAQ	2	1	1
												ESPGLMI	1	1	1
												qVGLHVF	1	1	1
												IADVAGV	1	1	1
												NSANWA	1	1	1
												eSPGLMII	1	1	1
												ALKPWEC	2	1	1
												LRESPGL	2	1	1
												DLHAPGY	2	1	1
Rv3479,1	57117112	POSSIBLE	3887.565	30.17	1	20	20	98	1021	109.1924	7.371582	VLEEELD	4	1	1

												SDADIAA	30	1	1
												AMLPAD	2	1	1
												AmLPAD	11	1	1
												DHVAAG	6	1	1
												SLLAPDV	3	1	1
												YALLNE	1	1	1
												LGAALV	5	1	1
												AWRPTA	14	1	1
												QGSLEN	2	1	1
												ADTNGP	1	1	1
												VVSLTK	1	1	1
												LFDLATT	2	1	1
												VLEEELD	2	1	1
												LDGAGW	6	1	1
												AAFQLA	2	1	1
												ANDWM	1	1	1
												ANDWmV	1	1	1
												TLTLSGY	1	1	1
												QLPSVLK	1	1	1
												LHHFGA	1	1	1
												ADDSRD	1	1	1
Rv3554,6	15610690	POSSIBL	3604.5	43.07	1	26	26	96	685	76.51734	6.946777	SSSPHAV	3	1	1
												NAAISTV	4	1	1
												IPVASVD	5	1	1
												AIFDLVP	14	1	1
												IPVASVD	15	1	1
												SDAPYA	2	1	1
												LEILHVL	3	1	1
												WLTSTL	3	1	1
												SSSPHAV	1	1	1
												AGDVLE	1	1	1
												FGTPLDP	5	1	1
												YADRLE	8	1	1
												TDLGGQ	5	1	1
												NYSIcAP	1	1	1
												ETLIEHG	4	1	1

											FEPGQHV	2	1	1
											DAFRFEP	4	1	1
											FTLIYGN	4	1	1
											RNEEAYI	2	1	1
											AELDRLE	1	1	1
											NEEAYLE	1	1	1
											VGAEWL	1	1	1
											RNYSIcAI	1	1	1
											FGTPLDP	2	1	1
											IHLELFY(	3	1	1
											IPVASVD	1	1	1
Rv2326c,(	15609463	POSSIBLE	1117.859	21.81	1	13	13	22	697	72.78937	7.122559	ALILDEPI	3	1	1
											LLSEVGL	3	1	1
											VAAALTY	2	1	1
											LLSEVGL	1	1	1
											DALLAVI	2	1	1
											TALVHIT	2	1	1
											VVLAGLI	1	1	1
											LALAAAI	1	1	1
											DTGSLSC	1	1	1
											IDQLSGG	1	1	1
											LGGTAVY	2	1	1
											AGQDALI	2	1	1
											RIDQLSG	1	1	1
Rv3479,1	57117112	POSSIBLE	3879.669	28.8	1	21	21	90	1021	109.1924	7.371582	TPSTTLY	27	1	1
											LGGTFPT	8	1	1
											VTGSDL(	3	1	1
											GLAAVTT	1	1	1
											LLTPSLLT	8	1	1
											EINLLAQ	4	1	1
											QAQTLTS	1	1	1
											QQITATII	11	1	1
											LATGPFP	2	1	1
											LAELAAT	7	1	1
											GLFTFTE	5	1	1
											VTGSDL(	1	1	1

												LSTcPPES	1	1	1
												TPSLLYG	1	1	1
												VLLFVVI	2	1	1
												FGQPAYI	1	1	1
												AAFQLAI	2	1	1
												SSASFPL	1	1	1
												DLWLDL	2	1	1
												VTGSDLC	1	1	1
												EATKQA	1	1	1
Rv3870,7	15611006	POSSIBLI	1731.705	37.22	1	24	24	48	747	80.86182	8.001465	VHLLLAT	4	1	1
												LPHTAAV	2	1	1
												vHLLLAT	1	1	1
												GPHAPDS	1	1	1
												VGAAGA	1	1	1
												QEGLSDI	3	1	1
												AVIGTPE	1	1	1
												MGEVLT	1	1	1
												mGEVLT	1	1	1
												IRVHEDG	1	1	1
												VHEDGT	3	1	1
												LPHTAAV	1	1	1
												VATDWH	2	1	1
												aGVTVIT	1	1	1
												AGVTVIT	2	1	1
												IRVHEDG	3	1	1
												mGEVLT	3	1	1
												VGMEDP	1	1	1
												MGEVLT	2	1	1
												GGSTFLC	1	1	1
												IDKLEPN	2	1	1
												WLPHVQ	2	1	1
												qEGLSDL	1	1	1
												LPNQSFR	1	1	1
												EGAEFGA	1	1	1
												MYTDTD	1	1	1
												QVTSVTI	1	1	1

												ESGVGFL	1	1	1
												mGEVLTC	1	1	1
												MGEVLTC	1	1	1
Rv3365c,	15610501	hypothetic	5773.429	36.3	1		32	32	135	876	93.38388	6.932129	ISDSGLGr	2	1	1
												VAAAIGS	22	1	1
												ISDSGLGI	2	1	1
												NSANLLV	10	1	1
												GPVTGEC	1	1	1
												SLVDQQI	7	1	1
												VAHTDLI	4	1	1
												RNSANLI	2	1	1
												RTPGSSG	3	1	1
												LLVNEmF	9	1	1
												LLVNEMI	2	1	1
												aLLLAGE	1	1	1
												TPGSSGIA	2	1	1
												LLVNEmF	5	1	1
												TPGSSGIA	2	1	1
												SLVDQQI	6	1	1
												LVPGAAV	2	1	1
												ISDSGLGI	3	1	1
												DHREPVF	3	1	1
												AGDEPIPI	4	1	1
												RTPGSSG	3	1	1
												AGGEVTI	1	1	1
												DHREPVF	4	1	1
												ALLLAGE	2	1	1
												LRAGGEV	1	1	1
												hMGLFVV	1	1	1
												TDYGLPV	1	1	1
												HmGLFVV	4	1	1
												SRSLVDQ	1	1	1
												NEEDPAF	4	1	1
												hAAAVRI	2	1	1
												HMGLFVV	3	1	1
												LDHLAAI	1	1	1

											ISDSGLG	2	1	1
											HAAAVR	2	1	1
											MLSEMV	1	1	1
											YSSPTTP	1	1	1
											LLVNEmI	1	1	1
											LLVNEMI	4	1	1
											ALLLAGI	1	1	1
											SLVDQQI	1	1	1
											TDYGLPV	1	1	1
											EPVPLST	1	1	1
Rv0151c,	57116694	PE FAMIL	223.3312	11.56	1	2	2	9	588	61.37294	4.525879	AIPVIGPI	5	1	1
											IPGLNVT	4	1	1
Rv2748c,	15609885	POSSIBLI	371.3143	9.97	1	6	6	8	883	94.34746	8.42627	NIAYAVA	1	1	1
											EYADDFI	2	1	1
											LADVLTA	1	1	1
											LAFATSS	2	1	1
											LmDLmE	1	1	1
											EYRPYPY	1	1	1
Rv3610c,	15610746	MEMBRA	1985.067	32.37	1	19	19	44	760	81.9349	5.846191	AAIAQAT	1	1	1
											AAEELVI	1	1	1
											TVGmTG	2	1	1
											TmGTQPI	1	1	1
											SEmIAQL	2	1	1
											EQTLNQI	3	1	1
											GAGLGG	2	1	1
											TVGMTG	2	1	1
											SEMIAQL	1	1	1
											SEMIAQL	2	1	1
											GAGLGG	2	1	1
											EQTLNQI	2	1	1
											QYGSTQI	2	1	1
											TMGTQPI	1	1	1
											TRSEmIA	3	1	1
											AAEELVI	1	1	1
											TPGELAI	1	1	1
											TRSEmIA	1	1	1

											TGGHAV.	2	1	1
											LTmFDDI	2	1	1
											LTMFDDI	2	1	1
											SmVTEFC	1	1	1
											YGSEHGI	1	1	1
											TmGTQPI	2	1	1
											VRDLFEC	1	1	1
											DFLQNPS	1	1	1
											TMGTQPI	1	1	1
											AGVILIA.	1	1	1
Rv3193c,!	15610329	hypothetic.	2954.183	38.71	1	32	32	73	992	107.4351	9.026855 IYFGPVIS	3	1	1
											NYSGDSI	5	1	1
											YTYTGSC	4	1	1
											VEAVAPV	2	1	1
											DDNSASY	1	1	1
											VAALQEI	2	1	1
											QAYGLTS	1	1	1
											nYSGDSP	1	1	1
											HTVYTHC	3	1	1
											GGLLYVI	4	1	1
											NFLFSNV	1	1	1
											QAYGLTS	6	1	1
											DDNSASY	1	1	1
											LANNAIT	2	1	1
											NGNLRDY	2	1	1
											SDIAPEL.	2	1	1
											LLDPTIVS	2	1	1
											NYSGDSI	2	1	1
											WGNLLT.	4	1	1
											GDFAAYC	1	1	1
											SITATRQ.	1	1	1
											VEAVAPV	1	1	1
											NFYYFPD	1	1	1
											VAMMYN	1	1	1
											SVFAAKI	1	1	1
											ELNPDRI	4	1	1

												wGNLLTI	1	1	1
												DYVVAA	2	1	1
												qAYGLTS	1	1	1
												IRWGNLI	2	1	1
												LIDNQRD	2	1	1
												LVWIVDC	1	1	1
												VAMMYN	1	1	1
												YPEDLFK	1	1	1
												ILFNRDP	2	1	1
												ELNPDRL	1	1	1
												gGLLYVE	1	1	1
Rv0083,1	15607225	PROBABI	915.4022	13.44	1	8	8	22	640	65.58546	10.06689	mQYTATS	3	1	1
												LPLWAcC	3	1	1
												MQYTAT	3	1	1
												LYTPVVC	2	1	1
												VFGDVLI	5	1	1
												tAVADAI	1	1	1
												TAVADA	1	1	1
												ITYRTAV	1	1	1
												LYTPVVC	1	1	1
												LYTPVVC	1	1	1
												YMAERIT	1	1	1
Rv0092,1	15607234	PROBABI	6699.621	19.71	1	20	20	127	761	78.80117	7.137207	TmLLTGI	18	1	1
												GTDVAIC	32	1	1
												tMLLTGD	1	1	1
												GHVVAm	3	1	1
												TMLLTGI	3	1	1
												AVDTVV	2	1	1
												DHLDVV	19	1	1
												VGIDEVL	2	1	1
												GTDVAIC	9	1	1
												ADLGmA	4	1	1
												VGIDEVL	1	1	1
												ADLGMA	1	1	1
												GHVVAM	1	1	1
												DAVAAL	16	1	1

												AVDTVV	1	1	1
												GLRTmLI	1	1	1
												VGIDEVL	2	1	1
												VDVIEQL	1	1	1
												DRGHVV	2	1	1
												GVSGTVS	1	1	1
												DAVAAL	2	1	1
												ADLGmA	2	1	1
												gHVVAm	1	1	1
												DAVTDFI	1	1	1
												AIPGRGV	1	1	1
Rv0176,5	15607317	PROBABI	1520.774	32.92	1	16	16	53	322	35.415	10.71143	GPKLVVI	1	1	1
												LDDLAV	1	1	1
												yRPQLSIC	3	1	1
												NQYWVT	5	1	1
												ESGPVRN	4	1	1
												ESGPVRN	2	1	1
												LDDLAV	1	1	1
												ESGPVRN	2	1	1
												GQWRLD	2	1	1
												NQYWVT	2	1	1
												LVVDVLS	2	1	1
												YRPQLSI	11	1	1
												GQWRLD	2	1	1
												NQYWVT	3	1	1
												NQYWVT	1	1	1
												LVVDVLS	2	1	1
												ESGPVRN	1	1	1
												YRPQLSI	1	1	1
												NQYWVT	1	1	1
												YIQSTVR	1	1	1
												SLATDRY	2	1	1
												ARSLATD	1	1	1
												SLATDRY	1	1	1
												NQYWVT	1	1	1
Rv0200,2	15607341	POSSIBLI	6884.885	50.66	1	11	11	154	229	24.01379	9.056152	DSREVVI	2	1	1

												RGVTAQ.	23	1	1
												EVVHLA7	28	1	1
												DSREVVI	5	1	1
												GVTAQA.	13	1	1
												GSGWLV	16	1	1
												EVVHLA7	23	1	1
												gSGWLVI	4	1	1
												DFNRDSI	3	1	1
												VSQSIPG	14	1	1
												gVTAQA/	1	1	1
												AAAMM	1	1	1
												AAAmMV	4	1	1
												eVVHLAT	4	1	1
												eVVHLAT	2	1	1
												RGSGWL	2	1	1
												AAAmmV	2	1	1
												vSQSIPGC	3	1	1
												VSQSIPG	1	1	1
												DFNRDSI	2	1	1
												ALRVTLT	1	1	1
Rv0206c,	15607347	POSSIBLI	1535.897	12.29	1	13	13	43	944	100.8407	9.45166	HVTQSGI	2	1	1
												HVTQSGI	3	1	1
												LAGLQP	2	1	1
												FQQDHPI	22	1	1
												ASQATGI	1	1	1
												SGHIVAII	2	1	1
												VVDELNI	1	1	1
												DRSGHIV	3	1	1
												fQQDHPD	1	1	1
												VVDELNI	1	1	1
												AIAPDLQ	2	1	1
												lDGGTVK	1	1	1
												KVVDELI	1	1	1
												LAGLQP	1	1	1
Rv0283,2	15607424	POSSIBLI	6007.533	57.99	1	19	19	144	538	55.9082	7.283691	MVQNTS	16	1	1
												GGADGV	27	1	1

												YGIDNEP	2	1	1
												sPIDLADI	4	1	1
												RSPIDLAI	2	1	1
												mVQNTST	7	1	1
												SPIDLADI	16	1	1
												YPSEPVS	2	1	1
												GNLIGIPC	7	1	1
												VALAAGT	20	1	1
												VGEQLHI	6	1	1
												STAALYV	5	1	1
												gGADGVC	3	1	1
												LGADEVA	4	1	1
												vGEQLHP	1	1	1
												RYPSEPV	2	1	1
												AAALGPC	4	1	1
												nNNSYGI	1	1	1
												NNNSYGI	2	1	1
												gNLIGIPG	2	1	1
												LIVGRPV	1	1	1
												aDALLAH	2	1	1
												AAALGPC	1	1	1
												STELDQF	1	1	1
												ADALLAI	4	1	1
												IIASGLFN	2	1	1
Rv0312,9	15607453	CONSERV	5567.151	18.87	1	12	12	148	620	64.10392	5.262207	TSIGLATA	14	1	1
												TSIGLATA	23	1	1
												ELAWSQT	12	1	1
												FEGDSYN	17	1	1
												TRTSIGLA	11	1	1
												rPVLTASC	4	1	1
												FEGDSYN	6	1	1
												TRTSIGLA	3	1	1
												RPVLTAS	20	1	1
												RPVLTAS	17	1	1
												ELAWSQT	3	1	1
												ELAWSQT	1	1	1

												RPVLTAS	2	1	1
												RPVLTAS	1	1	1
												RPVLTAS	1	1	1
												LNAVEPF	3	1	1
												fEGDSYN	1	1	1
												LNAVEPF	1	1	1
												LSFHTR	2	1	1
												LSFHTRR	3	1	1
												lSFHTR	1	1	1
												LSFHTRR	1	1	1
												eLAWSQT	1	1	1
Rv0338c,	15607479	PROBABI	3152.096	29.71	1		19	19	71	882	95.42499	7.312988	ELIGAAG	16	1	1
												VDEALAT	4	1	1
												INHERVD	6	1	1
												eLIGAAG	1	1	1
												GmLDFAT	1	1	1
												YQVMMF	1	1	1
												ELIGAAG	12	1	1
												LVPVTPV	1	1	1
												GNPWGQ	1	1	1
												RSGNEFL	1	1	1
												VMVTDG	2	1	1
												DASAGGI	2	1	1
												SFccGAG	1	1	1
												VMVTDG	3	1	1
												AVAELLA	1	1	1
												LPDGLGF	1	1	1
												VmVTDG	4	1	1
												VmVTDG	1	1	1
												MWmEEH	1	1	1
												MWMEEF	2	1	1
												sFccGAG	1	1	1
												LVIMDLF	1	1	1
												mWMEEH	1	1	1
												ELIGAAG	1	1	1
												qLGANYT	1	1	1

												vMVTDG	1	1	1
												LVImDLR	1	1	1
												mWmEEH	1	1	1
												lPDGLGP	1	1	1
Rv0362,1	15607503	POSSIBL	2814.258	51.52	1		17	17	85	460	49.28639	5.008301	LLGIIAE	14	1	1
												AVDLWL	1	1	1
												LLGIIAE	1	1	1
												TTAYVY	11	1	1
												QGGSAP	6	1	1
												VRELMG	2	1	1
												EALLTLL	11	1	1
												GLLSWP	2	1	1
												aLVLANP	3	1	1
												ALVLAN	8	1	1
												ELAAQTI	1	1	1
												GLLSWP	1	1	1
												ASPWLL	6	1	1
												HVIGIGT	1	1	1
												eALLTLL	3	1	1
												LAAEAL	1	1	1
												REALLTL	2	1	1
												SIRPAEN	2	1	1
												qGGSAPL	2	1	1
												EALLTLL	2	1	1
												ELAAQTI	1	1	1
												gLLSWPE	1	1	1
												GLLSWP	1	1	1
												ELMGDD	1	1	1
												ELMGDD	1	1	1
Rv0412c,	15607553	POSSIBL	9490.802	30.98	1		13	13	199	439	47.08688	9.715332	AGNPVG	1	1	1
												iNAELAV	2	1	1
												INAELAV	23	1	1
												YEQAITD	25	1	1
												ASSGLTD	18	1	1
												AGNPVG	12	1	1
												AGNPVG	17	1	1

												QQVLTA	16	1	1
												qQVLTA	2	1	1
												LYTTLSV	23	1	1
												LRYEQAI	41	1	1
												aGNPVGS	2	1	1
												lRYEQAI	2	1	1
												LRYEQAI	1	1	1
												INAELAV	1	1	1
												ANNRAG	1	1	1
												aSSGLTD	3	1	1
												yEQAITD	1	1	1
												LYQATS	3	1	1
												aGNPVGS	2	1	1
												ANNRAG	1	1	1
												LYQATS	2	1	1
Rv0431,1	15607572	PUTATIV	3134.614	23.17	1	6	6	56	164	16.92676	4.678223	VYNISGT	34	1	1
												ATADAV	3	1	1
												vYNISGT	2	1	1
												AEVRVY	7	1	1
												TLGAAV	6	1	1
												ATADAV	1	1	1
												VYNISGT	3	1	1
Rv0497,1	15607638	PROBABI	8057.695	63.55	1	18	18	136	310	33.07205	5.528809	HAAAAA	69	1	1
												RGDSDA	4	1	1
												VQVGEA	2	1	1
												RRGDSD	3	1	1
												QISVAEL	12	1	1
												GDSDAIT	2	1	1
												VQVGEA	9	1	1
												QISVAEL	1	1	1
												HAAAAA	1	1	1
												vQVGEA	2	1	1
												qISVAELI	1	1	1
												MTGPHP	1	1	1
												APAGPPS	2	1	1
												APAGPPS	4	1	1

												TVYWSQ	9	1	1
												tVYWSQF	1	1	1
												ESGPELS	4	1	1
												eSGPELSF	3	1	1
												QGVTGA	1	1	1
												qGVTGAF	1	1	1
												RESGPEL	2	1	1
												SPPQDRR	1	1	1
												gDSDAITY	1	1	1
Rv0676c,	15607816	PROBABI	1304.291	14.52	1	10	10	27	964	104.7172	6.036621	mLEQVNI	2	1	1
												MLEQVN	1	1	1
												MNPEVLr	3	1	1
												mLEQVNI	1	1	1
												NYLPADI	2	1	1
												mNPEVLr	2	1	1
												MNPEVLI	1	1	1
												FIISHEGD	2	1	1
												GLEQFLS	2	1	1
												FIISHEGD	2	1	1
												TNYNDRI	1	1	1
												mNPEVLr	2	1	1
												NSADFLV	1	1	1
												AIFAVEG	1	1	1
												NHIADFD	2	1	1
Rv0686,2	15607826	PROBABI	2426.192	51.7	1	13	13	47	265	28.55741	8.631348	SGVLALA	17	1	1
												QAPAAEI	7	1	1
												sGVLALA	2	1	1
												LVVLVNI	1	1	1
												VHLHISG	3	1	1
												AAERQAI	1	1	1
												ASGAVNA	1	1	1
												ASGAVNA	2	1	1
												VIASVTR	2	1	1
												qAPAAEP	2	1	1
												LAELESL	3	1	1
												AQIISEI	1	1	1

												qQVQAV	1	1	1
												mVDIRSN	1	1	1
												MVDIRSN	2	1	1
												RAQIISEI	1	1	1
Rv0816c,	15607956	PROBABI	5930.59	77.14	1	17	17	161	140	14.74884	9.26123	GVGDVcA	18	1	1
												TAELGLS	23	1	1
												tAELGLSG	3	1	1
												RGVGDV	12	1	1
												FSVLSLP	9	1	1
												RGVGDV	22	1	1
												RFSVLSL	15	1	1
												GVGDVcA	6	1	1
												RFSVLSL	18	1	1
												TAELGLS	3	1	1
												TSGVPKA	2	1	1
												EVGPETT	8	1	1
												FSVLSLP	13	1	1
												SALKPLL	1	1	1
												APGcAPcl	2	1	1
												APGcAPcl	1	1	1
												EVGPETT	2	1	1
												TAELGLS	2	1	1
												eVGPETT	1	1	1
Rv0901,9	15608041	POSSIBLI	280.1628	37.71	1	4	4	8	175	18.89778	9.656738	AGADGSG	1	1	1
												IPVVPYA	4	1	1
												VATESAA	2	1	1
												AFFTPWF	1	1	1
Rv0969,1	15608109	PROBABI	183.8737	7.92	1	5	5	6	770	80.05293	9.085449	AVAIEET	2	1	1
												VcVTGFN	1	1	1
												GPVRGSA	1	1	1
												AQHVPAI	1	1	1
												GSASWPG	1	1	1
Rv0982,5	15608122	PROBABI	5468.313	51.39	1	29	29	146	504	54.37377	9.671387	RRNDILF	2	1	1
												TNVELLn	4	1	1
												LSQLDAS	23	1	1
												IEDTDPG	8	1	1

											NDILFDV	2	1	1
											TNVELLN	7	1	1
											GSANVIS	3	1	1
											LSQLDAS	11	1	1
											RNDILFD	1	1	1
											GDAGEV	7	1	1
											LTEAFNL	15	1	1
											TDDLRPI	11	1	1
											GDAGEV	5	1	1
											LVTDAGI	4	1	1
											TNVELLm	5	1	1
											STDIENSI	2	1	1
											WSPPGGI	2	1	1
											LTEAFNL	6	1	1
											ALPGSGL	1	1	1
											ALPGSGL	1	1	1
											RmPIPQL	1	1	1
											IEDTDPG	4	1	1
											QARLVTI	2	1	1
											MALNLm	1	1	1
											RMPIPQL	2	1	1
											tDDLRPIP	1	1	1
											GSANVIS	1	1	1
											MPIPQLP	3	1	1
											LTEAAEF	1	1	1
											VARTDDI	1	1	1
											TPLTSLR	2	1	1
											LPKQEmV	2	1	1
											QVVLNH	1	1	1
											mPIPQLP	2	1	1
											GPGIPVQ	1	1	1
											LVTDAGI	1	1	1
Rv1115,9	15608255	POSSIBLI	718.5099	42.67	1	7	7	17	232	24.06704	5.998535 FNTGNEE	5	1	1
											VGGGGA	4	1	1
											GFSTSLL	2	1	1
											TmPGALS	2	1	1

												GFSTSLL	1	1	1
												GFQQGT	2	1	1
												LRTmPG	1	1	1
Rv1132,1	15608272	hypothetic	2556.089	26.04	1	16	16	81	576	63.97779	8.880371	GIVAIAS	4	1	1
												VVLLDA	8	1	1
												LVDAAT	4	1	1
												AmAGHD	8	1	1
												GIVAIAS	3	1	1
												AMAGH	6	1	1
												AQIPMY	5	1	1
												VcSTAV	2	1	1
												ISFLAAM	1	1	1
												cGFQPGE	3	1	1
												KISFLAA	2	1	1
												VcSTAV	3	1	1
												ALFTLA	5	1	1
												cGFQPGE	2	1	1
												AQIPmYL	4	1	1
												VADmVN	3	1	1
												QTQEYR	6	1	1
												AMAGH	1	1	1
												LVDAAT	2	1	1
												AmAGHD	1	1	1
												vVLLDA	1	1	1
												VADMV	5	1	1
												QTQEYR	2	1	1
Rv1318c,	15608458	POSSIBL	2462.09	22.18	1	11	11	55	541	59.29426	10.65283	LLASAQ	1	1	1
												TNLVVF	12	1	1
												LVNEMP	3	1	1
												AIADRL	2	1	1
												LVNEmP	9	1	1
												GFNAmV	4	1	1
												gFNAmV	2	1	1
												AIADRL	3	1	1
												ERFEYT	6	1	1
												GFNAMV	2	1	1

													HVGREV.	3	1	1
													FEYTVIG	3	1	1
													LLASAQ/	1	1	1
													HVGREV.	3	1	1
													LPcPEDK.	1	1	1
Rv1565c,	15608703	hypothetic	1439.073	17.42	1		16	16	51	729	80.1112	9.437012	TIALAGG	7	1	1
													AAGKELS	4	1	1
													AVEGNV.	5	1	1
													NPTLDFV	4	1	1
													TDTcRAV	2	1	1
													AVEGNV.	4	1	1
													AVEGNV.	2	1	1
													FPLLKPL.	1	1	1
													VLVDRNI	3	1	1
													TMTSELC	2	1	1
													QIAANTD	2	1	1
													MRPTVLI	1	1	1
													aLIDHVR	1	1	1
													TmTSELC	1	1	1
													SKVLVDI	3	1	1
													DSHHLTF	2	1	1
													mRPTVLE	1	1	1
													SKVLVDI	1	1	1
													DSHHLTF	4	1	1
													ALIDHVR	1	1	1
Rv1621c,	15608759	PROBABI	9120.066	43.83	1		31	31	229	527	54.76422	10.9458	TAADKA.	2	1	1
													AcAAAGI	14	1	1
													HNLVLLC	9	1	1
													AGATVLY	25	1	1
													ALGSPAA	6	1	1
													APYDLTA	8	1	1
													HNLVLLC	7	1	1
													VGVQFH.	55	1	1
													ALGSPAA	12	1	1
													STTLQAL	2	1	1
													AFALLGE	1	1	1

										ITVAGVI	13	1	1
										GGVGLSI	7	1	1
										QQVAAA	4	1	1
										TAADKA	3	1	1
										VVEVNSI	2	1	1
										DGRAPYI	6	1	1
										AcAAAGI	3	1	1
										ARAGAT	11	1	1
										TEQHVL(	7	1	1
										acAAAGF	1	1	1
										AFALLGI	1	1	1
										AGATVL	2	1	1
										GRDGRA	7	1	1
										GLDTVL(	5	1	1
										gGVGLSL	1	1	1
										APYDLTA	2	1	1
										DGRAPYI	2	1	1
										QQVAAA	2	1	1
										VGVQFH	1	1	1
										QLSWLP(	6	1	1
										VVEVNSI	1	1	1
										LERLSVR	1	1	1
Rv1635c,	15608773	Probable c	1233.483	6.83	1	3	3	28	556	59.99709	9.817871	IMSASTL	10	1	1
										ImSASTLI	15	1	1
										imSASTLI	1	1	1
										TAGDQD	1	1	1
										IMSASTL	1	1	1
Rv1736c,	15608874	PROBABI	858.6324	11.96	1	11	11	25	652	72.77936	9.158691	LGDPVA	6	1	1
										SGRFFIP(	2	1	1
										FFIPGEIS	6	1	1
										GAAFSW	4	1	1
										LGDPVA	1	1	1
										IEELLAR	1	1	1
										DGDVFYI	1	1	1
										ARLGDP	1	1	1
										GGRDGD	1	1	1

										FFIPGEIS.	1	1	1
										DRWSHD	1	1	1
Rv1743,1 15608881 PROBABI	1751.798	29.33	1	10	10	42	566	60.47443	6.062012	SYASDID	6	1	1
										TSAPPPPI	18	1	1
										SYASDID	2	1	1
										YHLLNFI	4	1	1
										AVAASY	4	1	1
										YHLLNFI	3	1	1
										LYASFYS	1	1	1
										VLNDGV.	1	1	1
										SSGPRTS.	2	1	1
										ATNASET	1	1	1
Rv1797,1 15608934 hypothetic	1036.764	42.12	1	11	11	23	406	44.1508	6.888184	LVSVIEV	3	1	1
										NLRDHDI	6	1	1
										DSLASTL	2	1	1
										VGSPALS	2	1	1
										DWSADP	2	1	1
										DHDELVI	2	1	1
										NVAAVA	2	1	1
										QFDIHLD	1	1	1
										LVQDLD	1	1	1
										TWLVLR	1	1	1
										RFGRDTV	1	1	1
Rv0062,2 57116687 POSSIBLI	2296.069	50.79	1	15	15	60	380	39.94905	8.104004	DcGSYAS	6	1	1
										APALILV.	2	1	1
										DPAAAV	7	1	1
										YTGAAQ.	3	1	1
										YTGAAQ.	1	1	1
										YAVDTL	8	1	1
										WLSAEAI	2	1	1
										WLSAEA	5	1	1
										YTGAAQ.	4	1	1
										NGAGPAI	3	1	1
										YAVDTL	4	1	1
										LNDVGV	1	1	1
										RPGESDC	1	1	1

												WLSAEAI	2	1	1
												NANPPNA	7	1	1
												lNDVGVC	1	1	1
												QERFDLV	2	1	1
												LNDVGV	1	1	1
Rv1625c,	57116894	MEMBRA	3874.876	38.6	1	20	20	94	443	47.33979	6.265137	mESTDSV	2	1	1
												FFYDVW	16	1	1
												SEALLAN	1	1	1
												MESTDSV	6	1	1
												VGLATGI	10	1	1
												SEALLAN	2	1	1
												AEAVmEA	2	1	1
												ASSTAPA	7	1	1
												LYSAFDE	3	1	1
												AEAVME	4	1	1
												RFFYDVV	9	1	1
												NIIADKY	1	1	1
												AEAVmEA	4	1	1
												GNPVPLF	4	1	1
												AEAVME	1	1	1
												FLDRLYS	2	1	1
												LKDDFVI	2	1	1
												AEAVmEA	1	1	1
												VAADPGI	1	1	1
												LKDDFVI	2	1	1
												aEAVMEA	1	1	1
												aSSTAPA	2	1	1
												TWYLIGF	6	1	1
												TWYLIGF	1	1	1
												VAADPGI	2	1	1
												RFFYDVV	1	1	1
												VGLATGI	1	1	1
Rv1819c,	15608956	PROBABI	1764.325	29.11	1	20	20	55	639	71.27251	8.572754	LHENQLF	11	1	1
												GGSLVIT	2	1	1
												SGAGKT	4	1	1
												ALPAVLT	6	1	1

												SELPDcIV	3	1	1
												NNYDAF.	2	1	1
												LDRGGSI	5	1	1
												DVVcYPN	2	1	1
												SLAELWI	2	1	1
												DVVcYPN	4	1	1
												LHGLVD.	2	1	1
												TPAGDRI	3	1	1
												SLAELWI	1	1	1
												LAPVEA.	1	1	1
												ALPAVLT	1	1	1
												LHENQLI	1	1	1
												LIDPLDV	1	1	1
												LIDPLDV	1	1	1
												TPAGDRI	1	1	1
												SELPDcIV	1	1	1
												ALPAVLT	1	1	1
Rv1845c,.	15608982	hypothetic.	2140.999	29.43	1	11	11	48	316	32.71624	11.78076	HDLVLE.	5	1	1
												SRVVVSI	3	1	1
												VVVSEGT	6	1	1
												ARHDLVI	20	1	1
												APSGAL.	4	1	1
												VLDVAQ	3	1	1
												SRVVVSI	1	1	1
												DLRVLD'	2	1	1
												ALVAcAS	1	1	1
												VVVSEGT	2	1	1
												ALVAcAS	1	1	1
Rv1877,1	15609014	PROBABI	3661.089	26.06	1	30	30	120	687	72.30787	6.741699	EVPLTDI	2	1	1
												EVPLTDI	9	1	1
												DWYEDL	5	1	1
												QVDSLA'	2	1	1
												AESPEDV	4	1	1
												QWLLDH	2	1	1
												LRDIATQ	5	1	1
												LVQTGY.	7	1	1

												DWYEDL	2	1	1
												HLHVPY(	8	1	1
												DGDILTL	10	1	1
												DIATQPG	3	1	1
												LVQTGY/	7	1	1
												QVDSLA\	7	1	1
												QVDSLA\	2	1	1
												RQPDHQI	1	1	1
												RQPDHQI	2	1	1
												HLHVPY(	6	1	1
												LFEAVRL	1	1	1
												QPDHQFI	7	1	1
												QPDHQFI	8	1	1
												LTDIGRH	1	1	1
												EVPLTDII	2	1	1
												RMLPNG\	1	1	1
												DGDILTL	2	1	1
												LTDIGRH	3	1	1
												AESPEDV	5	1	1
												MLPNGVI	1	1	1
												QWLLDH	1	1	1
												HLHVPY(	4	1	1
Rv1973,7	15609110	POSSIBLI	72.5697	21.25	1	2	2	2	160	16.81384	7.356934	AATDGT	1	1	1
												WLISQFE	1	1	1
Rv1997,9	15609134	PROBABI	2953.923	29.17	1	19	19	69	905	94.9729	5.60498	WSLLAG/	16	1	1
												GHVVAM	2	1	1
												AAAASA\	2	1	1
												GHVVAm	4	1	1
												RLPAVET	2	1	1
												LATTLPQ	7	1	1
												VLATGm(	2	1	1
												LVQALQ/	5	1	1
												ATEmLTS	1	1	1
												DGTRWQ	1	1	1
												QANIGV/	2	1	1
												DGTDHV	1	1	1

											ATEMLTS	1	1	1
											QANIGVA	1	1	1
											WSLLAG	3	1	1
											mLDLcGT	3	1	1
											ATEmLTS	1	1	1
											QYmATLI	1	1	1
											ATGTGYA	2	1	1
											LRLVQAI	1	1	1
											GVFDNLT	1	1	1
											MLDLcGT	2	1	1
											VLATGm	4	1	1
											VSPEQKL	1	1	1
											MLDLcGT	1	1	1
											gHVVAmT	1	1	1
											VLATGM	1	1	1
Rv2154c,	15609291	FtsW-like	832.7713	9.73	1	6	6	40	524	56.30131	11.15088	LPLPEPY	16	1	1
											ANPQPAC	4	1	1
											aNPQPAQ	1	1	1
											LLRLPLP	5	1	1
											LPLPEPY	8	1	1
											TAPGQPA	1	1	1
											QmGLPPF	4	1	1
											QMGLPPI	1	1	1
Rv2195,1	15609332	Probable R	259.1077	11.66	1	4	4	8	429	46.89304	6.150879	EPDEAAL	1	1	1
											ADDDAV	4	1	1
											IAYKEPR	2	1	1
											ADDDAV	1	1	1
Rv2200c,	15609337	PROBABI	821.4217	36.36	1	12	12	22	363	40.45389	7.840332	VETLGTS	1	1	1
											IDGKTNA	2	1	1
											AINQPPL	6	1	1
											DVMPNP	1	1	1
											DGTLTYI	2	1	1
											VNFKDG	4	1	1
											IEFQmAS	1	1	1
											YGEELVC	1	1	1
											AINQPPL	1	1	1

												AYLQQR	1	1	1
												TYLNFDK	1	1	1
												TYLNFDK	1	1	1
Rv2236c,1	15609373	cobalamin	3955.468	41.53	1	17	17	101	313	32.95573	10.9751	SWPVAAT	20	1	1
												LDDWAN	20	1	1
												AALESVA	10	1	1
												DPAQLGC	3	1	1
												FGWAAA	6	1	1
												AINTLDS	6	1	1
												AINTLDS	5	1	1
												TGRQISD	3	1	1
												RLLPSLcC	2	1	1
												LLPSLcGI	6	1	1
												QISDLLEI	4	1	1
												TGRQISD	1	1	1
												RLLPSLcC	1	1	1
												FGWAAA	2	1	1
												YLRFGW	2	1	1
												QISDLLEI	4	1	1
												AINTLDS	2	1	1
												QISDLLEI	3	1	1
												AINTLDS	1	1	1
Rv2262c,(	15609399	hypothetic	2165.192	30.56	1	10	10	66	360	37.23482	9.480957	LAAGEEI	18	1	1
												VGADLLY	3	1	1
												SAVLVGA	3	1	1
												LVPFGEY	15	1	1
												TRLVPFG	2	1	1
												lVPFGEY	1	1	1
												LAAGEEI	5	1	1
												LAELSQR	7	1	1
												HPDVLAI	2	1	1
												fSAGSPAT	1	1	1
												FSAGSPA	8	1	1
												LVPFGEY	1	1	1
Rv2306A	57116962	POSSIBLI	66.4177	12.18	1	2	2	3	197	21.21906	11.81006	WSVSASS	1	1	1
												cWVSIAP	2	1	1

Rv2339,1	15609476	PROBABI	2608.856	8.94	1	8	8	48	962	104.5453	7.166504	TYVTGPA	27	1	1
												DPLTAAC	1	1	1
												KIVANST	1	1	1
												AVAVAPI	8	1	1
												YYDHLVI	5	1	1
												YYDHLVI	2	1	1
												AVAVAPI	2	1	1
												IVANSTPI	1	1	1
												AVAVAPI	1	1	1
Rv2345,8	15609482	POSSIBLI	4060.194	14.85	1	13	13	102	660	69.98758	8.382324	TSELGNY	17	1	1
												LSNYVTI	14	1	1
												LWVVYV	15	1	1
												TSELGNY	5	1	1
												TSELGNY	4	1	1
												TTRTSEL	2	1	1
												TAVTAAY	3	1	1
												TAVTAAY	5	1	1
												EYAFLVP	3	1	1
												EYAFLVP	2	1	1
												IRLWVVY	3	1	1
												TAVTAAY	8	1	1
												TSELGNY	6	1	1
												LYADRR	1	1	1
												TSELGNY	10	1	1
												EYAFLVP	2	1	1
												EYAFLVP	2	1	1
Rv2443,1	15609580	PROBABI	242.4303	6.52	1	2	2	5	491	50.95558	8.279785	SLTNFSG	4	1	1
												TIDLSKA	1	1	1
Rv2643,1	15609780	PROBABI	1254.729	11.45	1	6	6	39	498	53.19164	8.51416	SQmAAGI	4	1	1
												VQALIAE	1	1	1
												LPDPAGC	3	1	1
												SQMAAG	2	1	1
												sQmAAGI	1	1	1
												SQMAAG	10	1	1
												NWKLPD	2	1	1
												SQmAAGI	10	1	1

												sQMAAGI	1	1	1
												MIRDDIA	3	1	1
												mIRDDIA	1	1	1
												nWKLPDI	1	1	1
Rv2673,2	57117165	6 KDA EA	1023.593	40	1	2	2	27	95	9.897765	4.513184	TISEAGQ	25	1	1
												WDATAT	1	1	1
												TISEAGQ	1	1	1
Rv2690c,	15609827	PROBABI	2374.085	20.4	1	17	17	81	657	70.75695	9.671387	ATRPDVI	11	1	1
												WWEQLL	3	1	1
												ATRPDVI	18	1	1
												IKTLQPH	2	1	1
												QWQDSD	2	1	1
												LLFMPGV	7	1	1
												LLFMPGV	6	1	1
												TLQPHAA	2	1	1
												TVVTVFI	4	1	1
												NHAVVLT	4	1	1
												GRLLFMI	1	1	1
												LLFmPGV	8	1	1
												GRLLFMI	2	1	1
												QWQDSD	1	1	1
												ELVRQW	1	1	1
												EITRPVLI	4	1	1
												EITRPVLI	2	1	1
												ATRPDVI	1	1	1
												NHAVVLT	1	1	1
												LHLPTLR	1	1	1
Rv2748c,	15609885	POSSIBLI	1626.183	5.44	1	4	4	33	883	94.34746	8.42627	GEQIAVT	27	1	1
												LAVmSSK	1	1	1
												SLLVSTG	1	1	1
												RPWQRG	4	1	1
Rv3252c,	15610388	PROBABI	1341.281	19.23	1	7	7	58	416	46.98435	9.129395	cAPVHSW	7	1	1
												VLEHYGC	23	1	1
												SMAGAPI	3	1	1
												SmAGAPI	8	1	1
												SmAGAPI	7	1	1

												VLEHYG	5	1	1
												VREKAL	3	1	1
												RYQTLR	1	1	1
												VNLHPR	1	1	1
Rv3764c,	15610900	POSSIBL	145.7162	5.89	1	2	2	3	475	50.31343	7.459473	TPLTLDL	2	1	1
												HLVAQL	1	1	1
Rv3779,1	15610915	PROBABI	397.6234	7.06	1	4	4	12	666	71.74907	9.246582	VLEAIQV	1	1	1
												GFAVPD	4	1	1
												FIFWAY	6	1	1
												DLDAmA	1	1	1
Rv3869,1	15611005	POSSIBL	2673.998	36.25	1	13	13	57	480	51.06069	7.620605	SAGDQS	11	1	1
												YTESmY	8	1	1
												ATNQLY	11	1	1
												ATQAHG	9	1	1
												YTESMY	2	1	1
												LGGTSL	2	1	1
												ATQAHG	2	1	1
												SLGLSSP	1	1	1
												FVALQS	1	1	1
												IVSRPQD	2	1	1
												LVLGNP	1	1	1
												HAIDLTD	3	1	1
												DAALLE	2	1	1
												LLVDGP	1	1	1
												GRHAIDL	1	1	1
Rv3877,1	15611013	PROBABI	246.8192	10.37	1	3	3	6	511	53.96515	6.800293	YRPLVE	2	1	1
												LDQSLD	2	1	1
												VTILTGR	2	1	1
Rv3894c,	15611030	POSSIBL	638.1271	7.74	1	11	11	18	1396	153.6044	6.442871	VGTGVQ	3	1	1
												EWQNAV	2	1	1
												SAPGFSF	1	1	1
												GDVDFL	1	1	1
												DEIQTAV	1	1	1
												GRGDVD	3	1	1
												DLDTNR	2	1	1
												VGTGVQ	2	1	1

												DFILEQR	1	1	1
												DLDTNRI	1	1	1
												YLRDLDT	1	1	1
Rv3921c,	15611057	putative in	123.93	10.38	1	2	2	3	366	40.8903	10.11084	TAPATNA	2	1	1
												TAPATNA	1	1	1
Rv0008c,	15607150	POSSIBLI	2247.172	48.97	1	6	6	39	145	15.66147	11.41455	Sequence	PSMs	Proteins	Protein Gr
												EVAAAQ	2	1	1
												GLLELGV	16	1	1
												EVAAAQ	17	1	1
												GLLELGV	2	1	1
												GLAYSAY	1	1	1
												STAAGLF	1	1	1
Rv0392c	15607533	PROBABI	1085.181	34.47	1	12	12	28	470	50.35336	8.470215	Sequence	PSMs	Proteins	Protein Gr
												ILGAFEA	5	1	1
												MIAEGSD	1	1	1
												VLLGEVN	2	1	1
												mIAEGSD	1	1	1
												RLTFVVV	1	1	1
												LTFVVVC	6	1	1
												IAALFAW	1	1	1
												VWAAGV	1	1	1
												VILLDAA	3	1	1
												TIDDALE	2	1	1
												VIVEPDL	1	1	1
												VILLDAA	1	1	1
												GHPNVFV	1	1	1
												ILGAFEA	2	1	1
Rv0758,1	15607898	POSSIBLI	5599.492	63.71	1	23	25	121	485	51.98448	7.078613	Sequence	PSMs	Proteins	Protein Gr
												ASDGYLT	19	1	1
												VGTEGDI	2	1	1
												ITLEVLD	8	1	1
												VGTEGDI	2	1	1
												MGLLVD	6	1	1
												VLLEEAC	4	1	1
												VGTEGDI	5	1	1
												mGLLVDI	10	1	1

												VDLLALA	10	1	1
												RITLEVLI	4	1	1
												QFITDASI	5	2	2
												SLRPLAE	6	1	1
												LSLALNG	3	1	1
												LSLALNG	1	1	1
												NLVANAI	6	1	1
												ASDGYLT	1	1	1
												RSLRPLA	3	1	1
												mGLLVDI	2	1	1
												DVGmLLS	5	1	1
												SLRPLAE	2	1	1
												IESEASRr	1	1	1
												sLRPLAEI	1	1	1
												TPLTTIR	3	1	1
												mRQFITD	4	2	2
												ITLEVLDG	1	1	1
												ASGGTGI	1	1	1
												GFAELYF	1	1	1
												DVGMLL	1	1	1
												LDAHRPI	1	1	1
												mGLLVDI	1	1	1
												VISPDGQ	1	1	1
												QFITDASI	1	1	1
Rv0774c	57116780	PROBABI	1769.626	49.5	1	6	6	42	303	30.63452	6.683105	Sequence	PSMs	Proteins	Protein Gr
												VAFLGW	4	1	1
												VAFLGW	15	1	1
												ASGEDAG	5	1	1
												VDcGNSI	1	1	1
												ASGEDAG	1	1	1
												TSHAAPA	4	1	1
												ASGEDAG	4	1	1
												ASGEDAG	1	1	1
												TSHAAPA	4	1	1
												aSGEDAG	2	1	1
												TAAIcAV	1	1	1

Rv1004c	15608144	PROBABI	358.4304	15.51	1	2	2	16	419	38.76112	4.932129	Sequence	PSMs	Proteins	Protein Gr
												LAAVLml	11	1	1
												VAGGTcS	3	1	1
												LAAVLM	2	1	1
Rv1032c	15608172	TWO COM	3074.361	42.24	1	18	18	71	509	54.55741	5.77002	Sequence	PSMs	Proteins	Protein Gr
												VESLGSL	4	1	1
												mTLLVDI	3	1	1
												LFVGVSL	1	1	1
												TPLAAIQ	17	1	1
												VAATAA	7	1	1
												VRPGDTI	5	1	1
												QDSSDLP	5	1	1
												GSVTAES	1	1	1
												AIEAQVV	4	1	1
												LLDNVDO	8	1	1
												RVAATA	2	1	1
												QFITDAS	4	1	1
												mRQFITD	3	1	1
												AIEAQVV	2	1	1
												TGPNAPc	1	1	1
												RmTLLVI	1	1	1
												TPLAAIQ	1	1	1
												LLDNVDO	2	1	1
Rv1352,:	15608492	hypothetic	807.5498	31.71	1	2	2	19	123	12.84139	5.808105	Sequence	PSMs	Proteins	Protein Gr
												TEGPSNP	11	1	1
												AETGEQI	8	1	1
Rv1418,:	15608556	PROBABI	524.8185	23.68	1	7	7	15	228	24.27214	8.396973	Sequence	PSMs	Proteins	Protein Gr
												APFGQVT	2	1	1
												SSGLPTS.	3	1	1
												DLLLQDO	4	1	1
												ARDLLLO	3	1	1
												ARDLLLO	1	1	1
												DLLLQDO	1	1	1
												VGDSYFT	1	1	1
Rv2261c	15609398	hypothetic	4349.961	53.57	1	4	4	94	140	14.93167	8.572754	Sequence	PSMs	Proteins	Protein Gr
												HAARLG.	4	1	1

												AVEAGIP	37	1	1
												LGAALLV	50	1	1
												HIAPLISY	2	1	1
												HIAPLISY	1	1	1
Rv2318,(	15609455	PROBABI	161.5461	11.82	1	4	4	4	440	46.99369	5.592285	Sequence	PSMs	Proteins	Protein Gr
												TNLVAYS	1	1	1
												LWDEPIA	1	1	1
												GVDVTPI	1	1	1
												GDDDTLI	1	1	1
Rv2914c	15610051	PROBABI	2470.296	15.21	1	6	6	58	585	61.76624	5.846191	Sequence	PSMs	Proteins	Protein Gr
												DVNPANY	9	1	1
												ILLADFG	29	1	1
												GLLHRDV	3	1	1
												ADQYAL	13	1	1
												LDGVLSF	3	1	1
												LDGVLSF	1	1	1
Rv3390,2	15610526	PROBABI	1132.147	27.12	1	6	6	33	236	25.04069	7.459473	Sequence	PSMs	Proteins	Protein Gr
												RDVDSIY	2	1	1
												DVDSIYS	2	1	1
												VVITGNP	16	1	1
												DVDSIYS	4	1	1
												LVEWDG	3	1	1
												vVITGNPY	1	1	1
												NSRDSLL	2	1	1
												DSLLTTH	3	1	1
Rv3492c	15610628	CONSERV	750.4831	40	1	6	6	22	160	17.99545	9.378418	Sequence	PSMs	Proteins	Protein Gr
												SLTAVYP	13	1	1
												VQTGGEA	1	1	1
												LAMQEIP	2	1	1
												sLTAVYP	1	1	1
												SLTAVYP	1	1	1
												LAmQEIP	2	1	1
												SANAQIII	1	1	1
												aLLPKLA	1	1	1
Rv3450c	15610586	PROBABI	6806.873	54.47	1	23	23	169	470	48.16933	6.785645	Sequence	PSMs	Proteins	Protein Gr
												ASGDEYY	22	1	1

												AVVDLA	15	1	1
												GPALDA	6	1	1
												LIAATNA	3	1	1
												VPHVVA	11	1	1
												ASVGLPC	12	1	1
												aSVGLPG	1	1	1
												LIAATNA	2	1	1
												AVTTLcV	7	1	1
												VAFLAGS	14	1	1
												LQGRVPI	1	1	1
												VDDVWH	8	1	1
												IGQVAAI	16	1	1
												RAVVDL	3	1	1
												FGDSQGS	10	1	1
												ESGALYV	7	1	1
												ITRASGD	1	1	1
												YLVTDTC	6	1	1
												VAFLAGS	10	1	1
												SLSGGGT	2	1	1
												VAPIVNT	7	1	1
												vAFLAGS	1	1	1
												SAYVAAI	1	1	1
												ESGALYV	1	1	1
												aVVDLAI	1	1	1
												ASVGLPC	1	1	1
Rv3604c	57117132	PROBABI	3246.252	44.84	1	16	16	99	397	43.03232	7.38623	Sequence	PSMs	Proteins	Protein Gr
												DPLTGGC	10	1	1
												ALDGESF	6	1	1
												DPLTGGC	2	1	1
												ARHASPA	2	1	1
												HASPADC	13	1	1
												DHDIGGS	3	1	1
												GGDGASI	5	1	1
												HSAEHRI	2	1	1
												HYEAPPF	16	1	1
												HASPADC	15	1	1

												TDEASIID	8	1	1
												RELASEL	1	1	1
												TSLEILFD	4	1	1
												ARHASPA	3	1	1
												DHDIGGS	5	1	1
												ALDGESP	1	1	1
												HSAEHRI	3	1	1
Rv0232,:	1.6E+07	PROBABL	24815	25424	39.7	229	5.9					1	1	513.7494	1025.484
												1	1	588.2906	1174.567
												1	1	690.9078	1379.801
												1	1	494.9238	1481.75
												1	1	777.3986	1552.783
Rv1698,5	15608836	hypothetic	6545.136	63.06	1	19	19	162	314	32.37181	5.478027	Sequence	PSMs	Proteins	Protein Gro
												LSAADNI	11	1	1
												IGTANAT	42	1	1
												EKLSAAD	4	1	1
												TPDAHDI	4	1	1
												DRIGTAN	27	1	1
												DTVLAAI	5	1	1
												SVVNSSII	3	1	1
												DGSANRI	7	1	1
												IVGQAGC	1	1	1
												FAAALAI	14	1	1
												LVDQGSC	5	1	1
												ETGFITY(	21	1	1
												FAAALAI	1	1	1
												eKLSAAD	1	1	1
												DLYTQID	4	1	1
												DLYTQID	7	1	1
												SVVNSSII	1	1	1
												GSGTLLA	2	1	1
												DTVLAAI	1	1	1
												GSGTLLA	1	1	1
Rv2945c,'	15610082	PROBABI	1241.392	65.67	1	11	11	24	233	24.12543	5.224121	Sequence	# PSMs	# Proteins	# Protein C
												LFDDWSI	2	1	1
												ASIDLGS(	2	1	1
												TTGKVDS	3	1	1

												VDSLLGI	1	1	1
												VQGDNIS	1	1	1
												VLDPAA(	6	1	1
												SARPATV	2	1	1
												GLTSVHV	2	1	1
												GVcTYNI	1	1	1
												ASIDLGS(	2	1	1
												QSLDATK	1	1	1
												vQGDNIS	1	1	1
Rv0286,:	57116716	PPE FAMI	1789.282	20.86	1	8	8	33	513	51.75526	5.592285	Sequence	# PSMs	# Proteins	# Protein C
												DYGDEFI	7	1	1
												SELRDYC	6	1	1
												DYGDEFI	1	1	1
												mPmVPG	5	1	1
												RSELRDY	3	1	1
												GAGTLGI	2	1	1
												APAATVI	3	1	1
												AVGLTAI	2	1	1
												mPMVPG	1	1	1
												GGIKAPA	3	1	1

Protein Group	Modificati	ΔCn	q-Value	PEP	IonScore	Exp Value	Charge	MH+ [Da]	ΔM [ppm]	RT [min]	Missed Cleavages
15607703		0	0	0.01907	89	1.83E-09	2	1494.7826	0.94266	23.31285	0
15607703	C5(Carban	0	0	0.06839	72.15	1.01E-07	2	1335.6513	0.346959	24.25277	0
15607703	M2(Oxidat	0	0	0.1275	67.55	1.75E-07	2	1931.9086	-0.88222	23.58878	0
15607703	M10(Oxid	0	0	0.06282	64.47	4.47E-07	2	1353.6578	-0.19492	16.35472	0
15607703		0	0	0.05142	59.86	2.22E-06	2	1667.8395	0.41601	29.37524	0
15607703	M15(Oxid	0	0	0.04419	59.07	1.36E-06	2	1915.9131	-1.18626	24.74454	0
15607245	M6(Oxidat	0	0	2.41E-10	105.55	2.78E-11	2	1526.675	-0.72703	26.40491	0
15607245	C15(Carba	0	0	6.07E-14	95.88	4.78E-10	2	2048.0037	-0.13457	38.57365	0
15607245	C8(Carban	0	0	1.43E-05	87.05	1.97E-09	2	1510.6819	0.505113	29.54958	0
15607245	C10(Carba	0	0	4.35E-08	86.32	2.92E-09	2	1779.8663	-0.05661	31.18058	1
15607245		0	0	0.000581	65.84	3.78E-07	2	865.45201	-0.77228	22.50983	0
15607245	M8(Oxidat	0	0	8.13E-06	63.02	5.99E-07	3	1795.864	1.510409	29.04548	1
15607245	N-Term(A	0	0	0.02377	62.95	7.6E-07	2	907.46251	-0.81031	22.13195	0
15607245	M9(Oxidat	0	0	5.19E-06	54.5	5.68E-06	2	2063.9949	-1.92813	36.30444	0
15607245		0	0	5.42E-06	42.25	0.00011	2	1397.7386	0.122834	25.35389	0
57116880	C14(Carba	0	0	2.7E-06	81.57	6.95E-09	2	2004.9241	-1.60377	36.73657	0
57116880		0	0	2.1E-08	78.12	3.7E-08	2	2245.1456	2.424837	47.85494	0
57116880		0	0	0.01251	71	2.22E-07	3	2678.3507	1.1947	39.53598	1
57116880	C1(Carban	0	0	0.000169	45.47	2.83E-05	2	1295.5448	1.120844	13.13315	0

57116880	M6(Oxidat	0	0	0.1283	44.53	3.52E-05	2	994.4395	-1.64472	13.57728	0
57116880	C5(Carban	0	0	0.00189	44.53	3.52E-05	3	1694.7281	-1.17647	13.51114	1
57116880		0	0	0.2392	39.26	0.000118	2	978.44866	2.488324	16.74608	0
57116880	M6(Oxidat	0	0	0.1947	25.74	0.002661	3	1692.7897	-0.86207	17.33233	1
15607431		0	0	0.008752	58.6	1.45E-06	2	1453.8072	-0.84247	35.28685	0
15607431	M4(Oxidat	0	0	0.001917	56.92	4.27E-06	2	1469.8045	0.799193	34.31469	0
15607431		0	0	0.01078	56.59	5.04E-06	2	844.48888	0.162069	21.31163	0
15607431		0	0	0.2531	43.38	4.59E-05	2	925.54637	-0.23556	25.39624	0
15607431		0	0	0.1097	40.2	9.53E-05	3	2360.3364	-0.31877	41.14003	1
15607431	M4(Oxidat	0	0	0.04108	30.41	0.000908	3	2376.3334	0.564683	39.74559	1
15607431	M12(Oxid	0	0	0.2876	24.5	0.0055	3	2295.2741	-0.05851	35.62276	1
15608503		0	0	0.006579	87.18	4.4E-09	3	2942.4924	1.183846	48.33012	0
15608503		0	0	0.03816	74.59	5.56E-08	2	1466.7317	0.597077	32.64097	0
15608503		0	0	0.1925	62.91	6.4E-07	2	1110.5418	-0.75247	20.83257	0
15608503		0	0	0.2386	58.3	3.11E-06	2	777.42485	-0.73192	15.91541	0
15608503		0	0	0.269	56.5	2.23E-06	3	1899.8824	-0.45946	30.23489	1
15608503		0	0	0.1428	47.13	2.81E-05	2	1244.6247	1.333806	16.18294	1
15608503	N-Term(A	0	0	0.271	34.66	0.000513	2	1152.5579	4.088842	23.18801	0
15608503		0	0	0.3186	27.55	0.001754	2	808.3579	-0.67369	13.74754	0
15608109		0	0	9.56E-05	112.17	1.21E-11	2	1498.8583	-0.35182	48.92715	0
15608109		0	0	0.01648	108.03	2.83E-11	2	1456.8127	0.527432	31.21349	0
15608109	M7(Oxidat	0	0	0.02595	106.37	2.54E-11	2	1777.8543	0.995214	25.44923	0
15608109		0	0	0.03949	91.33	1.18E-09	2	1761.8582	0.335212	28.89771	0
15608109		0	0	0.1032	71.74	1.44E-07	2	1157.5795	-0.21245	20.21351	0
15608109		0	0	0.05874	61.46	1.25E-06	3	2425.302	-1.08242	44.95697	1
15608109		0	0	0.1934	57.26	2.07E-06	2	2705.4776	2.016413	41.89345	0
15608109		0	0	0.1473	46.3	5.51E-05	3	1823.9698	4.742082	23.31755	1
15608109	N-Term(A	0	0	0.2119	22.54	0.010029	3	1865.9853	7.314302	22.59772	1
15608285		0	0	8.21E-05	100.91	1.26E-10	2	1639.8846	0.523075	30.19015	0
15608285	M1(Oxidat	0	0	0.00566	97.86	4.17E-10	2	1655.8801	0.861283	27.30072	0
15608285	M6(Oxidat	0	0	0.001339	87.02	1.98E-09	2	1383.6319	-0.23327	19.71933	0
15608285		0	0	0.001627	82.23	5.97E-09	2	1367.6375	0.1517	22.61759	0
15608285		0	0	0.02593	75.05	3.12E-08	3	2891.3296	1.111148	19.88149	1
15608285		0	0	0.1671	65	7.91E-07	2	1229.6736	-0.02417	33.49288	0
15608285		0	0	0.1092	63.11	4.87E-07	2	928.58269	0.051584	27.49772	0
15608285		0	0	0.01064	61.17	7.62E-07	3	2263.9923	-0.00335	21.43929	0

15608285		0	0	0.005337	59.59	2.14E-06	2	2800.4405	0.002138	41.01152	0
15608285		0	0	0.1188	57.6	2.78E-06	2	888.50365	-0.08174	22.6037	0
15608285	N-Term(A	0	0	0.1244	53.68	1.14E-05	2	1271.6834	-0.65196	38.63725	0
15608285		0	0	0.02854	42.65	0.000166	2	1385.7743	-0.31875	31.67197	1
15608285	N-Term(A	0	0	0.1766	41.15	7.66E-05	2	1409.6493	1.052241	24.35111	0
15608285		0	0	0.07438	38.15	0.000306	3	2956.5406	-0.32767	38.90236	1
15608773		0	0	0.05934	83.48	6.28E-09	2	1984.9529	-0.86291	29.50758	0
15608773	M10(Oxid	0	0	0.1288	74.75	3.35E-08	2	2000.9474	-1.06	26.59221	0
15608773		0	0	0.1314	70.98	1.12E-07	2	2228.0772	0.284881	28.62312	1
15608773		0	0	0.1242	64.05	7.87E-07	2	1434.7574	-0.98238	25.58994	1
15608773	C1(Carban	0	0	0.1694	47.44	4.15E-05	2	1231.6287	2.804748	30.86971	0
15608773		0	0	0.2619	41.41	0.000192	2	831.45812	1.220923	24.28398	0
15608773		0	0	0.3465	39.19	0.000175	2	1186.706	0.344581	24.64872	0
15608773		0	0	0.222	37.5	0.000365	2	1321.6703	1.153377	27.25954	0
15608773		0	0	0.1617	34.33	0.001015	3	1602.8417	-0.66279	34.1673	1
15608773		0	0	0.2904	30.69	0.001493	2	1483.8044	-0.87775	34.07757	1
15607453	M16(Oxid	0	0	4.13E-08	66.07	2.6E-07	3	2622.448	1.630499	33.59362	0
15607648		0	0	0.000174	108.95	1.27E-11	3	2594.2042	0.857855	34.28595	0
15607648		0	0	0.001804	104.22	6.24E-11	2	1861.8958	1.642547	24.43988	0
15607648		0	0	0.004252	95.29	3.7E-10	3	3142.5062	-0.49725	48.78731	0
15607648		0	0	0.001608	93.62	6.95E-10	2	1799.8945	-0.697	24.98661	0
15607648	M6(Oxidat	0	0	0.01621	88.26	2.46E-09	2	1815.8897	-0.51229	21.79304	0
15607648		0	0	0.01177	86.96	3.93E-09	3	2639.3155	2.058706	42.90119	0
15607648	N-Term(A	0	0	0.00505	71.33	1.51E-07	3	2681.3194	-0.47969	45.05091	0
15607648		0	0	0.02951	57.75	2.6E-06	2	1415.7095	-0.38217	17.30333	0
15607648	M6(Oxidat	0	0	0.03918	54.2	1.1E-05	3	1971.9887	-1.52316	20.31268	1
15607648		0	0	0.03596	52.36	1.39E-05	3	1955.9994	1.32541	23.29268	1
15607648	N-Term(A	0	0	0.1357	39.04	0.000143	3	3316.5707	-5.96685	28.12003	1
15607648	M6(Oxidat	0	0	0.217	38.94	0.000127	2	947.42845	-0.7525	21.58042	0
15607648	M2(Oxidat	0	0	0.2313	36.69	0.000311	2	1431.7039	-0.74825	18.09913	0
15607648		0	0	0.3513	34.64	0.000378	2	931.43419	-0.0651	27.08523	0
15607648	N-Term(A	0	0	0.008968	26.63	0.002168	3	2636.2102	-0.87135	33.26372	0
15607648	N-Term(A	0	0	0.3031	19.3	0.011721	2	973.4456	0.809433	29.5941	0
15607648	N-Term(A	0	0	0.3035	11.66	0.068072	2	989.44084	1.124066	24.75548	0
15608695	M4(Oxidat	0	0	0.1869	108.86	2.86E-11	2	1813.917	0.906043	31.74168	0

15608695		0	0	0.256	86.53	5.34E-09	2	2664.3631	0.571772	45.85352	0
15608695	M27(Oxid	0	0	0.2964	77.06	3.44E-08	4	3576.7262	-2.47693	48.71369	0
15608695	M1(Oxidat	0	0	0.032124	70.94	1.61E-07	3	2347.1577	-0.94042	25.4547	0
15608695		0	0	0.3907	66.61	2.18E-07	3	2501.4096	0.367707	41.0072	0
15608695		0	0	0.317	62.05	1.19E-06	3	2331.1648	-0.11722	28.89189	0
15608695		0	0	0.1813	59.53	1.45E-06	2	1148.6427	0.388714	25.76562	1
15608695		0	0	0.1979	45.64	2.72E-05	3	2286.9944	0.221959	37.03144	0
15607149		0	0	0.102925	80.07	1.62E-08	2	1571.8547	0.350259	25.82309	0
15607149		0	0	0.232	52.91	1.02E-05	3	1958.9833	0.03023	25.39747	1
15607149		0	0	0.3465	50.96	8E-06	2	1185.5495	-0.03815	20.17569	0
15607149		0	0	0.312	49.71	3.42E-05	3	1859.9569	-0.8	13.61221	1
15607149		0	0	0.2237	48.43	1.43E-05	3	1665.7765	-1.34945	14.288	1
15607149		0	0	0.3672	45.87	4.4E-05	2	1348.6619	-1.91929	24.89264	0
15607149		0	0	0.2882	36.49	0.000236	3	2310.0912	-0.84917	13.64848	1
15607149		0	0	0.3094	34.24	0.000735	4	2762.3383	-1.60507	13.91267	2
15607149		0	0	0.5341	25.64	0.004776	2	1407.7092	0.222165	13.36044	0
15608127		0	0	5.77E-16	124.22	8.7E-13	2	2327.2068	0.138968	45.3158	0
15608127		0	0	5.77E-16	104.15	6.73E-11	2	1829.9755	-0.08734	36.10377	0
15608127		0	0	5.77E-16	97.06	1.96E-10	2	1805.7986	-0.01646	36.88976	0
15608127	M5(Oxidat	0	0	5.77E-16	90.72	8.45E-10	2	1821.7947	0.630715	33.85423	0
15608127		0	0	5.77E-16	88.63	3.15E-09	2	1893.9478	-0.86581	33.23999	0
15608127	M18(Oxid	0	0	5.22E-08	75.98	7.57E-08	2	2343.2034	0.849452	42.65127	0
15608127	M2(Oxidat	0	0	5.77E-16	75.78	2.64E-08	2	1837.7887	0.137441	32.60723	0
15608127		0	0	5.77E-16	75.43	5.16E-08	2	1795.9915	0.10099	28.00924	1
15608127	N-Term(A	0	0	5.77E-16	75.42	5.74E-08	2	1872.0037	9.334067	34.19954	0
15608127		0	0	5.77E-16	72.51	9.26E-08	2	1164.6255	-0.38246	30.54485	0
15608127		0	0	5.77E-16	70.09	1.71E-07	2	2211.1866	-0.10866	38.60327	0
15608127	N-Term(A	0	0	1.77E-13	66.2	4.2E-07	2	1935.9516	-4.3496	38.09665	0
15608127		0	0	5.77E-16	54.62	9.84E-06	2	2050.0462	-2.13221	33.62959	1
15608127		0	0	5.77E-16	48.78	2.19E-05	3	1986.076	-0.41761	29.57991	1
15608127	N-Term(A	0	0	5.77E-16	47.6	1.73E-05	2	1847.8093	0.013746	42.56609	0
15608127		0	0	2.52E-06	43.63	9.1E-05	2	1560.7803	0.236892	20.58624	1
15608127		0	0	4.01E-08	39.45	0.00021	2	850.44109	-0.81565	21.20377	0
15608127		0	0	0.218	35.15	0.000916	3	1645.8645	-0.7201	26.84481	1
15608127		0	0	8.69E-16	33.5	0.000446	2	1051.6514	0.345634	28.56652	0
15608127		0	0	3.38E-12	28.11	0.001542	2	1137.685	-0.15574	19.22783	1

15608638	C12(Carba	0	0	0.05248	92.51	1.32E-09	2	1602.8051	-0.41427	27.71187		0
15608638	N-Term(A	0	0	0.02941	75.39	7.23E-08	2	2049.0408	-1.27924	34.49973		0
15608638	C6(Carban	0	0	0.03478	73.22	9.53E-08	2	2007.0312	-0.84691	29.19685		0
15608638		0	0	0.1771	55.26	7.6E-06	2	1131.6069	0.917123	22.5728		1
15608638	M2(Oxidat	0	0	0.126	53.69	4.28E-06	2	984.48131	-0.63637	20.41186		0
15608638		0	0	0.1416	52.04	7.5E-06	2	968.48717	0.152693	22.80557		0
15608638		0	0	0.2722	44.7	8.13E-05	3	1945.9922	0.426167	30.46861		1
15608638		0	0	0.2218	44.19	4.38E-05	2	946.46337	0.500991	20.26287		0
15608638	M2(Oxidat	0	0	0.3134	36.3	0.000445	3	1961.9879	0.836833	28.35243		1
15608638		0	0	0.2832	35.77	0.000305	2	841.47856	-0.73451	19.55968		0
15608638		0	0	0.294	35.73	0.000267	2	996.52117	-0.98898	13.0297		0
15607253		0	0	3.51E-13	87.14	3.19E-09	2	1295.7341	1.701806	45.40763		0
15607253		0	0	1.81E-09	85.65	3.68E-09	2	1059.6159	0.142528	49.11643		0
15607253		0	0	5.51E-09	71.23	7.52E-08	2	2247.0828	-0.08012	48.31967		0
15607253		0	0	1.09E-09	68.35	2.05E-07	2	1269.6427	-0.52259	18.72517		0
15607253		0	0	1.83E-06	64.8	5.13E-07	2	2735.4393	1.032844	32.49275		0
15607253		0	0	1.03E-08	63.79	8.15E-07	3	2137.1534	0.927947	34.4451		1
15607253	M20(Oxid	0	0	1.58E-10	58.83	2.16E-06	5	3420.6889	1.56833	45.42059		0
15607253	C9(Carban	0	0	3.51E-05	57.71	4.15E-06	2	1516.7927	-0.97255	27.75471		0
15607253		0	0	1.46E-05	56.96	3.73E-06	2	1107.5798	0.38662	22.05099		0
15607253		0	0	8.07E-10	54.21	6.07E-06	4	3404.6911	0.713558	46.2618		0
15607253	M2(Oxidat	0	0	3.32E-05	52.68	6.47E-06	2	1168.6019	-1.07997	21.87608		0
15607253	N-Term(A	0	0	2.62E-05	45.73	6.28E-05	2	1101.6285	1.960069	26.23085		0
15607253		0	0	0.004726	42.35	0.000148	2	1681.8621	-2.13047	26.1797		1
15607253		0	0	0.04156	40.63	0.000182	2	860.43761	0.287451	18.27145		0
15607253	C3(Carban	0	0	0.03432	39.52	0.000111	2	1061.4458	-1.05226	20.039		0
15607253		0	0	0.02434	38.84	0.000202	2	1360.6591	-1.00399	19.64368		1
15607253		0	0	0.003386	37.96	0.00024	2	1152.6081	-0.10539	23.69952		0
15607253		0	0	0.1327	34.7	0.000338	2	1146.542	-0.50719	17.89633		0
15608607		0	0	6.54E-09	94.47	7.15E-10	2	1891.0009	-1.26346	29.534		0
15608607		0	0	0.000327	91.44	1.51E-09	3	2545.2956	0.587796	49.53649		0
15608607		0	0	2.52E-05	89.46	2.15E-09	2	1145.6414	0.099264	26.39224		0
15608607	M22(Oxid	0	0	3.46E-09	78.96	2.8E-08	3	2561.2974	3.260509	26.8915		0
15608607		0	0	2.47E-08	77.77	2.84E-08	3	2047.1034	-0.5103	27.95141		1
15608607	C5(Carban	0	0	0.0191	59.51	1.68E-06	3	2643.297	-0.58454	34.51533		0
15608607		0	0	0.007128	59.15	1.34E-06	2	1536.8378	-0.19855	27.51356		0

15608607		0	0	0.02891	54.99	8.24E-06	2	845.44695	-0.7739	16.93503	0
15608607		0	0	0.000158	50.03	9.91E-06	2	1451.8569	-0.86935	25.07432	1
15608607		0	0	0.00924	44.11	4.08E-05	3	1340.7795	-0.22164	24.68926	2
15608607		0	0	0.04722	34.47	0.000356	2	869.50835	-0.91192	21.0811	0
15608607		0	0	0.01879	34.39	0.000363	2	922.58214	-1.28055	18.98703	1
15608379	M19(Oxid	0	0	0.01907	104.26	3.74E-11	4	3247.518	0.196686	42.57415	0
15608379		0	0	0.1478	71.21	7.55E-08	4	3231.5158	-2.05585	47.11509	0
15608379		0	0	0.1222	68.84	1.3E-07	2	1209.5162	-1.01728	20.90842	0
15608379		0	0	0.2019	68.67	2.17E-07	2	1094.591	-0.47627	32.38235	0
15608379	M5(Oxidat	0	0	0.3362	65.59	4.42E-07	2	1110.5856	-0.72701	29.1327	0
15608379		0	0	0.1437	61.67	1.57E-06	2	1583.8541	-0.0125	25.64875	0
15608379		0	0	0.1838	61.64	6.84E-07	2	1083.4885	-0.35107	20.25053	0
15608379	C1(Carban	0	0	0.2125	60.34	1.43E-06	2	1292.6414	-0.17815	29.06561	0
15608379	M3(Oxidat	0	0	0.2586	58.09	1.55E-06	2	1225.5108	-1.23745	16.71841	0
15608379		0	0	0.3251	53.75	8.43E-06	2	1240.6851	2.1721	37.88959	0
15608379		0	0	0.2665	52.72	7.22E-06	2	1177.57	-0.77907	15.55598	0
15608379	M22(Oxid	0	0	0.232	41.37	9.12E-05	4	3679.7716	1.573699	41.55376	1
15608379		0	0	0.3604	41.25	0.000109	2	928.48876	0.048245	22.49563	0
15608379		0	0	0.3291	36.98	0.000261	3	1323.7405	-1.16312	24.28651	1
15608379	C2(Carban	0	0	0.4346	32.63	0.00131	2	1448.7416	-0.78037	27.60532	1
15608379		0	0	0.3111	31.38	0.001019	2	835.46843	1.388548	26.82545	0
15608379		0	0	0.4241	29.63	0.001086	3	1344.8133	-0.32993	35.29193	1
15608921		0	0	5.79E-07	108.99	2.08E-11	2	1438.6966	0.263782	43.79205	0
15608921		0	0	0.000215	80.13	9.68E-09	2	1687.6861	-0.24207	20.56764	0
15608921		0	0	9.26E-14	77.65	3.69E-08	2	1791.9277	-0.74858	30.28371	0
15608921	M10(Oxid	0	0	0.000218	73.51	4.45E-08	2	1703.68	-0.83763	16.86951	0
15608921		0	0	6.27E-10	67.3	3.07E-07	2	955.55675	-0.43121	25.73214	0
15608921	M1(Oxidat	0	0	6.2E-12	65.67	7.18E-07	2	1877.9793	-2.01315	43.80722	0
15608921	M6(Oxidat	0	0	0.1653	65.37	3.34E-07	2	1400.6741	0.391643	35.44331	0
15608921	M6(Oxidat	0	0	5.2E-08	59.54	1.11E-06	2	1384.6789	0.161992	40.60149	0
15608921		0	0	8.17E-08	57.5	2.31E-06	2	1368.683	-0.51897	43.47664	0
15608921	M4(Oxidat	0	0	0.01973	56.47	2.25E-06	2	1416.6681	-0.24556	32.81721	0
15608921		0	0	0.06262	54.41	7.43E-06	2	1278.6346	1.60936	26.17332	0
15608921		0	0	0.00627	52.62	1.12E-05	2	1861.9892	0.548897	45.55264	0
15608921		0	0	2.09E-05	41.31	0.000141	2	919.4995	-0.15554	29.01694	0
15608921		0	0	0.03276	39.82	0.000104	2	1114.5435	0.541325	27.5584	0

15608921		0	0	0.1483	39.27	0.000201	2	1210.6471	0.389069	26.2581		0
15608921	M7(Oxidat	0	0	0.2982	33.81	0.000686	2	961.51372	0.138504	23.77703		0
15608921		0	0	0.000299	33.58	0.001009	2	855.43913	-0.23004	18.66718		0
15608921		0	0	0.2982	29.02	0.004135	2	945.5183	-0.39575	27.62212		0
15607648	M20(Oxid	0	0	0.000205	119.84	1.97E-12	3	2544.2534	-0.48156	38.84073		1
15607648		0	0	0.00686	113.82	7.68E-12	2	2257.1253	-2.80869	38.78537		0
15607648		0	0	0.002575	110.23	2.18E-11	3	2528.261	0.497633	40.37888		1
15607648	M13(Oxid	0	0	0.02796	107.93	2.74E-11	3	2560.2559	2.484997	34.90712		1
15607648		0	0	0.07311	97.06	1.96E-10	2	2920.3843	1.843471	38.16988		1
15607648		0	0	0.03409	93.3	1.05E-09	2	1836.9858	0.138309	36.56614		0
15607648	M18(Oxid	0	0	0.001504	91.67	1.33E-09	2	2273.1268	0.092508	37.11153		0
15607648		0	0	0.01189	88.02	1.57E-09	3	3408.5562	-0.52332	44.45226		0
15607648		0	0	0.01262	87.07	4.02E-09	2	1771.9245	1.069332	45.06882		0
15607648		0	0	0.05181	86.71	2.13E-09	2	1760.8073	1.401218	42.23598		0
15607648	M3(Oxidat	0	0	0.003094	79.67	1.08E-08	3	1629.7119	0.8872	19.26512		0
15607648	M2(Oxidat	0	0	0.02539	74.11	3.87E-08	2	1694.7635	0.506875	24.97954		0
15607648	M5(Oxidat	0	0	0.007323	73.68	4.28E-08	3	3424.5403	-3.7056	40.61521		0
15607648	M1(Oxidat	0	0	0.02951	71.92	6.41E-08	3	3472.5417	1.160494	31.66351		0
15607648	M14(Oxid	0	0	0.04077	71.5	1.38E-07	2	1810.8987	-0.95923	31.52195		0
15607648		0	0	0.04454	70.66	8.57E-08	2	2764.2801	0.815979	40.24657		0
15607648		0	0	0.005685	68.89	2.45E-07	2	1794.9047	-0.46833	35.21839		0
15607648	M11(Oxid	0	0	0.03148	68.04	2.2E-07	2	2289.1226	0.500145	32.95975		0
15607648	M1(Oxidat	0	0	0.07653	66.69	2.14E-07	4	3440.5487	0.251547	39.48866		0
15607648		0	0	0.09767	63.69	4.27E-07	2	1678.7629	-2.88088	26.83876		0
15607648	M3(Oxidat	0	0	0.09751	63.18	4.8E-07	2	1597.7209	0.163667	27.13616		0
15607648	M5(Oxidat	0	0	0.1029	63.01	1.43E-06	2	1159.6032	0.336937	28.2618		0
15607648	M3(Oxidat	0	0	0.05708	62.59	5.5E-07	2	1838.8531	0.311957	19.25085		1
15607648	M10(Oxid	0	0	0.02805	61.1	7.74E-07	2	1581.7264	0.423372	27.99231		0
15607648		0	0	0.09783	60.54	2.25E-06	2	1143.612	3.580563	31.88039		0
15607648		0	0	0.07933	58.92	1.6E-06	2	1777.8656	-0.04675	37.97361		0
15607648		0	0	0.04624	58.84	1.96E-06	2	1782.8671	-2.00142	40.67133		0
15607648		0	0	0.05728	57.98	4.06E-06	2	1558.8302	-1.92035	45.03989		0
15607648		0	0	0.02677	55.15	3.97E-06	2	1565.7316	0.532455	35.2606		0
15607648	M20(Oxid	0	0	0.1587	53.87	1.56E-05	3	2935.4941	1.225498	39.10471		1
15607648	M2(Oxidat	0	0	0.1327	53.67	4.29E-06	2	1710.7621	2.689596	20.44493		0
15607648	M6(Oxidat	0	0	0.1061	53.02	4.98E-06	2	1613.7154	-0.09089	21.58478		0

15607648		0	0	0.1103	49.8	3.61E-05	3	2919.494	-0.55134	42.14372	1
15607648	C2(Carban	0	0	0.03939	45.41	2.87E-05	3	1589.7354	0.09839	34.47466	0
15607648		0	0	0.1936	45.18	3.03E-05	2	1170.7203	-0.23819	21.29592	0
15607648	M12(Oxid	0	0	0.1666	39.94	0.000218	2	1574.8326	2.800825	41.50423	0
15607648		0	0	0.1405	37.21	0.000219	3	2278.2447	0.198747	35.76738	1
15607648	M1(Oxidat	0	0	0.171	36.29	0.000234	4	3456.5148	-8.09633	35.82337	0
15607648		0	0	0.242	34.63	0.000654	2	1384.7214	-0.44791	21.33981	0
15607648		0	0	0.1685	33.19	0.000528	2	768.38805	-0.79805	18.52718	0
15607648	M2(Oxidat	0	0	0.1901	28.79	0.00251	2	1590.8221	-0.63	37.81668	0
15607648	M14(Oxid	0	0	0.1506	28.37	0.004148	3	2951.4823	-1.04942	36.80113	1
15607648		0	0	0.2345	27.79	0.001659	2	764.45616	1.140708	23.77497	0
15607206		0	0	6.12E-16	116.55	2.43E-12	2	1862.8816	-0.23722	35.61487	0
15607206		0	0	3.77E-19	109.05	2.68E-11	2	2135.0774	0.271127	40.45891	0
15607206		0	0	6.9E-15	97.76	1.76E-10	2	1918.9183	0.025634	37.09056	0
15607206		0	0	4.85E-07	93.39	8.93E-10	2	1594.8067	-0.30918	26.63103	0
15607206		0	0	1.75E-14	88.84	2.16E-09	2	2636.2989	-0.13108	36.46547	0
15607206		0	0	1.32E-11	84.1	8.17E-09	2	1586.7857	0.429062	46.32529	0
15607206		0	0	1.26E-11	82.47	1.13E-08	2	1476.7696	0.154085	33.43573	0
15607206		0	0	3.97E-15	81.19	1.75E-08	2	2288.1604	0.273883	38.68246	0
15607206	M5(Oxidat	0	0	5.65E-05	80.92	1.09E-08	2	1097.5413	0.410977	19.09999	0
15607206		0	0	2.23E-11	77.97	5.11E-08	2	1499.8067	0.144634	49.77344	0
15607206		0	0	3.15E-11	76.83	2.7E-08	2	2902.5396	1.576923	41.807	0
15607206		0	0	4.18E-06	75.89	3.61E-08	2	1969.9277	-0.72662	31.18954	0
15607206		0	0	2.7E-10	74.97	4.3E-08	2	1081.5457	-0.22135	22.07482	0
15607206		0	0	1.49E-05	73.54	8.85E-08	2	1321.7116	0.35874	26.58271	0
15607206		0	0	1.15E-17	70.63	2.29E-07	2	2189.1072	-1.24067	22.003	0
15607206		0	0	3.36E-05	67.1	4E-07	2	1093.5418	-0.70016	27.24339	0
15607206		0	0	6.15E-14	64.59	5.91E-07	2	2113.0757	2.063292	33.86019	0
15607206		0	0	7.03E-07	63.38	6.43E-07	2	1157.6407	-0.53445	26.70921	0
15607206		0	0	2.46E-07	60.93	1.21E-06	3	1811.896	1.279274	19.4473	0
15607206		0	0	0.00066	60.6	8.69E-07	2	1063.627	1.033655	49.53152	0
15607206		0	0	1.06E-06	58.8	2.04E-06	2	1295.6275	0.61569	49.77706	0
15607206		0	0	9.6E-08	58.61	2.55E-06	3	1795.9345	0.384811	33.49833	1
15607206		0	0	5.94E-06	57.64	3.1E-06	2	1376.6816	0.758965	25.42329	0
15607206		0	0	4.05E-06	51.22	1.28E-05	2	1035.5574	-0.77316	15.58522	0
15607206		0	0	0.000134	48.52	4.01E-05	2	1203.6481	0.02292	20.60263	1

15607206	M22(Oxid	0	0	0.3159	46.82	4.89E-05	2	3015.5315	0.366582	36.4336	0
15607206		0	0	1.05E-05	46.7	3.31E-05	3	2338.252	0.233941	27.05341	1
15607206		0	0	7.63E-05	42.49	8.74E-05	3	1612.7887	1.245756	28.38823	1
15607206		0	0	6.43E-06	41.27	0.000149	3	2614.279	0.181127	38.24167	1
15607206	N-Term(A	0	0	0.02958	38.03	0.000441	2	1541.8171	0.018096	28.24087	0
15607206	N-Term(A	0	0	1.28E-06	37.52	0.000389	2	1636.8174	-0.19299	29.07223	0
15607206		0	0	4.82E-05	37.21	0.000295	2	1121.5944	-0.51119	17.49129	0
15607206		0	0	0.001699	35.53	0.000518	3	1905.9488	-0.32826	43.66145	1
15607206		0	0	0.000122	34.6	0.000347	2	1065.6069	1.583907	21.14904	0
15607206		0	0	1E-05	32.07	0.00149	3	2999.5378	0.764656	38.80759	0
15607206		0	0	0.000447	31.5	0.001487	2	1173.6598	0.993761	20.22164	1
15607206		0	0	6.8E-06	30.82	0.000826	2	915.43151	-0.46555	15.50254	0
15607206		0	0	0.000198	25.03	0.005025	2	1222.6161	-1.13268	16.56265	1
15607206		0	0	0.2045	18.82	0.014434	2	822.43498	-0.77475	18.10999	0
15607206		0	0.005	0.4244	22.92	0.005093	2	1054.6364	-0.38986	20.37815	1
15608597		0	0	2.3E-14	105.61	3.85E-11	2	2250.2413	0.798686	42.36528	0
15608597		0	0	8.13E-09	79.97	2.01E-08	2	1500.7328	-0.07038	32.24529	0
15608597		0	0	9.36E-09	65.95	4.57E-07	2	1716.9104	-0.25238	45.08467	0
15608597		0	0	2.5E-11	56.12	3.67E-06	3	2317.1092	-0.15763	34.75769	1
15608597	M6(Oxidat	0	0	5.54E-16	42.28	6.21E-05	2	2132.1897	-0.07806	49.10046	0
15608597		0	0	0.005485	41.62	7.23E-05	2	835.3947	0.250922	22.01693	0
15607351		0	0	0.07821	95.24	6.13E-10	2	1414.7527	-0.6914	30.77828	0
15607351		0	0	0.1034	91.37	9.12E-10	2	1587.8942	-0.6793	48.91044	0
15607351		0	0	0.09907	89.87	2.68E-09	3	2359.211	0.403265	46.23949	1
15607351		0	0	0.2841	70.14	2.08E-07	2	1101.6264	0.080855	26.84754	0
15607351		0	0	0.1453	66.81	5.42E-07	3	2220.1737	0.224715	27.2179	1
15607351		0	0	0.2348	66.14	6.57E-07	2	1119.5799	0.473645	27.59131	0
15607235		0	0	0.1474	92.08	1.42E-09	2	1549.7767	0.527577	33.39378	0
15607235	C3(Carban	0	0	0.02722	91.13	7.69E-10	3	1790.7733	0.061005	19.96738	1
15607235	M6(Oxidat	0	0	0.116	90.8	1.62E-09	2	1528.7582	0.294363	27.42418	0
15607235	N-Term(A	0	0	0.03244	88.18	2.81E-09	2	1591.7826	-2.36586	34.1725	0
15607235	C2(Carban	0	0	0.04168	80.33	9.25E-09	2	1634.6724	0.215077	19.90142	0
15607235		0	0	0.0536	79.62	2.29E-08	3	1705.8764	-0.30679	31.45115	1
15607235		0	0	0.1139	78.91	2.18E-08	2	1512.7635	0.405902	30.98426	0
15607235	C3(Carban	0	0	0.01577	75.71	2.68E-08	3	1806.7668	-0.70595	15.93021	1
15607235	C8(Carban	0	0	0.0671	53.81	4.15E-06	2	1432.595	0.404163	16.20199	0

15607235		0	0	0.1148	48.42	1.44E-05	3	1376.8009	-0.00278	22.27033	0
15607235	C2(Carban	0	0	0.1226	45.25	2.98E-05	3	1650.6659	-0.63404	16.8112	0
15607235		0	0	0.2914	34.96	0.000686	2	1668.8636	-0.24466	27.96165	1
15607235		0	0	0.3866	32.94	0.000507	2	1532.9017	-0.26249	20.83404	1
15607235		0	0	0.2026	32.12	0.000829	3	1267.6751	-0.22703	17.48952	1
15608452		0	0	0.008357	74.6	5.55E-08	2	1035.5468	-0.17457	32.95705	0
15608452		0	0	0.002893	60.23	2.32E-06	2	1164.5901	-0.68334	35.29856	0
15608452		0	0	0.0233	56.38	2.3E-06	2	870.41051	0.031852	19.85745	0
15608452		0	0	0.01773	54.2	9.5E-06	2	1191.6486	0.424512	25.11958	1
15608452		0	0	0.006812	47.49	5.26E-05	2	773.45189	0.348516	35.28761	0
15608452		0	0	0.05077	44.13	5.8E-05	2	1189.5735	-1.16628	19.50543	1
15608452		0	0	0.1292	43	9.02E-05	2	929.55229	-0.48153	21.16253	1
15608452		0	0	0.01429	42.6	7.42E-05	2	1426.7432	-0.41328	29.34903	1
15607367		0	0	1.06E-19	118.48	3.05E-12	2	2393.2281	-1.14755	35.91608	0
15607367		0	0	1.7E-13	99.69	2.04E-10	4	3717.8954	1.265541	48.74585	0
15607367	M33(Oxid	0	0	1.1E-09	90.23	1.56E-09	4	3733.8834	-0.5819	44.35201	0
15607367		0	0	2.19E-07	79.68	2.26E-08	2	1613.8403	0.468609	29.13944	1
15607367		0	0	8.51E-13	77.18	4.31E-08	2	2233.1585	3.189905	34.64764	0
15607367		0	0	2.56E-05	72.89	9.25E-08	2	1628.8449	1.040573	47.51477	0
15607367		0	0	0.000163	64.29	6.52E-07	2	994.48564	1.610885	27.76266	0
15607367	M10(Oxid	0	0	2.3E-06	61.94	1.57E-06	2	1131.6188	-0.2689	20.87325	0
15607367		0	0	1.15E-05	51.22	2.23E-05	3	1784.9443	-0.00686	33.11961	1
15607367		0	0	0.000299	43.91	9.35E-05	3	2070.0946	1.245037	38.6737	1
15607367		0	0	4.29E-06	32.71	0.001447	4	2041.0911	-1.35033	24.89121	2
15607367		0	0	0.000131	31.89	0.002006	2	1115.6231	-1.00108	25.04726	0
15607367		0	0	0.02163	26.24	0.004991	2	1271.7251	-0.14629	21.68712	1
15608660	M12(Oxid	0	0	5.71E-16	73.41	9.58E-08	2	1678.8866	1.952472	25.60953	0
15608660		0	0	5.71E-16	72.46	1.02E-07	2	1662.888	-0.27918	28.67113	0
15608660		0	0	1.44E-11	58.77	2.39E-06	3	2771.4898	2.583441	45.67285	0
15608660	M18(Oxid	0	0	1.04E-14	45.95	3.81E-05	2	2151.0623	-0.75444	33.54521	1
15608660		0	0	0.000133	40.37	0.000147	2	885.55138	-0.33102	23.66366	1
15608660		0	0	7.18E-08	37.88	0.000236	3	2306.1462	0.373357	38.44363	0
15608127	M5(Oxidat	0	0	0.0808	90.5	1.16E-09	2	1565.736	-0.26619	26.28688	0
15608127		0	0	0.04328	73.42	6.82E-08	2	1549.7427	0.782117	30.02072	0
15608127	M5(Oxidat	0	0	0.08167	67.13	3.39E-07	3	2138.0537	-0.50151	24.81781	1
15608127		0	0	0.04992	62.33	1.02E-06	2	1383.7226	-0.10083	20.11288	0

15608127		0	0	0.1268	61.53	1.09E-06	2	1500.7607	0.80387	29.276	1
15608127		0	0	0.2349	58.26	1.49E-06	2	1284.602	0.906903	29.54601	0
15608127	M3(Oxidat	0	0	0.1034	54.72	4.22E-06	2	1237.5905	-0.8885	22.48568	0
15608127		0	0	0.1116	47.47	4.21E-05	2	1205.603	0.980067	26.53286	0
15608127		0	0	0.09961	42.48	0.000138	3	2122.0603	0.23354	27.68211	1
15608127		0	0	0.2028	25.05	0.009535	3	2584.335	-0.6486	29.51221	0
15607347		0	0	0.000157	91.29	1.3E-09	2	1865.9603	-0.10109	30.89548	0
15607347	C6(Carban	0	0	0.01864	71.42	7.19E-08	2	1388.6431	1.050453	34.7185	0
15607347	M1(Oxidat	0	0	0.02222	59.41	1.26E-06	2	1420.6736	-0.07371	16.95591	0
15607347	M2(Oxidat	0	0	4.42E-06	53.79	9.82E-06	3	2706.3865	-0.59028	30.42411	1
15607347		0	0	0.02952	53.42	9.55E-06	3	2416.2138	1.398042	22.2334	0
15607347		0	0	0.003375	50.75	1.68E-05	3	2696.3404	1.092562	27.84779	0
15607347		0	0	0.3375	41.3	8.9E-05	2	1404.6788	-0.04468	20.03838	0
15607347		0	0	0.2711	35.73	0.000601	2	1348.7214	-0.4821	30.81098	0
15607668	N-Term(A	0	0	1.73E-13	109.61	1.42E-11	3	2787.52	-9.93128	38.22785	2
15607668		0	0	5.34E-16	100.62	1.99E-10	2	1630.8954	0.123883	35.47333	0
15607668		0	0	5.34E-16	88.88	2.98E-09	2	1183.6426	-0.34265	19.69519	0
15607668		0	0	5.34E-16	80.55	8.79E-09	4	4555.1642	-1.34485	37.90196	0
15607668		0	0	5.34E-16	75.9	3.21E-08	3	2589.438	0.760821	34.54827	1
15607668		0	0	4.89E-11	74.28	3.72E-08	3	2461.3449	1.568852	37.35268	0
15607668	C14(Carba	0	0	5.34E-16	74.02	9.11E-08	2	1696.8444	0.473025	25.38531	0
15607668		0	0	5.34E-16	72.81	1.07E-07	2	2455.2049	0.29337	33.0463	0
15607668		0	0	1.08E-12	71.66	6.81E-08	2	1527.709	1.015032	21.9354	0
15607668		0	0	1.29E-07	67.84	3.95E-07	3	2325.2298	-0.55944	49.71127	0
15607668		0	0	1.03E-09	64	8.76E-07	3	2072.0136	-0.99775	45.12619	0
15607668		0	0	1.92E-07	59.28	2.12E-06	3	1822.9881	-0.10368	21.90362	1
15607668		0	0	8.05E-12	53.75	4.21E-06	2	976.56822	-0.49913	17.46178	0
15607668		0	0	5.34E-16	51.68	6.78E-06	4	2745.5382	0.406993	34.80551	2
15607668	N-Term(A	0	0	5.34E-16	50.91	1.87E-05	3	2367.2457	1.716914	35.2503	0
15607668		0	0	1.3E-15	46.98	2.81E-05	3	2939.4439	0.098831	31.55768	1
15607668	N-Term(A	0	0	5.34E-16	46.87	2.88E-05	3	2114.0273	0.520602	30.79674	0
15607668	N-Term(A	0	0	5.34E-16	33.69	0.001069	2	1672.9083	1.46716	43.35682	0
15607668		0	0	0.2624	30.29	0.001543	3	1683.8075	-0.67292	19.63765	1
15607668		0	0	1.63E-08	25.83	0.00431	5	3683.8936	0.141331	38.77903	1
15607668	N-Term(A	0	0	0.000587	22.07	0.008071	2	1018.5788	-0.42443	20.81728	0
15608209		0	0	5.91E-17	98.35	1.75E-10	2	1995.9802	-0.18595	33.38138	0

15608209		0	0	1.42E-12	87.5	3.2E-09	2	1286.6377	0.102291	25.80353	0
15608209	M12(Oxid	0	0	1.47E-05	87.2	2.86E-09	2	2011.974	-0.6907	49.0693	0
15608209		0	0	3.06E-11	76.77	2.21E-08	2	1689.7898	1.799763	34.27333	0
15608209		0	0	0.00012	72.5	1.24E-07	2	1810.8893	3.400829	29.19157	0
15608209	M14(Oxid	0	0	0.000152	71.97	1.24E-07	3	2942.4737	0.197691	38.86577	0
15608209		0	0	8.93E-12	71.11	1.67E-07	2	2039.0452	-2.26463	36.45708	0
15608209	M18(Oxid	0	0	0.000393	70.69	1.79E-07	2	2768.4146	2.467867	44.41653	0
15608209	N-Term(A	0	0	9.32E-06	61.87	1.01E-06	2	1852.8918	-1.06054	33.16155	0
15608209		0	0	1.79E-08	54.7	3.38E-06	2	2483.1961	0.407225	39.61191	0
15608209		0	0	0.004902	54.59	7.3E-06	3	2752.4079	-1.80941	46.51455	0
15608209		0	0	1.83E-08	50.1	2.35E-05	3	2926.4769	-0.45429	42.55127	0
15608209		0	0	0.000267	47.97	3.11E-05	2	1048.5423	0.059728	24.41149	0
15608209		0	0	1.18E-06	47.74	3.62E-05	2	2345.1922	-0.15991	38.83663	0
15608209		0	0	0.000105	47.59	4.01E-05	3	1668.9078	0.562482	39.80349	1
15608209		0	0	0.003362	46.69	5.36E-05	2	1790.9528	0.332274	20.3204	1
15608209		0	0	0.1015	41.33	0.000162	3	1563.8117	-1.46542	21.24595	0
15608209		0	0	0.005163	33.83	0.001304	2	1344.7419	-0.35874	33.6784	1
15608209	N-Term(A	0	0	0.000106	33.4	0.000754	2	2053.9915	2.678383	27.59028	0
15608209	N-Term(A	0	0	0.1206	29.02	0.002882	3	2810.4219	1.273066	42.38535	0
15608660		0	0	0.01295	117.73	2.11E-12	2	1681.9233	0.008367	43.85251	0
15608660		0	0	0.02569	101.11	1.36E-10	2	1934.0374	1.638962	43.79677	0
15608660	M10(Oxid	0	0	0.09212	100.78	8.34E-11	2	1407.6637	2.122373	30.84932	0
15608660		0	0	0.010912	80.72	1.44E-08	3	3485.7524	0.448461	42.04279	0
15608660	M15(Oxid	0	0	0.01657	75.97	2.91E-08	2	2612.2637	-0.95198	36.5825	0
15608660	M10(Oxid	0	0	0.08594	74.75	3.34E-08	2	1423.6562	0.354179	25.42691	0
15608660		0	0	0.1082	73.06	7.66E-08	2	1413.7222	-0.8458	24.80749	0
15608660		0	0	0.08764	67.2	4E-07	2	2637.3821	-0.85449	34.44441	0
15608660		0	0	0.2861	64.68	9.36E-07	3	1541.8165	-1.21925	21.81586	1
15608660	M10(Oxid	0	0	0.211	61.79	1.66E-06	2	1399.7637	1.62397	32.56904	0
15608660		0	0	0.2507	59.31	2.23E-06	2	1383.7662	-0.26769	34.75659	0
15608660		0	0	0.2637	56.54	3.22E-06	3	2596.2751	1.474536	40.29927	0
15608660		0	0	0.289	56.43	3.53E-06	2	1264.6279	-0.17878	21.4014	0
15608660	M4(Oxidat	0	0	0.116	56.21	5.86E-06	3	2983.5177	0.750137	46.161	0
15608660		0	0	0.2969	50.67	1.71E-05	2	1061.5837	-0.08999	21.15849	0
15608660		0	0	0.4343	49.88	2.57E-05	4	3021.5973	0.261267	35.2726	1
15608660	M7(Oxidat	0	0	0.4661	42.42	0.000126	3	2107.0301	1.095468	32.55078	0

15607425	M12(Oxida	0	0	0.1877	97.06	3.94E-10	2	1663.8362	0.09192	34.97602	0
15607425		0	0	0.1674	88.69	2.84E-09	2	1963.0241	-0.17109	32.34687	0
15607425		0	0	0.1768	72.87	1.14E-07	2	1541.8696	0.572769	32.4018	0
15607425		0	0	0.2186	71.46	1.29E-07	2	1184.6999	0.055204	28.30137	0
15607425		0	0	0.1352	70.9	1.63E-07	2	1668.81	-0.96307	25.17789	0
15607425		0	0	0.1206	58.72	1.34E-06	2	1312.794	-0.60054	26.85193	1
15607425		0	0	0.08865	55.85	2.59E-06	3	2272.2735	1.483251	44.69248	0
15607425		0	0	0.3986	54.97	3.18E-06	2	1152.663	0.564056	21.67626	0
15607425		0	0	0.2504	53.54	6.2E-06	2	2922.4019	0.479663	32.70151	0
15607425		0	0	0.4529	52.11	1.45E-05	2	932.48332	-0.36176	27.06731	0
15607425		0	0	0.3777	52.02	9.73E-06	2	1254.6259	-0.07907	17.77629	0
15607425		0	0	0.4298	49.91	1.02E-05	2	1081.6294	0.176428	33.69802	0
15607425		0	0	0.3942	47.04	5.54E-05	2	1088.5854	0.545154	24.70353	1
15607425		0	0	0.2886	40.36	0.000212	2	1234.6539	-0.06083	21.65062	1
15607425		0	0	0.4018	34.69	0.000934	2	802.44072	-1.32081	14.52436	0
15607425		0	0	0.2025	29.33	0.001342	2	1213.7288	2.01533	22.51204	1
15607425		0	0	0.2765	24.21	0.004552	2	1451.8355	-1.03626	33.20301	0
15607425	M12(Oxida	0	0	0.3481	22.22	0.009897	3	1817.9108	-0.47062	30.39547	1
15607425		0	0	0.3356	20.49	0.017419	3	1645.8169	0.794368	34.71651	0
15608016		0	0	0.3958	48.86	2.47E-05	3	1365.7149	-1.4519	15.24226	1
15608016		0	0	0.3448	42.24	8.66E-05	2	1524.7677	-1.05164	25.84194	0
15608016		0	0	0.2947	38.78	0.000411	2	931.51738	-0.93366	24.79122	0
15608016	M5(Oxidat	0	0	0.4305	19.69	0.014499	2	1138.5788	0.111235	13.02856	0
15608048	M7(Oxidat	0	0	0.02782	126.19	2.4E-13	2	1962.8844	-0.93703	27.60519	0
15608048		0	0	0.00946	103.29	4.68E-11	2	1946.8859	-2.80419	47.77439	0
15608048		0	0	0.3813	45.14	3.05E-05	2	1278.8248	-0.73245	26.83684	0
15607218	M4(Oxidat	0	0	1.03E-14	78.33	1.47E-08	2	2132.021	-0.32486	31.37224	0
15607218		0	0	3.18E-08	57.83	2.23E-06	2	1568.8893	-0.93791	27.85112	1
15607218		0	0	1.06E-14	57.11	2.72E-06	2	2116.0271	0.154019	34.46828	0
15607218		0	0	0.01009	46.78	2.09E-05	2	952.55535	-2.26323	14.39804	1
15607218	M5(Oxidat	0	0	1.7E-08	44.07	5.29E-05	3	2288.1236	0.338288	30.49323	1
15607218		0	0	0.06857	42.93	5.35E-05	2	1061.6203	-1.12511	15.39374	1
15607218		0	0	0.000258	32.58	0.001353	2	1014.558	-1.16337	15.24502	1
15607218		0	0	0.1769	26.6	0.002953	2	799.48955	-0.23984	14.17672	1
15607218		0	0	0.009799	22.18	0.006039	4	1557.8964	-0.58302	19.74598	1
15608932		0	0	7.04E-17	93.4	6.63E-10	2	1242.6337	0.952244	22.53923	0

15608932		0	0	0.0003	92.1	6.15E-10	2	1697.7591	-1.34324	24.2625	0
15608932		0	0	5.55E-16	88.77	2.72E-09	2	1941.0383	-0.93027	25.93962	1
15608932		0	0	4E-13	86.16	6.66E-09	2	1784.9393	0.176635	30.16571	0
15608932	C4(Carban	0	0	1.66E-12	77.68	4.09E-08	2	1370.7266	1.17806	37.71358	0
15608932		0	0	0.03025	67.93	3.46E-07	2	1511.8172	-0.3539	22.84499	1
15608932		0	0	9.55E-15	66.93	2.43E-07	3	2266.1122	0.977195	30.48956	1
15608932	C6(Carban	0	0	7.1E-10	45.24	5.39E-05	2	1583.8471	-0.31051	32.81864	1
15608932	N-Term(A	0	0	9.43E-08	39.37	0.000208	2	1412.7372	1.182043	44.45985	0
15608932		0	0	0.00006	33.31	0.0007	2	1007.5263	-0.62389	15.66114	1
15608932		0	0	0.04076	31.22	0.001019	2	851.42552	-0.32607	17.37962	0
15608932		0	0	0.0331	18.59	0.029747	4	1941.0403	0.124989	29.58995	1
15607553		0	0	0.126399	82.29	1.36E-08	2	1875.0064	-1.05922	26.45845	0
15607553		0	0	0.2713	81.76	9.67E-09	2	1400.7864	0.544874	25.47988	0
15607553		0	0	0.4284	79.05	2.24E-08	2	1017.5577	0.111528	26.71991	0
15607553		0	0	0.2858	76.4	3.67E-08	2	1237.6183	0.931169	23.21956	0
15607553		0	0	0.467	60.5	1.29E-06	2	2031.1098	0.141553	25.55643	1
15607553		0	0	0.254	39.01	0.000201	2	1821.8804	-1.90057	22.36829	1
15608569		0	0	9.32E-16	99.99	1.7E-10	3	3495.8331	2.440534	34.95447	1
15608569	C12(Carba	0	0	1.22E-11	90.16	9.62E-10	2	1794.808	1.279077	48.46091	0
15608569	M14(Oxid	0	0	2.29E-08	87.43	2.8E-09	2	1903.9123	0.831311	38.21719	0
15608569		0	0	1.83E-06	79.66	2.22E-08	2	3226.6316	-2.39938	36.01105	0
15608569		0	0	1.66E-08	76.21	4.67E-08	2	1887.917	0.6666	44.6232	0
15608569	M7(Oxidat	0	0	1.86E-06	71.78	6.62E-08	2	1353.6205	0.15861	17.13135	0
15608569	M10(Oxid	0	0	8.12E-07	70.56	8.77E-08	2	1443.6667	-0.40364	21.71117	0
15608569	C1(Carban	0	0	0.000123	59.51	1.12E-06	2	1067.4572	-0.30388	21.41996	0
15608569		0	0	0.001359	57.13	1.93E-06	2	853.52568	0.259001	27.18291	0
15608569		0	0	0.000304	56.03	2.49E-06	2	1337.6227	-1.99835	22.0941	0
15608569		0	0	0.000266	53.93	4.04E-06	2	1473.8821	2.229742	26.77319	0
15608569		0	0	0.001018	51.28	7.43E-06	2	1089.4961	0.128545	18.51877	0
15608569		0	0	0.04756	44.4	9.08E-05	2	1358.6807	-0.35302	19.95212	1
15608569		0	0	0.000824	44.19	7.62E-05	2	1129.6564	-1.04601	24.61743	0
15608569	C2(Carban	0	0	0.01353	40.79	8.32E-05	3	1223.5579	-0.63776	19.16784	1
15608569		0	0	0.000218	39.26	0.000119	3	2107.1812	0.002466	29.73426	1
15607193	C6(Carban	0	0	2.48E-08	81.78	1.06E-08	2	2639.2715	0.639068	38.51988	0
15607193	M2(Oxidat	0	0	1.02E-05	67.57	3.06E-07	3	2370.1811	2.104534	48.98714	0
15607193	M2(Oxidat	0	0	0.000326	63.26	1.2E-06	3	2386.1739	1.203172	48.70123	0

15607193	C3(Carban	0	0	1.37E-05	62.73	5.32E-07	2	1518.6941	-1.6237	15.7256		0
15607193		0	0	3.51E-10	59.05	1.24E-06	3	1690.8026	-0.31041	27.44627		0
15607193		0	0	3.11E-05	58.53	2.45E-06	2	921.47856	-0.35603	17.69303		0
15607193		0	0	0.00182	56.37	2.3E-06	2	1002.474	-1.22269	16.41848		0
15607193	C6(Carban	0	0	0.00499	46.74	4.55E-05	3	3174.5511	0.565522	39.62387		1
15608948		0	0	0.1199	69.7	1.18E-07	2	1732.813	-0.4071	28.53535		0
15608948		0	0	0.02823	66.02	4.25E-07	3	2828.4768	1.541286	31.7726		1
15608948		0	0	0.07123	66.01	5.51E-07	3	2216.0956	0.592893	24.64408		1
15608948	M17(Oxid	0	0	0.09686	63.8	9.38E-07	3	2844.4673	-0.04852	30.43547		1
15608948		0	0	0.1633	56.4	4.01E-06	2	1006.5166	0.185617	18.62986		0
15608948	M17(Oxid	0	0	0.1929	54.75	8.37E-06	2	2446.241	0.575616	30.46684		0
15608948		0	0	0.2591	42.11	0.000138	2	1710.9247	0.063659	19.24538		1
15608948		0	0	0.3123	33.88	0.00133	2	1442.7874	0.748422	18.22611		1
15608948	C3(Carban	0	0	0.3444	32.9	0.000872	3	1491.8123	1.484925	20.14356		1
15608948		0	0	0.4834	25.56	0.008617	4	2430.2445	-0.06738	32.03932		0
15608122		0	0	0.00549	98.39	3.84E-10	2	1511.8425	-0.27547	29.80747		0
15608122		0	0	0.1537	45.58	4.43E-05	2	1243.7111	-0.53399	21.47913		1
15608122		0	0	0.195	44.04	6.9E-05	2	1278.701	1.71583	26.42811		1
15608122		0	0	0.09213	38.7	0.000189	2	839.40868	0.746561	23.67531		0
15608122	M5(Oxidat	0	0	0.2053	35.9	0.000256	2	855.40349	0.612204	20.58787		0
15608122	M9(Oxidat	0	0	0.1485	33.4	0.000823	3	1294.6893	-3.39242	24.3671		1
15608122		0	0	0.3027	24.46	0.004476	2	1399.8129	0.01608	18.4602		2
15608122	N-Term(A	0	0	0.2447	24.35	0.003664	2	881.41802	-0.68067	26.07945		0
15608122		0	0	0.2294	14.61	0.077836	3	1667.8302	1.159019	18.84064		2
15607161		0	0	1.59E-09	100.81	9.96E-11	2	1325.7784	-0.31935	47.09935		0
15607161		0	0	0.002663	99.64	1.68E-10	2	1349.743	0.419503	41.34886		0
15607161		0	0	0.008365	86.79	2.09E-09	2	1742.8099	1.079149	28.26743		0
15607161		0	0	0.002629	69.12	2.63E-07	3	3049.5739	1.64496	34.9025		1
15607161		0	0	4.43E-05	66.96	2.01E-07	3	2656.5064	1.140787	37.29014		1
15607161		0	0	0.000203	65.39	5.49E-07	3	1784.9747	-1.10183	26.95917		1
15607161		0	0	1.39E-05	46.63	3.26E-05	2	2107.0054	0.136399	24.51732		1
15607161		0	0	0.000134	42.6	5.48E-05	2	838.51451	-0.05768	20.19304		0
15607161		0	0	0.1287	39.88	0.000103	2	845.52056	0.217749	15.77886		1
15607161		0	0	0.1427	34.63	0.000344	2	1465.8831	-1.29414	25.5711		1
15607245		0	0	3.81E-05	113.08	1.16E-11	2	1649.8338	1.288938	33.93509		0
15607245	M14(Oxid	0	0	0.01442	103.79	7.1E-11	2	1665.8287	1.251377	25.78146		0

15607245	M17(Oxid	0	0	0.000108	103.16	1.16E-10	2	1979.0282	0.752663	44.37909	0
15607245		0	0	0.02548	94.39	8.92E-10	2	1963.0335	0.842352	48.68791	0
15607245		0	0	0.009001	77.56	2.89E-08	4	3518.7609	-0.31725	33.65187	0
15607245		0	0	0.07957	59.74	1.06E-06	2	2048.1624	-0.73245	41.00307	0
15608170		0	0	5.1E-05	84.11	9.51E-09	2	1261.674	-0.57345	24.82767	0
15608170		0	0	7.7E-06	82.15	1.16E-08	2	1288.6486	-0.48433	18.72202	0
15608170		0	0	0.004868	60.38	2.52E-06	2	1030.5728	1.31015	26.81579	0
15608170	M1(Oxidat	0	0	0.003675	60.29	2.81E-06	2	1046.5672	0.783486	22.54574	0
15608170		0	0	0.0918	60.24	1.42E-06	2	964.48436	-0.36261	18.8199	0
15608170		0	0	4.67E-08	57.6	2.87E-06	3	1862.9222	0.961926	21.2892	1
15608170		0	0	0.05188	30.13	0.00165	2	1444.7514	0.719205	17.2618	1
15608877	M19(Oxid	0	0	0.33457	88.31	2.51E-09	3	3158.544	-1.18276	30.20928	1
15608877	C6(Carban	0	0	0.3511	74.92	3.21E-08	2	1688.7374	-0.03139	32.92666	0
15608877	M18(Oxid	0	0	0.5525	66.07	3.58E-07	3	3002.4406	-2.02182	31.23917	0
15608877		0	0	0.6568	60.13	1.5E-06	3	2986.4569	1.741874	33.83284	0
15608877		0	0	0.5555	50.08	9.82E-06	2	902.54552	-0.36841	26.44034	0
15608877	M7(Oxidat	0	0	0.68	41.54	8.07E-05	2	1036.5719	-0.16331	20.78865	1
15608877		0	0	0.66	37.05	0.000197	2	1058.6464	-0.5303	23.65205	1
15608877		0	0	0.6458	31.45	0.000931	2	1020.5779	0.652692	26.44665	1
15608877	N-Term(A	0	0	0.6333	31.25	0.001387	2	944.55675	0.352503	34.94224	0
15608884	C2(Carban	0	0	0.01165	68.53	1.4E-07	2	1208.5492	-1.54182	14.05298	0
15608884	C2(Carban	0	0	0.008591	42.78	5.26E-05	2	1224.545	-0.85814	10.3726	0
15608884	C3(Carban	0	0	0.2567	19.47	0.02655	3	1147.5631	-0.50611	12.10258	2
15607816		0	0	0.006636	89.87	1.03E-09	3	3174.4999	0.721294	35.21663	0
15607816		0	0	0.01952	82.25	1.28E-08	2	1815.9087	0.191477	24.64953	0
15607816	M12(Oxid	0	0	0.1366	41.74	6.68E-05	3	3827.7663	0.564099	35.44199	0
15607591		0	0	0.068358	80.34	9.23E-09	2	1955.9039	0.477985	27.92034	0
15607591		0	0	0.1669	58.18	1.52E-06	2	1080.4778	-0.10227	26.892	0
15607591		0	0	0.2593	57.45	2.7E-06	2	1197.6277	1.212268	32.08222	0
15607591		0	0	0.2653	57.03	3.57E-06	2	1295.7229	1.803387	25.5919	0
15607591		0	0	0.3255	43.39	4.57E-05	2	2501.1482	-5.27371	37.20059	0
15607591	M8(Oxidat	0	0	0.4777	31.11	0.000773	2	1096.4726	-0.25037	21.14712	0
15607376		0	0.003	0.04188	17.89	0.016255	2	941.48332	-0.66633	20.31505	0
15607376		0	0.007	0.194	26.4	0.004353	2	1176.5912	0.274371	19.21037	0
15608660	M10(Oxid	0	0	0.01418	68.63	2.47E-07	2	1375.7619	0.328632	32.46459	0
15608660		0	0	0.05795	56.73	2.23E-06	2	1359.7677	0.901988	41.65423	0

15608660		0	0	0.2657	48.99	3.28E-05	3	2603.3629	0.456291	43.44338	0
15608660		0	0	0.2989	43.05	4.95E-05	2	1215.591	0.327973	34.97079	0
15608660		0	0	0.2238	41.14	9.61E-05	2	1184.5916	0.801148	16.01596	1
15608660		0	0	0.3589	28.36	0.002553	2	2038.9938	-2.15267	33.14544	1
15608951		0	0	1.27E-07	66.2	4.92E-07	3	2600.2738	-2.15918	33.03819	0
15608951		0	0	1	12.32	0.08499	3	1703.809	0.705955	34.50431	0
15608136		0	0	0.5732	33.04	0.000495	3	2672.1661	-1.06299	33.09152	0
15607535		0	0	5.32E-10	105.98	3.28E-11	2	2208.1873	0.068906	38.85358	1
15607535	C3(Carban	0	0	1.13E-13	102.6	6.87E-11	2	1592.7597	0.719414	25.97796	0
15607535		0	0	2.24E-06	92.13	1.56E-09	2	1527.8006	-0.53449	29.71708	0
15607535		0	0	1.17E-11	85.47	2.83E-09	5	4101.2739	0.748972	48.86734	1
15607535	N-Term(A	0	0	0.000597	51.65	8.55E-06	3	2250.212	6.351824	40.40341	1
15607535		0	0	5.21E-05	49.11	1.22E-05	2	1174.5326	-0.75002	14.84863	0
15607985		0	0	5.78E-08	135.21	8.14E-14	2	1760.9219	0.409614	47.81114	0
15607985	M8(Oxidat	0	0	7.43E-12	133.95	8.26E-14	2	1776.9191	1.687573	38.13756	0
15607985		0	0	5.77E-07	97.7	1.78E-10	2	1712.9823	0.949282	41.37668	1
15607985	N-Term(A	0	0	1.33E-05	89.08	2.04E-09	2	1144.5951	-0.52065	29.2617	0
15607985	C1(Carban	0	0	5.19E-06	89.03	1.25E-09	2	1966.8871	1.29253	29.12929	0
15607985	C8(Carban	0	0	2.38E-06	87.87	2.69E-09	2	2707.2908	-0.24608	33.84649	1
15607985	C1(Carban	0	0	6.62E-08	87.7	1.69E-09	2	1950.8888	-0.42737	30.32852	0
15607985		0	0	0.000209	86.51	3.8E-09	2	1684.8385	-0.21529	30.37818	0
15607985		0	0	2.66E-05	86.19	4.33E-09	2	873.51445	-0.96095	37.46905	0
15607985		0	0	3.09E-06	84.2	6.08E-09	2	1485.8448	1.543136	44.17545	0
15607985	N-Term(A	0	0	6.22E-05	83.96	8.44E-09	2	1300.728	1.473461	40.66753	0
15607985		0	0	1.64E-05	82.1	1.36E-08	2	1788.961	0.343103	33.53082	0
15607985		0	0	3.98E-07	80.92	1.21E-08	3	1945.0598	-0.87593	33.27588	1
15607985		0	0	3.49E-07	77.22	4.46E-08	2	1258.7153	-0.16981	39.73797	0
15607985		0	0	7.2E-06	75.8	3.16E-08	2	2204.2112	1.695817	35.77255	0
15607985		0	0	0.000164	75.49	4.66E-08	2	1577.8578	2.804045	31.01343	1
15607985		0	0	1.29E-07	75.22	5.26E-08	2	1402.7652	-0.77489	20.77728	1
15607985	C8(Carban	0	0	1.03E-05	75.17	4.41E-08	2	2723.2864	0.008905	32.68176	1
15607985		0	0	0.000154	71.93	7.37E-08	2	1102.5865	1.180932	45.08084	0
15607985		0	0	9.23E-08	69.33	1.75E-07	3	3130.701	0.035654	39.68839	1
15607985		0	0	2.66E-05	68.24	1.5E-07	2	2473.3968	1.655543	35.54476	1
15607985		0	0	0.00036	67.01	4.48E-07	2	1192.6209	0.021724	27.99421	0

15607985		0	0	0.000127	65.03	5.5E-07	2	2615.4141	-1.73632	44.35054	1
15607985	M17(Oxid	0	0	2.06E-05	61.89	1.1E-06	3	2631.4107	-1.09736	42.635	1
15607985		0	0	0.001208	57.8	5.23E-06	2	929.54033	-1.25922	25.35817	0
15607985		0	0	3.27E-06	54.91	7.59E-06	2	1997.0242	-2.85614	31.32871	1
15607985		0	0	5.77E-05	54.65	9.94E-06	2	1246.6641	-0.83519	23.38565	0
15607985	N-Term(A	0	0	0.000454	54.07	9.79E-06	2	1802.9319	0.092108	45.04561	0
15607985		0	0	0.000469	46.35	4.06E-05	3	3454.7809	-0.16916	42.51757	1
15607985	N-Term(A	0	0	0.007202	35.53	0.000532	3	1987.0741	1.013297	38.16168	1
15607985		0	0	0.01894	35.16	0.000533	2	1473.8005	-1.98715	18.85133	1
15607985		0	0	0.002358	33.05	0.000718	3	1629.9027	-1.12168	18.83572	2
15607985		0	0	0.007577	30.14	0.001404	2	986.50072	-0.71193	12.23509	0
15607985		0	0	0.004766	25.21	0.00467	4	3610.8842	0.44717	42.37064	2
15607985	M17(Oxid	0	0	0.04497	22.71	0.006965	4	2787.5204	2.052056	42.53981	2
15607985		0	0	0.04748	15.54	0.027925	4	3766.9713	-0.29656	42.35787	2
15607985	N-Term(A	0	0.002	0.07074	30.25	0.00151	2	2765.3191	8.018639	35.77039	1
15607985		0	0.002	0.09214	28.09	0.003959	3	1897.0254	0.148887	25.07404	1
15607985		0	0.004	0.1314	34.63	0.000826	2	1371.7713	0.675428	24.5778	1
15608459		0	0	1.52E-05	121.05	1.26E-12	2	1700.8432	-1.09008	25.1599	0
15608459		0	0	6.2E-05	113.79	1E-11	2	1747.8869	0.393114	45.95467	0
15608459		0	0	5.11E-05	104.21	7.4E-11	2	1490.7765	0.942835	37.03189	0
15608459	N-Term(A	0	0	6.83E-05	101.82	1.84E-10	2	1789.8947	-1.15391	41.214	0
15608459		0	0	0.000349	93.64	8E-10	3	2714.4693	2.498525	36.42383	0
15608459		0	0	0.00044	87.54	3.88E-09	2	2564.3443	-2.33019	37.4432	0
15608459	M5(Oxidat	0	0	0.000852	83.66	9.26E-09	2	2580.3448	-0.15585	33.36923	0
15608459		0	0	0.004423	78.79	2.91E-08	2	1308.696	0.946859	44.2157	0
15608459	C2(Carban	0	0	0.007245	70.2	1.43E-07	2	1281.6716	-1.34115	16.27476	1
15608459		0	0	0.01169	68.6	3.17E-07	2	1369.7221	-0.17361	29.55022	0
15608459		0	0	0.006807	66.32	4.78E-07	3	2545.2912	0.715668	41.39912	1
15608459		0	0	0.002302	66.03	4.24E-07	2	1368.7229	-0.1047	20.9024	0
15608459		0	0	0.00454	61.17	1.22E-06	2	1078.5451	-1.11238	29.98044	0
15608459		0	0	0.003243	57.74	3.45E-06	2	2108.1382	-0.86812	34.14927	1
15608459		0	0	0.1902	56.92	3.56E-06	4	2840.4877	2.573609	41.82678	1
15608459		0	0	0.02849	56.58	2.2E-06	2	1156.57	-0.51654	15.10096	0
15608459	N-Term(A	0	0	0.006536	54.78	3.83E-06	2	1742.8519	-2.15283	24.91849	0
15608459		0	0	0.1567	46.65	6.49E-05	2	816.42119	0.157856	13.01288	0
15608459		0	0	0.01114	46.38	4.6E-05	2	1678.8914	0.121342	25.65126	0

15608459		0	0	0.05334	39.98	0.000301	2	816.42113	0.095345	15.05583		0
15608459		0	0	0.09065	38.58	0.000243	2	1963.0517	0.39838	24.15579		1
15608459		0	0	0.07157	33.43	0.000953	4	2701.3937	1.172048	36.39362		2
15608459		0	0	0.2922	33.41	0.001003	2	862.48906	-0.40584	26.07726		1
15608459		0	0	0.2629	32.71	0.001366	3	1265.6697	-1.05118	16.2096		1
15608459		0	0	0.2986	22.09	0.010506	2	1953.9687	-2.62838	20.16143		1
15608459		0	0.003	0.3684	23.38	0.005051	2	824.40105	0.093735	4.661297		0
15608459		0	0.008	0.3932	14.39	0.080061	4	2175.1029	-1.16561	29.43022		1
15608071		0	0	2.08E-13	97.15	1.92E-10	2	2416.1409	-1.69881	30.2013		0
15608071	M21(Oxid	0	0	1.33E-07	90.44	9.02E-10	2	2432.1399	0.001594	44.48416		0
15608071		0	0	1.14E-07	61.61	9.66E-07	3	2816.5041	-0.00987	45.39937		0
15608071		0	0	1.18E-05	28.74	0.003609	5	3934.9788	-0.90071	45.16689		0
15608071		0	0	0.000911	12.81	0.052236	3	3508.6424	-1.1378	40.44787		0
15608071		0	0.003	0.03965	30.17	0.000959	2	872.55559	-0.96465	17.96076		1
15607433	C7(Carban	0	0	0.002898	88.09	1.55E-09	2	1095.5622	0.544093	24.33079		0
15607433		0	0	0.000635	87.68	2.47E-09	2	1296.7267	-0.27695	28.38648		0
15607433		0	0	0.000332	73.41	7.75E-08	3	2013.0765	0.019822	40.79504		0
15607433		0	0	0.007076	67.81	3.06E-07	2	2339.1492	-0.98488	38.84331		0
15607433		0	0	0.003314	61.43	7.18E-07	2	1297.5795	-0.97561	21.09503		0
15607433		0	0	0.05141	58.3	2.88E-06	2	1566.8497	0.622698	31.78828		0
15607433		0	0	0.002372	56.46	4.74E-06	3	3044.5406	-1.051	37.57315		1
15607433		0	0	0.06417	55.99	3.9E-06	3	1610.8985	0.727164	24.74832		1
15607433		0	0	0.000109	46.84	2.07E-05	2	2533.439	0.020187	46.54967		0
15607433		0	0	0.00043	45.55	5.71E-05	2	2378.2063	-1.53682	40.74113		0
15607433		0	0	0.01545	42.23	5.97E-05	2	880.51189	0.780363	17.39643		1
15607433		0	0	0.01563	35.12	0.000307	3	1641.743	-0.52151	21.00141		1
15607433	M2(Oxidat	0	0	0.09195	26.52	0.002223	3	1657.7376	-0.68906	19.89533		1
15607433		0	0	0.1276	23.91	0.00691	5	3214.6873	0.100431	35.86439		0
15607433		0	0	0.05529	13.34	0.062565	2	1452.8266	-1.11893	23.22193		1
15607433		0	0	0.1669	11.39	0.13433	3	1611.8595	-0.46599	25.89953		2
15607433		0	0.003	0.2476	18.3	0.014756	2	1055.5633	-0.00121	22.96104		1
15607245		0	0	4.83E-16	120.91	1.38E-12	3	3173.644	2.09374	49.31247		1
15607245		0	0	4.83E-16	108.79	2.44E-11	2	1483.8497	1.142753	40.78781		0
15607245	M10(Oxid	0	0	4.83E-16	107.01	3.58E-11	3	3189.6214	-3.40267	44.49254		1
15607245		0	0	4.83E-16	104.63	8.61E-11	2	2324.1768	0.203991	36.99368		0
15607245		0	0	4.83E-16	102.14	6.41E-11	2	2013.1344	0.221402	44.18935		1

15607245	M25(Oxid	0	0	1.59E-12	96.52	3.01E-10	3	3344.6836	-1.67114	39.13885	1
15607245		0	0	3.27E-14	95.41	6.47E-10	2	1015.5522	-0.90457	27.09216	0
15607245		0	0	2.76E-11	87.22	2.85E-09	2	1462.7919	1.241746	40.75571	0
15607245		0	0	2.52E-15	82.3	1.03E-08	3	2270.1311	-1.01476	34.9715	0
15607245	M16(Oxid	0	0	4.83E-16	80.84	1.61E-08	2	2340.1678	-1.48455	34.51656	0
15607245		0	0	2.64E-09	75.31	6.48E-08	3	2120.0991	0.35513	33.62181	1
15607245	C4(Carban	0	0	2.1E-15	66.76	2.53E-07	2	1398.6797	0.178596	20.13198	0
15607245	M19(Oxid	0	0	1.02E-15	64.79	6.64E-07	3	2286.1263	-0.89252	30.72579	0
15607245		0	0	6.03E-07	55.75	2.93E-06	2	1016.5988	0.109366	30.44244	0
15607245	M8(Oxidat	0	0	4.51E-09	50.06	2.12E-05	3	2920.4361	-3.7758	39.93403	0
15607245		0	0	0.001325	48.5	2.75E-05	2	1002.5591	1.245144	26.33477	0
15607245		0	0	2.06E-05	43.39	4.57E-05	2	946.5703	-1.88284	32.84447	0
15607245		0	0	2.31E-07	42.46	0.000111	3	2904.4544	0.777974	40.04803	0
15607245		0	0	0.000338	42.4	8.34E-05	2	1023.535	-0.77339	28.93467	0
15607245	N-Term(A	0	0	0.000285	41.62	7.23E-05	2	1440.6873	-1.90662	20.65301	0
15607245	N-Term(A	0	0	0.01018	40.74	0.000181	4	3231.642	-0.26699	37.99931	1
15607245	M19(Oxid	0	0	2.15E-05	38.34	0.000271	3	2996.5312	-1.56621	34.71262	1
15607245		0	0	5E-07	36.5	0.000437	3	3328.6988	1.377222	40.61695	1
15607245		0	0	5.86E-12	32.38	0.001156	3	2980.5415	0.180088	39.48021	1
15607245	N-Term(A	0	0.005	0.09088	29.98	0.002009	2	1044.5688	0.312937	33.11044	0
15608488		0	0	4.97E-16	109.76	1.64E-11	2	1977.9526	0.62739	46.40529	0
15608488		0	0	4.97E-16	95.69	6.88E-10	3	2346.2078	1.221769	32.26935	1
15608488		0	0	4.97E-16	85.56	5E-09	2	1623.8726	1.524068	45.69172	0
15608488		0	0	8.86E-11	73.74	6.55E-08	2	1138.5743	0.303053	38.1112	0
15608488		0	0	1.53E-09	72.64	1.12E-07	2	1222.654	0.046589	23.59441	0
15608488		0	0	3.98E-15	72.16	9.12E-08	2	1210.6202	2.16706	23.05867	0
15608488		0	0	4.97E-16	69.79	2.36E-07	2	1707.9087	-2.93476	21.15379	1
15608488		0	0	1.81E-12	69.3	1.53E-07	3	2306.2838	0.462358	36.03698	1
15608488		0	0	7.09E-11	68.64	2.39E-07	2	1944.0762	-0.63363	27.05862	1
15608488		0	0	1.44E-15	67.74	4.71E-07	2	2143.1275	0.91348	28.08033	1
15608488		0	0	1.31E-08	66.12	2.44E-07	2	1179.698	1.970289	23.1235	1
15608488		0	0	4.97E-16	65.43	4.3E-07	2	3682.9541	-0.32098	39.42589	0
15608488	N-Term(A	0	0	4.72E-07	63.94	4.44E-07	2	1252.6269	-0.98006	23.34272	0
15608488	N-Term(A	0	0	8.66E-09	63.38	6.2E-07	2	1180.5835	-0.79843	26.43731	0
15608488		0	0	0.005245	60.88	8.15E-07	2	945.56999	-2.96902	31.46719	0
15608488		0	0	3.4E-11	55.89	5.15E-06	3	1863.9551	-0.5097	28.98604	1

15608488	N-Term(A	0	0	0.001726	53.89	4.07E-06	2	950.57713	-1.13752	29.15726	0
15608488		0	0	4.46E-10	48.3	3.25E-05	4	3472.7914	1.909485	45.11687	0
15608488	C14(Carba	0	0	3.06E-05	47.93	2.09E-05	2	1689.8091	-2.0746	21.82787	1
15608488		0	0	2.34E-05	47.18	1.91E-05	2	908.56822	0.630187	35.24454	0
15608488		0	0	0.000713	40.48	0.000184	2	1199.6282	0.150152	17.9298	1
15608488		0	0	4.94E-07	37.22	0.000189	3	1549.9292	0.374879	22.09103	2
15608488		0	0	0.000658	34.57	0.000646	2	1719.9472	-1.70944	26.67102	1
15608488		0	0	5.74E-06	33.94	0.000989	3	1141.6431	-0.55294	18.17798	1
15608488		0	0	5.53E-06	30.42	0.001498	3	2262.1126	0.690364	29.08533	1
15608488	N-Term(A	0	0	0.000689	11.9	0.138816	3	2388.227	4.826844	34.98921	1
15608488	N-Term(A	0	0.002	0.04984	39.29	0.000318	2	1264.6626	-1.45576	28.91568	0
15608488		0	0.002	0.05327	38.39	0.000159	2	857.53142	-0.21181	17.7089	1
15608488		0	0.007	0.1211	25.85	0.002594	2	868.42723	0.047623	10.62447	0
15608109		0	0	5.4E-16	111.77	1.33E-11	2	2173.1863	-0.04804	37.97087	0
15608109		0	0	5.4E-16	92.11	1.41E-09	2	2327.2081	-0.99084	29.55597	0
15608109		0	0	5.4E-16	89.52	1.56E-09	2	1792.9847	1.609294	36.21306	0
15608109		0	0	5.4E-16	89.52	2.23E-09	2	3071.6006	-0.10345	42.83327	1
15608109		0	0	5.4E-16	87.74	4.21E-09	2	2582.3191	-0.73264	31.88658	0
15608109	M4(Oxidat	0	0	5.4E-16	87.09	3.32E-09	2	1813.9045	-0.21487	28.48084	0
15608109		0	0	5.4E-16	86.53	4.56E-09	2	1797.9076	-1.27978	31.59742	0
15608109		0	0	5.4E-16	81.02	9.88E-09	2	899.55535	-0.84025	30.92985	0
15608109		0	0	5.4E-16	80.47	2.42E-08	2	958.53166	-0.00781	21.38716	0
15608109		0	0	5.4E-16	73.83	5.17E-08	2	1299.7654	1.751976	26.32089	1
15608109	M4(Oxidat	0	0	5.4E-16	69.45	1.59E-07	2	1829.901	0.698002	25.78253	0
15608109		0	0	5.4E-16	62.78	5.26E-07	2	917.43236	-0.00184	17.46498	0
15608109		0	0	5.4E-16	62.65	1.09E-06	3	2710.4134	-0.97294	30.50374	1
15608109		0	0	8E-11	60.89	1.18E-06	4	2866.5233	2.168074	30.90465	2
15608109	N-Term(A	0	0	5.4E-16	60.45	1.8E-06	3	2369.2177	-1.34211	37.59951	0
15608109	M1(Oxidat	0	0	9.94E-08	58.98	3.35E-06	2	1261.6657	-1.98887	21.01308	1
15608109		0	0	9.84E-13	54.92	8.05E-06	2	1245.6718	-1.19676	22.24494	1
15608109		0	0	5.4E-16	50.65	1.12E-05	2	1111.6349	-0.78803	25.71361	0
15608109		0	0	1.83E-13	49.83	1.14E-05	2	936.50371	-0.01238	26.0892	0
15608109		0	0	3.28E-12	47.8	1.83E-05	2	1011.568	-1.45474	16.90538	1
15608109	N-Term(A	0	0	0.02039	26.11	0.006857	2	1000.5432	0.962631	21.95458	0
15608109		0	0	0.02574	17.65	0.017138	5	3319.7661	1.231474	38.24192	2
15608460	C8(Carban	0	0	0.002832	100.53	8.83E-11	2	2878.3909	-0.93405	30.50221	0

15608460		0	0	0.002652	87	3.99E-09	2	2476.2205	0.074825	47.53032	0
15608460		0	0	0.00604	85.05	6.88E-09	2	1549.7778	1.230021	31.81965	0
15608460		0	0	0.007477	81.98	1.81E-08	2	1161.626	-0.23858	47.29808	0
15608460	C2(Carban	0	0	0.01891	80.15	1.84E-08	2	1323.6777	3.826161	23.60005	1
15608460		0	0	0.01063	80.02	2.39E-08	2	1612.8234	0.186354	31.72461	0
15608460		0	0	0.002568	75.71	5.1E-08	2	1694.8389	0.243968	32.42146	0
15608460		0	0	0.01348	71.84	8.18E-08	2	988.57921	0.605815	26.25867	0
15608460	M5(Oxidat	0	0	0.003378	70.21	1.38E-07	2	2894.388	-0.18424	28.94287	0
15608460	N-Term(A	0	0	0.05358	53.77	1.15E-05	2	1203.6365	-0.28588	34.39398	0
15608460	M5(Oxidat	0	0	0.01432	52.28	5.9E-06	4	4570.2052	-0.86635	35.11406	1
15608460	C16(Carba	0	0	0.03347	51.86	1.24E-05	3	1962.9646	-0.85716	22.03752	1
15608459;156		0	0	0.1546	50.63	2.59E-05	2	816.42095	-0.14118	13.09	0
15608460	N-Term(A	0	0	0.01902	49.32	2.16E-05	2	2518.2376	2.664612	40.87699	0
15608460		0	0	0.05338	47.24	2.45E-05	2	1351.6967	0.134329	24.75494	0
15608460	N-Term(A	0	0	0.03221	46.27	5.19E-05	2	1393.705	-1.49432	22.32159	0
15608459;156		0	0	0.1842	45.06	7.49E-05	2	1265.6686	-1.86036	15.76128	1
15608460		0	0	0.1231	43.25	0.000102	2	1170.6265	3.31544	16.11131	1
15608459;156		0	0	0.2118	42.14	0.000134	2	862.48937	-0.052	23.30796	1
15608460	C8(Carban	0	0	0.02929	40.16	9.62E-05	4	4554.1925	-4.77356	35.89103	1
15608460		0	0	0.1699	17.96	0.046387	3	1965.032	0.939056	30.08566	1
15608488		0	0	5.26E-16	97.02	3.08E-10	2	1977.9518	0.257097	30.4559	0
15608488		0	0	5.26E-16	72.77	1.08E-07	2	1623.8669	-2.00904	30.38558	0
15608488		0	0	5.26E-16	55.82	3.67E-06	2	1210.6187	0.957066	20.0481	0
15608488		0	0	5.26E-16	48.53	3.51E-05	3	2346.2049	-0.02692	33.90816	1
15608488		0	0	0.02054	22.08	0.008672	3	3682.9669	3.138981	48.91383	0
15608489		0	0	3.31E-05	125.51	6.33E-13	2	1458.7808	0.601884	32.31237	0
15608489		0	0	6.69E-06	113.42	7.28E-12	2	1970.9777	-0.09263	32.64056	0
15608489		0	0	8.68E-05	103.55	1.15E-10	2	1602.8351	1.060188	33.80361	0
15608489		0	0	0.000714	90.36	9.18E-10	2	2093.9556	1.534276	34.85916	0
15608489		0	0	0.001186	70.74	8.41E-08	2	1590.9338	0.686464	40.49837	0
15608489	C6(Carban	0	0	0.001443	66.74	5.08E-07	2	2458.2422	-1.09436	33.89854	0
15608489		0	0	0.01692	59.19	2.59E-06	2	2094.0748	-0.33269	43.53834	0
15608489		0	0	0.01127	54.08	8.01E-06	3	1801.9619	-0.59551	36.25687	0
15608489		0	0	0.01529	53.08	1.18E-05	3	2504.3082	3.573584	38.15256	1
15608489		0	0	0.006012	51.83	2.17E-05	2	815.4619	-0.35645	21.31143	0
15608489	M10(Oxid	0	0	0.005222	49.72	2.77E-05	2	1817.9571	-0.47166	34.25936	0

15608489		0	0	0.07787	30.67	0.000855	2	870.42516	-0.65926	30.84161	0
15608489		0	0	0.05304	30.16	0.002458	2	920.48271	-1.02955	26.46471	0
15608489		0	0	0.03633	26.2	0.005157	4	3077.5656	0.543015	45.58755	0
15608489		0	0	0.07715	20.6	0.014371	3	1399.7066	-0.82422	24.78309	1
15607156		0	0	0.001433	103.2	1.1E-10	2	1430.7587	-0.79448	29.28662	0
15607156		0	0	0.000403	90.13	2.23E-09	2	2078.0347	-1.40793	40.69736	0
15607156		0	0	0.000238	89.86	2.12E-09	2	1466.7846	-0.28484	32.22049	0
15607156	M12(Oxid	0	0	0.00105	77.28	3.27E-08	2	2094.0342	0.797975	38.21855	0
15607156		0	0	0.001426	53	0.000005	2	969.44444	-1.48201	16.06612	0
15607156		0	0	0.01868	48.71	1.75E-05	2	981.49913	-0.92637	19.88373	0
15607156		0	0	0.02579	34.91	0.000549	2	908.46471	-1.32427	28.5909	0
15607156		0	0	0.0429	34.79	0.000514	2	1324.6556	-0.80483	21.84161	0
15607156		0	0	0.03071	31.81	0.000658	2	897.4464	-0.13773	27.6756	0
15607156	M9(Oxidat	0	0	0.03905	27.43	0.002801	2	1340.6506	-0.73547	18.98677	0
15607156		0	0	0.06039	23.52	0.005113	2	899.5667	-0.70402	25.9469	1
15607156		0	0.004	0.163	20.89	0.015479	3	1423.731	-1.36306	28.8681	1
15607156		0	0.004	0.1723	15.89	0.055391	3	2819.4118	-1.87023	33.46189	0
15608881	M3(Oxidat	0	0	5.09E-16	103.46	4.5E-11	2	1514.6424	-0.30682	26.53812	0
15608881	C4(Carban	0	0	5.09E-16	101.66	6.81E-11	2	2277.0569	0.174376	34.60625	0
15608881		0	0	5.09E-16	94.22	1.1E-09	2	1646.8366	0.470083	21.06385	0
15608881		0	0	5.09E-16	80.92	1.5E-08	3	2807.3835	0.429476	35.37832	0
15608881		0	0	5.09E-16	77.71	1.69E-08	2	1498.6482	0.206625	23.57534	0
15608881	M23(Oxid	0	0	5.09E-16	70.53	1.46E-07	3	2823.3753	-0.64709	34.33698	0
15608881		0	0	2.95E-07	68.14	1.53E-07	2	1097.4998	-1.15521	16.34462	0
15608881	M14(Oxid	0	0	5.09E-16	60.94	1.97E-06	2	2043.0168	-2.37642	31.17282	0
15608881	M2(Oxidat	0	0	1.56E-14	58.9	2.19E-06	2	1497.7244	-0.69002	28.32182	0
15608881	N-Term(A	0	0	5.09E-16	54.91	3.22E-06	2	1540.657	-0.95172	22.46142	0
15608881		0	0	2.87E-10	54.73	3.36E-06	2	835.47826	-0.30312	19.23026	0
15608881		0	0	3.37E-13	52.22	1.08E-05	2	1481.7311	0.401827	30.86251	0
15608881		0	0	5.09E-16	51.29	1.67E-05	2	2027.0221	-2.25403	34.52909	0
15608881	M7(Oxidat	0	0	1.17E-05	49.65	1.08E-05	3	1939.9186	0.322652	22.63898	1
15608881		0	0	9.19E-06	46.84	2.07E-05	2	991.57878	-0.85555	16.69003	1
15608881		0	0	0.000298	44.41	3.62E-05	2	1017.478	-0.25297	15.0296	1
15608881	C4(Carban	0	0	3.03E-09	36.73	0.000456	3	2946.4374	-0.04521	35.91409	1
15608881		0	0	0.008552	32.91	0.00051	2	1783.7898	-1.01401	25.30561	1
15608881	M3(Oxidat	0	0	0.02806	30.73	0.000843	2	1799.785	-0.89266	21.80107	1

15608881		0	0	0.02353	28.58	0.003883	2	1444.7892	-1.05146	21.71777	0
15608881		0	0	0.000422	28.55	0.001955	3	1923.921	-1.04873	26.39828	1
15608777	C17(Carba	0	0	0.000672	81.8	8.26E-09	2	1996.9612	-0.31851	29.53173	0
15608777		0	0	0.001426	73.73	4.23E-08	2	2197.0552	-0.41324	40.15698	0
15608777	M31(Oxid	0	0	0.0985	32.68	0.000538	3	3293.5125	-1.32987	36.63061	0
15608777	M13(Oxid	0	0	0.166	17.94	0.016069	3	2983.3969	-3.67586	36.08345	0
15607733		0	0	0.006845	99.9	2.3E-10	2	2068.0176	-1.27445	36.14309	0
15607733		0	0	0.007235	94.72	7.08E-10	2	1642.9131	0.488301	39.76945	0
15607733		0	0	0.002341	83.04	1.12E-08	2	1377.7117	-0.32211	49.30462	0
15607733		0	0	0.009934	81.32	2.1E-08	2	1101.6053	0.082523	47.31201	0
15607733		0	0	0.01125	79.83	2.13E-08	2	1755.9659	1.040232	45.42495	0
15607733		0	0	0.009706	79.79	1.68E-08	2	1696.8293	0.15908	26.66737	0
15607733	M7(Oxidat	0	0	0.00211	74.95	6.4E-08	2	2084.0137	-0.69905	31.43102	0
15607733		0	0	0.03252	68.57	2.5E-07	2	1868.0274	-0.17663	42.34562	0
15607733		0	0	0.01798	68.28	2.67E-07	2	3012.5333	0.600632	44.04206	0
15607733	M5(Oxidat	0	0	0.006217	67.48	1.78E-07	2	1174.5413	0.315923	23.24403	0
15607733		0	0	0.02581	66.67	3.44E-07	2	1896.909	0.138038	25.90723	1
15607733	M1(Oxidat	0	0	0.0134	63.97	6.61E-07	2	2100.0083	-0.82996	28.97033	0
15607733		0	0	0.03127	63.1	8.82E-07	3	2070.0088	0.647765	32.1499	1
15607733		0	0	0.02495	62.58	5.51E-07	2	1158.5456	-0.38106	27.57906	0
15607733		0	0	0.006481	60.86	8.18E-07	2	1004.5777	0.132411	25.64781	0
15607733		0	0	0.1226	45.47	4.82E-05	2	1356.7737	-0.73119	29.38204	1
15607733		0	0	0.163	45.1	5.25E-05	2	930.47844	-0.85993	24.68254	0
15607733		0	0	0.03709	44.09	3.89E-05	2	2692.5027	-0.22958	35.02396	0
15607733		0	0	0.0361	40.23	0.000142	2	822.41002	-0.56001	15.20187	0
15607733		0	0	0.01332	40.23	9.46E-05	3	2384.3598	-1.50351	29.25561	0
15607733	M13(Oxid	0	0	0.02502	39.11	0.00019	3	2086.0028	0.242315	29.11117	1
15607733		0	0	0.1768	38.49	0.000368	3	1919.9936	0.05892	28.40365	1
15607733	N-Term(A	0	0	0.1465	38.12	0.000362	2	1684.9225	-0.21565	42.49854	0
15607733		0	0	0.03552	35.15	0.000305	2	1123.5375	-0.30094	45.7627	0
15607733		0	0	0.1634	25.6	0.006059	3	1685.9315	-1.14455	34.08744	1
15607733	N-Term(A	0	0	0.09896	18.95	0.012705	3	2426.3712	-1.1529	32.83831	0
15607733	N-Term(A	0	0.003	0.2068	25.36	0.004657	2	864.42064	-0.46903	17.48988	0
15607591		0	0	9.4E-06	107.97	1.59E-11	2	1909.891	1.449041	32.75833	1
15607591		0	0	0.02657	103.7	8.96E-11	2	1519.779	0.280937	34.79953	0
15607591	M1(Oxidat	0	0	0.03346	89.97	1.01E-09	2	1925.8852	1.034919	31.39085	1

15607591	M8(Oxidat	0	0	0.00864	83.2	4.77E-09	2	1618.717	2.125291	19.79748	0
15607591	N-Term(A	0	0	0.02082	81.06	7.82E-09	2	1644.7287	-0.29853	33.3671	0
15607591	M1(Oxidat	0	0	5.65E-05	77.96	1.6E-08	2	1634.7077	-0.46008	19.51346	0
15607591		0	0	0.009581	75.74	4.27E-08	2	1412.8112	0.264384	38.64307	0
15607591		0	0	0.002417	73.61	4.34E-08	2	1602.7199	0.801719	27.73164	0
15607591		0	0	0.02692	63.29	1.01E-06	2	1193.6047	-0.20898	37.45601	0
15607591		0	0	0.03089	62.25	5.94E-07	2	1844.8682	-1.21398	29.5207	0
15607591		0	0	0.000301	61.69	1.05E-06	3	1477.7246	1.929097	24.48357	1
15607591	M1(Oxidat	0	0	0.05484	60.43	9.04E-07	2	1941.8802	1.067652	25.74599	1
15607591		0	0	0.000533	53.6	8.95E-06	3	3641.8366	-1.35873	34.16596	0
15607591	M2(Oxidat	0	0	0.01051	53.08	6.4E-06	3	2375.1245	0.753464	32.67188	0
15607591		0	0	0.1026	52.55	6.67E-06	2	1147.5477	-1.26904	19.19682	0
15607591	M1(Oxidat	0	0	0.000249	52.18	6.04E-06	2	1650.7041	0.480317	14.4727	0
15607591		0	0	0.02758	48.16	2.44E-05	2	1788.8456	0.166226	35.20919	0
15607591	M2(Oxidat	0	0	0.01105	46.78	2.2E-05	3	2874.3321	-4.1644	37.24889	1
15607591	M5(Oxidat	0	0	0.1104	45.93	4.21E-05	2	1535.774	0.330179	28.14648	0
15607591	M23(Oxid	0	0	0.00151	39.53	0.000223	3	3657.8335	-0.8303	31.65041	0
15607591	M19(Oxid	0	0	0.07816	39.36	0.000122	3	2343.1307	-0.94569	34.48531	0
15607591		0	0	0.00399	38.43	0.000143	3	2733.2525	-0.57285	33.43372	1
15607591		0	0	0.006634	36.15	0.000267	2	1996.9369	-1.53938	34.50803	0
15607591		0	0	0.07131	35.36	0.00029	2	1039.4424	-1.05911	17.65503	0
15607591	M5(Oxidat	0	0	0.01545	35.01	0.000473	3	2359.1294	0.646996	34.13754	0
15607591	M1(Oxidat	0	0	0.03349	34.96	0.000318	3	1860.8664	0.570646	27.67417	0
15607591		0	0	0.002968	34.83	0.000625	4	2327.1378	-0.07443	39.54426	0
15607591	M12(Oxid	0	0	0.01187	34.14	0.000482	3	3673.8435	3.282033	30.58516	0
15607591	C2(Carban	0	0	0.04015	26.09	0.002829	2	1595.7254	0.463799	35.7838	0
15607591		0	0	0.0702	25.97	0.003415	3	2858.3498	0.204418	40.15745	1
15607591	M2(Oxidat	0	0	0.04909	21.7	0.008451	2	2391.1158	-0.79505	28.35679	0
15607591		0	0	0.07003	18.25	0.028428	4	2659.2938	1.418665	38.62907	1
15607591	M6(Oxidat	0	0	0.04737	17.36	0.018322	3	2906.3298	-1.41732	31.7404	1
15607591	M1(Oxidat	0	0	0.1144	11.1	0.077441	4	1876.8589	-0.73201	25.74984	0
15607591		0	0.004	0.1978	49.8	3.04E-05	2	1131.6044	-0.0827	32.94816	0
15607591	M15(Oxid	0	0.004	0.1293	30.09	0.002106	3	1960.0146	-1.20192	36.70842	1
15607591		0	0.004	0.1313	28.18	0.003497	2	798.49858	0.10201	27.0117	0
15607425		0	0	2.01E-11	102.8	1.08E-10	2	2220.1109	1.363059	33.01094	1
15607425		0	0	5.81E-12	93.68	5.14E-10	2	1594.9362	0.748322	46.40479	0

15607425	M5(Oxidat	0	0	5.61E-16	85.44	3.71E-09	2	1610.9317	1.09373	40.21957	0
15607425	M4(Oxidat	0	0	5.61E-16	83.16	8.21E-09	2	3055.5342	1.13924	40.33176	0
15607425		0	0	7.46E-06	78.6	2.14E-08	2	1082.5961	0.716458	27.89785	0
15607425	M10(Oxid	0	0	5.61E-16	73.88	5.93E-08	2	2079.9983	-1.59504	31.2187	0
15607425	M9(Oxidat	0	0	0.000654	73.51	9.58E-08	2	1583.8553	-1.38529	28.68977	0
15607425		0	0	4.72E-07	72.76	5.3E-08	2	1763.9788	-0.87526	36.27932	0
15607425		0	0	4.16E-05	69.54	1.89E-07	2	2064.0069	0.068913	34.76883	0
15607425		0	0	4.61E-08	69.42	2E-07	3	1342.6893	-0.28088	21.33755	0
15607425		0	0	9.37E-06	67.94	2.41E-07	2	1567.8625	-0.04908	33.81679	0
15607425		0	0	7.33E-07	65.25	6.87E-07	2	2371.1536	-1.2438	38.06499	0
15607425	M6(Oxidat	0	0	0.000163	63.97	1.12E-06	3	1358.683	-1.16197	18.26591	0
15607425		0	0	0.005959	63.14	4.84E-07	2	920.51982	-0.23716	28.93573	0
15607425		0	0	5.83E-05	59.93	1.83E-06	3	1415.7126	-0.93396	16.19851	0
15607425		0	0	9.42E-05	59.21	1.2E-06	2	875.53136	0.488085	21.15107	1
15607425		0	0	0.005537	58.39	3.62E-06	2	1058.5448	-0.35322	25.74776	0
15607425	M11(Oxid	0	0	1.76E-05	57.41	3.81E-06	2	2236.0979	-2.15926	30.06682	1
15607425		0	0	0.000547	57.16	1.92E-06	2	1110.4799	-1.05837	15.59789	0
15607425		0	0	0.000123	52.06	1.4E-05	3	3039.5352	-0.21085	48.16008	0
15607425		0	0	8.86E-09	46.42	4.45E-05	3	1774.883	-0.07411	26.47363	1
15607425		0	0	0.001349	46.31	5.96E-05	3	2527.2678	4.001983	36.61095	1
15607425		0	0	0.002399	37.57	0.000175	2	1076.6211	-0.01853	27.47497	1
15607425		0	0	0.000261	34.99	0.000507	2	1723.9627	-0.56684	29.62183	1
15607425		0	0	6.92E-06	27.14	0.002222	3	2827.5547	-1.10684	34.90263	1
15607425	M4(Oxidat	0	0.003	0.03961	46.34	4.76E-05	2	1074.5398	-0.2734	20.86659	0
15608447		0	0	0.1835	112.98	5.02E-12	2	2233.2522	-1.30591	47.33485	0
15608447		0	0	0.2944	103.2	8.62E-11	2	1671.8172	-0.79257	24.51376	0
15608447		0	0	0.4142	95.85	3.25E-10	2	1505.7232	0.033758	30.85833	0
15608447		0	0	0.3298	93.61	7.19E-10	2	1200.5972	0.336722	18.87972	0
15608447		0	0	0.3319	81.76	1.1E-08	2	1639.7786	0.159017	15.34457	0
15608447		0	0	0.2223	79.29	2.47E-08	2	1872.0226	-0.01105	47.9309	0
15608447		0	0	0.4671	74.48	7.66E-08	2	1042.5893	0.086033	28.49692	0
15608447		0	0	0.5729	66.04	3.61E-07	2	1054.6252	-0.39031	28.69952	0
15608447	C8(Carban	0	0	0.3087	62.05	1.03E-06	2	1554.8321	0.735481	32.26523	0
15608447		0	0	0.3714	49.75	2.33E-05	3	1812.888	0.250065	36.12445	0
15608447		0	0	0.5035	48.43	3.01E-05	2	1332.679	-0.34414	23.52352	0
15608447		0	0	0.587	37.87	0.000343	3	1941.0137	-0.63641	27.41237	1

15608447	M12(Oxid	0	0	0.5727	30.48	0.002059	4	3531.7775	-0.42465	49.64066	0
15608447		0	0	0.6553	26.6	0.003719	3	1171.6799	0.395108	19.81075	1
15608323		0	0	0.003523	93.95	7.65E-10	2	1164.5756	-0.03622	49.38722	0
15608323	M8(Oxidat	0	0	0.009899	93.21	1.22E-09	2	1547.7781	3.009133	48.99315	0
15608323		0	0	0.006179	80.1	2.59E-08	2	1598.8516	1.162584	31.94137	0
15608323	N-Term(A	0	0	0.2251	73.96	6.43E-08	2	1484.7702	-0.15295	46.39405	0
15608323		0	0	0.1578	50.34	2.27E-05	2	1442.7583	-1.0417	37.18359	0
15608323		0	0	0.2662	28.14	0.00445	2	1156.6506	-0.17378	27.31328	0
15607425	M13(Oxid	0	0	0.010644	100.2	1.62E-10	2	1789.9416	0.569638	35.72299	0
15607425		0	0	0.1246	88.23	3.61E-09	2	1773.9438	-1.05309	38.72027	0
15607425		0	0	0.02857	87.04	3.46E-09	2	2081.0171	1.194677	31.42439	0
15607425		0	0	0.06674	83.07	9.86E-09	2	1537.7836	-1.38145	45.18737	0
15607425		0	0	0.06124	72.43	8.29E-08	2	1137.6414	-0.14616	37.32951	0
15607425		0	0	0.03235	70.77	1.88E-07	2	2020.023	-0.8735	32.84376	0
15607425		0	0	0.01773	68.64	3.69E-07	3	2335.2312	0.780646	39.40195	0
15607425	M18(Oxid	0	0	0.09087	66.49	5.5E-07	3	2432.2399	0.824761	38.48955	1
15607425		0	0	0.2032	64.33	4.24E-07	2	1070.6207	0.151097	30.06602	0
15607425		0	0	0.03549	60.47	1.08E-06	2	1594.9345	-0.32319	46.38641	0
15607425		0	0	0.0561	57.93	3.95E-06	2	1315.7111	0.506091	31.07917	0
15607425		0	0	0.07903	55.06	4.68E-06	2	1253.6015	-0.57695	22.08432	0
15607425	C8(Carban	0	0	0.02574	52.67	6.22E-06	3	1548.7679	-1.36744	17.79867	0
15607425		0	0	0.02926	51.35	7.31E-06	3	2904.3334	-0.10781	40.88713	0
15607425		0	0	0.05692	49.24	1.43E-05	3	2346.2765	1.973798	41.93273	0
15607425		0	0	0.1223	48.65	1.36E-05	2	1108.6476	0.242914	24.75167	0
15607425		0	0	0.04582	44.25	9.02E-05	3	2656.4114	0.707259	45.2879	1
15607425		0	0	0.07584	43.17	8.43E-05	3	1323.6697	0.332461	33.84401	0
15607425		0	0	0.1898	41.44	7.16E-05	2	1566.7377	-1.14429	23.09819	1
15607425		0	0	0.1453	39.42	0.000314	2	1474.7536	-0.07029	22.43539	0
15607425		0	0	0.166	34.98	0.000921	2	832.45207	-0.32114	22.74817	0
15607425		0	0	0.2221	17.8	0.022404	3	2367.3149	0.770165	40.19703	1
15607566		0	0	5.22E-16	114.08	5.47E-12	2	2112.1778	-1.65674	46.23584	0
15607566	M16(Oxid	0	0	5.22E-16	103.76	9.26E-11	2	1999.0645	0.142278	46.30428	0
15607566		0	0	5.22E-16	100.98	1.72E-10	2	2067.0806	-1.15491	34.73964	0
15607566		0	0	2.72E-11	99.79	1.05E-10	2	1094.6449	1.81224	18.78308	1
15607566		0	0	5.84E-16	97.33	3.98E-10	2	2267.222	-3.10065	42.80557	1
15607566		0	0	5.22E-16	91.27	1.12E-09	2	1983.0696	0.164582	49.98752	0

15607566		0	0	5.22E-16	89.99	1.9E-09	3	2223.1858	0.766408	29.7421	1
15607566		0	0	7.02E-13	83.8	5.63E-09	2	1853.0308	1.493778	41.95813	0
15607566		0	0	9.27E-12	78.2	1.51E-08	2	1041.5319	-0.48328	16.45635	0
15607566		0	0	2.7E-10	69.74	1.75E-07	2	1169.6253	-1.78652	15.911	1
15607566		0	0	3.89E-06	67.81	3.64E-07	2	1496.8061	-0.49668	32.3711	0
15607566		0	0	6.98E-11	67.75	2.1E-07	2	2009.1253	-1.92599	40.36609	1
15607566		0	0	2.86E-08	58.1	2.32E-06	2	1452.7699	0.401825	29.26991	0
15607566	N-Term(A	0	0	1.58E-05	52.4	1.41E-05	2	2041.0744	-0.19249	43.14348	0
15607566		0	0	4.27E-08	52.34	5.82E-06	2	1106.6696	1.348156	41.80804	0
15607566		0	0	1.26E-07	52.32	1.11E-05	2	1094.5577	-1.17537	16.71417	0
15607566	C1(Carban	0	0	1.53E-06	52.08	1.61E-05	3	2853.4118	-0.16401	39.79854	0
15607566		0	0	3.54E-05	43.64	4.31E-05	2	960.57213	-0.35519	27.85476	0
15607566		0	0	2.92E-06	43.27	4.7E-05	2	938.54167	-0.17871	18.05742	0
15607566		0	0	6.97E-08	42.25	0.000113	5	4140.0737	0.07135	45.0146	0
15607566	M18(Oxid	0	0	1.75E-06	41.05	0.000165	4	2283.2216	-1.02398	41.45442	1
15607566		0	0	0.000277	37.92	0.000161	2	1234.7616	-1.1638	22.01495	1
15607566	C1(Carban	0	0	5.4E-07	37.32	0.000297	3	2869.3888	-6.38889	37.42968	0
15607566		0	0	0.002354	35.64	0.000272	2	947.57848	-0.08815	41.13774	0
15607566		0	0	0.000765	35.07	0.000327	2	955.49883	-0.84187	18.17436	0
15607566	N-Term(A	0	0	0.01004	31.1	0.000774	2	989.59032	1.204837	29.07921	0
15607566	C2(Carban	0	0	0.05572	17.58	0.044518	3	3009.5213	2.628022	38.21663	1
15607566	N-Term(A	0	0	0.006755	14.21	0.055001	3	2154.1919	0.00991	44.58811	0
15607377		0	0	0.000185	123.99	3.98E-13	3	3811.8519	-1.17514	41.82647	0
15607377		0	0	0.001207	103.36	6.69E-11	3	1920.0498	0.670037	32.56248	1
15607377		0	0	0.000962	102.9	1.38E-10	2	2017.0066	-3.58051	28.61274	0
15607377		0	0	0.006608	93.67	8.59E-10	2	1686.9281	-0.31603	26.21674	0
15607377		0	0	0.002645	91.53	1.16E-09	2	1416.8046	-0.78929	27.39881	0
15607377		0	0	0.0147	87.52	1.77E-09	2	1034.5978	-1.46471	33.50044	0
15607377		0	0	0.000411	86.29	4.35E-09	3	2681.3218	-0.10101	29.32684	1
15607377	M13(Oxid	0	0	0.001678	81.46	1.14E-08	3	1936.0413	-1.09108	26.72123	1
15607377		0	0	0.004773	80.63	9.51E-09	2	1054.5885	-0.61894	40.0199	0
15607377	M12(Oxid	0	0	0.0235	78.54	3.08E-08	2	1779.9445	1.24684	29.52623	0
15607377		0	0	0.003673	76.15	5.7E-08	2	2010.0082	0.082094	39.98633	0
15607377		0	0	0.01711	67.96	2.24E-07	2	1761.9877	1.054035	31.05975	0
15607377		0	0	0.01169	64.72	7.08E-07	2	1763.95	1.489538	32.69693	0
15607377		0	0	0.07929	56.74	6.78E-06	2	788.42656	0.545353	14.92286	0

15607377		0	0	0.07237	56.36	3.01E-06	2	1024.5463	0.079008	29.97135	0
15607377		0	0	0.00663	56.3	5.04E-06	2	2318.1775	-1.78307	34.19262	0
15607377		0	0	0.06757	55.6	6.06E-06	4	2552.3307	1.942492	38.63136	0
15607377		0	0	0.09165	45.51	2.81E-05	2	1046.4743	-0.62165	17.06159	0
15607377		0	0	0.04952	44.82	3.29E-05	3	1742.8192	-0.74206	16.35413	1
15607377		0	0	0.06791	44.64	6.7E-05	2	1145.58	-0.93197	12.07119	0
15607377		0	0	0.07733	43.66	7.96E-05	2	1337.7428	0.332015	30.54013	0
15607377		0	0	0.03233	38.23	0.000271	4	3484.8019	0.149015	47.97169	0
15607377		0	0	0.1134	18.08	0.015523	5	4803.5203	-1.16775	47.43708	1
15607377		0	0	0.1227	12.25	0.092328	4	3715.9774	2.119603	38.22539	1
15607377		0	0.003	0.1791	31.55	0.000698	2	1062.6545	1.265928	23.83502	0
15607377		0	0.005	0.1973	37.44	0.00055	2	900.51457	-0.43013	19.89935	0
15607377		0	0	5.41E-16	104.58	3.48E-11	3	3811.8537	-0.69478	45.58734	0
15607377		0	0	5.42E-16	100.85	1.64E-10	2	1686.9297	0.624684	32.58909	0
15607377		0	0	5.42E-16	99.99	1.6E-10	3	1920.047	-0.76044	30.43096	1
15607377		0	0	9.61E-16	93.45	4.51E-10	2	1034.5984	-0.87476	30.03387	0
15607377	M13(Oxid	0	0	5.42E-16	93.19	7.92E-10	3	1936.0462	1.462499	27.94767	1
15607377		0	0	5.42E-16	88.21	2.49E-09	2	1416.8041	-1.13393	28.21383	0
15607377		0	0	5.42E-16	84.87	7.66E-09	2	2010.0087	0.325019	34.46095	0
15607377		0	0	5.42E-16	84.29	7.26E-09	3	2681.3157	-2.35456	31.29341	1
15607377		0	0	1.25E-13	83.57	4.83E-09	2	1054.5889	-0.27168	33.43768	0
15607377		0	0	5.42E-16	80.03	2.98E-08	2	2017.0114	-1.2202	30.24422	0
15607377	M12(Oxid	0	0	1.55E-14	78.72	3.02E-08	2	1779.9406	-0.94775	34.2387	0
15607377		0	0	5.42E-16	69.91	1.38E-07	2	1761.9883	1.400434	30.49381	0
15607377		0	0	2.67E-11	65.24	6.28E-07	2	1763.9496	1.28193	33.39788	0
15607377	N-Term(A	0	0	5.42E-16	59.84	2.54E-06	2	2059.0201	-2.11711	31.92318	0
15607377		0	0	8.23E-09	58.18	2.13E-06	2	1024.5452	-0.9933	42.07958	0
15607377		0	0	1.08E-12	50.37	1.93E-05	2	2318.178	-1.57244	35.25908	0
15607377		0	0	8.61E-15	47.68	3.33E-05	5	3484.8115	2.912467	45.05658	0
15607377		0	0	5.11E-05	46.64	4.01E-05	2	1337.7438	1.062022	29.41124	0
15607377		0	0	8.69E-06	45.91	8.21E-05	2	788.42674	0.777594	15.58859	0
15607377		0	0	0.1259	45.1	3.08E-05	2	1062.6528	-0.34229	20.64691	0
15607377		0	0	0.007216	43.73	0.000129	2	900.51427	-0.76902	20.17902	0
15607377		0	0	1.32E-09	43.26	0.000123	2	1021.5163	-1.09479	12.73508	0
15607377		0	0	9.97E-07	41.63	6.85E-05	2	1046.4745	-0.38835	17.53162	0
15607377		0	0	0.00327	38.29	0.000148	3	1742.8198	-0.42687	17.12169	1

15607377		0	0	3.5E-08	37.67	0.000291	2	1145.5794	-1.46476	12.66521	0
15607377		0	0	2.21E-10	32.34	0.001342	3	2552.3276	0.728173	39.50536	0
15607377		0	0	3.53E-10	29.11	0.001225	5	4803.5322	1.309978	43.95697	1
15607377	N-Term(A	0	0	0.001945	24.51	0.00708	3	1728.9386	-0.39015	34.58893	0
15607377		0	0	0.000243	20.65	0.016359	4	1588.7942	0.162928	15.57018	1
15607377		0	0	4.9E-06	19.51	0.011168	4	3433.8288	1.71376	41.7151	1
15609300		0	0	0.1004	41.34	0.000187	2	1130.6274	-0.29573	16.39129	1
15609300		0	0	0.3206	37.7	0.000178	2	783.41423	-0.77753	26.01891	0
15609300		0	0	0.122	37.04	0.000346	2	1194.6335	-0.32717	13.91742	0
15609300		0	0	0.2743	34.47	0.001	2	873.47875	-0.17742	16.71107	0
15609300		0	0	0.1916	26.2	0.005397	3	1520.8395	-0.68392	16.63572	1
15609909		0	0	4.1E-11	119.75	2.54E-12	2	1668.8803	-0.05673	37.72687	0
15609909		0	0	2.7E-10	94.65	6.68E-10	2	1119.5793	-0.08938	41.20281	0
15609909	N-Term(A	0	0	0.001621	74.29	7.45E-08	2	1161.5894	-0.45905	38.89226	0
15609909		0	0	0.003675	63.39	4.57E-07	2	1364.5599	-0.21197	25.33473	0
15609909		0	0	0.01464	63.04	1.09E-06	2	1516.8232	0.017415	30.57073	1
15609909		0	0	0.2088	50.6	8.69E-06	2	1520.6626	0.903524	23.13865	1
15609909		0	0	0.000121	48.97	1.26E-05	2	812.40526	0.330748	17.86371	0
15609909		0	0	0.06057	40.25	9.42E-05	2	930.39452	-0.74537	16.36009	0
15609909	N-Term(A	0	0	0.03655	35.34	0.000614	2	1710.888	-1.7355	39.87693	0
15609909		0	0	0.003457	28.36	0.003428	2	1824.983	0.792585	29.7628	1
15609980		0	0	0.2866	61.23	1.73E-06	2	844.48845	-0.34385	20.52217	0
15609980		0	0	0.3972	42.96	5.05E-05	2	967.52074	-0.01321	17.85111	0
15609980		0	0	0.4331	41.64	9.6E-05	3	1726.868	-2.26201	21.59095	1
15610388		0	0	0.1608	54.49	6.05E-06	2	1394.7107	0.257873	41.42768	0
15610388	M9(Oxidat	0	0	0.06271	52.94	1.3E-05	2	1549.7825	-0.45684	34.12379	0
15610388		0	0	0.3177	38.91	0.000128	2	1129.5477	-0.70495	16.15706	0
15610388	M10(Oxida	0	0	0.4402	32.89	0.001311	3	1705.8836	-0.44975	31.93073	1
15609673		0	0	0.08298	82.37	5.78E-09	2	2672.2354	-4.32677	27.37978	0
15609673		0	0	0.1767	81.54	9.47E-09	2	1151.5683	-0.7871	27.29324	0
15609673	M9(Oxidat	0	0	0.05982	72.61	5.47E-08	2	2688.2374	-1.68289	24.49219	0
15609673		0	0	0.2524	56.56	3.2E-06	2	1348.6596	0.350123	20.9298	0
15609673		0	0	0.4385	41.7	0.000108	2	914.49352	-0.77033	19.0342	0
15609673	M20(Oxida	0	0	0.3014	28.3	0.001476	4	3820.7975	1.120122	30.19538	1
15610830		0	0	2.59E-07	92.48	1.13E-09	2	1958.9816	-0.13084	35.66553	0
15610830	M8(Oxidat	0	0	0.000818	77.13	1.93E-08	2	1168.5265	0.171632	14.50005	0

15610830		0	0	0.07667	75.95	2.53E-08	2	1152.5303	-0.95465	19.63638	0
15610830		0	0	3.72E-05	65.06	3.12E-07	2	1180.6418	-1.33831	18.07644	0
15610830		0	0	6.92E-05	54.78	9.31E-06	2	1471.7735	0.124882	43.81581	0
15610830	N-Term(A	0	0	0.05709	52.93	1.04E-05	2	1222.6531	-0.6482	21.51842	0
15610830	M7(Oxidat	0	0	0.02287	51.62	2.2E-05	2	1059.5618	0.216011	21.52116	0
15610830		0	0	0.03068	44.77	9.84E-05	2	1043.5666	-0.0914	21.37662	0
15610830		0	0	0.000654	41.4	0.000163	3	1989.078	1.701666	42.52547	1
15610830		0	0	0.1598	26.17	0.005193	3	1878.9713	0.567507	30.25182	1
15609346		0	0	7.57E-10	65.4	4.33E-07	3	3177.5932	0.243435	36.41128	0
15609346		0	0	0.000157	55.82	2.61E-06	2	1013.5004	-0.74842	16.25993	0
15609346		0	0	1.04E-07	53.11	7.09E-06	2	1108.5724	-1.94485	17.83168	0
15609346	C11(Carba	0	0	0.004517	44.23	7.74E-05	3	1420.7578	-0.91478	15.59308	0
15609346		0	0	9.56E-06	40.76	8.39E-05	2	799.3839	-0.86767	17.17134	0
15609346		0	0	1.95E-08	33.03	0.000548	2	1526.8906	-0.62268	18.62607	0
15609162		0	0	4.1E-05	118.62	2.75E-12	2	2170.087	-0.86155	30.24664	0
15609162		0	0	0.002577	80.12	2.33E-08	4	2733.4389	1.266602	38.37948	0
15609162		0	0	0.01211	77.42	4.08E-08	4	2889.5387	0.759877	37.42964	1
15609162	M9(Oxidat	0	0	0.002551	76.41	5.94E-08	4	2749.4269	-1.24234	34.01108	0
15609162		0	0	0.001244	70.66	1.93E-07	2	1697.8587	-1.13739	20.13769	0
15609162	M10(Oxid.	0	0	0.008677	58.81	2.63E-06	4	2905.5285	-1.02329	34.6241	1
15609162	N-Term(A	0	0	0.002474	50.16	2.31E-05	3	2775.4457	-0.11331	41.47843	0
15609162		0	0	0.01606	38.49	0.000354	3	3075.6408	1.258586	37.29497	1
15610142	M11(Oxid.	0	0	7.34E-12	81.04	7.85E-09	2	1691.7123	-3.25021	18.26901	0
15610142		0	0	0.000095	56.15	2.42E-06	5	4657.2255	-2.49508	38.97372	0

15610011	C1(Carban	0	0	0.385	74.75	3.34E-08	2	1848.8078	-0.25878	21.28228	0
15610011		0	0	0.6105	60.43	1.13E-06	2	1568.8276	-0.24162	48.68504	0
15610011	C2(Carban	0	0	0.5234	46.71	3.84E-05	2	1537.8154	-0.16262	22.61809	0
15610584	C20(Carba	0	0	0.08701	79.62	2.84E-08	3	2386.2599	2.127408	47.14695	0

15610584	C1(Carban	0	0	0.07284	79.31	1.17E-08	3	2418.1186	2.012375	32.80796	0
15610584		0	0	0.07788	76.92	3.86E-08	2	1873.027	-1.11379	22.30081	1
15610584		0	0	0.1461	76.77	2.1E-08	3	3548.9985	-5.06144	47.78586	1
15610584		0	0	0.1519	72.15	6.08E-08	3	4175.3203	0.331548	49.38479	1
15610584		0	0	0.3823	69.98	1E-07	4	4331.4186	-0.32818	46.97317	2
15610584		0	0	0.1942	55.98	2.52E-06	2	951.56182	-0.44781	22.88041	0
15610584	C29(Carba	0	0	0.05611	53.63	7.59E-06	3	3168.6566	-0.56995	34.68262	1
15610584		0	0	0.2967	38.88	0.000129	3	3392.9227	2.16666	47.20688	0
15610584	N-Term(A	0	0	0.3337	35.95	0.000445	3	1915.0497	5.242929	24.36776	1
15610584	C20(Carba	0	0	0.2939	18.51	0.015502	4	3318.8051	1.75639	39.87702	1
57116765		0	0	0.2869	74.73	8.41E-08	2	1527.8194	-0.8167	44.0511	0
57116765		0	0	0.2245	68.02	1.57E-07	2	2332.085	2.65738	24.85931	0
15610979		0	0	0.000834	91	1.51E-09	2	1791.902	-0.27131	26.81149	0
15610979	M2(Oxidat	0	0	0.000346	80.99	1.63E-08	3	1571.7925	0.247056	33.37931	0
15610979	M2(Oxidat	0	0	0.004087	69.88	2E-07	2	1587.7834	-2.31046	28.364	0
15610979		0	0	0.001601	65.14	7.35E-07	3	1555.7962	-0.62575	36.49061	0
15610979	N-Term(A	0	0	0.01277	63.74	8.24E-07	2	1613.8039	0.736959	38.53626	0
15610979		0	0	0.01969	50.32	1.16E-05	2	1387.797	0.89593	26.27325	0
15610979	N-Term(A	0	0	0.01149	49.36	2.32E-05	2	1597.8078	0.006607	43.63819	0
15610979	C1(Carban	0	0	0.07869	47.31	4.27E-05	3	1887.9239	0.012189	31.30162	1
15610979		0	0	0.04679	43.76	6.73E-05	3	1496.781	-0.44918	22.97597	0
15610979	C1(Carban	0	0	0.03733	41.09	0.00014	3	1903.9191	0.15034	26.79456	1
15610979		0	0	0.04214	36.88	0.000246	2	1137.6524	-0.36035	24.07144	1
15610979		0	0	0.1917	31.8	0.001454	3	1652.8817	-0.65602	17.65985	1
15610979	C1(Carban	0	0	0.06007	25.74	0.004267	3	1871.9269	-1.10649	34.00824	1
15610979	M2(Oxidat	0	0	0.217	19.06	0.023591	3	1929.9671	-0.17881	32.59762	1
15610979	M10(Oxid	0	0	0.1003	14.95	0.067177	3	1913.9731	0.256176	35.46109	1
15609424	M5(Oxidat	0	0	3.35E-12	136.06	3.22E-14	2	1907.8997	-1.00136	25.61603	0
15609424		0	0	5.49E-16	133.56	5.51E-14	2	1891.9051	-0.85861	47.45646	0
15609424	M1(Oxidat	0	0	4.58E-07	102.35	1.08E-10	2	1709.8285	0.527836	23.53023	0
15609424		0	0	1.85E-15	85.24	5.24E-09	2	1693.8249	-4.63129	25.14213	0
15609424		0	0	2.54E-14	76.07	7.54E-08	2	1312.7217	-0.20874	45.42843	0
15609424		0	0	0.002636	75.59	5.8E-08	2	2489.2283	-0.75213	30.89489	1
15609424	N-Term(A	0	0	0.001563	68.6	1.73E-07	2	1751.835	-1.82253	26.9547	0
15609424		0	0	3.84E-11	58.75	1.33E-06	2	1205.7217	-0.72489	19.89778	1
15609424		0	0	3.22E-12	57.12	2.52E-06	3	2364.2653	-0.22348	40.84301	0

15609424	M4(Oxidat	0	0	5.01E-10	53.7	6.19E-06	3	2123.0442	-0.48691	25.21279	1
15609424		0	0	1.64E-08	53.07	4.92E-06	2	950.5526	-0.48649	18.26747	0
15609424	M5(Oxidat	0	0	0.01823	52.65	1.17E-05	3	2505.2272	0.838528	28.65916	1
15609424		0	0	0.0561	49.87	2.42E-05	3	2645.3313	-0.01488	28.77909	2
15609424		0	0	1.96E-06	34.86	0.000327	3	2082.1671	0.229141	34.47911	1
15609424		0	0	0.003408	31.5	0.001345	2	788.46208	-0.54228	18.78255	0
15609424	N-Term(A	0	0.004	0.1494	49.61	1.09E-05	2	992.56304	-0.59484	27.53417	0
15611006		0	0	5.82E-16	93.66	7.32E-10	3	3245.6209	-0.53503	45.42897	0
15611006		0	0	5.82E-16	79.75	1.8E-08	2	1140.6008	-0.06636	46.07935	0
15611006		0	0	5.82E-16	77.58	2.71E-08	4	3502.7721	0.181037	37.56817	1
15611006		0	0	3.41E-14	54.31	4.63E-06	2	1753.8414	0.453229	24.6352	0
15611006		0	0	2.07E-10	54.16	3.84E-06	2	913.47319	-0.68877	18.06559	0
15611006	C8(Carban	0	0	7.53E-12	39.17	0.000206	4	1800.9881	-1.19239	29.2729	1
15611006		0	0	4.74E-08	29.51	0.002351	4	2875.4176	-2.06163	29.65271	1
15611023	M20(Oxida	0	0	0.2717	82.28	9.17E-09	3	2288.0735	-0.76783	30.80576	0
15611023		0	0	0.3583	80.82	1.03E-08	3	2272.0812	0.400342	34.73747	0
15611023		0	0	0.2336	66	4.52E-07	2	1788.9264	-0.86762	28.05869	1
15611023		0	0	0.3355	59.2	3.01E-06	3	1926.0218	1.978881	36.21578	0
15611023	C1(Carban	0	0	0.6567	46.17	2.41E-05	2	1142.4963	-0.58283	21.63714	0
15611023		0	0	0.5279	34.37	0.000676	2	1660.8334	0.241037	29.59514	0
15610302		0	0	4.59E-16	130.78	2.01E-13	2	1754.9552	0.203136	38.37714	0
15610302		0	0	4.59E-16	107.38	4.75E-11	2	1359.6938	0.001386	46.34876	0
15610302	M9(Oxidat	0	0	5.01E-13	84.54	6.68E-09	2	1375.6897	0.769479	35.34305	0
15610302	C6(Carban	0	0	2.84E-15	84.03	4.15E-09	2	1441.6818	0.235832	44.3478	0
15610302	M9(Oxidat	0	0	4.05E-11	75.26	6.7E-08	2	1531.7889	-0.61388	47.86223	1
15610302	C6(Carban	0	0	1.35E-13	75.18	4.25E-08	2	1425.6864	-0.07458	45.05338	0
15610302		0	0	3.99E-15	74.52	9.71E-08	3	1515.7944	-0.33983	29.45451	1
15610302		0	0	3.09E-15	71.43	1.12E-07	3	2378.1996	0.352289	47.27254	0
15610302	M9(Oxidat	0	0	4.92E-11	67.31	4.64E-07	2	1913.9903	2.08239	23.63973	2
15610302		0	0	6.63E-10	56.95	3.33E-06	3	1455.7553	0.118066	21.65848	1
15610302		0	0	4.87E-10	51.88	1.46E-05	4	1897.9914	-0.01453	27.99529	2
15610302		0	0	2.33E-07	49.61	1.09E-05	2	1382.6283	0.934595	23.9521	0
15610302		0	0	6.12E-07	46.56	2.2E-05	2	927.48998	0.545017	15.43128	0
15610302		0	0	1.79E-06	46.38	2.65E-05	3	1968.917	0.078218	20.2068	1
15610302	M6(Oxidat	0	0	2.09E-07	43.99	3.98E-05	2	1398.6221	0.1084	15.10339	0
15610302	N-Term(A	0	0	7.51E-08	43.69	7.91E-05	3	2420.2097	0.141977	33.74866	0

15610302	C6(Carban	0	0	3.75E-07	41.01	0.000115	3	3784.8556	-3.1524	39.18079		1
15610302	M6(Oxidat	0	0	9.22E-06	40.89	8.13E-05	3	1984.9109	-0.43554	18.33968		1
15610302		0	0	1.61E-08	37.99	0.000469	4	2159.1239	0.028147	36.2116		2
15610302	N-Term(A	0	0	0.00128	32.03	0.000658	2	1467.6985	0.963159	42.95147		0
15610302		0	0	0.004685	29.02	0.002318	2	2099.0835	-0.25612	20.4332		2
15610302	N-Term(A	0	0	0.01458	26.73	0.003291	2	1497.7654	-0.16547	20.92226		1
15610302	M6(Oxidat	0	0	0.09697	21.49	0.007079	3	2000.9053	-0.66655	16.28562		1
15610302		0	0.002	0.1963	23.44	0.009737	2	1570.8185	0.055859	19.17891		1
15610302	C6(Carban	0	0.004	0.2644	21.19	0.008744	4	3800.8524	-2.62378	36.3913		1
15609134		0	0	0.03779	114.58	6.1E-12	3	3164.6294	-1.34096	37.35385		0
15609134		0	0	0.08844	67.77	2.67E-07	3	3660.8412	-1.47279	28.08707		2
15609134		0	0	0.06681	52.65	1.06E-05	3	2055.0333	-1.00874	25.27643		0
15609134		0	0	0.1993	39.43	0.000257	3	1264.6943	-0.00137	15.9043		1
15609134		0	0	0.1547	23.2	0.007658	4	3504.7462	0.229753	29.09296		1
15610079		0	0	0.01277	92.26	8.91E-10	2	1575.7609	-0.05042	25.12815		0
15610079		0	0	0.01866	84.17	6.89E-09	2	2549.2417	-0.11944	39.29253		0
15610079		0	0	0.02385	78.53	3.37E-08	2	1034.5451	-0.12542	33.68932		0
15610079		0	0	0.01199	75.57	5.27E-08	3	2992.4527	-0.73164	37.62249		1
15610079	M5(Oxidat	0	0	0.09326	69.03	2.94E-07	2	1050.5389	-1.20923	26.00053		0
15610079	M5(Oxidat	0	0	0.009734	62.04	1.25E-06	3	2607.284	0.262112	31.2696		1
15610079		0	0	0.009499	51.69	1.08E-05	5	4110.0514	1.400849	43.30474		0
15610079		0	0	0.1738	50.21	9.51E-06	2	996.54711	-0.21634	22.93133		0
15610079	N-Term(A	0	0	0.2245	45.51	6.89E-05	2	1092.556	4.809366	26.44424		0
15610079		0	0	0.0855	43.47	8.77E-05	2	2591.2874	-0.3744	33.7946		1
15610079	N-Term(A	0	0	0.1511	43.44	6.34E-05	3	2591.2538	0.46388	40.55036		0
15610079		0	0	0.08548	33.01	0.001275	3	2031.0468	0.106751	28.80106		1
15610079		0	0	0.2638	31.11	0.000773	2	1451.8385	3.848414	24.08354		1
15610879		0	0	1.1E-15	84.44	5.04E-09	2	1515.7374	-1.66191	29.56546		0
15610879		0	0	2.22E-08	77.07	3.73E-08	2	1288.711	0.173661	22.59835		0
15610879	M3(Oxidat	0	0	1.45E-05	70.02	2.34E-07	2	1160.6088	-0.41707	22.02155		0
15610879		0	0	1.4E-06	60.21	2.1E-06	2	1144.6137	-0.59954	27.55621		0
15610879		0	0	0.04119	50.22	9.48E-06	2	878.49834	0.122157	24.27544		0
15610879		0	0	3.61E-05	48.04	1.88E-05	3	1323.7741	-0.2567	28.42108		0
15610879	M3(Oxidat	0	0	1.29E-06	46.25	5.45E-05	3	2657.3298	-0.5717	27.60408		1
15610879		0	0	7.7E-10	45.69	7.01E-05	3	2740.3846	-1.54892	31.64378		0
15610879		0	0	0.000667	37.96	0.00032	2	986.57292	-1.30525	14.79117		1

15610879		0	0	0.004988	29.24	0.001727	3	1390.767	-1.37971	26.12857		1
15610879		0	0	0.01116	22.54	0.008636	3	1515.8482	-0.52254	21.41374		1
15609129		0	0	9.8E-06	80.41	9.08E-09	2	1495.8848	0.322166	36.16327		0
15609129		0	0	8.53E-05	64.65	4.11E-07	2	1292.7325	0.230654	42.45534		0
15609129		0	0	0.03894	64.05	1.06E-06	2	858.50505	0.770155	24.36599		0
15609129		0	0	4.84E-05	59.07	1.3E-06	2	1420.8268	-0.21922	19.6648		1
15609129		0	0	6.07E-05	54.34	3.67E-06	2	1822.0926	0.870206	39.40124		1
15609129	N-Term(A	0	0	0.000487	49.19	1.69E-05	2	1334.7437	0.721968	32.72831		0
15609129		0	0	0.004411	48.77	2.59E-05	3	2696.3617	2.060611	30.61564		0
15609129	N-Term(A	0	0	0.000155	41.78	6.62E-05	2	1537.8943	-0.36517	43.81342		0
15609129		0	0	0.04399	23.78	0.008166	3	1142.6633	-0.67175	21.11645		1
15610900		0	0	5.17E-16	109.71	2.51E-11	2	1771.9075	-0.62814	34.54695		0
15610900		0	0	5.17E-16	97.07	5.1E-10	2	1270.7006	0.313073	40.81439		0
15610900		0	0	3.17E-09	85.48	3.4E-09	4	3123.5465	1.54254	46.77856		0
15610900		0	0	5.17E-16	84.21	1.06E-08	3	1928.0092	-0.29143	24.65831		1
15610900	M11(Oxid	0	0	5.17E-16	84.02	5.15E-09	4	3139.5397	0.976977	41.70951		0
15610900	N-Term(A	0	0	5.82E-10	64.74	8.23E-07	2	1312.711	0.159059	39.49514		0
15610900		0	0	5.17E-16	63.5	7.82E-07	3	1754.9775	0.814737	32.6355		1
15610900	M11(Oxid	0	0	6.34E-09	61.04	8.66E-07	3	3896.9403	-1.18954	40.74921		1
15610900		0	0	3.71E-06	44.61	3.45E-05	2	1069.5168	-0.57042	13.09664		0
15610900		0	0	1.8E-08	42.69	7E-05	2	1032.5945	-0.38861	19.323		1
15610900		0	0	1.75E-06	39.46	0.000266	2	776.42589	-0.31093	13.53001		0
15610900		0	0	0.000484	39.13	0.000299	3	1389.7439	-0.43221	17.38546		1
15610900		0	0	0.000385	38.96	0.000165	4	3880.9628	3.295049	45.15834		1
15610900		0	0	0.000381	34.68	0.000579	2	1620.7983	-0.46022	16.03942		1
15610900		0	0	0.04747	34.03	0.000988	2	1116.6111	-0.82367	18.09402		1
15610900	M4(Oxidat	0	0	0.000273	27.68	0.002133	2	982.49034	-1.11278	21.6262		0
15610900	N-Term(A	0	0	0.01341	19.08	0.014831	3	3181.5611	4.381196	41.99681		0
15610959		0	0	2.06E-09	98.51	2.54E-10	2	1585.8779	-1.13255	33.90888		0
15610959		0	0	0.000623	69.86	1.19E-07	3	1772.0072	0.335194	35.65822		0
15610959		0	0	5.76E-15	66.38	5.18E-07	2	2103.0899	-0.24723	37.49992		1
15610959		0	0	4.65E-14	62.05	1.43E-06	2	1730.9155	0.457314	39.44442		0
15610959	M4(Oxidat	0	0	1.1E-05	59.76	2.32E-06	2	1746.9106	0.568867	36.83979		0
15610959		0	0	0.000688	59.18	1.45E-06	2	1193.6513	-1.02089	28.35012		1
15610959		0	0	8.49E-05	44.74	6.55E-05	3	1349.7524	-0.90148	30.2646		2
15610959	M2(Oxidat	0	0	0.009451	41.9	6.44E-05	2	840.40361	0.375715	15.13336		0

15610959		0	0	0.01722	41.21	0.000121	3	3403.779	-0.80071	36.30675		0
15610959	M7(Oxidat	0	0	0.001806	35.93	0.000472	3	2119.084	-0.64594	35.26683		1
15610959		0	0	0.000886	35.33	0.000292	2	824.40843	0.063717	19.13796		0
15610571		0	0	0.000133	113.28	8.69E-12	2	2197.1011	-1.1968	38.15102		0
15610571		0	0	0.01839	72.9	1.1E-07	3	3100.5868	0.501134	47.16103		1
15610571		0	0	0.02539	70.4	1.23E-07	3	3300.6174	1.81586	43.12237		0
15610571		0	0	0.02854	67.02	3.18E-07	2	1133.6318	0.344173	48.91167		0
15610571		0	0	0.06545	62.75	5.3E-07	2	922.49883	-0.51426	27.00033		0
15610571	N-Term(A	0	0	0.1634	43.21	9.79E-05	2	1175.6418	-0.14041	31.75353		0
15610571		0	0	0.2101	38.34	0.000249	2	1711.887	-0.30423	21.71603		1
15610571		0	0	0.2301	37.82	0.000487	3	1923.0159	-1.92302	25.51557		1
15610571	N-Term(A	0	0	0.3957	31.91	0.000805	2	964.50762	-2.33315	25.41962		0
15609356		0	0	0.005398	113.07	1.16E-11	2	1771.9086	-0.03068	30.40192		1
15609356		0	0	0.00987	98.68	2.57E-10	2	1577.8385	-0.61015	22.22124		0
15609356		0	0	2.78E-05	92.1	1.02E-09	2	1643.8147	0.634843	32.50073		0
15609356		0	0	8.52E-05	83.16	1.01E-08	3	1684.9493	-0.06372	30.97549		0
15609356	N-Term(A	0	0	0.000516	68.88	3.43E-07	2	1619.8521	1.248311	26.70443		0
15609356		0	0	0.004573	66.27	2.35E-07	2	855.53002	0.168972	22.03969		0
57117150		0	0	0.01111	110.87	1.72E-11	2	1940.0452	0.19191	31.23806		0
57117150		0	0	0.1159	77.41	4.08E-08	2	1105.6009	0.693177	28.60871		0
57117150		0	0	0.01503	61.84	1.15E-06	3	3006.5936	-1.08437	27.56721		1
57117150		0	0	0.466	43.06	9.89E-05	2	1085.5687	-1.11479	13.39184		0
57117150		0	0	0.01885	36.88	0.000318	4	3026.6271	-0.00509	32.947		1
57117150		0	0	0.4458	25.54	0.002786	3	1772.0182	0.180751	26.19673		0
15609762		0	0	0.008236	97.72	2.7E-10	2	2184.1963	-0.94068	30.10696		1
15609762		0	0	0.02921	97.61	2.08E-10	2	1219.6819	2.075184	33.68053		0
15609762		0	0	0.000102	95.1	4.64E-10	2	1941.0656	0.184464	48.80788		0
15609762		0	0	0.01074	95.1	5.25E-10	3	2667.303	1.680112	36.61731		0
15609762	M5(Oxidat	0	0	0.01073	95.09	3.72E-10	3	2683.287	-2.39439	44.38933		0
15609762		0	0	0.01291	87.12	4.17E-09	2	1101.6263	-0.03903	35.69721		0
15609762		0	0	0.09436	81.61	1.76E-08	2	2098.1055	0.68874	48.27692		1
15609762		0	0	0.0452	48.91	1.54E-05	3	3169.7259	1.332596	37.9067		0
15609762	N-Term(A	0	0	0.001776	48.34	2.05E-05	3	3211.727	-1.63227	39.00375		0
15609762	N-Term(A	0	0	0.01759	47.07	3.14E-05	3	2709.3094	0.119979	37.11931		0
15609762		0	0	0.09606	46.5	2.23E-05	2	1015.4947	-0.91068	14.97556		0
15609762		0	0	0.1108	36.9	0.000204	3	3325.8197	-0.94594	34.10666		1

15609762		0	0	0.1441	36.59	0.000439	3	1732.8752	-0.83392	21.03861	1
15609762		0	0	0.2775	33.52	0.000444	2	778.44579	-0.00508	41.99107	0
15609762		0	0	0.07796	32.66	0.000976	2	1041.5791	-0.83732	15.57067	1
15609762		0	0	0.03384	25.28	0.002958	4	3951.1364	-1.22953	33.91577	2
15609762		0	0	0.1619	21.38	0.015283	5	2815.4841	-0.33145	30.54496	2
15609762		0	0	0.2593	19.93	0.016768	3	1197.6801	-0.85408	16.13294	2
15609762	N-Term(A	0	0	0.1228	19.03	0.02563	2	2140.1106	-1.86579	30.78219	1
15609762	M5(Oxidat	0	0	0.05649	16.92	0.030485	4	3400.6786	1.224248	45.90045	1
15610873		0	0	6.54E-15	89.62	1.47E-09	2	1171.57	-0.21118	19.43807	0
15610873	N-Term(A	0	0	1.93E-11	69.43	1.31E-07	2	1213.581	0.143308	19.49857	0
15610873		0	0	1.25E-12	62.85	9.86E-07	2	1414.7033	-0.0683	18.2327	1
15610873		0	0	9.95E-07	62.05	1.06E-06	2	1212.707	0.820873	43.91353	1
15610873		0	0	6.37E-09	50.28	1.13E-05	2	980.56194	-1.74803	18.60782	1
15610873		0	0	0.000164	46.48	2.24E-05	2	957.60832	-0.90573	25.64158	1
15610873		0	0	1.63E-09	41.12	7.71E-05	2	824.46221	-0.3948	24.43098	0
15610873		0	0	0.002573	36.36	0.000474	4	1570.8028	-1.11804	15.60222	2
15610873		0	0	0.003263	36.18	0.000289	2	829.51366	-0.67854	28.2716	0
15610873		0	0	3.81E-07	29.99	0.001904	3	1425.6974	0.365118	23.51266	1
15610873		0	0	1.28E-06	18.58	0.017334	3	1193.6867	0.367232	19.87589	2
15610873	N-Term(A	0	0.003	0.05201	29.45	0.00193	2	1254.7182	1.323761	39.06954	1
15610873		0	0.003	0.06425	12.12	0.119684	4	1922.0093	0.10757	30.21887	2
15610930		0	0	4.9E-06	95.83	8.23E-10	2	859.46343	0.192219	17.19044	0
15610930		0	0	8.62E-11	87.26	3.95E-09	3	2060.0899	0.726977	40.48858	1
15610930		0	0	5.03E-07	86.68	4.51E-09	2	1219.6436	0.476357	49.65616	0
15610930		0	0	1.06E-06	65.74	2.66E-07	2	955.53148	-0.55529	16.3913	0
15610930	N-Term(A	0	0	5.81E-05	54.76	7.19E-06	2	901.47331	-0.56807	18.82328	0
15610930	N-Term(A	0	0	0.02366	26.62	0.005989	2	1261.6574	3.019783	30.88089	0
15609880	M23(Oxida	0	0	1.83E-05	92.57	7.19E-10	3	2524.2039	0.778572	40.89861	0
15609880	M9(Oxidat	0	0	0.001956	82.69	8.34E-09	2	1271.644	-1.08348	42.28862	0
15609880		0	0	0.00012	82.19	7.85E-09	2	2038.0965	-0.17573	40.01718	0
15609880		0	0	0.00013	76.78	3.04E-08	3	2508.2098	1.116607	49.71311	0
15609880		0	0	0.003529	72.29	6.79E-08	2	1255.6506	0.102731	46.24121	0
15609880		0	0	0.006683	70.55	1.81E-07	2	1280.6231	0.064948	20.88714	1
15609880	M13(Oxida	0	0	0.000375	69.09	1.36E-07	3	2540.1998	1.165624	31.83883	0
15609880	M3(Oxidat	0	0	0.00191	68.07	1.56E-07	3	3609.7145	-1.41165	38.82726	1
15609880	C4(Carban	0	0	0.01022	62.83	5.2E-07	2	949.45128	-0.79498	12.04974	0

15609880	M3(Oxidat	0	0	0.005706	60.77	1.05E-06	3	3593.7195	-1.44022	47.71433	1
15609880	M33(Oxid	0	0	0.001319	60.48	1.07E-06	3	3577.7248	-1.3667	49.2673	1
15609880	C4(Carban	0	0	0.0031	50.28	2.3E-05	3	2968.5315	0.145512	37.8923	1
15609880		0	0	0.003958	48.47	3.34E-05	3	1936.0087	0.862519	26.93205	0
15609880	M12(Oxid	0	0	8.47E-05	48.16	3.97E-05	3	1952.0022	0.146064	21.68477	0
15609880	M13(Oxid	0	0	0.03234	40.76	0.000176	4	2108.1029	-0.06656	20.82616	1
15609880	N-Term(A	0	0	0.00991	40.12	0.000107	2	1313.6557	-0.17056	45.42235	0
15609880	M3(Oxidat	0	0	0.01734	37.44	0.000261	2	1088.5403	-0.19797	21.93682	0
15609880	N-Term(A	0	0	0.0146	35.26	0.000313	3	2791.3061	-0.16726	37.53306	0
15609880		0	0	0.02126	33.73	0.000868	2	1072.5452	-0.38943	24.37908	0
15609880		0	0	0.06312	32.6	0.001182	4	2092.1076	-0.28041	23.84085	1
15609880		0	0	0.0291	26.63	0.00239	3	1252.7159	-0.1479	25.5267	1
15609880		0	0.003	0.2068	33.77	0.000419	2	797.45226	0.797253	19.87143	0
15609880	N-Term(A	0	0.003	0.1223	30.46	0.001394	3	1322.6366	2.286353	19.30869	1
15609880		0	0.003	0.1322	21.1	0.010091	3	1463.8449	0.465545	18.45882	1
15609880	N-Term(A	0	0.003	0.1671	18.81	0.017098	3	2080.107	-0.18149	46.81402	0
15610137		0	0	0.008443	88.95	1.27E-09	2	2063.1807	-2.57837	40.43659	0
15610137		0	0	0.02473	81.13	1.19E-08	2	1147.6462	2.739924	21.0262	1
15610137		0	0	0.033	73.23	4.74E-08	2	1923.0893	-3.01614	47.09251	0
15610137		0	0	0.02784	71.16	7.64E-08	2	2219.2864	-0.32747	40.96831	1
15610137	N-Term(A	0	0	0.02581	61.54	1.96E-06	2	1385.7752	0.30148	31.24352	1
15610137		0	0	0.06871	61.23	9.04E-07	2	1343.7632	-0.72938	22.78085	1
15610137		0	0	0.03998	54.12	3.86E-06	3	2375.3877	-0.2538	38.31064	2
15610137	N-Term(A	0	0	0.0917	51.58	6.93E-06	3	2261.2965	-0.54588	44.8339	1
15610137	N-Term(A	0	0	0.1379	47.61	2.51E-05	2	1214.6324	-2.25128	39.82718	0
15610137		0	0	0.2556	47.14	4.15E-05	2	801.48241	-0.63989	25.02012	0
15610137		0	0	0.1579	46.48	2.7E-05	2	1095.59	-0.53659	17.60488	0
15610137		0	0	0.2228	43.33	4.88E-05	2	997.52178	0.345671	19.50407	0
15610137	C2(Carban	0	0	0.2282	42.57	0.000108	2	904.46672	-0.2575	17.92893	0
15610137		0	0	0.03207	41.24	7.5E-05	3	2734.5393	0.091238	45.56955	1
15610137		0	0	0.1502	37.86	0.00018	4	1878.0552	-0.25815	28.84783	1
15610137	N-Term(A	0	0	0.03836	35.63	0.000274	3	2776.5465	-1.14329	45.42308	1
15610137		0	0	0.2349	34.25	0.00077	2	1041.5837	-0.36054	26.06385	0
15610137		0	0	0.3253	34.06	0.00053	2	1069.5525	-1.1813	19.67626	0
15610137		0	0	0.2366	33.72	0.001146	2	1129.6464	0.034173	27.8288	0
15610137		0	0	0.3395	27.76	0.003852	2	1424.7742	-1.09054	20.69755	1

15610137		0	0	0.286	27.74	0.004375	3	1853.9608	-0.52469	28.03486	1
15610137		0	0.006	0.3422	35.02	0.000881	2	803.42504	-0.9533	17.07463	0
15610831		0	0	0.4607	61.25	7.48E-07	2	980.55205	-0.36892	35.73096	0
15610831		0	0	0.1461	59.89	3.28E-06	2	800.46196	-0.71164	17.59442	0
15610831		0	0	0.2818	57.61	3.64E-06	3	2195.1656	-0.01358	29.76495	0
15610831	M7(Oxidat	0	0	0.1874	56.9	3.27E-06	4	3303.7208	0.36916	45.37411	0
15610831		0	0	0.2807	52.24	5.96E-06	3	2202.3025	0.654793	43.75685	0
15610831	M16(Oxida	0	0	0.2716	47.73	3.88E-05	3	2211.1589	-0.72253	27.7918	0
15610831		0	0	0.2622	41.54	0.000144	2	1758.8943	-0.74853	26.22189	1
15610831		0	0	0.2335	31.22	0.001397	4	3287.7248	0.012425	47.65809	0
15610831	M12(Oxida	0	0	0.3928	19.19	0.027113	3	1774.8889	-0.94494	23.28292	1
15610928		0	0	5.57E-16	98.03	1.57E-10	2	1574.8956	0.872509	48.93375	0
15610928		0	0	5.57E-16	94.69	6.28E-10	3	2339.1452	2.127054	43.79765	0
15610928		0	0	5.57E-16	68.62	1.37E-07	3	4072.9804	0.176914	46.19836	0
15610928		0	0	1.96E-14	66.92	3.96E-07	2	1513.778	1.44037	21.7499	0
15610928		0	0	4.6E-12	57.61	4.68E-06	2	890.47252	-0.64073	24.45936	0
15610928		0	0	9.11E-10	52.81	6.28E-06	2	1155.5662	0.699578	17.67601	0
15610928	N-Term(A	0	0	2.82E-09	52.43	1.34E-05	2	932.48302	-0.68367	31.97392	0
15610928		0	0	1.37E-08	49.85	1.66E-05	2	1152.6006	-3.63332	29.61199	0
15610928		0	0	5.84E-07	48.12	2.7E-05	4	2232.1503	-0.80731	24.86922	2
15610928		0	0	9.81E-08	44.69	6.45E-05	3	1669.8741	-1.7269	23.88847	1
15610928		0	0	1.61E-07	43.74	5.28E-05	3	2446.3482	-0.51204	47.02425	1
15610928		0	0	0.002452	41.7	0.000152	4	2060.1049	0.586283	25.789	2
15610928		0	0	1.42E-08	41.19	7.59E-05	2	766.40929	-0.14741	23.36889	0
15610928		0	0	6.58E-09	41.02	0.000245	2	1484.8101	1.195367	40.20002	0
15610928		0	0	6.54E-07	40.2	0.000215	3	2650.322	-0.52322	24.93265	1
15610928		0	0	6.13E-06	37.42	0.000362	3	1637.8648	0.115993	33.54906	1
15610928		0	0	3.95E-05	32.1	0.000678	2	922.50768	-3.08323	19.69245	1
15610928		0	0	0.005801	31.92	0.000803	4	1793.9663	0.311042	31.71203	2
15610928	N-Term(A	0	0	7.74E-05	28.07	0.002651	2	1555.7858	-0.36772	24.3969	0
15610928	M11(Oxida	0	0	0.004672	22.31	0.0141	2	1500.8023	-0.63473	37.05159	0
15610928		0	0	3.75E-07	21.04	0.009051	4	4229.0895	2.047	44.79568	1
15610928		0	0	0.09076	17.58	0.027933	2	1308.7059	-0.06354	25.78258	1
15610241		0	0	5.45E-16	114.57	9.25E-12	2	2223.1307	-0.90794	41.57571	0
15610241	M16(Oxida	0	0	5.45E-16	100.95	1.29E-10	2	2239.1299	1.042419	38.98692	0
15610241		0	0	5.45E-16	84.35	4.41E-09	2	1509.8283	0.710921	31.61615	0

15610241		0	0	4.04E-12	64.91	4.52E-07	2	1045.5888	-0.05603	35.40572	0
15610241	M12(Oxid	0	0	5.45E-16	62.9	8.21E-07	3	3070.4648	-0.88242	40.3412	0
15610241	C9(Carban	0	0	2.39E-11	55.82	2.88E-06	2	1620.7554	0.396971	15.88055	0
15610241		0	0	2.71E-09	51.67	1.94E-05	2	1203.6507	2.136055	23.47692	1
15610241		0	0	7.73E-07	33.99	0.001097	2	1676.8479	-0.66989	29.37567	0
15610929	C17(Carba	0	0	0.009851	87.66	3E-09	2	2050.1111	-0.16278	39.22273	0
15610929		0	0	0.03004	80.08	2.99E-08	2	1113.6265	0.180619	29.84539	0
15610929		0	0	0.04439	77.68	3.92E-08	2	2229.1785	0.409611	30.85328	0
15610929		0	0	0.08564	66.7	4.17E-07	3	2571.3437	0.338649	28.9796	1
15610929		0	0	0.1467	59.28	3.01E-06	2	1031.5265	-0.363	21.70485	0
15610929		0	0	0.2208	41.49	7.08E-05	2	957.59685	-1.15074	25.36763	0
15610929		0	0	0.1448	35.63	0.000438	3	3424.6915	-7.551	42.22481	0
15610929		0	0	0.321	28.78	0.002516	3	1140.6854	0.523492	23.4636	1
57116830	M5(Oxidat	0	0	1.9E-11	97.7	1.69E-10	2	1541.6373	-0.80542	17.93241	0
57116830		0	0	2.8E-06	88.27	1.49E-09	2	1525.6425	-0.70636	22.14833	0
57116830		0	0	1.23E-05	85.95	2.53E-09	2	1653.7342	-2.64422	21.6244	1
57116830		0	0	5.31E-05	71.43	7.18E-08	2	1714.801	-1.29483	23.87684	0
57116830	N-Term(A	0	0	1.74E-06	61.31	7.38E-07	2	1695.7612	7.099878	24.177	1
57116830		0	0	8.68E-09	52.67	5.39E-06	3	2383.0918	-1.33452	22.32297	1
57116830		0	0	2.16E-08	47.9	3.08E-05	3	2180.1416	-0.76446	16.62692	1
57116830		0	0	2.15E-08	44.47	3.57E-05	3	1809.838	-0.89757	20.03939	2
57116830	M6(Oxidat	0	0	8.49E-05	43.61	4.34E-05	3	1825.8338	-0.44468	15.89843	2
57116830		0	0	1.62E-07	43.51	4.45E-05	2	1182.5423	-0.26838	18.70106	0
57116830		0	0	5.12E-05	40.85	8.2E-05	2	1265.5972	-0.76306	11.70636	2
57116830		0	0	2.77E-10	39.78	0.000274	3	2024.0401	-0.98172	19.95935	0
57116830		0	0	0.000284	38.31	0.000325	2	1873.9251	-0.71768	17.99124	1
57116830		0	0	1.76E-08	35.86	0.000584	3	2117.0584	-0.54155	14.50927	2
57116830	M5(Oxidat	0	0	0.005437	35.29	0.000295	2	1697.7371	-1.47735	16.58767	1
57116830		0	0	0.000248	33.84	0.000412	4	2831.2992	-1.00175	23.06437	2
57116830		0	0	0.000767	32.03	0.000625	3	2163.0119	0.769146	21.22246	1
57116830		0	0	0.001964	27.97	0.004149	4	2030.0253	-1.11922	14.33868	2
57116830	N-Term(A	0	0.003	0.008904	39.19	0.00012	2	1756.81	-2.13579	22.37185	0
57116830		0	0.003	0.01588	28.65	0.002729	3	1369.7184	-0.90045	12.16063	0
57116830		0	0.003	0.01108	26.97	0.002004	2	1109.4961	-0.82916	12.47107	1
57116830	N-Term(A	0	0.003	0.008716	25.51	0.006186	4	2066.0468	-2.86183	18.30732	0
57116830		0	0.003	0.01461	15.26	0.038721	2	1338.6432	-0.36247	17.60265	1

57116830		0	0.003	0.008716	13.47	0.110196	3	1525.8191	-1.07834	12.87114		1
57116830		0	0.003	0.009464	11.21	0.075504	3	1960.9235	2.378862	22.65614		1
15609332		0	0	1.74E-10	80.01	1.4E-08	2	1213.621	-0.14174	29.86408		0
15609332		0	0	2.94E-08	71.15	1.76E-07	2	1401.7651	0.892409	29.88242		1
15609332	C2(Carban	0	0	4.08E-07	69.52	1.11E-07	2	2170.9595	-2.29348	24.03299		0
15609332		0	0	5.15E-05	66.88	3.9E-07	2	1232.6115	-3.49949	24.75378		0
15609332		0	0	0.00086	63.98	5.6E-07	2	1883.8974	0.544173	39.96939		1
15609332	M20(Oxid	0	0	0.000315	63.96	4.01E-07	3	2526.1847	-1.70208	23.99902		1
15609332		0	0	0.008098	60.55	1.85E-06	2	1030.5722	0.727609	26.67953		0
15609332	M6(Oxidat	0	0	3.57E-06	57.08	5.29E-06	2	1046.5657	-0.60663	22.1103		0
15609332		0	0	8.66E-06	56.59	5.59E-06	2	1273.6696	0.598242	33.94068		0
15609332		0	0	0.05941	54.61	1.73E-05	2	842.49187	0.17518	27.66107		0
15609332	N-Term(A	0	0	0.04072	52.58	6.35E-06	2	1255.6296	-1.74582	30.64154		0
15609332		0	0	7.52E-07	52.53	7.26E-06	2	1854.0439	-0.78232	35.482		1
15609332	M6(Oxidat	0	0	2.76E-05	51.93	6.4E-06	3	2156.9187	-0.59205	30.41352		0
15609332	M13(Oxid	0	0	8.07E-05	49.21	1.92E-05	3	1870.0396	-0.31568	33.9972		1
15609332	M4(Oxidat	0	0	0.002064	47.16	7.98E-05	2	858.48631	-0.37463	24.24992		0
15609332	M6(Oxidat	0	0	6.91E-05	45.94	3.44E-05	3	1886.0354	0.117817	31.30137		1
15609332	M11(Oxid	0	0	0.000377	44.96	3.18E-05	3	2140.9316	3.043725	32.23365		0
15609332	M1(Oxidat	0	0	0.02462	41.9	6.44E-05	3	2172.9133	-0.71936	29.74228		0
15609332		0	0	0.001525	38.95	0.000299	2	885.51598	0.775225	19.43385		0
15609332	C3(Carban	0	0	2.13E-05	33.4	0.000823	3	2881.4361	-1.01685	32.93391		0
15610006		0	0	2.82E-12	113.89	4.29E-12	2	1751.8271	0.052897	34.28084		0
15610006		0	0	3.24E-14	109.98	1.61E-11	2	1767.8752	-0.47041	31.58391		0
15610006	C8(Carban	0	0	6.86E-15	109	2.33E-11	2	1732.8528	-0.39501	25.20614		0
15610006	M12(Oxid	0	0	2.45E-10	92.88	5.14E-10	2	1767.8218	-0.04036	27.16114		0
15610006	C4(Carban	0	0	6.81E-08	91.13	1.35E-09	2	1610.8334	0.885456	28.02269		0
15610006	C8(Carban	0	0	3.5E-08	84.38	6.75E-09	3	3324.661	-2.01086	32.60793		1
15610006		0	0	0.001939	53.52	9.56E-06	2	2319.2254	2.090394	31.74442		0
15610006		0	0	0.000462	52.83	7.82E-06	2	1179.6469	-1.01989	20.78693		0
15610006	N-Term(A	0	0	0.001471	48.43	1.65E-05	2	2478.1958	-9.02992	33.75397		1
15610006		0	0	0.05569	36.55	0.000354	2	1307.7431	0.013987	19.26304		1
15610040	C11(Carba	0	0	1.02E-09	84.2	9.12E-09	2	1287.6834	-0.34798	23.76546		0
15610040		0	0	2.68E-09	72.23	1.8E-07	2	1418.7416	-0.79405	44.2928		0
15610040		0	0	0.000662	58.88	1.29E-06	2	1730.8019	-0.51064	20.22526		1
15610040		0	0	1.62E-05	57.64	2.15E-06	2	1124.6138	0.467565	27.11121		1

15610040	M2(Oxidat	0	0	0.000114	48.44	2.15E-05	2	1140.6095	1.173381	25.26648	1
15610040	M4(Oxidat	0	0	0.004043	47.66	1.71E-05	2	992.46196	0.075934	29.20953	0
15610040		0	0	0.001203	47.62	2.59E-05	2	1104.5469	-0.35191	25.01796	0
15610040		0	0	0.001598	47.44	3.79E-05	3	2544.3257	0.415217	40.75847	1
15610040	C12(Carba	0	0	2.79E-07	45.62	6.31E-05	2	1443.7845	-0.34207	19.30482	1
15610040		0	0	9.58E-05	39.55	0.000227	2	976.4677	0.745202	24.12483	0
15610040		0	0	0.001957	37.23	0.000189	2	967.57225	-0.16444	32.86839	0
15610040	M4(Oxidat	0	0	0.1061	36.82	0.000207	2	1746.7979	0.098959	16.94697	1
15610040		0	0	0.04103	30.92	0.000807	2	782.4262	-0.76204	11.26492	0
15610040		0	0	1.87E-06	29.78	0.00263	3	2504.2729	0.255564	35.64125	1
15610040	C3(Carban	0	0	0.004295	28.94	0.003	3	2544.2972	0.838736	34.51551	0
15610040		0	0	0.001794	19.34	0.015134	3	1564.9109	-0.19139	35.2556	1
15610040		0	0	0.1061	14.99	0.06973	3	2388.2254	0.768206	42.72978	0
15610375		0	0	0.010213	125.24	5.09E-13	2	2006.0656	-0.52937	43.494	0
15610375		0	0	0.03043	109.64	2.55E-11	2	1691.9073	-0.18744	32.25672	0
15610375		0	0	0.1437	97.3	4.66E-10	2	1306.7006	0.304448	40.65109	0
15610375		0	0	0.03415	89.61	1.15E-09	3	3274.7593	1.471608	40.96942	0
15610375	N-Term(A	0	0	0.1909	85.13	7.37E-09	2	1348.7094	-1.0218	47.55081	0
15610375		0	0	0.1234	78.87	1.88E-08	2	1415.7065	-0.14325	31.15428	0
15610375		0	0	0.215	68.84	1.7E-07	2	2115.1714	0.814599	42.51916	1
15610375		0	0	0.1606	66.45	3.4E-07	2	1462.8018	0.324112	35.63984	1
15610375	N-Term(A	0	0	0.1677	64.75	3.68E-07	3	3316.7611	-1.1803	44.79927	0
15610375	C7(Carban	0	0	0.1683	62.65	1.52E-06	2	1131.6198	0.603803	28.82511	0
15610375	M7(Oxidat	0	0	0.1612	56.91	3.77E-06	2	1411.7178	-0.50666	28.74752	0
15610375		0	0	0.1925	52.48	1.16E-05	2	1395.722	-1.18209	34.35594	0
15610375	M15(Oxida	0	0	0.1315	51.79	9.6E-06	3	1686.8452	-1.69449	25.15408	1
15610375	C1(Carban	0	0	0.1977	48.83	3.34E-05	2	1032.5505	-0.22533	33.66865	0
15610375		0	0	0.1984	46.27	7.08E-05	2	1058.5663	-0.02789	27.96778	0
15610375	C7(Carban	0	0	0.1654	39.39	0.000121	3	2586.4023	6.209603	38.51479	1
15610375		0	0	0.09956	22.98	0.00579	5	3687.9579	0.312287	39.97365	1
15609331		0	0	4.73E-08	76.26	6.51E-08	2	1258.6386	-0.05077	23.99629	0
15609331	M1(Oxidat	0	0	0.000684	29.97	0.001005	3	1346.6287	-1.28805	12.59819	1
15610336		0	0	2.6E-13	125.51	5.2E-13	2	1563.7604	-0.35666	27.20758	0
15610336		0	0	2.02E-09	104.74	1.07E-10	2	1274.6965	1.049216	46.072	0
15610336		0	0	1.49E-09	96.67	2.69E-10	2	1442.7118	-0.28537	24.50989	0
15610336		0	0	3.17E-15	82.49	5.62E-09	2	1257.6069	-0.14486	19.04777	0

15610336		0	0	8.2E-09	81.92	1.12E-08	2	1381.7426	-0.63687	20.60297	0
15610336		0	0	5.53E-07	76.45	4.98E-08	2	1611.8833	1.232769	42.01703	0
15610336		0	0	1.28E-11	75.75	3.33E-08	3	2867.5572	-0.49422	33.23729	1
15610336		0	0	9.49E-06	74.17	7.27E-08	2	1443.8067	-0.02291	18.76763	1
15610336		0	0	5.34E-07	65.96	6.08E-07	2	2871.4588	-2.42506	40.66251	0
15610336	M6(Oxidat	0	0	6.05E-16	64.58	7.49E-07	3	2887.4608	0.042337	38.13357	0
15610336		0	0	1.93E-05	63.77	6.09E-07	2	870.54106	0.323849	28.89887	0
15610336		0	0	0.000539	57.85	1.64E-06	3	1276.7851	0.146487	29.85674	1
15610336		0	0	1.99E-08	55.91	2.56E-06	4	3109.6752	1.582257	46.98248	0
15610336		0	0	0.05267	54.71	3.37E-06	2	1552.9158	-0.78705	33.33192	1
15610336		0	0	1.22E-05	53.27	8.71E-06	2	1109.5705	0.577583	16.30621	0
15610336		0	0	1.36E-05	50.02	3.78E-05	3	1952.0519	-2.16096	23.83778	1
15610336		0	0	0.1194	39.02	0.000125	4	1959.1627	0.816233	33.46407	2
15610336		0	0	0.000608	26.26	0.005323	3	1188.6361	-0.92525	19.49997	0
15610336		0	0	1.34E-08	26.17	0.004106	3	2101.1317	1.354851	34.39058	1
15610941		0	0	3.15E-14	94.14	8.29E-10	2	1846.0179	-0.18496	33.92394	0
15610941		0	0	4.1E-12	86.88	2.05E-09	2	2478.1846	0.349696	28.65899	0
15610941		0	0	3.43E-05	82.66	5.69E-09	2	1138.5657	-7.20193	48.08397	0
15610941		0	0	9.1E-06	62.73	1.28E-06	2	1612.8128	0.576905	32.80647	0
15610941		0	0	1.91E-08	55.62	6.17E-06	3	2634.2823	-0.96783	26.48978	1
15610941		0	0	0.000109	53.85	8.65E-06	2	1756.9084	1.457955	43.56753	0
15610941		0	0	0.01692	50.93	8.05E-06	2	807.48393	0.437182	18.54036	0
15610941	C23(Carba	0	0	0.2956	37.07	0.000295	3	3119.5074	0.940567	42.95656	0
15610941	N-Term(A	0	0	0.000375	34.22	0.000511	2	1180.5835	-0.79843	25.0261	0
15610941		0	0	0.2956	34.2	0.000456	2	1294.6742	-0.61743	21.31592	1
15610941		0	0	0.001264	24.73	0.004711	2	1074.6161	-0.55353	17.71949	2
15610501		0	0	9.81E-14	115.52	7.29E-12	2	1187.6274	0.552054	24.27328	0
15610501		0	0	6.63E-08	95.87	3.75E-10	2	1192.5983	-0.67676	20.73475	0
15610501		0	0	2.53E-06	95.66	6.66E-10	2	2733.4407	-0.74627	39.55345	0
15610501	M5(Oxidat	0	0	3.87E-11	94.86	5.23E-10	2	1208.5911	-2.41955	21.11728	0
15610501	M3(Oxidat	0	0	9.13E-09	94.15	5.96E-10	3	2784.3506	2.669847	37.63561	0
15610501		0	0	3.64E-10	92.94	1.17E-09	2	1733.882	0.907966	37.01131	0
15610501	M1(Oxidat	0	0	6.41E-07	86.78	5.04E-09	2	2749.441	1.19634	37.44695	0
15610501		0	0	4.22E-10	86.15	4.85E-09	2	1406.7155	1.699278	43.08178	0
15610501		0	0	9.98E-08	83.62	7.6E-09	3	2386.3138	1.996198	48.95606	1
15610501		0	0	2.61E-07	82.93	5.08E-09	2	2115.1814	0.247158	47.81458	0

15610501	M1(Oxidat	0	0	8.21E-13	81.89	1.71E-08	3	2765.4333	0.242418	35.99109		0
15610501		0	0	6.27E-07	73.94	7.67E-08	2	943.55675	-0.43669	25.83556		0
15610501	M3(Oxidat	0	0	9.81E-06	71.43	7.91E-08	2	2800.3335	-1.62752	33.44902		0
15610501	M5(Oxidat	0	0	5.58E-08	70.34	1.71E-07	2	1422.7082	0.106263	24.27508		0
15610501		0	0	3.62E-09	69.04	1.24E-07	4	3213.7243	0.467548	43.68461		2
15610501		0	0	0.000019	67.07	2.65E-07	3	2942.5971	0.832525	43.68234		1
15610501		0	0	4.26E-10	66.09	5.78E-07	3	1715.9029	-0.36953	21.76948		1
15610501		0	0	6.69E-05	65.39	5.06E-07	2	1069.565	1.194602	25.5331		0
15610501	M3(Oxidat	0	0	4.06E-05	62.01	9.44E-07	2	1438.7035	0.415311	22.79875		0
15610501		0	0	1.31E-05	51.95	1.05E-05	3	2768.3492	0.341352	39.40819		0
15610501		0	0	0.003698	44.92	8.54E-05	2	846.43157	-0.05334	14.21751		0
15610501		0	0	0.0311	41.76	0.00018	2	1117.5766	-1.53512	19.5564		0
15610501	M3(Oxidat	0	0	0.02495	39.95	0.000233	2	989.53465	1.00879	21.8631		0
15610501		0	0	0.04546	39.25	0.000119	2	937.51036	0.193321	18.36344		1
15610501		0	0	0.04927	37.34	0.000498	3	1214.6843	-0.76729	23.19799		1
15610501		0	0	0.001159	30.63	0.001989	3	1342.7548	-0.20624	19.9881		1
15610501	M6(Oxidat	0	0.006	0.1343	35.02	0.000598	2	1133.5737	0.387886	14.99365		0
15610781		0	0	0.02324	87.81	2.98E-09	2	2366.2371	0.814308	42.2069		0
15610781		0	0	0.1342	72.16	1E-07	2	1680.8221	-0.46538	32.88571		0
15610781		0	0	0.1516	68.18	3.57E-07	3	2694.3655	0.686742	36.88042		0
15610781		0	0	0.1273	61.45	7.14E-07	2	1064.5959	-0.1301	15.91454		0
15610781		0	0	0.07913	61.07	1.13E-06	2	872.52007	0.029604	20.2927		0
15610781		0	0	0.09947	60.88	2.04E-06	2	988.5211	0.003467	26.96886		0
15610781	M7(Oxidat	0	0	0.06837	59.95	1.01E-06	2	3059.4014	0.883665	35.39485		0
15610781		0	0	0.07053	55.57	2.77E-06	3	3027.4079	-0.31354	40.63139		0
15610781	M7(Oxidat	0	0	0.09558	51.77	6.64E-06	4	3043.4135	3.179507	39.32915		0
15610781		0	0	0.2647	48.03	2.44E-05	2	946.45781	-1.15169	13.92884		0
15610781		0	0	0.1254	40.92	0.000129	2	908.44786	0.661832	19.04604		0
15610781		0	0	0.1819	35.52	0.000337	2	876.50493	-0.14933	27.97806		1
15610781	N-Term(A	0	0	0.281	30.45	0.000899	3	3101.414	1.534564	37.60351		0
15610879		0	0	0.003568	121.23	1.7E-12	2	1501.8223	0.127794	30.59072		0
15610879		0	0	0.000479	97.93	3.7E-10	3	2547.3376	1.051412	35.17208		0
15610879	C13(Carba	0	0	0.01344	89.95	1.82E-09	2	1541.7521	-0.56592	26.31711		0
15610879		0	0	0.03536	80.25	1.94E-08	2	1490.7675	2.436566	34.32092		0
15610879		0	0	0.008386	79.71	2.14E-08	2	1908.021	-0.83207	26.39442		0
15610879	M23(Oxid	0	0	0.02039	79.29	2.41E-08	2	2563.3221	-3.03794	31.80519		0

15610879		0	0	0.02319	76.09	6.15E-08	2	1170.6482	0.392653	40.4301	0
15610879		0	0	0.05904	68.96	3.37E-07	2	800.43669	-0.84728	12.28591	0
15610879		0	0	0.0261	67.29	3.55E-07	2	1684.8943	-2.03965	27.16695	1
15610879		0	0	0.08089	66.39	5.74E-07	2	1164.6011	0.258043	31.114	1
15610879		0	0	0.01371	65.13	3.99E-07	2	1222.6788	-0.2558	22.49376	1
15610879		0	0	0.006328	63.13	5.84E-07	2	1324.758	-0.32393	24.71959	1
15610879	C15(Carba	0	0	0.03097	62.44	1.34E-06	3	1810.937	-0.62919	28.96333	1
15610879		0	0	0.01294	57.68	3.84E-06	2	1454.8324	-0.12801	22.76918	0
15610879		0	0	0.1454	55.58	3.04E-06	2	1008.499	-0.68547	28.09018	0
15610879		0	0	0.0342	43.39	4.57E-05	2	828.44585	-0.43807	21.54671	0
15610879		0	0	0.08203	39.36	0.000145	2	1296.7494	-0.10338	19.23737	1
15610879		0	0	0.04708	31.21	0.001552	3	2421.2884	-0.09791	27.73636	1
15610879	N-Term(A	0	0.003	0.2006	27.09	0.003909	2	1366.7764	5.442358	27.50338	1
15610879		0	0.003	0.1993	22.48	0.014406	4	2897.5001	-1.50078	38.62685	1
57117003		0	0	0.00573	116.82	5.72E-12	2	1284.728	0.655059	34.68195	0
57117003		0	0	0.007952	98.59	2.84E-10	2	1404.7156	-0.14774	44.6943	0
57117003		0	0	0.01215	88.63	2.4E-09	2	1627.7978	0.918905	27.35389	0
57117003	C10(Carba	0	0	0.0415	84.58	4.35E-09	2	1442.7109	0.795536	32.5238	0
57117003		0	0	0.00536	83.54	5.75E-09	2	1667.9135	1.114235	38.35056	0
57117003		0	0	0.01666	77.55	1.75E-08	2	1157.5562	1.38105	49.32187	0
57117003		0	0	0.02611	74.33	6.46E-08	2	1540.7961	1.250527	36.26572	0
57117003		0	0	0.06207	73.57	1.32E-07	2	1302.6803	0.179745	21.85776	0
57117003	M8(Oxidat	0	0	0.00338	72.6	1.02E-07	2	2493.2554	-1.68359	39.49729	0
57117003		0	0	0.002185	72.56	8.32E-08	2	1791.0018	0.326791	46.60152	0
57117003	N-Term(A	0	0	0.025	71.31	1.96E-07	2	1326.7393	1.227906	35.40052	0
57117003	M9(Oxidat	0	0	0.09108	68.46	2.49E-07	2	1556.7886	-0.27909	32.20007	0
57117003		0	0	0.0127	67.23	2.46E-07	2	1952.9377	-0.32855	29.87121	0
57117003		0	0	0.002203	66.53	5.22E-07	3	2262.19	0.97927	44.24698	0
57117003		0	0	0.003778	62.71	1.15E-06	2	2477.2657	0.392085	39.39908	0
57117003		0	0	0.03792	62.16	1.28E-06	2	1628.8339	-0.76214	21.79428	1
57117003		0	0	0.09031	56.08	3.45E-06	2	1450.7685	-0.31093	32.24879	0
57117003		0	0	0.03763	55.79	2.63E-06	2	2191.0286	-0.75729	29.13092	1
57117003		0	0	0.008892	54.33	7.38E-06	4	2618.3976	1.436915	37.87658	1
57117003		0	0	0.01278	54.28	3.72E-06	3	2828.5845	-1.29552	42.47546	1
57117003	M16(Oxid	0	0	0.08083	53.98	5.4E-06	2	1968.9274	-2.95112	26.40765	0
57117003		0	0	0.009088	53.78	1.05E-05	3	2719.3366	-1.85275	38.92183	0

57117003		0	0	0.08305	49.2	2.71E-05	2	1458.7792	-1.37711	19.73507	1
57117003		0	0	0.2217	47.77	1.67E-05	2	969.50005	0.006497	20.1865	0
57117003	N-Term(A	0	0.002	0.2442	30.73	0.001014	2	1833.0163	2.48042	30.66384	0
15610564	M18(Oxid	0	0	4.38E-05	104.18	8.4E-11	2	2100.0508	-1.6797	31.86138	0
15610564		0	0	0.000215	103.5	8.71E-11	2	1863.9236	0.010882	37.12489	0
15610564		0	0	0.000896	89.22	1.97E-09	2	2084.0591	-0.14955	36.26905	0
15610564		0	0	0.000504	88.25	2.99E-09	2	2069.0762	-0.63402	33.01388	0
15610564	M1(Oxidat	0	0	0.001095	77.02	3.87E-08	2	1828.9635	0.471456	33.82723	0
15610564	M8(Oxidat	0	0	0.004925	76.57	2.31E-08	3	2554.2204	-1.56025	40.79965	0
15610564	M10(Oxid	0	0	0.00575	70.07	1.77E-07	3	2767.3471	0.048332	36.2522	1
15610564		0	0	0.01187	67.58	2.71E-07	2	900.52611	-0.09071	26.47235	0
15610564		0	0	0.01202	66.66	4.1E-07	2	1730.8664	-2.5887	24.57328	0
15610564		0	0	0.006639	63.11	1.1E-06	2	2061.0657	0.242655	37.07186	0
15610564	N-Term(A	0	0	0.01117	62.19	1.18E-06	2	1905.9338	-0.21663	37.23555	0
15610564		0	0	0.005678	59.21	2.52E-06	2	1812.9681	0.162097	37.11984	0
15610564		0	0	0.01985	59.04	1.24E-06	2	954.53636	-0.41673	20.84433	0
15610564		0	0	0.06345	56.86	5.56E-06	2	975.5114	-0.53425	14.36929	0
15610564		0	0	0.03486	55.6	2.75E-06	2	1307.59	-0.21251	20.38223	0
15610564		0	0	0.01631	53.09	1.08E-05	3	1150.6021	-1.42997	18.94985	0
15610564		0	0	0.04485	48.65	1.91E-05	2	1106.5477	-0.05086	23.4445	0
15610564		0	0	0.01074	42.9	6.41E-05	3	2538.23	0.201828	45.42094	0
15610564	M5(Oxidat	0	0	0.04412	41.23	0.000136	3	1166.5987	-0.00756	17.21787	0
15610564		0	0	0.1699	36.32	0.000455	2	991.53594	-0.07567	30.24325	0
15610564	N-Term(A	0	0	0.09101	35.59	0.000621	2	942.53551	-1.32332	29.91296	0
15610564		0	0	0.109	34.75	0.00062	2	1386.7842	-0.75283	25.21263	1
15610564	C3(Carban	0	0	0.1592	34.04	0.000828	2	889.4558	-0.29028	18.27304	0
15610564		0	0	0.03915	33.77	0.001259	3	2091.0606	-0.60748	31.13414	1
15610564	C5(Carban	0	0	0.1133	33.76	0.00042	2	820.37694	-0.15311	18.46567	0
15610564	C3(Carban	0	0	0.05049	27.97	0.001592	2	1415.6249	-1.28832	14.98828	1
15610564	M10(Oxid	0	0	0.04112	26.49	0.004039	3	2764.4813	-0.11212	39.87122	1
15610564	C5(Carban	0	0.002	0.2195	36.71	0.000363	2	790.38671	-1.18927	13.41452	0
15610564		0	0.002	0.2025	35.13	0.000306	2	895.442	-0.13193	25.23723	1
15610564		0	0.002	0.1861	31.7	0.002569	2	774.40764	-0.44024	21.1311	0
15610564	C5(Carban	0	0.002	0.272	20.2	0.009527	3	1658.748	-0.39144	15.00044	2
15610564	N-Term(A	0	0.002	0.2528	20.03	0.022345	3	1772.8908	5.311076	27.16384	0
15610564	N-Term(A	0	0.005	0.3511	12.73	0.053207	3	2120.9934	3.521944	25.95901	1

15609421	M4(Oxidat	0	0	3.91E-12	78.92	1.6E-08	3	3158.5338	-0.8674	47.35147	0
15609421		0	0	5.77E-16	75.89	2.57E-08	2	1717.7112	1.349435	22.5924	0
15609421		0	0	9.41E-11	73.79	4.17E-08	2	1296.5865	0.695735	23.77552	0
15609421		0	0	5.77E-16	72.34	1.08E-07	2	1226.6503	1.210494	22.12133	0
15609421		0	0	5.77E-16	70.4	2.14E-07	4	2901.5314	1.902952	38.79304	1
15609421		0	0	5.1E-09	70.08	9.79E-08	3	2149.9506	-3.17478	24.95788	1
15609421		0	0	5.77E-16	68.99	2.52E-07	2	1732.8705	0.657693	38.31426	0
15609421		0	0	5.77E-16	68.23	2.41E-07	3	3142.5406	-0.31464	38.82963	0
15609421	M7(Oxidat	0	0	5.77E-16	67.01	3.98E-07	2	2652.3248	2.746394	37.85675	0
15609421		0	0	5.77E-16	65.39	4.63E-07	3	2881.5357	1.258837	37.78848	1
15609421		0	0	4.18E-08	63.5	8.04E-07	2	861.45683	-1.08722	24.31991	0
15609421	C17(Carba	0	0	1.32E-09	63.13	1.02E-06	3	2636.3192	-1.27766	39.9586	0
15609421		0	0	3.05E-11	62.06	1.21E-06	3	2725.4341	1.146184	38.95595	0
15609421	M5(Oxidat	0	0	0.000517	61.85	1.08E-06	2	1826.8963	0.00905	31.73334	0
15609421		0	0	0.07859	57.19	4.68E-06	3	2459.274	0.849289	40.66392	0
15609421		0	0	2.29E-11	56.16	6.29E-06	2	1337.6827	-1.65698	28.75079	0
15609421		0	0	4.97E-05	55.05	6.56E-06	3	1794.9054	-0.56362	35.63315	0
15609421	M8(Oxidat	0	0	4.52E-05	54.77	6.67E-06	2	1810.9001	-0.7092	33.24434	0
15609421		0	0	5.52E-06	52.75	1.09E-05	2	1493.7839	-1.43275	25.72867	1
15609421		0	0	3.68E-06	52.71	8.57E-06	3	3564.7364	0.250232	34.93313	0
15609421		0	0	1.05E-05	42.64	7.62E-05	2	1024.5322	-0.17492	24.77263	0
15609421		0	0	0.02217	35.1	0.000525	2	1017.558	-0.84546	20.49714	1
15609421	M5(Oxidat	0	0	1.13E-05	34.52	0.000636	3	1842.8906	-0.31916	28.95424	0
15609421	N-Term(A	0	0	4.41E-07	34.13	0.000695	2	1268.6619	1.983612	23.1805	0
15609421	N-Term(A	0	0	4.92E-07	27.9	0.00373	4	3200.5626	4.858982	36.71868	0
15609421		0	0	0.1444	23.27	0.004699	3	1435.8145	1.076365	17.43463	1
15610011		0	0	0.01989	103.37	4.59E-11	2	1895.8764	-0.36444	28.85971	0
15610011		0	0	0.05339	97.95	2.97E-10	2	1610.8393	0.49024	24.43467	0
15610011		0	0	0.006125	90.53	1.42E-09	2	1599.7657	0.425874	30.22951	0
15610011		0	0	0.03581	86.27	4.37E-09	2	1754.8752	0.170023	41.51343	0
15610011		0	0	0.05902	80.03	3.28E-08	2	1329.7378	0.390504	20.07783	0
15610011		0	0	0.04592	78.99	3.34E-08	2	1978.0016	-0.66066	28.85593	1
15610011		0	0	0.07686	74.61	9.34E-08	2	1353.7034	-1.1336	30.92516	0
15610011		0	0	0.01636	72.69	1.13E-07	3	2362.2506	-0.37584	26.28728	0
15610011		0	0	0.05209	72.42	1.2E-07	2	2025.0179	-0.51922	36.39528	1
15610011		0	0	0.08426	69.33	1.98E-07	2	2658.2913	-1.30307	38.06651	0

15610011		0	0	0.08595	59.38	2.08E-06	2	1531.8057	0.039405	29.60823	0
15610011		0	0	0.2109	48.74	1.54E-05	2	3164.4966	0.166955	38.48186	0
15610011	N-Term(A	0	0	0.05571	45.66	6.11E-05	2	2020.0207	3.550054	30.34773	1
15610011		0	0	0.1071	38.93	0.000217	3	1932.9724	1.135537	32.43154	0
15610011		0	0	0.2389	36.54	0.000455	2	1182.6841	-0.02678	18.92538	0
15610011		0	0	0.1518	34.37	0.000365	3	2109.0014	0.837676	26.87416	1
15610375		0	0	0.002722	106.03	4.86E-11	2	1056.6164	0.273805	30.11034	0
15610375		0	0	0.001855	105.91	2.95E-11	2	1551.8748	0.400907	44.25567	0
15610375		0	0	0.005822	88.06	1.56E-09	2	1696.9955	-0.08857	33.02052	1
15610375	N-Term(A	0	0	0.00928	86.88	5.85E-09	2	1098.6276	0.869062	34.75748	0
15610375		0	0	0.00894	84.65	3.77E-09	3	4138.2231	-3.99535	49.60313	1
15610375		0	0	0.00608	82.98	1.11E-08	2	1768.9191	-2.15178	33.05719	0
15610375		0	0	0.01117	80.3	1.87E-08	2	1197.6584	-0.26594	28.74317	0
15610375		0	0	0.009336	77.63	3.19E-08	2	1835.9773	0.53444	27.01131	1
15610375		0	0	0.02462	76.4	2.86E-08	2	1420.7894	-0.99738	25.009	0
15610375		0	0	0.01215	73.46	7.66E-08	2	1455.7596	0.300725	36.25515	0
15610375	M5(Oxidat	0	0	0.03081	72.24	1.73E-07	2	1603.8288	1.619032	25.19727	0
15610375		0	0	0.03115	69.31	1.17E-07	2	2007.123	-0.23362	40.04452	1
15610375		0	0	0.06784	67.02	4.87E-07	2	1327.7117	-0.04051	26.87563	1
15610375		0	0	0.01386	66.97	2.11E-07	3	1241.7455	-0.70323	29.99625	0
15610375		0	0	0.03351	65.81	6.17E-07	2	1587.8306	-0.41396	26.31422	0
15610375		0	0	0.04168	63.55	9.71E-07	2	1171.6111	0.409934	32.76646	0
15610375	M8(Oxidat	0	0	0.01389	62.04	1.59E-06	3	1975.0197	0.896787	24.52717	1
15610375		0	0	0.02233	61.88	1.78E-06	3	1959.0176	-2.78204	25.04042	1
15610375	N-Term(A	0	0	0.03175	60.43	1.31E-06	2	1462.7997	-1.09795	33.93633	0
15610375		0	0	0.03988	59.82	1.04E-06	2	996.5363	-1.0926	16.74317	0
15610375		0	0	0.02721	58.31	2.36E-06	2	1424.7953	-1.11989	23.22801	1
15610375		0	0	0.05745	54.11	4.27E-06	3	2066.1599	-0.32237	38.79322	1
15610375	N-Term(A	0	0	0.07886	53.94	4.04E-06	2	1593.8833	-0.87705	49.03861	0
15610375	N-Term(A	0	0	0.07338	52.69	8.88E-06	3	1497.7802	7.003177	34.18537	0
15610375		0	0	0.04106	49.66	1.68E-05	3	1422.749	0.045501	25.81966	0
15610375		0	0	0.09703	48.19	3.34E-05	4	1619.8628	0.921297	21.5473	1
15610375	N-Term(A	0	0	0.104	44.75	4.02E-05	2	1038.5466	-1.34795	18.54383	0
15610375		0	0	0.1161	44.65	3.42E-05	2	1407.8512	-2.22141	21.99228	1
15610375		0	0	0.1681	42	6.29E-05	3	1251.753	-0.20527	26.43499	0
15610375		0	0	0.0309	40.29	0.000131	3	1862.0248	0.199831	24.6898	1

15610375	N-Term(A	0	0	0.1522	35.79	0.000738	3	2017.0261	-1.1825	25.87977	1
15610375	C20(Carba	0	0	0.1146	34.42	0.001211	3	2527.2856	-0.79876	32.04911	2
15610375		0	0	0.1093	26.22	0.002382	5	3869.0623	2.032238	49.17688	0
15610375	C16(Carba	0	0.003	0.2738	37.32	0.000408	2	2114.0655	2.536339	32.06715	1
15610375	N-Term(A	0	0.003	0.2716	35.43	0.000559	2	1877.996	4.841852	30.37295	1
15610375	N-Term(A	0	0.009	0.3619	10.5	0.200531	3	2569.2839	-5.53914	34.68682	2
15610079		0	0	5.25E-16	127.86	1.63E-13	2	1893.8665	-3.25716	38.34806	0
15610079		0	0	3.94E-13	121.31	1.41E-12	2	1723.9028	0.763535	49.65224	0
15610079		0	0	5.25E-16	117.65	3.35E-12	2	1720.8886	-0.75188	29.96605	0
15610079		0	0	6.19E-16	113.1	1.18E-11	3	2575.3066	1.2707	44.11329	0
15610079	M9(Oxidat	0	0	5.25E-16	109.13	2.2E-11	2	1736.8859	0.636517	34.2406	0
15610079	M6(Oxidat	0	0	5.25E-16	94.46	7.52E-10	2	2591.2974	-0.32625	35.14857	0
15610079		0	0	5.25E-16	90.04	1.93E-09	2	2279.1216	-0.38246	42.42859	0
15610079		0	0	5.25E-16	84.73	5.72E-09	2	2014.0954	-0.73819	33.30739	0
15610079		0	0	5.34E-08	84.28	3.73E-09	2	1875.0684	-0.81294	34.5263	0
15610079		0	0	5.25E-16	82.88	5.14E-09	2	1288.5491	1.279396	26.5753	0
15610079		0	0	5.25E-16	80.58	1.36E-08	2	1504.823	-0.05027	29.01113	0
15610079		0	0	5.25E-16	78.08	3.5E-08	2	2414.2093	-0.31281	33.28615	1
15610079	M13(Oxid	0	0	9.85E-16	75.84	5.86E-08	3	2423.2234	4.554481	33.05891	1
15610079	N-Term(A	0	0	6.39E-16	74.59	9.21E-08	2	2617.3138	-0.05273	37.12168	0
15610079	M9(Oxidat	0	0	5.25E-16	72.46	1.11E-07	2	1591.7929	1.958784	29.56131	0
15610079		0	0	5.66E-16	72.02	1.19E-07	2	1575.7966	1.075702	32.73452	0
15610079		0	0	4.77E-07	71.25	1.76E-07	2	2407.2168	-0.26039	39.61048	1
15610079		0	0	3.34E-13	69.19	1.99E-07	3	1889.0379	1.416427	30.73016	1
15610079	M3(Oxidat	0	0	5.25E-16	68.01	1.58E-07	2	1304.5409	-1.10778	19.37615	0
15610079		0	0	0.001637	67.52	3.54E-07	2	1028.5743	0.704907	19.82543	0
15610079	M12(Oxid	0	0	1.28E-12	60.34	1.43E-06	3	2295.1195	0.925742	35.97271	0
15610079	M6(Oxidat	0	0	3.84E-12	57.37	1.83E-06	3	1696.7688	-1.21664	15.60698	1
15610079		0	0	6.41E-12	53.83	4.13E-06	2	868.52544	0.353385	23.58399	0
15610079		0	0	0.03198	53.8	4.16E-06	3	1680.7752	-0.40435	19.06757	1
15610079		0	0	3.06E-12	53.09	4.9E-06	3	2189.2373	-1.30751	32.24663	1
15610079		0	0	1.7E-13	52.35	1.43E-05	3	2160.1102	2.861571	37.28465	0
15610079		0	0	1.1E-13	51.88	7.78E-06	2	1302.7553	1.830983	25.20796	1
15610079		0	0	1.86E-09	46.57	2.2E-05	4	2738.5414	-0.41948	37.93014	2
15610079	N-Term(A	0	0	5.04E-10	44.57	0.000119	2	786.44658	-0.36302	23.38903	0
15610079		0	0	2.58E-11	44.23	6.42E-05	2	1064.5345	-2.50802	19.79732	1

15610079	N-Term(A	0	0	0.002565	41.08	0.000203	2	2449.2303	0.912902	42.00775	1
15610079		0	0	2.07E-07	33.07	0.001356	2	1460.7829	0.234951	25.42113	1
15610079	N-Term(A	0	0	3.59E-06	29.33	0.001984	3	1931.0476	0.940046	34.10062	1
15610079		0	0	2.44E-06	22.38	0.00925	2	936.44176	-0.44026	20.42108	0
15609724		0	0	0.000176	116.77	4.31E-12	2	1589.7923	0.246967	28.20512	0
15609724		0	0	0.001002	101.24	1.17E-10	2	2145.0311	4.383227	41.82276	0
15609724		0	0	0.002695	98.09	3.1E-10	2	1398.7704	0.294574	44.29473	0
15609724		0	0	2.83E-06	78.06	2.89E-08	2	2650.3174	1.21084	36.12045	0
15609724	M9(Oxidat	0	0	0.001828	74.91	5E-08	2	2161.0228	2.862708	33.8314	0
15609724	M7(Oxidat	0	0	0.002202	71.33	7.34E-08	3	2177.0154	1.806929	33.15566	0
15609724		0	0	0.001134	68.24	3E-07	2	1029.5689	0.100969	26.38422	0
15609724		0	0	0.04557	64.03	7.31E-07	3	2267.1749	0.413984	32.4188	1
15609724	N-Term(A	0	0	0.02417	63.19	6E-07	3	2203.0268	-0.13741	35.14243	0
15609724	M22(Oxida	0	0	1.75E-05	60.57	1.58E-06	2	2666.3089	-0.09407	34.81369	0
15609724		0	0	0.000674	58.08	2.1E-06	2	1238.6767	-1.06401	49.36943	0
15609724	N-Term(A	0	0	0.003088	54.57	5.06E-06	2	1631.8029	0.274386	26.39186	0
15609724	C6(Carban	0	0	0.001418	49.92	1.83E-05	2	1270.6451	-0.76982	21.15855	0
15609724		0	0	0.00534	40.28	0.000295	2	759.4099	-1.20176	12.45028	0
15609724		0	0	0.02935	39.81	0.000198	2	785.48772	-0.36003	22.43177	0
15609724		0	0	0.07447	37.94	0.000442	3	1528.8091	0.77436	18.55473	1
15609724		0	0	0.3203	35.71	0.000309	3	4223.1031	-3.00224	39.69751	0
15609724	N-Term(A	0	0	0.1143	35.21	0.000301	3	2187.0243	-3.60771	33.86614	0
15609724		0	0	0.000763	25.41	0.006906	2	3025.5025	-1.63355	34.47231	0
15609476		0	0	0.001452	136.52	3.68E-14	2	1908.9543	-0.38244	38.97441	0
15609476		0	0	0.001763	124.57	8.55E-13	2	1541.7961	0.121162	28.21692	0
15609476	M14(Oxida	0	0	0.002675	110.01	9.95E-12	2	1823.7975	0.102342	31.90464	0
15609476	M1(Oxidat	0	0	0.004775	100.59	8.71E-11	2	1839.7878	-2.44266	28.42477	0
15609476		0	0	0.0166	91.67	6.79E-10	2	1807.8029	0.261502	32.81228	0
15609476	M1(Oxidat	0	0	0.01319	88.39	1.45E-09	2	1855.7878	0.318477	26.59454	0
15609476		0	0	0.01362	81.2	7.97E-09	2	1859.8749	-1.15909	22.96368	0
15609476		0	0	0.06403	80.86	1.23E-08	2	1388.6807	-0.83507	17.51598	1
15609476		0	0	0.04917	67.95	4.25E-07	2	1681.8666	0.531224	28.29565	0
15609476		0	0	0.06033	64.96	9.42E-07	2	1057.5723	1.136493	26.35904	0
15609476		0	0	0.0376	61.98	1.9E-06	3	2442.2278	0.694047	29.37812	1
15609476	M5(Oxidat	0	0	0.06102	61.51	2.05E-06	2	1073.5652	-0.7388	22.28467	0
15609476		0	0	0.05453	57.8	4.31E-06	2	2246.1983	-0.69518	47.95267	0

15609476		0	0	0.069	57.26	5.17E-06	2	2440.2583	0.129937	32.89755	1
15609476		0	0	0.1281	53.27	4.7E-06	2	883.41985	-0.04969	26.41466	0
15609476	C9(Carban	0	0	0.04569	50.61	8.67E-06	2	2489.0809	0.260677	33.38267	0
15609476		0	0	0.03191	48.22	1.5E-05	3	2955.2862	-1.93698	33.01062	0
15609476		0	0	0.1751	46.45	5.55E-05	2	1147.6325	0.62092	20.56486	1
15609476		0	0	0.1422	44.55	4.38E-05	3	3987.0172	1.463182	39.53527	0
15609476		0	0	0.1524	43.39	6.64E-05	2	1124.605	-0.77648	23.69752	0
15609476	M3(Oxidat	0	0	0.1624	40.41	9.08E-05	2	899.41454	-0.29903	23.31777	0
15609476		0	0	0.2527	35.74	0.00044	2	1309.7002	-0.71649	20.45677	1
15609476	N-Term(A	0	0	0.1308	35.7	0.000458	2	1950.9606	-2.53589	45.36044	0
15609476	M2(Oxidat	0	0	0.3101	34.08	0.00039	2	915.41448	5.194379	18.01016	0
15609476		0	0	0.1667	32.14	0.000609	3	1436.8243	1.54655	24.3586	1
15609476	M24(Oxida	0	0	0.1753	30.37	0.001928	5	4003.0061	-0.03885	37.3431	0
15609476	N-Term(A	0	0	0.3766	29.39	0.001148	2	941.42577	0.421184	28.37971	0
15609476	M24(Oxida	0	0	0.2303	24.1	0.009726	4	4018.9967	-1.10886	35.43132	0
15609476	M23(Oxida	0	0	0.3736	22.01	0.010072	4	4034.9984	0.579304	33.5914	0
15609476		0	0	0.3519	14.89	0.082707	2	1418.7138	0.156068	15.79503	1
15610929		0	0	4.79E-16	90.18	1.44E-09	3	2683.3009	1.526542	39.90691	0
15610929		0	0	1.61E-12	79.5	1.12E-08	2	1067.6567	-0.52216	27.49291	0
15610929	M18(Oxida	0	0	5.83E-16	73.12	4.86E-08	3	3153.4244	0.344164	30.00972	0
15610929		0	0	4.79E-16	71.73	1.71E-07	2	2205.1165	-0.99706	32.84216	0
15610929		0	0	8.93E-15	68.73	1.54E-07	2	1411.7908	0.281758	26.11813	0
15610929		0	0	1.94E-11	65.94	4.07E-07	2	1338.7837	-1.15948	28.08704	1
15610929		0	0	4.79E-16	65.04	3.13E-07	3	3137.4254	-0.96357	31.87196	0
15610929		0	0	4.79E-16	65	7.75E-07	2	1672.8323	-0.41146	34.72388	1
15610929		0	0	4.79E-16	62.37	1.01E-06	2	2256.178	0.343419	34.65735	0
15610929		0	0	3.07E-08	60.35	1.29E-06	3	2622.4153	0.017167	35.23493	1
15610929	M10(Oxida	0	0	4.44E-06	50.72	1.53E-05	3	2699.3033	4.260547	38.83414	0
15610929		0	0	7.55E-12	49.09	3.64E-05	2	1058.5617	-1.19803	25.69731	0
15610929		0	0	1.83E-11	49.04	3.18E-05	2	901.50878	-1.58143	19.63859	0
15610929		0	0	2.86E-09	47.82	1.65E-05	2	1795.0187	0.10198	27.90031	1
15610929		0	0	1.25E-07	45.32	6.32E-05	4	3277.6564	3.225316	39.4929	1
15610929		0	0	7.99E-13	44.62	7.25E-05	3	1285.7014	0.146094	24.94579	1
15610929		0	0	0.000176	41.58	0.000149	2	1041.587	-0.45089	28.47908	0
15610929		0	0	1.24E-07	41.28	0.000175	2	845.42681	0.395198	24.73073	0
15610929	M8(Oxidat	0	0	0.001442	38.52	0.000359	2	1057.5824	0.093382	23.60266	0

15610929		0	0	2.44E-07	38.28	0.000438	2	915.50463	-0.10493	29.51641	0
15610929		0	0	0.000312	36.65	0.000368	2	1506.7442	-0.44007	24.59763	1
15610929		0	0	0.02346	35.44	0.000285	2	953.53038	0.137144	24.06953	0
15610929		0	0	8.85E-07	32.2	0.001145	3	2499.2461	1.770761	38.76812	2
15610929		0	0	1.46E-05	30.06	0.001282	3	2177.2166	0.737925	26.64905	2
15610929	N-Term(A	0	0	7.95E-09	21.13	0.013876	4	3335.6476	-1.10851	37.73552	1
15610971	C9(Carban	0	0	0.002107	72.38	5.77E-08	3	1660.7964	0.262266	20.96168	0
15610971		0	0	0.0146	67.75	1.67E-07	2	1890.8783	-1.08828	26.53818	0
15610971	M1(Oxidat	0	0	0.005933	65.96	3.04E-07	2	1407.7669	0.267801	28.97466	1
15610971	M1(Oxidat	0	0	0.005543	65.96	3.8E-07	2	2018.978	-0.39001	28.13552	0
15610971	M9(Oxidat	0	0	0.01628	63.65	4.3E-07	2	1212.5572	0.486744	24.45022	0
15610971	C4(Carban	0	0	0.001772	62.02	1.29E-06	3	2159.0909	2.736876	27.95203	1
15610971		0	0	0.01737	61.35	1.47E-06	2	1122.5861	-0.27092	27.62649	0
15610971	M5(Oxidat	0	0	0.001899	61.33	7.34E-07	2	1906.8747	-0.33296	39.30609	0
15610971		0	0	0.01762	59.16	1.21E-06	2	1196.5628	0.936382	28.33994	0
15610971	C6(Carban	0	0	0.01681	56.17	2.41E-06	3	2947.263	-0.89563	30.94026	0
15610971		0	0	0.01399	55.99	3.65E-06	2	1789.8619	-1.41759	31.93409	0
15610971	M2(Oxidat	0	0	0.002823	54.44	7.02E-06	3	2175.0807	0.396458	26.01473	1
15610971	C19(Carba	0	0	0.008801	49.01	1.25E-05	3	2393.1079	-0.01756	30.07903	1
15610971		0	0	0.1595	45.77	2.91E-05	2	1391.7723	0.47644	30.59649	1
15610971		0	0	0.01787	45.34	4.82E-05	3	2279.2307	1.094994	41.64314	0
15610971		0	0	0.06216	44.87	4.07E-05	2	1322.6563	0.042565	38.633	0
15610971		0	0	0.1451	43.81	5.41E-05	2	821.42644	-0.0269	17.93513	0
15610971	M1(Oxidat	0	0	0.08611	42.36	8.13E-05	2	1138.5812	-0.08954	25.50447	0
15610971		0	0	0.06602	40.58	0.000153	2	2109.1375	1.658247	32.0515	0
15610971	M5(Oxidat	0	0	0.1089	37.85	0.000213	2	1338.6526	1.104984	36.21282	0
15610971	C9(Carban	0	0	0.1049	37.67	0.000325	4	1816.8969	-0.1288	19.8572	1
15610971	C6(Carban	0	0	0.0904	35.78	0.000264	4	4140.8053	0.21717	34.34488	1
15610971	C6(Carban	0	0	0.0705	26.66	0.002153	4	4124.8292	4.785662	35.34625	1
15610971	C6(Carban	0	0	0.09	20.48	0.008932	4	4108.8117	-0.71142	36.68723	1
15610971	N-Term(A	0	0	0.2203	14.02	0.039534	2	1948.881	-2.48977	27.05202	0
15608991		0	0	4.91E-07	108.09	1.55E-11	2	1402.806	0.423795	31.82945	0
15608991		0	0	2.79E-08	107.47	2.78E-11	2	3089.6338	-2.62964	41.2373	0
15608991		0	0	5.46E-05	79.3	2.76E-08	2	1163.6061	0.47731	25.98194	0
15608991		0	0	5.87E-06	73.05	1.11E-07	2	1019.5267	-0.19748	21.23154	0
15608991		0	0	0.008962	69.19	1.63E-07	2	1113.662	-0.59838	29.5901	1

15608991		0	0	0.002003	64.84	4.59E-07	2	1098.5528	-1.0007	17.25641	0
15608991		0	0	0.000126	63.6	1E-06	2	1031.5367	-0.14794	30.64647	0
15608991		0	0	0.000829	59.74	1.06E-06	2	1675.983	0.785482	37.07104	0
15608991	M14(Oxid	0	0	8.01E-05	56.07	2.47E-06	2	1691.9747	-1.12255	33.86538	0
15608991		0	0	6.01E-06	54.56	8.57E-06	2	1376.7272	-0.6834	25.1683	1
15608991	C6(Carban	0	0	0.000603	52.1	1.17E-05	3	1863.9485	-0.55037	22.77864	2
15608991	M1(Oxida	0	0	0.01916	41.77	7.65E-05	2	1482.6996	-0.67022	15.93164	0
15608991		0	0	0.4654	40.64	0.000181	2	1144.6061	-0.7379	15.66449	1
15608991		0	0	8.1E-06	23.9	0.005907	4	3863.0448	0.887696	45.8967	0
15610058		0	0	0.005184	103.51	4.45E-11	2	2344.0992	-2.59059	29.96475	0
15610058		0	0	0.003659	89.39	1.15E-09	2	1645.772	0.916868	29.47482	0
15610058		0	0	0.03445	86.09	6.64E-09	2	1372.6958	-0.73791	26.55506	0
15610058		0	0	0.01489	84.65	7.71E-09	2	1683.8558	0.510251	27.27278	0
15610058		0	0	0.04314	80.35	1.94E-08	3	3125.5785	1.059973	45.20429	1
15610058		0	0	0.01147	79.99	2.21E-08	2	1498.8249	1.598186	31.08324	0
15610058		0	0	0.2504	72.11	8.3E-08	2	3321.6255	-0.60977	34.27357	0
15610058		0	0	0.05309	70.87	8.18E-08	3	4008.9448	0.622617	31.00173	1
15610058	M11(Oxid	0	0	0.1688	69.18	1.75E-07	2	1515.7466	-0.41344	28.21786	0
15610058		0	0	0.0734	68.69	1.83E-07	2	1048.5766	-1.95085	25.41618	0
15610058		0	0	0.09828	67.21	1.9E-07	2	1096.6351	-0.98842	21.99873	0
15610058		0	0	0.03216	66.43	3.64E-07	3	1547.8687	0.42975	35.44773	0
15610058		0	0	0.02951	62.5	7.03E-07	3	2870.391	-0.22888	25.031	1
15610058		0	0	0.1138	55.86	4.02E-06	3	1204.6791	-0.58193	23.79938	1
15610058		0	0	0.05119	53.53	8.65E-06	3	3477.7258	-0.82758	33.15477	1
15610058	M15(Oxid	0	0	0.1526	46.37	5.65E-05	2	1955.0391	0.574822	32.00602	1
15610058		0	0	0.1981	45.81	3.67E-05	4	3777.9782	0.245851	44.01834	2
15610058		0	0	0.05841	34.63	0.000568	2	1499.7493	-2.01775	30.38937	0
15610058		0	0	0.316	31.96	0.000635	2	1054.6	-0.3775	18.20816	0
15610058	M15(Oxid	0	0	0.315	31.69	0.001423	4	2311.2528	-1.02887	31.52177	2
15610058		0	0	0.2517	23.39	0.010079	3	1939.0446	0.783126	33.23851	1
15610583		0	0	0.7056	80.88	1.51E-08	2	1297.7188	2.848305	38.78749	0
15610583		0	0	0.651	49.77	1.05E-05	2	1177.7052	0.014005	26.10407	0
15610583		0	0	0.7514	43.27	0.000125	2	963.51012	-1.42489	28.14585	0
15610583		0	0	0.6366	37.48	0.000277	3	1296.7754	0.542482	23.4766	1
57116701		0	0	0.1287	96.95	3.03E-10	3	3435.6738	0.972136	46.84489	0
57116701	C21(Carba	0	0	0.1166	77.25	3.67E-08	2	2652.3584	-0.22594	44.13994	0

57116701	M10(Oxid	0	0	0.3819	63.7	4.26E-07	3	4070.9183	2.203871	44.02969		0
57116701		0	0	0.06898	62.74	9.05E-07	4	2980.4611	-1.25518	43.52537		0
57116701	C9(Carban	0	0	0.2481	62.28	6.21E-07	2	1510.7209	-0.77612	26.18025		0
57116701		0	0	0.4702	57.45	4.05E-06	2	1019.5355	-1.27732	20.50874		0
57116701	M5(Oxidat	0	0	0.412	41.25	0.000127	2	1036.5444	-1.20321	20.7129		0
57116701	M10(Oxid	0	0	0.4942	40.1	9.75E-05	3	4086.9104	1.498004	41.75913		0
57116701		0	0	0.4449	32.18	0.000878	2	1155.6712	-1.79214	28.75175		1
15611030		0	0	0.000067	86.37	2.54E-09	2	1826.8715	0.698604	25.26058		0
15611030		0	0	4.75E-05	77.51	4.52E-08	2	1445.7804	1.578252	36.69717		0
15611030	M12(Oxid	0	0	0.01948	74	1.04E-07	2	1461.7735	0.279659	31.81401		0
15611030	C10(Carba	0	0	9.4E-05	69.45	1.13E-07	3	2212.0158	-0.78799	30.55987		0
15611030		0	0	0.113	67.42	3.62E-07	2	2178.179	1.097879	36.45047		0
15611030	M9(Oxidat	0	0	0.000436	61.54	7E-07	2	1842.8661	0.537297	21.66167		0
15611030		0	0	0.006556	60.94	2.62E-06	2	1129.6319	-0.49376	25.77291		0
15611030		0	0	0.001444	57.35	3.77E-06	2	1940.0358	0.460249	29.83892		0
15611030	M6(Oxidat	0	0	0.000498	55.79	2.63E-06	2	1579.7165	-0.32793	19.44734		0
15611030		0	0	0.01419	55.47	2.83E-06	2	1563.7225	0.241992	22.38129		0
15611030		0	0	0.06917	54.65	8.06E-06	2	1341.7663	-0.68443	29.93902		1
15611030	N-Term(A	0	0	0.08233	54.32	1.26E-05	2	1171.6446	1.342172	31.85698		0
15611030	M6(Oxidat	0	0	0.1093	52.19	1.39E-05	2	1088.5762	-0.64931	29.30915		0
15611030		0	0	0.001684	51.15	2.38E-05	2	931.48253	-1.98693	18.64163		0
15611030	M10(Oxid	0	0	0.2292	49.26	1.18E-05	2	1763.7887	0.372827	32.77579		0
15611030		0	0	0.2656	47.75	1.67E-05	2	1747.7934	0.120872	36.18189		0
15611030		0	0	0.07155	46.44	2.27E-05	2	775.46782	0.721805	20.96535		0
15611030	N-Term(A	0	0	0.003609	45.57	7.21E-05	3	1982.0451	-0.20595	33.29741		0
15611030		0	0	0.000924	44.98	5.88E-05	2	873.50377	-0.33263	18.68609		0
15611030		0	0	0.08103	44.97	8.28E-05	2	1585.8595	0.65792	31.02318		0
15611030		0	0	0.1113	44.79	8.63E-05	2	1072.5817	-0.27845	31.30841		0
15611030	N-Term(A	0	0	0.0106	43.98	9.6E-05	2	1185.6387	1.006355	29.38287		0
15611030		0	0	0.2698	39.95	0.000121	3	1565.8811	3.350425	23.6311		1
15611030	M8(Oxidat	0	0	0.1128	39.59	0.00011	2	1779.7822	-0.47714	29.3385		0
15611030	N-Term(A	0	0	0.007076	35.78	0.000687	2	1114.5916	-0.87563	33.33409		0
15611030		0	0	0.1003	30.55	0.000969	2	1053.5838	-0.25007	21.35573		0
15611030	M8(Oxidat	0	0	0.08559	29.69	0.002255	2	1357.7637	1.180746	27.36287		1
15611030		0	0	0.04617	27.38	0.002377	2	1027.532	0.037683	21.09964		0
57117112		0	0	5.84E-05	111.71	1.48E-11	2	2137.094	-3.92101	33.28056		0

57117112		0	0	0.00147	103.51	1.18E-10	2	1781.9035	0.324755	34.93712	0
57117112		0	0	0.003985	92.6	1.35E-09	2	2456.2325	-0.57072	40.9557	0
57117112	M2(Oxidat	0	0	0.003978	90.87	1.88E-09	2	2472.2352	2.576098	38.91382	0
57117112		0	0	0.009578	78.42	2.52E-08	4	2311.1395	1.793822	37.64151	0
57117112		0	0	0.002951	71.07	1.88E-07	2	1485.7698	0.095364	28.30999	0
57117112		0	0	0.002257	71	1.55E-07	2	1952.957	-1.59989	32.89218	0
57117112		0	0	0.000665	69.1	1.48E-07	4	3186.6701	1.824271	40.71771	0
57117112		0	0	0.01486	64.14	8.67E-07	2	1657.9103	4.954803	30.05242	0
57117112		0	0	0.005015	62.65	5.43E-07	2	3033.4224	1.262462	39.20384	0
57117112		0	0	0.03634	60.53	1.02E-06	2	1676.8034	0.358435	30.87877	0
57117112		0	0	0.01433	59.13	1.22E-06	2	1043.658	0.762015	24.76932	1
57117112		0	0	0.05747	58.77	1.99E-06	2	1064.5734	-0.1122	28.33788	0
57117112		0	0	0.01268	53.04	9.19E-06	3	3263.6396	-1.09379	39.95629	1
57117112		0	0	0.01327	50.31	1.54E-05	3	1660.9169	-0.0825	43.99079	0
57117112		0	0	0.03373	50.14	1.11E-05	2	776.44267	1.652732	23.11376	0
57117112		0	0	0.2483	49.6	1.09E-05	2	1035.4458	-0.69817	29.40721	0
57117112	M5(Oxidat	0	0	0.21	40.7	8.49E-05	2	1051.4413	-0.14696	25.54557	0
57117112		0	0	0.06151	39.63	0.000109	2	910.49932	0.037207	24.46939	0
57117112		0	0	0.2182	38.22	0.00015	3	1574.9992	-0.03848	34.78558	0
57117112		0	0	0.06469	34.39	0.00091	3	1275.6771	1.98	22.68479	1
57117112	M10(Oxid	0	0	0.04399	32.67	0.000539	3	1479.6518	-1.03248	18.16584	1
15610690		0	0	0.02022	120.28	1.08E-12	3	3637.9566	-3.73648	39.50094	1
15610690		0	0	0.01409	109.66	1.78E-11	2	2022.9847	-1.65822	31.24503	0
15610690		0	0	0.02027	100.27	1.08E-10	3	3055.6501	1.207043	46.67737	1
15610690		0	0	0.013043	99.98	1.51E-10	2	1956.0796	-0.27292	47.88151	0
15610690		0	0	0.02634	91.03	9.07E-10	2	2566.4139	0.40705	46.71155	0
15610690	C7(Carban	0	0	0.0353	89.42	1.14E-09	2	1749.6438	-0.57833	16.61117	0
15610690		0	0	0.05355	76.3	3.05E-08	2	2083.1524	-1.10887	34.68304	0
15610690	C17(Carba	0	0	0.05695	70.4	1.87E-07	3	3034.5329	1.654885	40.34197	0
15610690		0	0	0.305	67.26	3.76E-07	2	1090.5752	-0.00769	13.97102	0
15610690	M8(Oxidat	0	0	0.1214	64.92	5.64E-07	2	1375.6862	-1.79654	26.20426	0
15610690		0	0	0.3073	60.84	1.15E-06	2	1152.616	-0.04937	26.19829	0
15610690		0	0	0.08932	60.63	9.95E-07	3	2588.3822	-0.40026	34.96971	1
15610690		0	0	0.3212	60.28	2.2E-06	2	916.48473	0.007065	17.01164	0
15610690	C5(Carban	0	0	0.1979	57.51	1.86E-06	2	1152.5452	-1.25087	20.67284	0
15610690		0	0	0.2716	56.89	2.04E-06	2	1384.6689	-1.0764	20.18228	0

15610690		0	0	0.3661	56.79	3.35E-06	2	1169.6059	-0.29817	17.61514	0
15610690		0	0	0.4606	50.06	2.47E-05	2	1658.8398	-0.04342	24.34947	1
15610690		0	0	0.1982	41.63	0.000103	2	983.53325	2.368424	30.36416	0
15610690		0	0	0.3643	41.57	0.000174	2	1179.5743	-0.89435	16.9836	1
15610690		0	0	0.5175	39.61	0.000224	2	1088.5683	-1.1358	16.24678	1
15610690		0	0	0.268	38.6	0.000138	2	1023.4731	-1.1053	18.83332	0
15610690		0	0	0.2926	34.83	0.000526	5	3556.8641	-0.01077	40.82672	0
15610690	C6(Carban	0	0	0.4231	31.1	0.001164	2	1308.6462	-1.22993	18.19318	1
15610690		0	0	0.4529	28.26	0.001791	2	1280.7103	-0.61577	23.72344	1
15610690		0	0	0.1761	21.1	0.011644	4	3513.7826	0.669415	47.81631	0
15610690		0	0	0.1209	12.56	0.061009	5	4206.2391	1.02745	46.92866	2
15609463		0	0	0.02495	96.29	3.52E-10	2	1752.9468	-1.32141	35.35581	0
15609463		0	0	0.015383	89.68	2.31E-09	3	2600.3514	-0.51061	34.34289	1
15609463		0	0	0.06022	78.19	1.52E-08	2	1593.9347	1.486059	35.21271	0
15609463		0	0	0.1375	77.49	3.65E-08	2	1399.7795	0.18949	33.84094	0
15609463		0	0	0.02577	76.5	3.13E-08	2	1356.7843	-0.19306	42.28579	0
15609463		0	0	0.06466	72.26	5.93E-08	2	2027.9413	-0.2266	21.50957	0
15609463		0	0	0.1475	63	5E-07	2	911.60332	-0.44122	31.95693	0
15609463		0	0	0.184	61.27	7.45E-07	2	869.55693	0.185226	28.30527	0
15609463		0	0	0.1246	60.3	1.54E-06	2	1219.5906	-0.62113	19.79012	0
15609463		0	0	0.1276	59.43	1.71E-06	2	1104.547	0.328976	19.135	0
15609463		0	0	0.05842	57.06	3.25E-06	2	1962.074	-1.41131	27.27818	0
15609463		0	0	0.06107	55.16	8.38E-06	3	1528.8071	-0.48977	28.33386	1
15609463		0	0	0.1713	29.47	0.002542	3	1260.6466	-0.94493	18.91776	1
57117112		0	0	0.001173	122.53	1.4E-12	2	2026.0697	-0.29197	44.08087	0
57117112		0	0	0.07602	90.3	2.61E-09	2	2064.0369	0.582128	29.9658	0
57117112		0	0	0.02279	88.82	3.74E-09	2	974.52611	-0.49428	19.92515	0
57117112		0	0	0.03104	84.29	9.5E-09	2	1486.8234	0.63932	31.55989	0
57117112		0	0	0.05328	78.95	1.4E-08	2	1291.7259	0.159768	33.70919	0
57117112		0	0	0.0605	77.62	5.02E-08	2	1428.7599	0.338751	34.62741	0
57117112		0	0	0.04042	75.4	3.89E-08	2	1201.689	-0.7896	28.4528	0
57117112		0	0	0.0106	72.83	5.73E-08	2	3045.6358	-0.36167	39.70924	0
57117112		0	0	0.0116	71.28	1.71E-07	2	1672.8672	-1.30686	30.51026	0
57117112		0	0	0.08329	69.92	1.17E-07	2	957.57311	0.308468	26.65962	0
57117112		0	0	0.1167	59.18	2.9E-06	3	2518.3302	0.032977	38.51573	0
57117112		0	0	0.1946	58.76	1.53E-06	3	2583.4147	0.188008	50.00474	1

57117112	C4(Carban	0	0	0.2505	56.04	3.73E-06	2	1770.8583	0.225126	23.0425	0
57117112		0	0	0.4332	52.83	9.64E-06	2	1150.5711	-2.45859	24.35453	0
57117112	M16(Oxid	0	0	0.1435	50.26	1.22E-05	3	3790.964	0.674255	45.86472	0
57117112		0	0	0.5135	49.82	1.77E-05	2	1109.5613	-1.1756	23.44264	0
57117112		0	0	0.2877	48.71	2.42E-05	2	776.44072	-0.86275	23.90832	0
57117112		0	0	0.07656	45.75	6.25E-05	2	1973.0068	0.598156	43.15508	0
57117112		0	0	0.3618	44.16	6.14E-05	3	1996.0873	0.471969	49.61204	1
57117112		0	0	0.3589	24.19	0.00705	4	2951.5954	0.100393	47.87906	2
57117112		0	0	0.3773	14.09	0.07019	3	1630.9127	0.221682	27.94475	1
15611006		0	0	0.009539	108.87	1.29E-11	2	1692.9758	0.091145	29.16851	0
15611006		0	0	0.007273	98.17	2.67E-10	2	2138.087	-1.95485	33.57055	0
15611006	N-Term(A	0	0	0.01316	92.68	5.93E-10	2	1734.987	0.472497	36.44393	0
15611006		0	0	0.000954	86.37	4.61E-09	3	2232.1651	-0.39739	37.04315	0
15611006		0	0	0.002185	78.11	2.32E-08	2	1421.7277	0.385047	26.35079	0
15611006		0	0	0.00546	77.81	2.73E-08	2	1059.5425	-0.41832	22.39315	0
15611006		0	0	0.006692	72.36	1.1E-07	2	1504.8014	0.530236	25.84878	0
15611006		0	0	0.03037	63.8	7.3E-07	2	1219.5982	-0.49128	26.99528	0
15611006	M1(Oxidat	0	0	0.005692	62.33	8.48E-07	2	1235.5947	0.765454	24.22741	0
15611006		0	0	0.01009	60.81	9.96E-07	2	1383.6612	-0.0151	14.52803	1
15611006		0	0	0.0703	59.41	1.14E-06	2	1956.9152	-0.36924	20.50779	1
15611006	M11(Oxid	0	0	0.006007	58.8	2.7E-06	3	2154.0862	0.074042	26.90942	0
15611006		0	0	0.00441	58.71	2.36E-06	2	2323.1629	-2.11432	31.39571	0
15611006	N-Term(A	0	0	0.01205	56.54	2.21E-06	2	1279.7123	0.438827	29.27963	0
15611006		0	0	0.03471	49.41	1.14E-05	2	1237.7002	-0.77433	24.34183	0
15611006		0	0	0.02496	48.59	2.91E-05	4	2226.0993	-0.83439	22.66158	2
15611006	M1(Oxidat	0	0	0.1153	48.25	2.62E-05	2	1391.695	0.12042	22.60544	1
15611006		0	0	0.1931	47.96	4E-05	2	874.43303	-1.04102	19.74904	0
15611006		0	0	0.02058	46.33	5.59E-05	3	1375.6995	-0.32331	24.2131	1
15611006		0	0	0.1055	41.64	6.84E-05	2	1026.4911	-1.3491	25.46268	0
15611006		0	0	0.08233	40.93	0.000178	3	1361.7412	-0.84172	24.31326	1
15611006		0	0	0.07	39.61	0.000137	3	2033.9775	-0.68838	20.92376	0
15611006	N-Term(A	0	0	0.0733	39.11	0.00019	2	1101.5507	-2.56867	26.89899	0
15611006		0	0	0.1705	39.03	0.000225	2	861.45738	-0.43795	18.64571	0
15611006		0	0	0.008798	36.15	0.000255	3	1973.9359	-1.41094	27.48042	0
15611006		0	0	0.05318	35.97	0.000252	2	1172.5018	1.403224	14.15484	1
15611006		0	0	0.1417	29.24	0.002204	3	1790.8802	-0.68634	19.37686	1

15611006		0	0	0.07076	27.69	0.003064	2	864.45763	0.216165	25.13806	0
15611006	M1(Oxidat	0	0	0.2614	19.25	0.033278	3	1989.054	-0.35548	26.89513	2
15611006		0	0	0.2614	18.49	0.028316	3	1973.0594	-0.21336	28.82119	2
15610501	M8(Oxidat	0	0	3.81E-05	113.01	4.99E-12	2	1353.5947	-0.37988	18.86984	0
15610501		0	0	0.00743	110.4	1.87E-11	2	1342.7682	-0.54754	39.10708	0
15610501		0	0	5.11E-05	106.26	2.36E-11	2	1337.5997	-0.44432	22.51546	0
15610501		0	0	0.01273	99.03	2.75E-10	2	1540.8809	0.172417	33.7701	0
15610501		0	0	0.01411	95.39	5.49E-10	3	3402.7899	-0.18843	38.97921	0
15610501		0	0	0.05151	85.09	7.43E-09	2	1742.9256	-1.60454	36.12753	0
15610501		0	0	0.01947	77.72	3.3E-08	2	1466.7603	0.300121	24.66326	0
15610501		0	0	0.01472	77.54	2.03E-08	2	1696.9834	0.992783	32.28637	1
15610501		0	0	0.00615	77.17	3.93E-08	2	1996.0009	-1.0467	22.24679	1
15610501	M6(Oxidat	0	0	0.0213	77.06	2.46E-08	2	1485.7072	-0.33343	29.64354	0
15610501		0	0	0.03413	70.54	1.02E-07	2	1469.7153	1.684857	38.47941	0
15610501	N-Term(A	0	0	0.05626	65.75	7.98E-07	2	1114.6081	-2.01548	35.86984	0
15610501		0	0	0.01466	65.22	4.51E-07	2	1839.9006	-0.71254	23.74154	0
15610501	M6(Oxidat	0	0	0.001773	63.37	4.59E-07	2	1501.702	-0.4391	29.51828	0
15610501	M15(Oxid	0	0	0.03469	63.36	8.3E-07	2	1855.8964	-0.20281	21.10718	0
15610501		0	0	0.0002	62.69	1.16E-06	3	2554.274	-0.1763	35.23618	1
15610501		0	0	0.05507	61.09	1.52E-06	2	1277.6862	1.012397	22.28925	0
15610501		0	0	0.004293	60.52	8.87E-07	3	1493.7005	-0.58056	20.72228	1
15610501		0	0	0.0223	60.03	2.48E-06	3	2569.287	-0.91848	43.20351	1
15610501		0	0	0.03354	59.59	1.48E-06	3	3357.6559	-1.20155	40.00396	0
15610501	M16(Oxid	0	0	0.003501	58.95	2.87E-06	2	2011.9974	-0.2705	19.75895	1
15610501		0	0	0.002424	56.91	2.03E-06	2	1059.5068	0.160609	14.38654	0
15610501		0	0	0.02031	55.41	7.77E-06	4	2725.3903	-0.08138	41.1335	2
15610501		0	0	0.11	51.27	2.28E-05	2	1072.5989	-0.78011	25.20042	0
15610501		0	0	0.03922	49.58	2.37E-05	2	1328.6922	0.304789	16.05158	1
15610501	N-Term(A	0	0	0.008958	48.73	3.48E-05	2	1057.5607	-0.4631	32.37388	0
15610501		0	0	0.01545	48.52	3.59E-05	2	920.48436	0.782483	23.99606	0
15610501	M2(Oxidat	0	0	0.1021	47.4	5E-05	2	1031.5469	1.324514	25.4647	0
15610501		0	0	0.2853	45.15	4.58E-05	3	1986.0547	-3.42873	34.39308	1
15610501		0	0	0.2175	39.1	0.000185	2	1561.7593	-0.81085	30.28146	1
15610501	N-Term(A	0	0	0.2202	38.07	0.000242	2	1319.6844	2.142536	16.99932	1
15610501		0	0	0.09388	37.89	0.000488	2	1015.5502	-0.47643	27.77874	0
15610501		0	0	0.07619	37.55	0.000175	2	795.44695	-0.31968	14.70222	0

15610501	M8(Oxidat	0	0	0.2928	35.78	0.000264	3	1509.6949	-0.8852	16.72393	1
15610501		0	0	0.0344	33.48	0.00083	3	1277.6707	-0.25309	14.53002	1
15610501		0	0	0.00825	32.28	0.00059	3	2407.1074	0.653769	31.90837	0
15610501		0	0	0.05495	31.09	0.001323	2	1007.514	-1.68502	17.55376	0
15610501	M6(Oxidat	0	0	0.051	30.24	0.001372	3	1657.8025	-0.76484	24.43185	1
15610501		0	0	0.09854	25.44	0.005572	2	1625.8128	-0.68247	34.00921	1
15610501		0	0	0.2966	24.79	0.005808	2	1341.7843	-0.44861	26.90373	1
15610501		0	0	0.08375	19.09	0.026512	3	3285.6705	-0.19051	41.16186	2
15610501		0	0	0.07479	16.89	0.056277	3	1430.7367	-1.43552	23.46197	1
15610501		0	0	0.2151	11.07	0.195407	3	2317.2035	0.001754	44.47077	1
57116694		0	0	0.007144	53.22	4.75E-06	3	2996.6865	1.473379	49.86352	0
57116694		0	0	0.01348	52.77	5.55E-06	3	4295.0973	2.6034	44.93911	0
15609885		0	0	9.27E-07	95.65	5.58E-10	2	1293.679	-0.6096	26.00156	0
15609885		0	0	1.26E-15	94.44	3.59E-10	2	2727.0874	0.747553	36.51467	0
15609885		0	0	0.02005	58.48	3.83E-06	2	858.50566	1.4811	23.55168	0
15609885		0	0	1.87E-08	56.55	4.09E-06	2	1268.649	0.640114	26.9225	0
15609885	M2(Oxidat	0	0	2.84E-06	45.97	2.52E-05	2	1040.4745	-0.60203	17.98953	0
15609885	M19(Oxida	0	0	0.006676	32.68	0.000944	3	2844.3889	-2.07502	47.87444	0
15610746		0	0	0.000449	102.02	1.6E-10	2	1342.7061	-0.9898	21.51975	0
15610746		0	0	0.0301	95.01	6.63E-10	3	2462.237	-1.65649	31.78845	1
15610746	M4(Oxidat	0	0	0.000234	94.02	9.91E-10	2	2217.1536	-0.39431	41.16056	0
15610746	M2(Oxidat	0	0	0.05622	86.8	2.08E-09	2	1607.7137	1.097425	16.82747	0
15610746	M3(Oxidat	0	0	0.03096	86.25	3.68E-09	2	1541.7443	-0.55198	31.64582	0
15610746		0	0	0.01642	85.78	2.64E-09	2	1964.9396	0.662358	37.85014	0
15610746	M22(Oxida	0	0	0.000973	82.41	5.73E-09	3	2987.382	-3.13456	31.10382	1
15610746		0	0	0.02106	81.3	1.74E-08	2	2201.1597	0.065542	43.13577	0
15610746		0	0	0.001836	80.63	1.69E-08	2	1509.7569	1.028137	40.79771	0
15610746	M11(Oxida	0	0	0.03495	79.91	1.12E-08	2	1525.7511	0.589829	35.10421	0
15610746		0	0	0.04057	75.01	3.47E-08	3	2971.3897	-2.25405	37.73255	1
15610746	M11(Oxida	0	0	0.03572	71.9	1.26E-07	2	1980.936	1.375302	33.00023	0
15610746		0	0	0.01862	70.99	7.94E-08	3	2496.147	-0.42245	30.91853	0
15610746		0	0	0.05012	69.55	1.11E-07	2	1591.7166	-0.24563	18.68305	0
15610746	M5(Oxidat	0	0	0.1333	66.14	5.84E-07	2	1782.8978	-0.72557	34.64759	1
15610746		0	0	0.1063	63.67	1.07E-06	2	934.49761	-1.82462	25.79333	0
15610746		0	0	0.1133	62.85	1.4E-06	2	985.52867	-2.70739	21.99615	0
15610746	M5(Oxidat	0	0	0.1995	58.18	2.81E-06	3	1798.8927	-0.68206	29.72354	1

15610746		0	0	0.2209	58.17	4.65E-06	3	1635.8432	-1.11716	18.96668	1
15610746	M3(Oxidat	0	0	0.02227	56.01	2.5E-06	2	1174.5195	-0.29759	26.47956	0
15610746		0	0	0.1785	53.53	4.43E-06	2	1158.5251	0.155985	32.18738	0
15610746	M2(Oxidat	0	0	0.01154	53.1	4.89E-06	2	1235.528	-0.25867	18.66534	0
15610746		0	0	0.09499	42.61	5.47E-05	2	1334.6133	0.665023	22.90076	0
15610746	M2(Oxidat	0	0	0.04713	42.34	5.82E-05	3	2478.119	-0.51435	27.6499	1
15610746		0	0	0.1814	38.64	0.000328	2	1105.5997	-0.39284	21.92107	1
15610746		0	0	0.2008	32.71	0.000857	2	976.48412	-0.60818	22.36755	0
15610746		0	0	0.2209	23.03	0.004966	3	2462.1245	-0.32713	28.43877	1
15610746		0	0	0.2128	16.5	0.022334	4	2512.4686	-1.47372	36.62979	0
15610329		0	0	0.000394	114.68	8.68E-12	2	2043.056	0.6434	35.29288	0
15610329		0	0	0.003983	97.05	1.97E-10	2	1750.801	1.027883	22.4918	0
15610329		0	0	0.001107	91.8	1.39E-09	2	1754.8859	-0.11365	34.73749	0
15610329		0	0	0.000961	87.98	3.74E-09	2	2244.1902	-0.41358	37.22196	0
15610329	M13(Oxid	0	0	0.002785	86.42	2.27E-09	2	1700.7529	-0.9708	24.90539	0
15610329		0	0	0.00311	86	3.14E-09	2	1481.8437	0.090582	36.73614	0
15610329		0	0	0.001522	84.08	3.9E-09	3	3204.5174	-0.59358	27.83219	1
15610329	N-Term(A	0	0	0.002568	83.14	4.84E-09	2	1792.8107	0.55792	21.0611	0
15610329		0	0	0.001825	81.47	1.57E-08	2	2042.0157	-2.27163	22.66549	0
15610329		0	0	0.003128	80.4	1.37E-08	2	2427.1795	-1.3515	32.67997	0
15610329		0	0	0.006177	80.13	1.41E-08	2	1426.7341	1.047909	34.40164	0
15610329		0	0	0.005893	78.71	2.69E-08	2	1472.7378	-0.12272	27.74707	0
15610329		0	0	0.007134	72.28	5.9E-08	2	1684.7583	-0.73774	30.42827	0
15610329		0	0	0.005141	71.44	1.26E-07	2	2125.1255	0.308019	31.71993	0
15610329		0	0	0.05413	61.58	1.77E-06	2	1347.7148	1.437974	23.11893	1
15610329		0	0	0.02108	58.68	2.17E-06	2	1350.7027	1.046135	26.89014	0
15610329		0	0	0.005332	57.83	2.97E-06	2	1990.0604	-2.08082	35.90862	0
15610329		0	0	0.009185	56.94	2.23E-06	3	2494.1896	-0.8708	21.51729	1
15610329		0	0	0.0131	55.55	4.74E-06	2	1239.7222	1.076263	36.02072	0
15610329		0	0	0.1075	55.16	4.42E-06	2	1255.606	-0.4991	25.73459	0
15610329		0	0	0.006976	54.99	7.77E-06	3	2102.0874	-0.16452	28.00125	1
15610329		0	0	0.01739	48.54	2.8E-05	3	2400.2907	-0.64021	35.41858	1
15610329		0	0	0.0279	47.43	1.99E-05	2	1677.7861	-2.82096	35.75348	0
15610329		0	0	0.02613	47.41	3.63E-05	3	1800.8774	0.107294	25.45756	1
15610329		0	0	0.07111	47.05	2.37E-05	2	1125.6064	1.0568	23.84594	1
15610329		0	0	0.02031	46.86	4.12E-05	3	1482.7653	-0.49831	24.82103	1

15610329	N-Term(A	0	0	0.03304	46.1	4.17E-05	2	1281.7356	3.274468	44.23871	0
15610329		0	0	0.1417	44.66	7.01E-05	2	793.41985	-0.58468	17.19827	0
15610329	N-Term(A	0	0	0.01908	44.09	5.46E-05	2	1514.7465	-1.37234	31.79057	0
15610329		0	0	0.04302	39.17	0.000121	2	1508.9054	-0.41632	36.82449	1
15610329		0	0	0.2151	36.63	0.000532	2	1442.7522	1.589715	23.68268	1
15610329		0	0	0.06565	35.7	0.000269	3	4011.9096	-0.98213	42.90689	0
15610329		0	0	0.1926	31.12	0.000771	2	971.43248	-0.07167	19.27895	0
15610329		0	0	0.2668	30.26	0.001413	2	911.45018	-0.8332	27.21688	0
15610329		0	0	0.1161	26.96	0.005135	3	1229.6739	-0.91815	21.61722	1
15610329		0	0	0.1546	25.43	0.005442	2	2167.1043	1.801642	29.73812	2
15610329	N-Term(A	0	0	0.3457	12.33	0.070175	3	2469.1924	-0.37232	37.307	0
15607225	M1(Oxidat	0	0	0.08438	92.51	5.6E-10	2	1658.7847	0.222613	27.37134	0
15607225	C6(Carban	0	0	0.1435	84.38	8.21E-09	2	1542.8139	2.654288	36.50144	0
15607225		0	0	0.06669	83.62	7.17E-09	2	1642.7907	0.770465	28.94656	0
15607225	M12(Oxida	0	0	0.1323	80.1	1.61E-08	2	1789.9886	0.227202	37.81664	0
15607225		0	0	0.1365	73.98	8.8E-08	3	2357.1714	-0.79351	28.73437	0
15607225	N-Term(A	0	0	0.2243	59.31	2.58E-06	2	1115.5679	-1.13629	29.50116	0
15607225		0	0	0.1961	52.63	1.15E-05	2	1073.5583	-0.32244	20.62882	0
15607225		0	0	0.3637	40.24	0.000175	2	1606.8547	-0.093	26.32085	1
15607225	M12(Oxida	0	0	0.1749	30.19	0.000955	3	1946.0905	0.617625	35.80665	1
15607225		0	0	0.2594	26.61	0.002401	3	1930.0938	-0.27258	47.38637	1
15607225		0	0	0.1431	19.03	0.021254	3	1202.5987	0.005141	20.61436	1
15607234	M2(Oxidat	0	0	0.02244	122.4	1.18E-12	2	1833.8996	-0.88476	26.91472	0
15607234		0	0	0.009825	118.52	4.08E-12	2	1483.8478	-0.09124	40.04091	0
15607234	N-Term(A	0	0	0.01844	113.91	7.52E-12	2	1859.9164	-0.22951	28.25323	0
15607234	M6(Oxidat	0	0	0.03115	112.17	1.21E-11	2	1864.9333	-0.13468	23.72414	0
15607234		0	0	0.02366	109.62	1.86E-11	2	1817.9064	0.070614	28.2813	0
15607234		0	0	0.04362	92.12	1.1E-09	2	1780.9454	0.740505	26.76794	1
15607234		0	0	0.07609	88.43	2.01E-09	2	1546.8593	0.290413	37.27947	0
15607234		0	0	0.04699	86.93	3.95E-09	2	1567.8583	0.295094	41.26927	0
15607234		0	0	0.01786	76.21	2.39E-08	3	3011.6852	-1.2473	46.48889	1
15607234	M5(Oxidat	0	0	0.2053	75.28	4.74E-08	2	919.46532	-1.444	20.63077	0
15607234		0	0	0.03858	63.63	4.32E-07	3	2520.394	0.763685	43.98172	1
15607234		0	0	0.08468	62	1.42E-06	2	903.47099	-0.81512	24.33313	0
15607234		0	0	0.03022	61.75	1.24E-06	3	1848.934	-2.49722	26.60958	0
15607234		0	0	0.2237	60.9	1.75E-06	2	901.47173	-2.33398	21.28142	0

15607234		0	0	0.09789	59.05	1.74E-06	2	993.52458	-0.61153	23.92202	0
15607234	M5(Oxidat	0	0	0.1348	56.05	6.08E-06	3	2160.1073	-0.2747	26.13473	1
15607234		0	0	0.09772	55.5	3.1E-06	3	2791.521	0.311281	42.58352	2
15607234		0	0	0.3052	54.93	8.03E-06	2	971.55297	0.919949	26.15488	0
15607234	M8(Oxidat	0	0	0.1271	54.06	8.25E-06	4	2136.0585	-1.4531	23.37862	1
15607234		0	0	0.2512	51.89	1.55E-05	2	819.43163	-0.39553	12.93409	0
15607234		0	0	0.2942	51.49	1.31E-05	2	1227.6819	1.173809	24.6298	1
15607234	M5(Oxidat	0	0	0.2716	45.11	4.78E-05	3	2384.3001	1.416649	40.15316	1
15607234	N-Term(A	0	0	0.1866	30.32	0.001486	3	1906.9438	-0.14186	26.97361	0
15607234		0	0	0.3115	28.96	0.003494	3	1317.6902	-0.65426	25.02123	1
15607234		0	0	0.2274	24.76	0.006684	2	1313.7275	-0.78914	17.07382	1
15607317		0	0	0.1144	73.03	9.21E-08	2	2015.0834	1.265616	33.42058	1
15607317	M8(Oxidat	0	0	0.1935	69.47	3.22E-07	2	1272.6988	0.519972	22.40788	0
15607317	N-Term(A	0	0	0.1784	65.26	6.55E-07	2	1631.8508	0.434664	26.77303	0
15607317		0	0	0.2276	63.59	8.31E-07	3	3128.5235	1.73289	47.43148	0
15607317	M30(Oxid	0	0	0.2143	62.07	6.19E-07	4	3769.8429	3.045197	41.02542	1
15607317	M26(Oxid	0	0	0.1183	61.54	7E-07	4	3785.8346	2.182909	39.96786	1
15607317		0	0	0.3034	60.33	2.13E-06	2	1256.7023	-0.70279	29.2791	0
15607317		0	0	0.07763	59.85	1.45E-06	3	3753.8371	0.162506	43.4635	1
15607317	M12(Oxid	0	0	0.1153	57.13	4.07E-06	3	1799.958	-0.31353	29.71352	1
15607317	M20(Oxid	0	0	0.1446	55.29	4.44E-06	3	3144.513	0.002652	42.60554	0
15607317		0	0	0.2254	54.41	6.7E-06	2	1732.9133	0.920015	33.18016	0
15607317		0	0	0.2597	53.34	1.02E-05	2	1589.844	2.868482	23.36466	0
15607317		0	0	0.1885	52.01	1.35E-05	2	1783.9637	-0.01158	33.60315	1
15607317	M20(Oxid	0	0	0.3133	45.1	4.17E-05	3	3160.5044	-1.13072	42.2424	0
15607317	M20(Oxid	0	0	0.212	39.94	0.000122	3	3894.8965	1.569026	40.53421	1
15607317		0	0	0.3554	38.66	0.00032	3	2280.1507	-0.03613	34.26965	1
15607317		0	0	0.1772	37.66	0.000171	4	4504.2165	0.579473	40.87104	2
15607317		0	0	0.2253	36.78	0.000441	4	2215.1623	1.968637	23.44283	1
15607317		0	0	0.1697	35.01	0.000347	3	3878.904	2.215716	42.81894	1
15607317		0	0	0.3221	31.54	0.000737	2	866.47276	-0.3421	16.48914	0
15607317		0	0	0.266	24.93	0.00707	4	2233.1688	0.175826	24.31601	1
15607317		0	0	0.1962	22.95	0.006591	4	2460.3148	3.312073	23.90027	2
15607317		0	0	0.2641	21	0.011518	4	2858.4862	-0.22198	25.09406	2
15607317		0	0	0.2578	12.15	0.060809	4	4726.338	-2.6697	41.94914	2
15607341	M12(Oxid	0	0	5.41E-16	118.38	2.18E-12	3	3058.4894	-0.2136	30.87509	1

15607341		0	0	5.41E-16	98.91	1.28E-10	3	2720.5446	0.988227	40.47234	1
15607341		0	0	5.41E-16	97.38	4.2E-10	2	2684.3392	1.552571	35.14304	0
15607341		0	0	5.41E-16	94.81	6.94E-10	3	3042.4951	-0.00033	38.30741	1
15607341		0	0	5.41E-16	90.58	8.73E-10	3	2564.4426	0.709226	41.92978	0
15607341		0	0	3.67E-08	87.15	3.08E-09	2	1597.8709	0.717147	43.6329	0
15607341	M9(Oxidat	0	0	5.41E-16	86.91	3.97E-09	3	2700.331	0.415321	30.24596	0
15607341	N-Term(A	0	0	1.09E-06	83.21	7.4E-09	2	1639.8807	0.211316	41.98582	0
15607341		0	0	0.000213	68.27	2.46E-07	3	3574.7347	0.056795	40.04972	2
15607341		0	0	8.99E-15	66.72	3.72E-07	2	1526.7923	0.047568	20.48081	0
15607341	N-Term(A	0	0	2.15E-15	64.2	3.79E-07	3	2606.453	0.648691	46.61952	0
15607341		0	0	3.46E-11	59.58	1.38E-06	2	1416.6807	3.077872	24.01549	0
15607341	M4(Oxidat	0	0	3.16E-07	57.21	2E-06	2	1432.6724	0.798913	21.30045	0
15607341	N-Term(A	0	0	3.92E-11	52.74	1.2E-05	4	2726.3483	1.001709	44.06004	0
15607341	N-Term(A	0	0	1.32E-05	51.38	1.16E-05	3	2742.3438	1.230293	36.6945	0
15607341		0	0	0.001343	50.44	1.27E-05	2	1753.9718	0.557624	35.68109	1
15607341	M4(Oxidat	0	0	5.23E-05	40.9	8.11E-05	2	1448.668	1.266714	18.24836	0
15607341	N-Term(A	0	0	2.92E-07	37.42	0.000308	2	1568.801	-1.16355	19.50494	0
15607341		0	0	0.000132	31.3	0.001001	2	1867.0166	1.139712	20.99817	1
15607341	M16(Oxid	0	0	0.00109	29.46	0.001699	4	3590.7326	0.874014	33.32487	2
15607341		0	0	1.68E-06	23.45	0.004508	2	901.58373	0.837968	20.6416	1
15607347		0	0	0.000142	120.49	8.91E-13	3	3171.472	-0.22144	28.5406	1
15607347		0	0	0.000171	94.42	3.61E-10	2	2900.3465	0.631595	29.69615	0
15607347		0	0	0.000263	83.06	1.06E-08	2	2025.0987	0.532868	36.68947	0
15607347		0	0	0.03074	71.92	1.09E-07	2	2029.9881	0.060793	34.45768	0
15607347		0	0	0.02388	67.67	1.71E-07	2	1151.5351	-0.95483	14.49072	0
15607347		0	0	0.02058	67.47	3.04E-07	2	1395.7741	-0.2048	27.21187	0
15607347		0	0	0.06466	61.56	9.43E-07	3	2855.4236	0.380259	35.49968	1
15607347		0	0	0.01161	56.39	4.82E-06	3	1666.9034	0.541822	26.81066	1
15607347	N-Term(A	0	0	0.2546	46.96	3.73E-05	3	2072.0053	3.290489	38.1708	0
15607347		0	0	0.1762	36.93	0.000568	2	844.45281	0.538914	16.77363	0
15607347		0	0	0.2721	36.86	0.000206	2	883.4998	0.183541	20.74863	0
15607347	N-Term(A	0	0	0.1325	28.45	0.002715	3	2737.4619	-4.13124	40.5469	1
15607347		0	0	0.1837	28.3	0.004511	2	972.54686	-0.47271	19.91754	1
15607347		0	0	0.26	17.25	0.029197	2	2181.1993	0.249885	35.6276	1
15607424	C14(Carba	0	0	5.05E-16	96.57	2.2E-10	2	2437.0911	1.607142	27.27943	0
15607424		0	0	5.05E-16	91.93	1.86E-09	2	1911.9776	0.217242	40.65932	0

15607424		0	0	1.17E-11	84.32	5.18E-09	2	1461.6967	-0.14943	21.7238	0
15607424	N-Term(A	0	0	5.05E-16	83.32	1.07E-08	2	2371.2259	0.224094	40.81419	0
15607424		0	0	7.17E-14	83.05	1.09E-08	3	2485.3196	1.493945	34.49312	1
15607424	M1(Oxidat	0	0	4.84E-05	82.97	5.03E-09	2	2453.0921	4.067648	28.25989	0
15607424		0	0	5.05E-16	80.78	1.84E-08	2	2329.2161	0.57133	32.47422	0
15607424		0	0	5.05E-16	77.67	5.22E-08	2	1461.7601	1.105561	28.82243	0
15607424		0	0	5.05E-16	76.76	4.22E-08	2	1193.6641	0.283831	28.61867	0
15607424		0	0	1.26E-13	76.58	4.84E-08	3	3382.6717	0.385715	42.01711	0
15607424		0	0	2.34E-15	74.57	4.36E-08	2	1633.9025	0.25223	27.52971	0
15607424		0	0	1.2E-05	74.24	3.76E-08	2	880.48863	-0.09912	19.88697	0
15607424	N-Term(A	0	0	5.05E-16	73.77	8.61E-08	2	1953.9867	-0.50889	33.22731	0
15607424		0	0	2.38E-13	62.7	9.94E-07	2	1354.7725	2.610945	23.87368	1
15607424	N-Term(A	0	0	1.22E-14	62.47	1.1E-06	2	1675.9114	-0.74094	32.90946	0
15607424		0	0	2.57E-05	57.17	4.41E-06	3	1617.8573	-1.41445	26.82354	1
15607424		0	0	1.55E-07	54.21	5.31E-06	2	2609.3851	0.558011	39.61384	1
15607424	N-Term(A	0	0	7.04E-09	51.12	1.04E-05	2	1429.6799	-1.43328	21.39627	0
15607424		0	0	3.51E-06	49.79	1.47E-05	2	1387.6695	-1.34045	19.24455	0
15607424	N-Term(A	0	0	3.56E-06	42.37	8.69E-05	2	1235.6725	-1.45941	29.1169	0
15607424		0	0	3.46E-05	40.6	8.69E-05	2	1366.842	0.172108	22.65745	0
15607424	N-Term(A	0	0	3.15E-06	40.49	0.000197	3	2876.4664	0.458021	30.84573	0
15607424		0	0	3.68E-14	40.27	0.000183	3	2453.286	1.45258	41.84661	0
15607424		0	0	0.09942	37.79	0.000258	2	1092.5317	-0.36382	24.47976	0
15607424		0	0	9.32E-05	36.27	0.00059	3	2834.4561	0.574563	25.69323	0
15607424		0	0	6.86E-10	21.56	0.006966	5	4484.4566	1.877588	48.27348	0
15607453	M24(Oxid	0	0	5.49E-16	109.67	1.94E-11	3	3381.7203	-0.14971	37.45438	0
15607453		0	0	5.49E-16	99.49	2.25E-10	3	3365.7304	1.349058	39.13451	0
15607453		0	0	5.49E-16	94.84	4.1E-10	2	1804.8536	1.895956	49.62737	0
15607453	C12(Carba	0	0	5.49E-16	94.75	3.34E-10	2	1891.7393	0.95685	26.86827	0
15607453		0	0	5.49E-16	93.7	7.25E-10	3	3622.8746	-0.02605	39.43347	1
15607453	N-Term(A	0	0	5.49E-16	75.81	5.38E-08	2	2380.2593	0.169999	37.75288	0
15607453	C12(Carba	0	0	5.49E-16	72.6	5.48E-08	2	1907.732	-0.22493	25.38309	0
15607453	M26(Oxid	0	0	5.49E-16	68.55	2.3E-07	3	3638.8619	-2.11734	37.91493	1
15607453	C11(Carba	0	0	5.49E-16	65.58	4.15E-07	3	2338.2504	0.874613	36.90312	0
15607453	C11(Carba	0	0	1.25E-14	63.59	1.01E-06	3	2354.2359	-3.1417	43.84159	0
15607453	C28(Carba	0	0	5.49E-16	62.77	5.27E-07	3	3677.5634	-1.7255	34.53638	1
15607453	C28(Carba	0	0	1.03E-14	58.39	1.45E-06	3	3693.5705	1.575564	32.85303	1

15607453	C11(Carba	0	0	5.49E-16	54.25	6.77E-06	3	2737.4363	0.451924	32.3613		1
15607453	C11(Carba	0	0	4.58E-13	50.55	1.98E-05	4	2753.4293	-0.21366	28.91172		1
15607453	C11(Carba	0	0	2.87E-13	47.68	3.5E-05	4	3095.5934	-0.55266	31.26226		2
15607453		0	0	1.66E-10	46.88	3.59E-05	2	1434.7854	0.109208	24.64057		1
15607453	N-Term(A	0	0	2.31E-10	42.63	5.44E-05	2	1933.7513	1.658953	28.77112		0
15607453		0	0	1.35E-05	40.81	8.28E-05	2	867.49376	0.31087	17.81164		0
15607453		0	0	0.04424	35.1	0.001004	2	760.40972	-0.46782	15.12147		0
15607453	C17(Carba	0	0	0.09143	33.6	0.000589	3	3079.6435	0.955469	32.67607		1
15607453	N-Term(A	0	0	0.1887	33.48	0.001099	2	802.41997	-0.83102	24.42621		0
15607453	C17(Carba	0	0	0.000869	25.47	0.003547	5	3095.6364	0.297365	29.53143		1
15607453	N-Term(A	0	0	0.1887	15.07	0.031117	2	1846.865	2.345344	31.88042		0
15607479		0	0	0.004771	88.13	3.85E-09	2	1515.7861	1.587982	30.85575		0
15607479	C16(Carba	0	0	0.000555	85.93	2.55E-09	2	2153.003	1.253127	31.7404		0
15607479	C21(Carba	0	0	4.3E-07	84.42	3.61E-09	3	2802.3301	0.072432	30.06412		1
15607479	N-Term(A	0	0	1.05E-05	82.26	1.4E-08	2	1557.7966	1.502178	37.76307		0
15607479	M2(Oxidat	0	0	8.47E-05	80.46	8.97E-09	2	1533.6138	0.438722	24.65367		0
15607479		0	0	1.33E-05	78.63	1.85E-08	2	2164.0364	1.031984	43.53854		0
15607479	M13(Oxid	0	0	0.005614	77.66	3.51E-08	2	1531.7781	-0.2889	26.27233		0
15607479	C17(Carba	0	0	0.000129	67.4	3.91E-07	2	2430.2117	-0.23363	30.77754		0
15607479		0	0	0.000874	63.33	4.63E-07	2	1201.5334	-0.96419	18.3742		0
15607479		0	0	0.01051	60.43	1.49E-06	3	3496.7365	0.593148	48.17401		1
15607479		0	0	0.02358	59.08	1.24E-06	2	1105.5304	-0.23355	18.97982		0
15607479		0	0	0.000162	57.98	1.59E-06	3	2369.0412	0.389011	16.32354		0
15607479	C3(Carban	0	0	0.01597	57.65	1.71E-06	2	1042.4183	-1.06369	15.05768		0
15607479		0	0	0.002715	57.5	1.77E-06	2	1775.8335	-0.36468	17.53708		1
15607479		0	0	0.03345	56.26	2.72E-06	2	1012.6145	-0.52615	29.67148		0
15607479		0	0	0.000363	55.81	3.94E-06	3	3278.6605	0.718061	41.24972		0
15607479	M2(Oxidat	0	0	0.000911	52.3	5.87E-06	2	1791.8289	-0.11233	16.39837		1
15607479	M2(Oxidat	0	0	0.01336	40.57	0.000114	2	1121.5248	-0.703	17.26921		0
15607479	M3(Oxidat	0	0	0.06743	39.55	0.000111	2	1332.6152	-2.74378	18.92379		1
15607479		0	0	0.07057	37.38	0.000192	2	1316.6247	0.592491	22.5268		1
15607479	N-Term(A	0	0	0.01285	35.41	0.000287	2	1084.4311	1.054537	20.36538		0
15607479		0	0	0.02463	34.97	0.000621	2	859.50792	1.023648	30.48153		0
15607479	M1(Oxidat	0	0	0.04826	33.53	0.000443	2	1176.518	0.251279	22.41777		0
15607479		0	0	0.08106	31.54	0.001263	3	1995.0104	1.332739	26.59745		1
15607479	N-Term(A	0	0	0.004274	30.34	0.002266	3	2020.0732	0.404921	29.3583		0

15607479	N-Term(A	0	0	0.09313	29.23	0.001191	3	1817.8432	-0.83696	20.96644	1
15607479	M4(Oxidat	0	0	0.03855	26.66	0.002481	2	875.49999	-2.24984	25.57115	0
15607479	M1(Oxidat	0	0	0.08218	26.39	0.002291	3	1348.6118	-1.46246	17.16973	1
15607479	N-Term(A	0	0	0.0911	13.96	0.098439	3	3320.6869	5.467684	44.38759	0
15607503		0	0	0.01345	83.29	5.86E-09	2	2615.2569	-0.11386	37.65024	0
15607503		0	0	0.005924	82.57	1.19E-08	2	1740.9186	1.065759	42.64217	0
15607503		0	0	0.06163	81.62	1.03E-08	3	3982.9826	1.417991	42.08096	1
15607503		0	0	0.01445	76.25	5.1E-08	3	2312.1928	0.278052	45.04032	0
15607503		0	0	0.01661	66.45	5.55E-07	2	1386.7374	-0.21562	31.63003	0
15607503		0	0	0.02	66.31	3.63E-07	4	4446.2741	-2.05627	40.87973	2
15607503		0	0	0.1009	55.69	3.37E-06	2	1267.7638	1.388464	40.94766	0
15607503	C9(Carban	0	0	0.1213	55.18	5.61E-06	3	3793.886	0.926265	38.00215	0
15607503	N-Term(A	0	0	0.09747	54.65	5.31E-06	2	1152.6376	0.359646	30.81792	0
15607503		0	0	0.03767	54.33	5.53E-06	2	1110.6268	0.1038	23.51063	0
15607503	M8(Oxidat	0	0	0.05253	54.14	6.17E-06	3	2497.2152	-0.90789	28.26346	0
15607503	C9(Carban	0	0	0.1188	51.4	1.01E-05	3	3809.8733	-1.07515	36.39718	0
15607503		0	0	0.1441	50.49	2.5E-05	2	1028.5678	0.131564	36.68821	0
15607503		0	0	0.1493	50.35	9.2E-06	2	921.55144	-0.25186	18.88438	0
15607503	N-Term(A	0	0	0.1198	48.19	1.9E-05	2	1309.7724	-0.19838	48.3266	0
15607503		0	0	0.0225	46.94	5.16E-05	4	3247.6344	-2.26047	44.39501	0
15607503		0	0	0.1604	44.69	3.39E-05	2	1423.8633	0.089579	36.23044	1
15607503		0	0	0.06186	43	0.000153	3	1471.7847	-1.1545	22.24596	0
15607503	N-Term(A	0	0	0.1123	34.64	0.00079	2	1428.7519	2.563324	33.76834	0
15607503		0	0	0.09191	33.5	0.000446	2	1838.0953	1.061502	42.51619	1
15607503		0	0	0.1014	33.19	0.001079	3	2465.232	1.763518	35.12923	0
15607503	N-Term(A	0	0	0.1294	21.11	0.010455	4	3835.8957	0.688311	41.28431	0
15607503	C9(Carban	0	0	0.1775	19.3	0.020561	3	3825.8679	-1.14543	35.11738	0
15607503	M24(Oxid	0	0	0.3843	12.01	0.084983	4	4207.1471	9.158019	40.30105	1
15607503		0	0	0.3721	11.62	0.130844	4	4191.1173	0.873031	42.24882	1
15607553		0	0	5.17E-16	115.59	3.86E-12	3	3639.8075	0.045864	43.28705	1
15607553	N-Term(A	0	0	5.17E-16	108.46	2.42E-11	2	1773.9712	-1.89817	48.07084	0
15607553		0	0	5.17E-16	107.5	2.76E-11	2	1731.9644	0.208877	49.03442	0
15607553		0	0	7.66E-09	106.96	4.93E-11	2	1523.7701	-0.00274	25.30392	0
15607553		0	0	1.68E-09	105.99	6.55E-11	2	1645.8763	0.393105	35.75004	0
15607553	M17(Oxid	0	0	5.17E-16	97.51	3.55E-10	2	2765.3433	0.771339	31.84954	0
15607553		0	0	0.002931	95.34	6.14E-10	2	2749.3426	-1.3401	38.25077	0

15607553		0	0	5.17E-16	87.45	4.41E-09	2	2153.1355	0.234238	33.18029	0
15607553	N-Term(A	0	0	5.86E-10	87.23	4.83E-09	2	2195.1431	-1.13537	44.26368	0
15607553		0	0	4E-08	82.96	1.19E-08	2	2564.3353	-0.14375	38.54868	0
15607553		0	0	1.34E-14	82.57	1.27E-08	2	1792.9559	0.332884	46.27607	1
15607553	N-Term(A	0	0	9.04E-14	80.16	2.31E-08	2	2791.3685	4.166237	39.77114	0
15607553	N-Term(A	0	0	7.26E-07	71.72	1.45E-07	2	1834.9667	0.488349	34.43853	1
15607553		0	0	4.42E-11	68.36	2.19E-07	3	3419.8051	-2.30785	39.64555	2
15607553		0	0	2.09E-11	67.38	3.02E-07	3	2187.1875	-0.3055	39.99188	1
15607553	M21(Oxid	0	0	3.28E-12	64.22	4.54E-07	3	3220.5538	-3.56155	31.44517	1
15607553	N-Term(A	0	0	3.76E-09	56.66	6.37E-06	2	1687.8813	-2.91082	37.61891	0
15607553	N-Term(A	0	0	1.51E-06	54.17	9.38E-06	2	1565.7798	-0.51319	34.43595	0
15607553		0	0	0.005076	51.37	7.28E-06	2	909.47832	-0.61818	15.80115	0
15607553	N-Term(A	0	0	1.34E-08	39.24	0.000208	2	2807.3577	2.127379	33.94805	0
15607553		0	0	0.002913	28.08	0.002723	3	3204.5676	-0.86165	37.17375	1
15607553		0	0	0.01188	27.07	0.002945	2	1279.675	-0.31019	16.66474	1
15607572		0	0	0.001897	97.44	2.88E-10	2	1308.6548	0.398466	24.41657	0
15607572		0	0	0.002437	89.96	1.51E-09	3	1670.9167	-1.10788	30.10825	1
15607572	N-Term(A	0	0	0.1982	72.38	8.96E-08	2	1350.6641	-0.56728	26.77621	0
15607572		0	0	0.008351	70.99	1.79E-07	3	1763.9022	-0.78037	23.21074	1
15607572		0	0	0.01897	61.07	2.46E-06	2	929.5407	-0.87601	30.31675	0
15607572		0	0	0.02989	49.59	2.31E-05	2	760.39409	-0.98729	12.33868	0
15607572		0	0	0.1179	31.27	0.00112	2	1751.8674	0.100389	21.27611	1
15607638		0	0	0.000137	125.11	3.08E-13	2	1810.8322	0.353218	48.15344	0
15607638		0	0	0.003627	95.6	6.06E-10	2	2126.1453	0.009393	38.07154	1
15607638		0	0	0.006331	89.68	1.35E-09	3	3832.8838	1.542149	30.41105	1
15607638		0	0	0.02629	76.97	2.41E-08	3	2282.247	0.276977	36.71831	2
15607638		0	0	0.02248	76.91	2.24E-08	2	1099.6471	0.091871	33.40955	0
15607638		0	0	0.005446	76.08	6.66E-08	2	1970.044	-0.09055	39.90096	0
15607638		0	0	0.02355	74.16	6.14E-08	2	2589.2759	-1.2802	27.11381	0
15607638		0	0	0.004873	73.58	4.82E-08	3	1937.0952	1.268753	32.3378	1
15607638		0	0	0.008869	66.26	4.26E-07	3	2137.036	-1.04698	19.5138	1
15607638	N-Term(A	0	0	0.0262	63.45	8.13E-07	3	2631.2843	-2.07898	29.7574	0
15607638	N-Term(A	0	0	0.02072	61.19	1.33E-06	2	1141.6597	1.847545	38.67274	0
15607638		0	0	0.04155	59.92	1.02E-06	2	1499.6534	-0.68909	19.68939	0
15607638	M12(Oxid	0	0	0.0148	59.14	1.22E-06	3	4515.0685	2.50899	42.75134	0
15607638		0	0	0.006088	56.47	2.25E-06	3	4499.0709	1.930325	43.11242	0

15607638		0	0	0.08746	46.75	3.17E-05	2	1262.6165	0.040691	25.76808	0
15607638	N-Term(A	0	0	0.1557	44.58	4.88E-05	2	1304.6271	0.081628	29.82055	0
15607638		0	0	0.1294	44.41	7.06E-05	2	1629.8226	-0.38282	25.34402	0
15607638	N-Term(A	0	0	0.07798	41.58	0.000136	2	1671.8332	-0.34024	27.11606	0
15607638		0	0	0.1253	39.46	0.000113	2	1012.5641	-0.62991	13.11869	1
15607638	N-Term(A	0	0	0.0661	31.7	0.000676	2	898.47258	-1.75244	16.48071	0
15607638		0	0	0.09618	31.53	0.001301	2	1785.9252	0.51357	22.72059	1
15607638		0	0	0.1852	27.65	0.00292	2	855.44542	2.618325	18.76734	1
15607638	N-Term(A	0	0	0.2259	20.79	0.00917	6	4140.9772	1.248586	33.25235	1
15607816	M1(Oxidat	0	0	4.26E-16	128.57	1.39E-13	2	2078.9476	-0.32658	29.84228	0
15607816		0	0	5.08E-13	89.3	1.17E-09	2	2062.9539	0.282954	31.74773	0
15607816	M7(Oxidat	0	0	7.79E-05	85.1	3.08E-09	2	1818.7824	0.203395	25.59455	0
15607816	M1(Oxidat	0	0	7.04E-10	84.98	3.17E-09	2	2094.9476	2.103186	25.53748	0
15607816		0	0	1.81E-07	83.59	4.81E-09	2	1934.9233	-0.56639	28.57351	0
15607816	M1(Oxidat	0	0	9.5E-13	81.58	6.93E-09	2	1834.7798	1.575914	23.47538	0
15607816		0	0	4.37E-13	79.68	1.07E-08	2	1802.7864	-0.38094	29.40068	0
15607816		0	0	1.74E-13	73.09	8.35E-08	2	1828.8987	-1.31886	27.28505	0
15607816		0	0	1.6E-13	71.69	9.49E-08	2	1525.7748	-0.66421	29.58582	0
15607816	M10(Oxid	0	0	1.14E-05	63.8	7.3E-07	2	1844.8993	1.779659	23.41151	0
15607816		0	0	1.04E-13	62.63	5.44E-07	3	2698.2398	-3.52491	28.09949	1
15607816	M1(Oxidat	0	0	0.001696	60.46	8.97E-07	2	1850.7726	0.418369	21.33746	0
15607816		0	0	1.95E-10	59.52	1.73E-06	2	1120.5995	-0.19311	28.3879	0
15607816		0	0	1.25E-11	59.04	1.56E-06	2	1062.5944	0.11919	29.40312	0
15607816		0	0	4.49E-09	45.86	5.19E-05	2	1762.8648	-0.71906	34.88412	0
15607826		0	0	0.004036	120.36	1.56E-12	2	1773.9712	0.365116	37.20862	0
15607826		0	0	0.00291	88.08	2.33E-09	2	1582.7815	-0.32826	17.74376	0
15607826	N-Term(A	0	0	0.01547	79.85	1.71E-08	2	1815.9797	-0.82294	42.82296	0
15607826		0	0	0.01822	78.2	2.5E-08	2	2157.168	-1.08242	40.07973	0
15607826		0	0	0.006259	76.06	6.32E-08	2	1990.9941	-2.07693	29.27646	0
15607826		0	0	0.009337	71.99	1.64E-07	3	2009.9981	-0.93494	17.36579	1
15607826		0	0	0.02291	71.64	6.85E-08	2	1296.6066	-0.05886	17.64029	0
15607826		0	0	0.02976	61.79	9.93E-07	2	1452.7077	-0.08405	17.09095	1
15607826		0	0	0.06863	61.16	7.64E-07	2	1470.8741	-0.74124	26.7575	1
15607826	N-Term(A	0	0	0.03635	60.27	1.74E-06	2	1624.7917	-0.58637	20.82336	0
15607826		0	0	0.01438	58.5	2.47E-06	2	930.52544	-0.08928	25.15304	0
15607826		0	0	0.2737	46.97	4.42E-05	2	773.43993	-0.58612	29.55865	0

15607826	N-Term(A	0	0	0.1534	33.4	0.00048	2	1125.6481	-0.57515	20.67767	1
15607826	M1(Oxidat	0	0	0.2554	33.25	0.001136	2	1287.6845	0.49666	20.39746	1
15607826		0	0	0.3125	31.7	0.001859	2	1271.6897	0.631887	22.53408	1
15607826		0	0	0.2225	24.36	0.01136	2	929.54082	-0.73393	26.36054	1
15607956	C6(Carban	0	0	4.95E-16	105.44	3.29E-11	3	2793.3085	-0.88049	45.15551	0
15607956		0	0	4.95E-16	89.83	1.98E-09	2	1711.9139	0.692008	29.35642	0
15607956	N-Term(A	0	0	4.95E-16	88.32	2.65E-09	2	1753.9252	1.124448	33.3487	0
15607956	C7(Carban	0	0	4.95E-16	88.13	2.08E-09	4	3105.5082	-1.61194	48.16564	2
15607956		0	0	4.95E-16	85.37	5.08E-09	2	1880.0175	0.5438	47.05702	0
15607956	C7(Carban	0	0	4.95E-16	72.37	8.4E-08	3	2949.4136	0.516398	47.47549	1
15607956		0	0	4.95E-16	67.31	2.42E-07	3	2036.1198	1.072544	49.08601	1
15607956	C6(Carban	0	0	4.95E-16	67.03	2.87E-07	3	2949.4085	-1.2219	39.33495	1
15607956		0	0	4.95E-16	64.84	3.77E-07	4	2320.2777	0.18505	40.56905	2
15607956	C21(Carba	0	0	4.95E-16	56.05	4.47E-06	2	2696.3099	2.430267	28.18406	1
15607956		0	0	4.33E-13	49.22	3.05E-05	2	1114.6203	-1.14339	18.25895	1
15607956		0	0	4.5E-10	48.66	2.52E-05	2	1295.6587	-0.26419	18.84985	0
15607956		0	0	1.82E-11	40.91	0.000118	3	2164.1757	-0.22548	42.30079	1
15607956		0	0	2.79E-06	40.19	0.000144	2	812.52379	-0.33356	26.51008	0
15607956	C4(Carban	0	0	6.05E-12	39.77	0.000105	3	1258.5788	0.628265	14.31262	1
15607956	C4(Carban	0	0	2.48E-08	35.22	0.0003	2	1003.4077	-0.76528	13.14319	0
15607956		0	0	4.03E-15	32.31	0.001175	3	2988.5528	-0.38573	30.18801	1
15607956	C21(Carba	0	0	0.04976	25.78	0.00436	3	2951.4701	-0.94306	27.85096	2
15607956	N-Term(A	0	0	0.04222	21.02	0.018976	2	1337.6703	0.515362	20.36197	0
15608041		0	0	0.03958	74.16	5.95E-08	2	1342.676	0.655124	27.66711	0
15608041		0	0	0.03942	58.99	4.23E-06	2	1543.8262	-0.37687	29.20482	0
15608041		0	0	0.06082	49.66	2.16E-05	3	2898.4879	1.182648	29.82767	0
15608041		0	0	0.2378	32.31	0.000705	2	924.47246	-0.25051	33.03039	0
15608109		0	0	0.04586	78.94	3.19E-08	3	2334.221	0.351345	30.33104	0
15608109	C2(Carban	0	0	0.1998	60.06	1.68E-06	2	1336.6668	-0.80024	28.77648	0
15608109		0	0	0.1557	54.19	5.72E-06	2	1597.7831	0.042154	19.82211	1
15608109		0	0	0.4266	28.3	0.003328	3	1287.7168	-0.02351	22.11539	0
15608109		0	0	0.1971	23.67	0.004285	2	1188.5418	2.115488	18.85239	0
15608122		0	0	0.000232	126.77	4E-13	3	2879.4854	1.891119	45.31182	2
15608122	M7(Oxidat	0	0	0.01078	106.2	4.56E-11	2	1689.8321	1.253112	25.8282	0
15608122		0	0	0.02106	94.8	8.94E-10	2	1739.892	-0.18158	26.13183	0
15608122		0	0	0.02302	86.29	3.76E-09	2	2282.1687	1.024232	34.65073	0

15608122		0	0	0.0102	79.18	2.42E-08	2	2567.2776	-0.04901	49.72286	0
15608122		0	0	0.008731	76.09	5.78E-08	2	1657.8403	0.076159	35.11969	0
15608122		0	0	0.009143	76.08	4.69E-08	2	1434.7184	0.041881	19.61858	0
15608122		0	0	0.006046	75.49	5.23E-08	3	2673.3957	0.023466	27.92509	1
15608122		0	0	0.006483	73.97	1.5E-07	3	2723.3789	-0.00071	47.25294	1
15608122		0	0	0.007699	73.5	4.46E-08	2	2009.9197	-1.83886	27.60543	0
15608122		0	0	0.02304	72.67	7.03E-08	2	1207.6521	1.429143	36.5544	0
15608122		0	0	0.01829	69.24	2.14E-07	3	1900.9777	0.670174	32.02234	0
15608122	M13(Oxid	0	0	0.05363	67.72	1.69E-07	2	2025.9227	2.13173	22.61738	0
15608122		0	0	0.07155	67.64	2.41E-07	2	1110.5903	0.005636	19.30541	0
15608122	M7(Oxidat	0	0	0.03083	64.68	7.32E-07	3	1673.835	-0.03059	29.22314	0
15608122		0	0	0.03526	58.89	1.81E-06	2	2337.1287	0.397243	22.60154	1
15608122		0	0	0.1214	58.84	2.81E-06	2	1148.5966	0.51708	21.09952	0
15608122	M8(Oxidat	0	0	0.04388	57.14	3.77E-06	2	1223.6458	0.378583	31.42216	0
15608122		0	0	0.09451	55.62	2.74E-06	2	1195.7417	0.656929	32.2763	0
15608122		0	0	0.02664	54.41	3.61E-06	3	2382.4321	-1.00287	41.87458	1
15608122	M2(Oxidat	0	0	0.06218	52.49	7.33E-06	3	1551.8428	0.19415	23.54398	1
15608122		0	0	0.04422	51.19	1.63E-05	3	2438.269	0.608951	32.28985	1
15608122		0	0	0.06401	50.17	1.78E-05	3	1465.7868	-0.17974	19.71184	1
15608122	M6(Oxidat	0	0	0.09493	47.88	1.79E-05	2	1207.5801	-0.75405	24.08827	0
15608122		0	0	0.08751	45.05	3.44E-05	2	1535.8486	0.629646	27.5201	1
15608122	N-Term(A	0	0	0.2047	44.64	6.18E-05	2	1942.9852	-0.94251	35.75165	0
15608122		0	0	0.2334	44.19	8.76E-05	2	1606.8028	-0.2423	21.9055	1
15608122		0	0	0.05325	43.13	0.000119	3	1379.7473	0.591609	29.4557	0
15608122		0	0	0.4854	42.88	0.000149	2	789.40984	-0.39734	13.00851	0
15608122		0	0	0.1342	42.22	0.000111	4	2227.1806	-1.13085	30.76061	1
15608122		0	0	0.2267	41.41	0.000192	2	787.46654	-0.91687	19.62087	0
15608122	M6(Oxidat	0	0	0.1933	31.79	0.001622	2	1244.6675	0.579888	19.797	1
15608122		0	0	0.3449	31.25	0.000748	3	1205.7123	0.782215	23.61164	0
15608122	M1(Oxidat	0	0	0.3733	29.14	0.001585	2	1395.7399	-1.05359	25.39028	0
15608122		0	0	0.1559	28.94	0.002042	2	952.52098	-0.13505	19.03981	0
15608122		0	0	0.4026	18.91	0.016066	3	1879.0395	-0.11093	27.9741	1
15608255		0	0	0.0456	90.9	1.46E-09	2	1363.6615	1.030551	28.04891	0
15608255		0	0	0.004253	67.97	3.35E-07	2	933.47832	-0.62371	15.99471	0
15608255		0	0	0.007555	65.6	2.75E-07	2	1079.6225	1.540189	32.60028	1
15608255	M2(Oxidat	0	0	0.06188	55.05	7.82E-06	2	2452.2466	-0.43661	44.3838	0

15608255		0	0	0.1447	37.11	0.000194	2	923.52037	0.726574	33.24095	0
15608255		0	0	0.1447	32.33	0.000643	4	3841.9025	0.475084	39.22967	0
15608255	M4(Oxidat	0	0	0.07042	25.06	0.005614	3	2721.4341	0.450455	45.42835	1
15608272	M8(Oxidat	0	0	5.42E-16	90.6	2E-09	2	1542.8328	1.215278	30.94891	0
15608272		0	0	5.42E-16	88.27	1.49E-09	2	1260.7426	0.206235	27	0
15608272		0	0	5.42E-16	85.51	4.36E-09	2	1207.5953	-0.12327	26.20841	0
15608272	M2(Oxidat	0	0	5.42E-16	85.39	2.88E-09	2	1909.787	0.041158	23.25005	0
15608272		0	0	5.42E-16	72.46	1.11E-07	2	1526.8384	1.575285	34.93653	0
15608272		0	0	5.42E-16	71.81	6.58E-08	2	1893.7914	-0.32309	23.75763	0
15608272		0	0	5.42E-16	59.42	1.77E-06	2	1429.7295	-0.12696	39.07327	0
15608272	C2(Carban	0	0	5.42E-16	59.07	1.24E-06	3	3055.4291	0.929821	32.49651	0
15608272		0	0	5.42E-16	57.03	2.38E-06	2	908.50212	-0.18548	35.6783	0
15608272	C1(Carban	0	0	5.42E-16	55.93	2.55E-06	2	1049.4837	0.328616	22.92135	0
15608272	M8(Oxidat	0	0	1.75E-15	55.62	3.29E-06	2	1052.594	1.772231	27.43508	1
15608272	C2(Carban	0	0	0.1179	53.84	4.12E-06	3	3039.4149	-5.41722	35.75398	0
15608272		0	0	3.53E-09	52.62	1.67E-05	2	928.536	-0.38124	28.66019	0
15608272	C1(Carban	0	0	5.42E-16	48.96	2.86E-05	4	2291.204	-1.6782	36.79632	1
15608272	M5(Oxidat	0	0	2.32E-10	46.76	3.48E-05	2	1445.7259	0.858653	37.09165	0
15608272	M4(Oxidat	0	0	0.003831	42.03	8.46E-05	2	2375.1416	-1.40503	34.48662	1
15608272		0	0	5.42E-16	36.32	0.00028	3	2012.9679	0.317124	28.64394	1
15608272	C19(Carba	0	0	5.42E-16	36.21	0.000239	5	4914.2048	-0.1453	37.62121	1
15608272		0	0	3.66E-15	35.57	0.000541	2	1682.8505	0.457656	28.20593	1
15608272	M2(Oxidat	0	0	5.42E-16	32.47	0.000565	5	4930.2052	0.960843	37.46495	1
15608272	N-Term(A	0	0	5.42E-16	30.73	0.001141	2	1302.7535	0.429296	37.86877	0
15608272		0	0	1.48E-10	29.85	0.001967	3	2359.1506	0.227447	35.51491	1
15608272		0	0	1.63E-13	22.28	0.009169	4	2488.2189	-1.09658	35.1052	2
15608458		0	0	5.55E-16	106.33	3.49E-11	2	1659.8173	-0.72475	25.43297	0
15608458		0	0	1.91E-10	103.09	1.33E-10	2	1790.9294	0.545855	36.8848	0
15608458	C8(Carban	0	0	1.68E-09	80.93	2.74E-08	2	2681.357	2.624004	34.36428	0
15608458	C13(Carba	0	0	3.06E-09	75.53	5.32E-08	4	3207.6369	0.167172	35.69893	1
15608458	M5(Oxidat	0	0	0.002836	74.82	9.06E-08	2	2697.3421	-1.02756	32.63732	0
15608458	M5(Oxidat	0	0	3.41E-09	74.28	7.28E-08	2	1051.5352	-0.18302	27.10611	0
15608458	N-Term(A	0	0	4.38E-05	66.52	5.35E-07	2	1093.5462	0.209298	31.16914	0
15608458	M10(Oxid	0	0	4.69E-06	66.37	4.5E-07	3	3223.6307	-0.16437	34.9216	1
15608458		0	0	0.00016	65.85	4.16E-07	2	1979.9749	-3.69711	29.74624	1
15608458		0	0	0.000329	61.31	1.52E-06	2	1035.5416	1.033474	32.62378	0

15608458		0	0	0.001159	56.65	5.41E-06	2	1265.6706	-0.3172	17.05501		1
15608458		0	0	2.43E-07	50.87	1.51E-05	2	1694.8401	0.964214	31.07483		0
15608458		0	0	0.01761	48.97	3.17E-05	3	1886.9558	-0.48046	25.21672		1
15608458		0	0	0.02222	39.93	0.000224	3	1550.8132	-0.9645	18.06279		2
15608458	C3(Carban	0	0	0.1132	37.7	0.00034	2	1411.7385	1.526925	29.08555		1
15608703		0	0	0.01063	78.28	2.01E-08	3	2302.2317	0.596076	41.75042		0
15608703		0	0	0.04058	75.12	5.54E-08	2	1175.6392	1.077248	19.25291		1
15608703		0	0	0.08063	67.51	4.35E-07	2	1119.6164	0.579937	25.86171		0
15608703		0	0	0.121	58.92	2.89E-06	2	1032.5472	-0.14969	27.79815		0
15608703	C4(Carban	0	0	0.06239	56.33	4.89E-06	3	1752.8721	1.305951	26.46827		1
15608703		0	0	0.02832	56.1	5.15E-06	3	2458.226	-1.47422	28.04097		1
15608703	M20(Oxida	0	0	0.06066	52.19	1.51E-05	3	2474.2254	0.343818	26.98166		1
15608703	C14(Carba	0	0	0.1586	50.74	1.64E-05	3	1775.9145	-2.20305	37.86191		0
15608703		0	0	0.0656	47.79	3.66E-05	3	1614.8968	0.32349	31.21502		1
15608703		0	0	0.3101	44.15	9.23E-05	2	894.43456	-0.4857	19.41619		0
15608703		0	0	0.2339	43.5	4.46E-05	2	1104.5114	0.424475	36.80787		0
15608703		0	0	0.2726	42.12	0.000178	2	1100.6246	0.01637	25.14325		0
15608703	N-Term(A	0	0	0.3398	37.35	0.000184	2	865.48882	-0.2879	26.82447		0
15608703	M2(Oxidat	0	0	0.3201	36.58	0.000219	2	910.42912	-0.85844	15.80159		0
15608703		0	0	0.1125	35.61	0.000357	3	1830.0284	2.761249	31.78286		2
15608703	M10(Oxida	0	0	0.2951	31.39	0.000724	3	1373.6257	-0.72127	18.23076		0
15608703	M1(Oxidat	0	0	0.3946	30.26	0.001554	2	1116.6181	-1.22397	22.38665		0
15608703		0	0	0.3829	28.71	0.002153	2	816.49443	0.7484	18.92899		1
15608703		0	0	0.3948	27.72	0.001686	2	1357.6314	-0.28435	20.75317		0
15608703		0	0	0.3948	27.26	0.001875	2	1147.6938	-0.77383	25.17719		1
15608759		0	0	4.95E-16	112.18	1.27E-11	2	1986.03	0.384448	32.3093		1
15608759	C2(Carban	0	0	4.95E-16	102.94	8.64E-11	2	1675.8111	0.08799	31.89814		0
15608759	C16(Carba	0	0	4.95E-16	96.1	5.03E-10	3	3246.6573	-0.13354	42.74052		1
15608759		0	0	4.95E-16	91.56	6.97E-10	2	1093.6481	0.372686	49.75799		0
15608759		0	0	4.95E-16	88.26	2.39E-09	3	3462.8697	-0.23022	40.70986		1
15608759		0	0	4.95E-16	82.31	5.86E-09	2	1417.6961	0.17015	31.44982		0
15608759		0	0	4.95E-16	82.23	1.41E-08	2	1589.8649	0.160053	35.00379		0
15608759		0	0	4.95E-16	79.69	1.77E-08	2	1227.6219	-0.88588	40.31782		0
15608759		0	0	4.95E-16	78.56	2.16E-08	2	2130.1563	0.3926	38.39247		0
15608759		0	0	4.95E-16	78.32	3.61E-08	2	1630.8772	0.793171	32.93558		0
15608759		0	0	4.95E-16	77.28	4.77E-08	2	1499.7854	0.064469	32.21224		0

15608759		0	0	4.95E-16	72.26	1.37E-07	2	1940.0386	-0.17499	41.89351	0
15608759		0	0	1.3E-12	67.18	8.23E-07	2	943.5407	9.205446	44.25286	0
15608759		0	0	4.95E-16	67.18	5.07E-07	3	2174.1059	-0.16199	23.57089	2
15608759		0	0	4.95E-16	67.03	4.36E-07	3	2142.13	-0.15559	31.03926	2
15608759		0	0	4.15E-12	63.67	5.37E-07	2	1121.5566	-1.83241	22.12639	0
15608759		0	0	5.07E-16	62.61	8.5E-07	2	1745.8418	-2.03159	28.3432	1
15608759	C2(Carban	0	0	7.18E-14	60.23	1.94E-06	3	2487.2642	-0.80047	41.2258	1
15608759		0	0	6.32E-13	59.99	1E-06	2	1320.7848	-0.84318	22.74144	1
15608759		0	0	3.25E-11	59.8	1.94E-06	2	1351.7317	-0.8613	25.95244	0
15608759	N-Term(A	0	0	1.13E-12	59.23	1.85E-06	2	1717.8233	1.041717	44.40797	0
15608759		0	0	3.76E-06	57.86	3.27E-06	3	1655.8857	-0.45655	30.23737	1
15608759		0	0	7.77E-09	54.98	4.92E-06	2	1990.0889	-2.6514	23.23703	1
15608759		0	0	4.26E-14	52.84	8.84E-06	2	1958.9666	-0.70495	27.95671	2
15608759		0	0	0.001676	51.81	2.04E-05	2	830.47331	0.268402	26.73512	0
15608759	N-Term(A	0	0	9.02E-07	51.63	1.58E-05	2	985.54228	-0.29666	28.77391	0
15608759		0	0	1.07E-07	42.74	8.51E-05	3	2144.1353	0.29746	33.72216	1
15608759		0	0	1.87E-09	40.74	0.00019	4	2472.287	1.115045	33.48218	2
15608759		0	0	6.26E-06	40.14	0.000169	3	2018.0086	1.753615	25.13761	1
15608759		0	0	0.0112	38.98	0.000291	3	1713.8646	-1.28608	20.41448	1
15608759		0	0	0.000175	35.92	0.000255	3	1946.1341	0.280585	48.23539	0
15608759		0	0.003	0.07599	28.55	0.003491	2	1277.6596	-0.11568	20.62957	1
15608759		0	0.006	0.209	24.7	0.00338	2	872.52971	-1.78861	19.905	1
15608773		0	0	0.1118	113.98	1E-11	2	2209.127	-0.57062	29.11841	0
15608773	M2(Oxidat	0	0	0.09312	102.88	1.06E-10	2	2225.1211	-0.91454	28.22213	0
15608773	N-Term(A	0	0	0.07436	42.53	0.000131	3	2267.1441	4.585882	27.64659	0
15608773		0	0	0.263	28.52	0.002461	3	1666.8343	5.202918	13.70566	2
15608773		0	0	0.3309	23.89	0.010208	4	2496.2741	1.677403	31.38782	1
15608874		0	0	0.03949	72.16	1.13E-07	2	1694.8494	-0.2056	36.038	0
15608874		0	0	0.04959	70.51	1.33E-07	2	1664.8793	2.251671	35.18739	1
15608874		0	0	0.1275	68.55	2.65E-07	2	1364.7216	0.479157	38.4364	0
15608874		0	0	0.1211	60.57	8.75E-07	2	1506.7013	0.066532	34.55754	0
15608874		0	0	0.1308	55.07	6.38E-06	3	1850.9502	-0.38514	33.40822	1
15608874		0	0	0.2149	48.06	3.6E-05	2	843.49285	-0.76568	25.87205	0
15608874		0	0	0.26	38.6	0.000138	2	871.39354	-1.09026	23.97988	0
15608874		0	0	0.06592	35.12	0.000538	3	1921.9896	0.830307	34.02699	1
15608874		0	0	0.2402	25.47	0.002831	2	1141.5379	-0.57984	20.93801	1

15608874		0	0	0.1259	17.69	0.02383	3	1821.986	0.079455	38.84824	1
15608874		0	0	0.207	14.73	0.067302	3	1297.6769	0.617796	17.02079	2
15608881		0	0	0.04264	124.47	9.11E-13	2	1922.9831	0.623264	29.51861	1
15608881		0	0	0.08506	94.25	7.33E-10	3	1614.8489	0.098319	27.17225	0
15608881		0	0	0.1162	92.71	5.35E-10	2	1355.6324	-0.14715	22.49887	0
15608881		0	0	0.126	79.26	2.02E-08	2	2338.1199	-0.51779	31.74311	1
15608881	C7(Carban	0	0	0.09834	76.16	2.42E-08	2	1550.7309	2.609905	21.43206	0
15608881		0	0	0.1379	62.19	1.06E-06	2	1716.8349	0.486457	32.20596	0
15608881		0	0	0.4155	37.19	0.000191	3	2684.2331	2.49461	42.5266	0
15608881	C25(Carba	0	0	0.2206	35.43	0.000415	4	3578.7653	-0.19554	43.6694	0
15608881		0	0	0.1294	30.29	0.001777	2	2099.0862	-0.89661	24.54856	1
15608881	M24(Oxid	0	0	0.3703	23.6	0.004355	3	3875.7953	-0.48896	41.81006	0
15608934	C9(Carban	0	0	0.02949	88.97	2.98E-09	2	1746.8685	-0.40615	28.75004	0
15608934		0	0	0.01314	85.51	4.64E-09	3	2890.4355	0.836792	38.47857	1
15608934		0	0	0.01286	85.19	7.11E-09	2	1333.696	0.089338	34.13702	0
15608934		0	0	0.1643	80.13	4.8E-08	2	1142.6318	0.0264	31.99028	0
15608934	C14(Carba	0	0	0.1578	72.66	5.41E-08	2	2326.0508	-0.41549	27.75706	0
15608934		0	0	0.07429	66.86	4.84E-07	2	2507.2112	2.491988	40.46983	0
15608934	C7(Carban	0	0	0.04065	65.9	5.91E-07	2	2175.1197	0.423519	32.86598	1
15608934		0	0	0.1181	55.57	4.85E-06	3	1939.0636	-0.61809	44.94598	0
15608934	C10(Carba	0	0	0.1045	51.43	7.55E-06	4	4524.2072	-0.67222	43.09333	0
15608934		0	0	0.2743	35	0.000522	2	787.48186	-0.84088	33.39763	0
15608934		0	0	0.2347	24.46	0.005192	3	1162.6441	0.039411	18.11949	2
57116687	C2(Carban	0	0	0.01423	109	1.26E-11	2	1784.7175	-0.37007	25.90719	0
57116687	M27(Oxid	0	0	0.02541	97.02	3.38E-10	3	3096.6635	2.683868	40.68172	0
57116687		0	0	0.04847	83.1	7.1E-09	2	1428.6847	-1.35882	22.48309	0
57116687		0	0	0.03891	80.36	2.07E-08	2	2258.1724	-1.2047	36.46444	0
57116687	M11(Oxid	0	0	0.006755	77.27	1.87E-08	3	4039.8563	-3.4714	33.63462	1
57116687		0	0	0.03056	72.42	8.88E-08	3	2348.1661	1.307196	29.56254	1
57116687		0	0	0.05476	67.52	2.66E-07	2	1105.5447	-1.11797	27.55142	0
57116687	M7(Oxidat	0	0	0.04079	66.69	2.68E-07	2	1121.5402	-0.59522	23.61353	0
57116687	M11(Oxid	0	0	0.03314	63.71	8.72E-07	3	2274.1719	0.819134	33.41358	0
57116687	C14(Carba	0	0	0.1126	62.45	5.68E-07	3	1950.8904	1.429968	29.07338	0
57116687		0	0	0.07278	54.13	3.85E-06	2	938.49388	-0.33911	22.20544	0
57116687		0	0	0.2172	44.46	6.8E-05	2	829.4525	-0.22917	16.94587	0
57116687	C9(Carban	0	0	0.2801	42.52	5.58E-05	2	1886.8531	0.407897	13.86335	1

57116687		0	0	0.2131	40.74	0.000169	3	1915.9847	2.005232	35.25929	1
57116687		0	0	0.1036	40.11	0.000175	3	3754.8431	-0.80657	36.9623	0
57116687	N-Term(A	0	0	0.2898	33.89	0.000449	2	871.46306	-0.22492	21.72729	0
57116687		0	0	0.3013	33.65	0.0011	2	1062.5682	-0.90203	24.55843	1
57116687		0	0	0.2855	29.46	0.002548	2	1056.5898	-1.09978	16.65972	1
57116894	M1(Oxidat	0	0	0.001012	97.23	1.89E-10	2	2197.9913	-0.27651	28.92445	0
57116894		0	0	0.001031	93.26	7.32E-10	2	1745.8285	0.153228	41.39913	0
57116894		0	0	9.54E-05	92.02	1.7E-09	2	1685.8907	0.884576	37.74564	0
57116894		0	0	0.001795	88.86	1.3E-09	2	2181.9979	0.412023	28.88079	0
57116894		0	0	0.007624	86.83	2.7E-09	2	1438.8398	1.416404	34.71522	0
57116894	M8(Oxidat	0	0	0.004942	84.05	1.28E-08	2	1701.8843	0.134347	31.67612	0
57116894	M5(Oxidat	0	0	0.006976	80.56	1.19E-08	3	2956.4095	-1.5398	30.70959	1
57116894		0	0	0.00376	75.11	6.17E-08	2	1087.5738	-0.45352	21.62623	0
57116894		0	0	0.000316	73.06	1.09E-07	2	1863.9125	0.070757	34.269	0
57116894		0	0	0.04893	72.56	7.76E-08	2	2924.4227	-0.54242	36.64054	1
57116894		0	0	0.000541	68.46	2.78E-07	2	1901.9324	1.592772	36.82835	1
57116894		0	0	0.03228	60.08	2.36E-06	3	2586.314	2.036758	48.22348	1
57116894	M5(Oxidat	0	0	0.04681	59.72	1.55E-06	3	2940.422	0.977724	35.34307	1
57116894		0	0	0.03562	55.94	2.54E-06	3	2172.2607	-0.26056	33.72243	1
57116894		0	0	0.02454	54.16	3.83E-06	2	1257.5528	-0.09489	16.49157	0
57116894		0	0	0.07112	53.49	1.03E-05	3	2395.1947	0.745242	41.99849	1
57116894		0	0	0.0106	47.91	1.61E-05	2	1005.5734	0.597228	29.30753	1
57116894	M5(Oxidat	0	0	0.1088	47.47	1.79E-05	2	1273.5475	-0.22249	14.78535	0
57116894		0	0	0.1182	36.91	0.000204	2	913.47399	0.168901	16.33181	0
57116894		0	0	0.02856	35.34	0.000409	2	1290.7165	-0.04109	24.19876	2
57116894	N-Term(A	0	0	0.32	35.07	0.000373	3	2982.4197	-3.3766	30.34776	1
57116894	N-Term(A	0	0	0.2578	32.17	0.001031	2	1129.5828	-1.79272	23.11802	0
57116894		0	0	0.1832	30.03	0.001092	2	908.49919	0.332203	34.64719	0
57116894		0	0	0.06696	28.28	0.00208	2	1036.5958	1.82272	28.009	1
57116894		0	0	0.3437	25.59	0.005383	2	1423.7293	0.269957	18.09495	1
57116894	M17(Oxid	0	0	0.2711	21.51	0.007046	4	4080.904	0.131116	38.48539	2
57116894		0	0	0.2308	17.57	0.020123	3	1594.9383	-0.32835	29.70804	1
15608956		0	0	3.02E-14	108.02	3.08E-11	2	1749.9039	0.391389	32.04502	0
15608956		0	0	0.02858	65.91	3.21E-07	2	859.49993	0.33069	21.70202	0
15608956		0	0	0.003703	64.51	3.53E-07	2	1003.5898	0.247091	22.7992	1
15608956		0	0	2.85E-08	61.45	1.61E-06	3	2538.312	2.861562	32.75543	0

15608956	C6(Carban	0	0	0.01383	58.84	2.81E-06	3	2178.1364	1.249413	31.38755	0
15608956		0	0	0.06424	58.76	1.33E-06	2	1204.5378	-0.36028	29.84585	0
15608956		0	0	0.003101	58.45	2.29E-06	2	1243.7122	0.341318	21.73703	1
15608956	C4(Carban	0	0	0.002023	57.88	4.56E-06	2	2491.2471	-1.08676	32.57422	1
15608956	M23(Oxid	0	0	4.21E-05	52.28	1.42E-05	3	4117.092	1.621978	42.87875	0
15608956	C4(Carban	0	0	1.16E-08	51.61	1.04E-05	2	1932.9468	-0.8538	30.78991	0
15608956		0	0	0.00021	47.72	3.3E-05	2	1308.6994	-1.9507	17.44585	1
15608956		0	0	0.000164	46.15	5.58E-05	3	1537.8341	0.500247	30.39483	1
15608956		0	0	4.43E-05	43.17	9.64E-05	3	4101.0846	-1.42729	43.94465	0
15608956		0	0	0.0106	39.43	0.00016	2	1066.5773	-0.61242	28.36572	0
15608956		0	0	1.41E-05	30.32	0.001579	4	3135.589	-0.89978	32.23417	1
15608956		0	0	0.00498	30.08	0.00162	3	2797.4688	1.981751	38.26099	1
15608956		0	0	0.2084	29.94	0.001012	2	940.54613	-0.14051	30.83896	0
15608956		0	0	0.003442	28.15	0.001527	3	2165.2406	0.20018	35.65691	2
15608956		0	0	0.2084	27.58	0.002444	3	1922.0421	-1.74964	30.33424	2
15608956	C6(Carban	0	0	0.001075	19.85	0.016045	6	3909.0093	-2.48762	36.78255	1
15608956		0	0	0.08855	12.9	0.056415	5	4057.1226	0.585535	37.30404	2
15608982		0	0	0.1264	107.79	3.66E-11	2	1893.9952	-1.01189	40.01835	0
15608982		0	0	0.1169	101.1	1.44E-10	3	2708.4561	-0.63557	42.67382	1
15608982		0	0	0.048144	89	2.64E-09	2	2465.3243	-0.18454	44.59122	0
15608982		0	0	0.06051	82.91	9.98E-09	2	2121.1407	2.52284	38.51485	1
15608982		0	0	0.1644	70.76	1.09E-07	2	1452.8163	-0.45396	27.64686	0
15608982	C11(Carba	0	0	0.153	70.52	1.86E-07	2	1770.9583	0.62918	35.88617	0
15608982		0	0	0.09448	63.18	4.81E-07	5	3185.7414	0.725107	40.65027	2
15608982	C14(Carba	0	0	0.1878	59.84	2.28E-06	2	2155.1685	-0.38079	38.97916	1
15608982	C5(Carban	0	0	0.1308	50.57	1.32E-05	3	2338.2623	-1.63073	29.61053	1
15608982		0	0	0.4195	46.63	2.82E-05	3	2942.6079	0.680692	41.98482	1
15608982	C5(Carban	0	0	0.3621	42.94	9.91E-05	2	904.46709	0.136339	17.04103	0
15609014		0	0	0.0004	101.23	9.79E-11	3	3761.822	2.050416	39.10357	1
15609014		0	0	0.000731	98.24	1.5E-10	2	2353.0938	-0.25231	33.54292	0
15609014		0	0	0.01016	87.59	1.74E-09	2	1584.6827	0.847795	35.96334	0
15609014		0	0	0.001002	84.08	3.9E-09	3	2655.5385	1.46298	49.53403	1
15609014		0	0	0.01087	77.38	4.39E-08	2	1427.7371	-0.43646	31.72551	0
15609014		0	0	0.03713	76.21	2.39E-08	2	1560.8907	0.590239	37.0097	0
15609014	C10(Carba	0	0	0.01135	73.18	6.01E-08	3	2409.3111	1.587179	45.63499	1
15609014		0	0	0.001406	69.51	2.97E-07	3	2341.2248	-0.51926	28.24057	1

15609014		0	0	0.03269	67.37	1.83E-07	2	2169.9935	-0.05323	38.89948	1
15609014		0	0	0.008741	66.35	4.63E-07	4	2842.4823	1.224004	39.68638	1
15609014		0	0	0.01206	66.21	3.71E-07	3	1381.7054	-1.2042	28.57034	0
15609014	C8(Carban	0	0	0.06071	66.15	4.85E-07	2	2140.118	-1.91007	47.25556	0
15609014		0	0	0.1114	65.97	3.04E-07	2	978.53581	-0.94744	19.12373	0
15609014		0	0	0.01031	65.52	3.79E-07	2	1113.6624	-0.26057	33.13425	0
15609014		0	0	0.003534	62.95	5.06E-07	3	2811.6352	-0.1975	47.84989	2
15609014		0	0	0.01559	61.06	1.76E-06	4	2558.3202	-0.83655	35.17926	1
15609014		0	0	0.0388	51.49	9.22E-06	4	4123.9904	1.102294	41.74102	2
15609014		0	0	0.04501	51.12	1.7E-05	2	1882.9572	-1.45536	33.92355	0
15609014		0	0	0.2209	46.32	2.92E-05	2	1389.7822	-2.01414	32.43315	1
15609014		0	0	0.07424	45.94	6.49E-05	3	2402.2218	0.22628	37.72999	0
15609014		0	0	0.006625	42.76	5.28E-05	3	3967.872	-3.19261	44.06022	1
15609014		0	0	0.01625	39.5	0.000258	3	2538.3237	-0.62944	35.30497	1
15609014		0	0	0.1034	36.12	0.00033	3	3917.9103	-1.31444	38.1733	2
15609014		0	0	0.1071	35.36	0.000975	2	942.52995	-0.30394	18.78868	1
15609014		0	0	0.02875	34.03	0.000593	3	2476.3548	1.146516	36.32656	1
15609014		0	0	0.08936	33.74	0.000549	4	3497.851	1.935918	40.09572	2
15609014		0	0	0.09861	30.1	0.001906	2	1583.8381	-0.49942	33.11867	1
15609014		0	0	0.3041	28.73	0.003885	2	786.42949	0.470046	18.80467	0
15609014		0	0	0.06619	24.39	0.003631	4	1716.9893	-0.92766	34.53756	1
15609014		0	0	0.096	23.05	0.00545	5	4205.1619	-1.47647	41.00702	2
15609110		0	0	5.09E-07	64.08	7.82E-07	3	2688.3202	-0.83573	30.86129	0
15609110		0	0.005	0.1144	25	0.008538	2	1132.603	-0.72261	41.86921	0
15609134	C8(Carban	0	0	4.09E-14	101.47	1.43E-10	2	1703.8558	1.352925	34.48532	0
15609134		0	0	1.31E-14	89.59	1.92E-09	2	1779.8799	-0.4612	24.6357	0
15609134	C10(Carba	0	0	2.92E-10	81.91	1.1E-08	2	1625.8428	-0.04568	21.28539	0
15609134	M6(Oxidat	0	0	1.66E-10	72.64	9.53E-08	2	1795.8764	0.403186	21.55762	0
15609134	C15(Carba	0	0	9.9E-08	64.98	6.35E-07	2	1931.0247	-1.051	29.59936	1
15609134		0	0	4.97E-06	64.62	6.04E-07	3	1800.9864	0.472506	36.2459	0
15609134	M6(Oxidat	0	0	3.54E-05	62.13	7.35E-07	4	3773.8082	2.20916	37.95093	0
15609134		0	0	0.000686	60.57	1.18E-06	2	898.5465	-0.47404	20.93354	0
15609134	M4(Oxidat	0	0	0.000539	59.47	1.53E-06	2	924.44481	-0.80645	14.85436	0
15609134	M16(Oxid	0	0	1.18E-06	58.44	3.72E-06	3	2330.1908	4.198691	33.42343	1
15609134	M8(Oxidat	0	0	1.42E-07	57.54	2.64E-06	2	1032.5248	-0.70548	17.89782	0
15609134		0	0	1.15E-08	55.9	4.24E-06	3	1566.8236	0.032049	23.32615	1

15609134		0	0	0.000833	55.6	5.78E-06	2	908.45024	-0.43854	18.20964	0
15609134		0	0	7.09E-05	55.04	8.62E-06	2	1016.5301	-0.49518	21.88759	0
15609134	C8(Carban	0	0	3.88E-10	50.17	2.4E-05	3	2133.0492	-0.67828	32.03804	1
15609134	M1(Oxidat	0	0	1.6E-06	49.24	1.19E-05	3	2223.0205	0.03852	28.51946	0
15609134	M4(Oxidat	0	0	0.1044	44.07	6.07E-05	3	1250.6515	-0.55402	18.33333	1
15609134	M3(Oxidat	0	0	0.006835	42.44	7.41E-05	2	1035.5036	-0.45259	16.02363	0
15609134	C13(Carba	0	0	3.86E-07	40.66	0.00015	2	2957.4332	0.702558	34.97057	0
15609134		0	0	0.002466	40.24	9.44E-05	2	1167.7311	-0.84317	27.13342	1
15609134		0	0	0.01021	39	0.000126	2	893.47215	-0.66792	27.76402	0
15609134	C5(Carban	0	0	0.01107	38.79	0.000145	3	2207.0218	-1.6568	30.51545	0
15609134	M6(Oxidat	0	0	0.000125	34.46	0.000357	4	3757.8021	-0.75884	41.67922	0
15609134		0	0	0.07086	32.91	0.001151	2	956.55138	-1.06961	20.10585	1
15609134	C5(Carban	0	0	0.04251	26.94	0.002018	3	2191.0283	-1.0369	33.23678	0
15609134	N-Term(A	0	0	0.1074	26.92	0.00315	3	1837.8852	-0.54643	25.42817	0
15609134		0	0	0.07732	12.58	0.07177	3	3741.8148	1.259056	42.30293	0
15609291		0	0	0.1795	62.78	5.26E-07	2	1291.7415	0.434589	33.69759	0
15609291		0	0	0.2819	57.92	3.23E-06	2	1278.6536	-1.07085	14.0196	0
15609291	N-Term(A	0	0	0.2961	41.09	0.000144	2	1320.6642	-0.99505	16.48086	0
15609291		0	0	0.1728	37.84	0.000164	3	1674.01	-0.09401	37.30385	1
15609291		0	0	0.3137	34.41	0.000398	2	1908.075	0.381259	38.73367	1
15609291		0	0	0.5139	31.68	0.000713	2	797.4262	-0.34641	13.12892	0
15609291	M2(Oxidat	0	0	0.3852	25.17	0.006386	2	1308.6857	1.161657	18.28115	0
15609291		0	0	0.456	15.48	0.052381	3	1292.6901	0.607888	20.59658	0
15609332	M10(Oxid	0	0	0.004031	87.71	2.12E-09	2	2144.0513	-1.30089	33.23331	0
15609332	C12(Carba	0	0	0.05681	47.57	1.75E-05	2	1486.66	0.527631	19.95438	0
15609332		0	0	0.2808	39.57	0.000199	2	876.49419	0.430036	19.71211	1
15609332	C12(Carba	0	0	0.4386	35.99	0.000251	3	2388.0412	-2.94109	18.28206	2
15609337		0	0	6.67E-10	83.43	6.81E-09	2	1940.0921	-1.56832	36.84068	0
15609337		0	0	1.88E-06	71.59	1.42E-07	2	1187.6406	2.196985	19.89494	1
15609337		0	0	1.24E-05	67.64	4.13E-07	2	1877.9892	1.215151	28.60215	0
15609337		0	0	2.08E-06	65.27	6.39E-07	3	2458.2323	1.587166	38.08516	0
15609337		0	0	1.04E-06	63.69	4.27E-07	2	1409.617	-0.70966	21.897	0
15609337		0	0	1.9E-09	63.38	5.51E-07	2	1897.8914	-0.69882	25.70512	1
15609337	M5(Oxidat	0	0	7.67E-06	59.45	2.72E-06	3	2641.3165	0.160957	48.12797	0
15609337		0	0	0.001477	53.58	9.21E-06	2	1118.584	-0.06198	25.0158	0
15609337		0	0	0.022	36.19	0.000361	2	2034.0895	0.739317	27.29119	1

15609337		0	0	0.2128	36.06	0.000446	2	778.42082	0.247509	15.28094	0
15609337		0	0	0.006999	35.73	0.000321	3	2821.5269	1.211515	40.03702	1
15609337		0	0	0.4054	26.61	0.002183	2	900.44615	-0.03081	25.26193	0
15609373		0	0	3.1E-06	97.13	5.42E-10	2	2159.1246	0.031502	39.85477	0
15609373		0	0	8.27E-06	83.23	6.42E-09	2	1279.6086	1.513593	31.9063	0
15609373		0	0	0.005242	78.8	2.18E-08	3	3325.7494	1.957894	45.77644	0
15609373		0	0	0.000197	72.44	5.69E-08	2	1181.6263	-0.94192	23.44118	0
15609373		0	0	2.53E-05	71.1	1.16E-07	2	2038.9897	0.662251	35.88453	1
15609373		0	0	0.002085	70.22	1.85E-07	2	1353.6834	0.166884	31.79701	0
15609373	M8(Oxidat	0	0	0.000528	67.46	3.23E-07	2	1369.6766	-1.11342	25.81739	0
15609373		0	0	0.002161	57.22	4.84E-06	2	2044.04	-0.93771	26.74455	2
15609373	C7(Carban	0	0	0.1099	54.18	4.97E-06	4	2234.2201	0.607899	28.82186	2
15609373	C6(Carban	0	0	5.44E-05	53.52	6.22E-06	3	2078.1147	-1.43261	30.71584	1
15609373		0	0	0.001351	44.61	5.19E-05	3	1729.8719	0.143955	26.92024	1
15609373		0	0	0.000615	43.98	0.000108	3	1287.7007	-0.68615	25.63126	1
15609373	C7(Carban	0	0	0.001008	39.36	0.0004	2	1071.6092	-0.03438	24.93689	1
15609373		0	0	0.08782	39.09	0.000166	2	778.40019	0.851468	31.39981	0
15609373		0	0	0.1185	38.55	0.000237	3	1210.6479	-0.09826	27.00658	1
15609373		0	0	0.1861	37.82	0.000496	3	1885.9724	-0.1835	25.53117	2
15609373	M8(Oxidat	0	0	0.000603	33.07	0.00074	3	1709.865	0.595419	24.60976	1
15609373		0	0	0.1203	27.21	0.004563	2	973.53166	0.34155	24.93872	0
15609373		0	0	0.0112	18.92	0.028852	3	1693.8715	1.418533	29.17472	1
15609399		0	0	6.85E-11	113.35	8.09E-12	3	3387.6844	0.797022	40.03924	0
15609399		0	0	7.04E-08	89.74	2.76E-09	2	1984.0357	-1.51165	34.2971	0
15609399		0	0	2.36E-06	67.04	6.03E-07	2	1515.7909	-0.35635	23.20143	0
15609399		0	0	5.51E-07	58.67	1.36E-06	2	1192.6731	0.521519	38.07705	0
15609399		0	0	1.27E-06	52.54	5.56E-06	3	1449.8221	0.5486	33.54784	1
15609399	N-Term(A	0	0	1.39E-06	49.11	1.41E-05	2	1234.683	-0.04479	44.48807	0
15609399		0	0	0.000302	48.48	2.13E-05	3	4176.1087	-0.56118	37.95982	1
15609399		0	0	0.1155	48.09	4.97E-05	2	816.45848	1.287705	19.93664	0
15609399		0	0	0.000124	40.4	0.000141	2	807.44652	-0.84406	16.61907	0
15609399	N-Term(A	0	0	0.006148	38.76	0.000259	2	1484.7599	-0.39805	22.82213	0
15609399		0	0	0.007488	34.36	0.000971	3	1442.7487	-0.90136	18.04177	0
15609399	C11(Carba	0	0	0.02809	19.73	0.021283	2	1579.8437	1.430666	33.64612	1
57116962		0	0	0.01858	34.41	0.000561	2	1134.5541	0.232203	20.37704	0
57116962	C1(Carban	0	0	0.03353	31.31	0.001109	3	1543.7506	-0.15922	28.98583	0

15609476		0	0	0.007879	125.43	4.44E-13	2	1936.9755	-0.46456	42.90806	0
15609476		0	0	0.07349	96.52	3.68E-10	2	1272.6434	0.201821	21.90422	0
15609476		0	0	0.07268	77.18	3.54E-08	2	1381.779	-0.63906	18.82401	1
15609476	M12(Oxid	0	0	0.1556	74.95	3.52E-08	2	1346.6366	-0.24656	16.96627	0
15609476		0	0	0.1554	62.15	9.45E-07	3	3041.4946	1.344971	37.8973	0
15609476		0	0	0.09805	52.23	6.58E-06	4	4295.1198	0.931379	40.30461	1
15609476		0	0	0.356	49.89	1.54E-05	2	1330.6407	-1.04363	20.22055	0
15609476		0	0	0.3226	45.86	4.8E-05	2	1253.6833	-1.28913	19.73199	0
15609476	M12(Oxid	0	0	0.4127	26.55	0.002324	2	1720.8101	-0.30515	15.8971	1
15609482		0	0	2.19E-13	121.6	1.69E-12	2	1851.9543	5.135079	36.63778	0
15609482		0	0	2.05E-13	99.66	1.89E-10	2	1753.8716	0.027556	48.0447	0
15609482		0	0	0.000283	92.26	1.13E-09	3	2367.1889	0.340767	43.01648	0
15609482	M28(Oxid	0	0	0.000396	91.32	1.14E-09	3	4214.0735	0.529926	42.03777	1
15609482		0	0	2.44E-10	83.9	5.09E-09	3	4198.0741	-0.53394	43.84723	1
15609482		0	0	1.05E-11	80.35	1.85E-08	2	2210.1436	1.06825	34.89256	1
15609482		0	0	5.33E-05	73.97	1.12E-07	2	903.48924	-0.25116	17.14082	0
15609482		0	0	0.000603	61.78	1.56E-06	2	1521.8028	0.480137	28.88847	1
15609482	M10(Oxid	0	0	1.23E-05	60.13	2.04E-06	2	2381.1451	0.319523	32.41568	0
15609482		0	0	9.63E-05	59.16	2.37E-06	2	2365.1482	-0.48637	35.9718	0
15609482		0	0	5.37E-10	49.74	2.44E-05	3	2636.3692	-1.57282	43.6546	1
15609482		0	0	2.48E-06	49.56	2.1E-05	3	1677.9025	-0.4001	25.96664	2
15609482		0	0	0.000196	47.33	3.51E-05	3	4354.1894	2.754805	42.68587	2
15609482		0	0	0.1326	31.57	0.001567	2	793.43065	-1.12256	12.92676	1
15609482	M28(Oxid	0	0	1.36E-07	30.92	0.001416	4	4370.1754	0.706938	48.74269	2
15609482		0	0	0.0493	30.4	0.002234	2	2521.2479	-1.05542	34.21524	1
15609482	M10(Oxid	0	0	0.1397	27.81	0.003808	3	2537.2412	-1.67199	31.97979	1
15609580		0	0	0.1733	75.13	4.3E-08	3	2080.1059	-0.71686	43.0302	0
15609580		0	0	0.29	62.4	1.06E-06	2	1359.7476	-0.20968	28.07018	1
15609780	M3(Oxidat	0	0	2.52E-08	98.92	1.8E-10	2	1556.784	-0.67649	28.87559	0
15609780		0	0	2.27E-08	93.26	4.71E-10	2	1340.8158	0.739995	41.16178	0
15609780		0	0	3.82E-12	82.2	1.33E-08	2	1376.7532	-0.07254	29.63572	0
15609780		0	0	4.59E-10	79.94	2.08E-08	2	1540.7898	-0.18093	30.8469	0
15609780	N-Term(A	0	0	3.85E-10	64.2	8.93E-07	3	2096.0901	-0.80591	34.90844	1
15609780		0	0	2.49E-06	54.69	4.75E-06	3	2038.0823	-1.9733	31.89066	1
15609780		0	0	2.44E-08	50.15	2.13E-05	2	1804.9682	-1.30421	32.92684	1
15609780	M3(Oxidat	0	0	1.85E-06	42.82	9.93E-05	4	2054.0814	0.095081	30.79669	1

15609780	N-Term(A	0	0	0.000227	37.05	0.000306	3	2080.0968	-0.05837	38.87841	1
15609780		0	0	0.137	36.11	0.000404	2	1104.5448	-1.66937	19.66622	1
15609780	M1(Oxidat	0	0	0.007555	34.04	0.000454	2	1120.5406	-0.92041	17.1456	1
15609780	N-Term(A	0	0	0.001386	30.23	0.002181	2	1846.9734	-4.15276	35.13676	1
57117165	M9(Oxidat	0	0	5.8E-16	108.36	1.46E-11	2	2104.9175	0.592312	31.66443	0
57117165		0	0	5.8E-16	89.61	1.42E-09	2	1900.9546	1.781285	37.92465	0
57117165	M19(Oxid	0	0	0.126	38.22	0.00015	2	2088.9173	-1.9543	31.17942	0
15609827		0	0	5.23E-10	108	3.8E-11	2	2171.0948	0.36519	29.82635	0
15609827		0	0	1E-06	79.86	1.65E-08	2	1680.8608	0.121409	33.73466	0
15609827		0	0	6.46E-09	71.35	1.5E-07	4	2668.3913	0.399026	34.0085	1
15609827		0	0	8.63E-06	59.24	2.5E-06	2	1348.7216	-0.29367	18.12818	1
15609827		0	0	3.7E-05	57.41	2.81E-06	2	1401.7011	0.118501	27.82784	0
15609827		0	0	3.02E-09	55.19	6.51E-06	2	2378.2222	-1.09146	46.42046	0
15609827	M8(Oxidat	0	0	1.3E-09	52.57	1.6E-05	3	2394.2243	1.900831	43.06958	0
15609827		0	0	6.49E-05	52.02	7.22E-06	2	1107.5422	-0.72182	17.28343	0
15609827		0	0	2.97E-07	51.94	7.36E-06	3	1578.8899	0.503135	37.79106	0
15609827		0	0	0.000287	51.37	7.28E-06	4	1797.0863	0.279963	31.39416	1
15609827		0	0	7.4E-05	48.01	3E-05	3	2591.3501	1.053701	42.33923	1
15609827	M4(Oxidat	0	0	0.001989	47.44	4.51E-05	2	2410.211	-1.51696	39.19064	0
15609827	M10(Oxid	0	0	7.58E-07	45.6	5.78E-05	3	2607.3389	-1.30975	39.85742	1
15609827		0	0	0.000525	44.47	8.93E-05	3	2188.1407	0.42908	33.74858	1
15609827		0	0	0.002063	42.53	0.000131	2	1898.9939	-1.68364	29.11332	1
15609827		0	0	0.000495	41.58	0.000136	2	1488.8533	-0.02164	24.47042	1
15609827		0	0	0.002149	40.92	0.000146	2	1332.7524	0.10177	26.53558	0
15609827		0	0	9.64E-05	40.29	0.000159	4	4051.0709	-0.57707	39.02939	2
15609827		0	0	0.001592	39.14	0.000122	2	966.57268	-0.46749	17.56891	0
15609827		0	0	0.000103	29.89	0.001023	2	849.52971	-0.96596	27.4762	0
15609885		0	0	0.1187	130.34	2.08E-13	2	1958.019	-0.02697	28.61219	0
15609885	M4(Oxidat	0	0	0.2164	79.69	1.24E-08	2	1178.6573	0.880545	18.1715	1
15609885	C10(Carba	0	0	0.3544	59.05	2.86E-06	2	1233.6623	0.189472	27.09364	0
15609885		0	0	0.2707	54.53	6.34E-06	3	2681.4128	0.306771	32.431	1
15610388	C1(Carban	0	0	0.07718	75.34	5.12E-08	3	2807.3857	0.751222	35.62327	0
15610388		0	0	0.1287	68.29	4E-07	2	1259.6376	-0.23853	21.65587	0
15610388		0	0	0.157	61.93	8.98E-07	2	2827.3924	-0.83105	46.69122	0
15610388	M2(Oxidat	0	0	0.1852	56.07	3.58E-06	2	2843.3882	-0.49768	46.98901	0
15610388	M2(Oxidat	0	0	0.09889	52.75	7.17E-06	2	2859.3777	-2.38799	42.75087	0

15610388		0	0	0.4645	37.13	0.000484	2	1976.0427	-1.76674	24.33787	1
15610388		0	0	0.425	31.89	0.000777	2	942.5855	1.183466	17.99532	2
15610388		0	0	0.5085	29.12	0.001222	2	836.47392	0.231032	14.89546	1
15610388		0	0	0.4851	21.03	0.00787	2	990.59734	1.752819	16.31083	1
15610900		0	0	1.27E-05	80.5	2.23E-08	2	1270.6999	-0.26332	34.15514	0
15610900		0	0	0.1221	31.36	0.001718	2	1771.9074	-0.69703	25.24632	0
15610915		0	0	5.08E-06	69.42	1.14E-07	2	1040.6458	-0.49176	31.01852	0
15610915		0	0	0.002292	55.54	5.86E-06	2	1547.8077	0.764621	33.45231	0
15610915		0	0	2.83E-10	49.15	2.61E-05	2	1059.5406	-0.49678	37.53486	0
15610915	M5(Oxidat	0	0	0.1439	44.01	6.16E-05	2	1579.7776	-0.58287	34.05459	0
15611005		0	0	0.1777	92.38	6.07E-10	2	1489.7242	-0.01518	23.28719	0
15611005	M5(Oxidat	0	0	0.1689	82.21	6E-09	2	1737.7795	0.322391	27.82088	0
15611005		0	0	0.2524	77.72	3.04E-08	3	2558.3772	1.80197	42.36358	0
15611005	M13(Oxida	0	0	0.08311	76.15	2.91E-08	2	2030.1224	0.34764	30.18549	0
15611005		0	0	0.1825	68.75	1.33E-07	2	1721.7848	0.491547	28.6114	0
15611005		0	0	0.2061	67.63	1.9E-07	2	1066.553	0.210786	26.46687	0
15611005		0	0	0.2055	67	2.79E-07	2	2014.1275	0.37123	33.04285	0
15611005		0	0	0.1552	64.33	6.64E-07	2	1753.9239	0.361717	33.99631	0
15611005		0	0	0.2476	59.47	3.22E-06	2	1129.6003	0.15583	23.48824	0
15611005	C11(Carba	0	0	0.2485	57.9	3.24E-06	3	1999.0027	2.948152	30.28227	0
15611005		0	0	0.297	57.32	2.41E-06	2	1293.7514	-0.92052	24.31052	0
15611005		0	0	0.2323	50.88	8.15E-06	2	940.48418	-0.56656	17.63975	0
15611005		0	0	0.2668	50.72	1.57E-05	2	1817.9006	-1.29325	26.216	0
15611005		0	0	0.2117	36.4	0.000229	2	1040.6351	-0.023	27.03524	0
15611005		0	0	0.2614	32.88	0.000721	3	1153.607	-0.26269	16.88893	1
15611013		0	0	1.72E-10	78.96	3.49E-08	3	2661.3426	0.6734	40.87763	0
15611013		0	0	4.78E-08	69.49	3.21E-07	2	2359.2342	-0.44626	41.78415	0
15611013		0	0	0.009884	38.7	0.000155	2	759.47191	-0.58055	21.95002	0
15611030		0	0	5.85E-09	116.98	4.41E-12	3	4591.2964	0.228375	43.20343	1
15611030		0	0	9.27E-08	80.11	2.34E-08	2	2171.095	-1.99116	32.72424	0
15611030		0	0	0.001481	72.91	5.1E-08	2	1526.7144	1.415961	33.07915	0
15611030		0	0	7.64E-05	59.65	1.46E-06	2	1049.5267	0.378863	30.62284	0
15611030	C8(Carban	0	0	0.02296	57.34	1.84E-06	2	1290.6084	-1.77379	21.12061	0
15611030		0	0	5.98E-05	56.56	5.74E-06	2	1262.6481	-0.58006	28.22075	1
15611030	C14(Carba	0	0	0.05759	41.79	6.62E-05	3	2004.9399	-0.29019	24.44459	1
15611030		0	0	2.06E-05	40.96	8.82E-05	3	3689.8279	-0.44645	38.35802	0

15611030		0	0	0.01305	34.52	0.000901	2	920.48338	-0.2893	26.6984	0
15611030	C14(Carba	0	0	1.58E-05	31.26	0.000746	4	4157.0114	-2.54078	37.08525	2
15611030	C17(Carba	0	0	0.09651	27.7	0.002802	4	2437.1891	0.066601	25.49513	2
15611057		0	0	0.000347	98.56	1.39E-10	3	3601.5736	-0.63855	18.75213	0
15611057		0	0	0.02137	28.64	0.001364	3	3757.667	-2.6708	18.40651	1

Protein Group	Modificati	ΔCn	q-Value	PEP	IonScore	Exp Value	Charge	MH+ [Da]	ΔM [ppm]	RT [min]	Missed Cleavages
15607150		0	0	0.004659	135.97	4.55E-14	3	3619.8537	-3.83673	45.49051	1
15607150		0	0	0.005751	100.01	1.05E-10	2	1482.8637	-0.16606	48.11373	0
15607150		0	0	0.08067	86.9	4.19E-09	3	3234.6588	-0.44972	48.62929	0
15607150		0	0	6.47E-05	84	3.97E-09	3	2139.2254	0.38549	37.10605	1
15607150		0	0	0.141	66.35	6.49E-07	2	1404.7461	-1.48066	26.95729	0
15607150		0	0	0.3377	39.7	0.000182	2	831.47923	-0.40493	12.06068	1

Protein Group	Modificati	ΔCn	q-Value	PEP	IonScore	Exp Value	Charge	MH+ [Da]	ΔM [ppm]	RT [min]	Missed Cleavages
15607533		0	0	0.06903	96.08	3.33E-10	2	1815.8831	-2.28928	28.96778	0
15607533		0	0	0.0883	93.23	4.74E-10	2	1393.6255	-0.67121	19.75921	0
15607533		0	0	0.07057	72.29	7.38E-08	2	1283.757	0.003355	32.54902	0
15607533	M1(Oxidat	0	0	0.0921	71.79	6.61E-08	2	1409.6203	-0.77996	17.70078	0
15607533		0	0	0.06174	71.13	8.48E-08	3	2667.4825	-0.19692	41.16794	1
15607533		0	0	0.04295	70.96	1.2E-07	2	2511.3807	-0.47721	43.40828	0
15607533	M12(Oxid	0	0	0.2814	67.59	4.01E-07	2	1555.8101	0.661965	41.91245	0
15607533		0	0	0.08239	60.94	1.57E-06	2	1226.6873	-1.59604	27.48663	0
15607533	M14(Oxid	0	0	0.145	51.03	7.87E-06	2	1717.9902	-1.19116	34.72686	0
15607533		0	0	0.2047	48.78	2.45E-05	2	1045.5519	-0.52013	26.86565	0
15607533		0	0	0.279	44.22	8.14E-05	2	1112.6572	0.845535	26.54247	0
15607533		0	0	0.2454	37.69	0.00017	2	1701.9985	0.687066	37.10055	0
15607533	M12(Oxid	0	0	0.1205	35.59	0.000663	4	2905.5128	-0.50876	38.36481	0
15607533		0	0	0.2734	27.39	0.004104	2	2101.0293	-0.77906	26.37076	1

Protein Group	Modificati	ΔCn	q-Value	PEP	IonScore	Exp Value	Charge	MH+ [Da]	ΔM [ppm]	RT [min]	Missed Cleavages
15607898		0	0	2.63E-14	118.52	3.02E-12	2	1779.9113	-0.61734	41.17775	0
15607898	M19(Oxid	0	0	6.38E-14	112.77	5.27E-12	3	3207.479	1.290959	34.94491	1
15607898		0	0	1.85E-09	98.26	2.91E-10	2	2097.0696	-0.71224	45.90267	0
15607898		0	0	8.97E-11	94.82	3.29E-10	2	2660.202	0.973834	33.13654	0
15607898		0	0	4.55E-08	91.86	8.47E-10	2	1441.8447	-0.09655	43.99313	0
15607898		0	0	7.76E-14	90.52	1.46E-09	3	3590.8714	-1.158	41.83492	0
15607898	M19(Oxid	0	0	8.29E-13	90.41	9.08E-10	3	2676.191	-1.24213	29.68399	0
15607898	M1(Oxidat	0	0	4.09E-10	89.91	1.48E-09	2	1457.8396	-0.04053	45.00327	0

15607898		0	0	5.81E-10	87.62	3.2E-09	2	1537.7969	-0.03817	34.45873	0
15607898		0	0	9.82E-08	77.95	3.61E-08	3	2253.1741	0.827774	32.92234	1
15608172;156		0	0	7.82E-08	73.59	9.63E-08	2	1316.6587	-0.49542	24.31009	0
15607898		0	0	6.16E-10	72.02	1.35E-07	2	2314.2127	-1.04451	38.66034	0
15607898	M8(Oxidat	0	0	3.26E-05	68.02	2.6E-07	2	1543.8633	0.5004	31.55365	0
15607898		0	0	1.25E-07	67.79	2.66E-07	2	1527.8678	0.133618	37.19997	0
15607898		0	0	9.44E-09	62.03	1.16E-06	2	2035.0523	-2.20516	25.94058	0
15607898		0	0	7.51E-06	59	3.34E-06	3	2223.1628	0.495951	36.496	1
15607898		0	0	2.74E-07	58.07	3.51E-06	3	2470.3145	-0.70599	35.30825	1
15607898	N-Term(A	0	0	0.000497	54.32	5.73E-06	2	1483.8537	-1.12613	48.7345	0
15607898	M4(Oxidat	0	0	0.000163	52.79	1.29E-05	2	906.47063	-0.83582	28.57275	0
15607898		0	0	2.65E-05	52.21	1.47E-05	3	2470.3181	0.776453	37.36199	1
15607898	M8(Oxidat	0	0	2.49E-05	47.91	2.91E-05	3	2230.2088	-1.09446	41.93747	1
15607898	N-Term(A	0	0	2.04E-05	46.35	4.75E-05	3	2356.2271	0.623738	43.09678	0
15607898		0	0	0.004975	45.34	6.29E-05	2	801.4821	-1.00818	21.09463	0
15608172;156	M1(Oxidat	0	0	0.002642	42.09	9.89E-05	3	1619.7934	-1.54103	21.73065	1
15607898		0	0	0.000104	40.42	0.000191	3	2366.2549	-0.58926	34.35999	1
15607898	C33(Carba	0	0	3.03E-06	36.28	0.000377	3	3461.7077	-2.19906	42.59772	0
15607898		0	0	0.06412	35.6	0.000372	2	855.43584	-0.14342	25.02374	0
15607898		0	0	0.04075	35.48	0.000878	2	890.47637	-0.1183	27.02932	0
15607898	C10(Carba	0	0	0.1299	28.1	0.00302	3	1379.7211	-0.13902	21.10854	0
15607898	N-Term(A	0	0	0.07816	27.78	0.003418	2	1499.8509	0.404291	43.55904	0
15607898		0	0	0.1167	26.45	0.003963	4	2969.4601	0.017651	29.22018	0
15607898		0	0	0.08802	20.31	0.01257	2	2099.1243	-0.06961	30.69439	1

Protein Group	Modificati	ΔCn	q-Value	PEP	IonScore	Exp Value	Charge	MH+ [Da]	ΔM [ppm]	RT [min]	Missed Cleavages
57116780		0	0	0.1295	132.49	1.35E-13	2	1955.023	1.087208	43.14178	0
57116780	M8(Oxidat	0	0	0.0303	120.48	2.37E-12	2	1971.016	0.128111	48.95236	0
57116780	M9(Oxidat	0	0	0.321	80.31	2E-08	2	2329.1727	0.909645	44.53406	0
57116780	C3(Carban	0	0	0.08742	77.34	1.84E-08	2	1544.6698	0.778227	24.0885	0
57116780	M9(Oxidat	0	0	0.05061	76.96	5.94E-08	3	2901.4634	0.353876	42.641	1
57116780	M34(Oxid	0	0	0.0835	67.78	3.58E-07	3	4168.1114	-0.47335	31.88748	0
57116780		0	0	0.2521	66.37	6.11E-07	2	2313.1783	1.145158	44.5669	0
57116780		0	0	0.4991	61.64	2.02E-06	3	2885.4688	0.454988	44.40164	1
57116780		0	0	0.3958	53.23	7.37E-06	3	4152.1153	-0.75907	33.44992	0
57116780	N-Term(A	0	0	0.3989	33.57	0.000681	3	2371.1819	0.344815	41.39873	0
57116780	C5(Carban	0	0	0.4503	13.26	0.063729	5	4598.3037	0.213527	48.34713	0

Protein Group	Modificati	ΔCn	q-Value	PEP	IonScore	Exp Value	Charge	MH+ [Da]	ΔM [ppm]	RT [min]	Missed Cleavages
15608144	M6(Oxidat	0	0	3.78E-11	89.86	1.03E-09	3	4920.6075	-0.17056	38.03296	0
15608144	C6(Carban	0	0	3.64E-09	81.3	7.4E-09	2	1466.6119	0.480499	12.52906	0
15608144		0	0	0.09153	16.13	0.02432	5	4904.6211	1.561733	39.16704	0
Protein Group	Modificati	ΔCn	q-Value	PEP	IonScore	Exp Value	Charge	MH+ [Da]	ΔM [ppm]	RT [min]	Missed Cleavages
15608172		0	0	0.002386	114.7	4.91E-12	2	2052.9822	-0.54845	29.19319	0
15608172	M1(Oxidat	0	0	0.05562	93.15	5.57E-10	2	1531.8752	-0.85296	38.74747	0
15608172		0	0	0.003414	92.61	5.47E-10	2	1701.006	0.104493	39.95024	0
15608172		0	0	0.1048	88.57	3.47E-09	2	1503.8163	-0.19917	35.41361	0
15608172	M11(Oxida	0	0	0.02277	88.52	1.97E-09	3	2427.1827	0.229103	26.19492	0
15608172		0	0	0.004873	82.03	1.1E-08	3	2261.1288	0.772179	25.53896	0
15608172		0	0	0.04706	81.92	7.71E-09	2	1763.8429	-0.9968	27.39812	0
15608172		0	0	0.07949	81.25	7.48E-09	2	1509.7292	-0.07135	21.48954	0
15608172		0	0	0.03214	79.62	2.29E-08	2	1395.7022	0.41179	25.59095	0
15608172		0	0	0.08929	69.43	2.05E-07	2	1293.6905	-0.43597	26.54617	0
15608172	M12(Oxida	0	0	0.01981	65.66	3.8E-07	3	2583.2816	-0.65303	26.30525	1
15608172		0	0	0.03859	55.18	6.67E-06	2	1316.6593	-0.03186	22.95008	0
15608172	M1(Oxidat	0	0	0.2477	48.55	2.37E-05	2	1619.7957	-0.13854	22.61061	1
15608172		0	0	0.09149	45.94	4.71E-05	3	3429.6643	-0.83143	34.23113	1
15608172	C7(Carban	0	0	0.1844	42.25	5.96E-05	3	3289.5725	1.266907	40.60222	0
15608172	M2(Oxidat	0	0	0.2042	41.19	7.59E-05	3	1687.9766	-0.59222	41.32914	1
15608172		0	0	0.1456	36.02	0.0004	3	3248.6404	-0.96573	36.59938	1
15608172		0	0	0.3302	21.38	0.017103	3	1979.0297	-0.44671	29.23893	1
Protein Group	Modificati	ΔCn	q-Value	PEP	IonScore	Exp Value	Charge	MH+ [Da]	ΔM [ppm]	RT [min]	Missed Cleavages
15608492		0	0	5.25E-16	77.93	4.43E-08	2	1499.8211	-0.4259	40.43769	0
15608492		0	0	5.25E-16	75.85	3.12E-08	3	2597.2556	1.423844	36.61773	0
Protein Group	Modificati	ΔCn	q-Value	PEP	IonScore	Exp Value	Charge	MH+ [Da]	ΔM [ppm]	RT [min]	Missed Cleavages
15608556		0	0	0.01226	83.72	1.08E-08	2	1090.5644	0.318508	19.48068	0
15608556		0	0	0.009006	78.27	5.21E-08	2	1171.6474	3.725665	18.35078	0
15608556		0	0	6.35E-10	55.04	7.52E-06	3	2116.0821	1.826554	31.18826	1
15608556		0	0	0.000318	51.95	1.85E-05	4	2343.2152	-0.54226	27.66431	2
15608556		0	0	0.00016	47.18	4.69E-05	2	1271.6734	2.418972	21.31119	1
15608556		0	0	0.00031	36.55	0.000564	2	1044.5313	-0.74072	24.39332	0
15608556	C14(Carba	0	0	0.06377	26.12	0.003909	3	2259.0979	-1.045	37.03779	0
Protein Group	Modificati	ΔCn	q-Value	PEP	IonScore	Exp Value	Charge	MH+ [Da]	ΔM [ppm]	RT [min]	Missed Cleavages
15609398		0	0	0.0129	110.24	2.18E-11	3	3681.8987	-7.15298	35.34178	1

15609398		0	0	0.03709	104.22	7.19E-11	2	2258.1033	-0.69183	31.9073	0
15609398		0	0	0.06174	84.48	7.66E-09	3	3246.6943	1.105049	36.79667	0
15609398	M10(Oxid	0	0	0.06708	65.02	8.18E-07	3	2010.0024	1.088152	36.49417	0
15609398		0	0	0.2372	43.63	0.000139	3	1994.009	1.883151	38.23623	0
Protein Group	Modificati	ΔCn	q-Value	PEP	IonScore	Exp Value	Charge	MH+ [Da]	ΔM [ppm]	RT [min]	Missed Cleavages
15609455		0	0	0.3567	83.71	1.13E-08	2	1677.8472	-0.6471	36.33354	0
15609455		0	0	0.07534	57.2	4.29E-06	2	1304.6634	-0.03344	30.72633	0
15609455		0	0	0.06946	42.09	0.00013	2	1588.8459	-1.52218	40.57957	0
15609455	M9(Oxida	0	0	0.2367	27.13	0.002905	3	1375.6644	0.63644	21.15603	0
Protein Group	Modificati	ΔCn	q-Value	PEP	IonScore	Exp Value	Charge	MH+ [Da]	ΔM [ppm]	RT [min]	Missed Cleavages
15610051		0	0	2.2E-13	93.51	1.05E-09	2	1870.93	2.430844	26.91898	0
15610051		0	0	1.2E-18	92.82	1.04E-09	3	2830.4293	1.195263	38.02902	0
15610051		0	0	6.35E-12	91.06	2.12E-09	3	2447.2767	0.668178	26.00805	1
15610051		0	0	1.97E-08	80.17	1.78E-08	2	2310.2229	-0.54251	42.6764	0
15610051		0	0	2.97E-06	69.74	3.5E-07	2	759.43566	-0.41582	20.56123	0
15610051		0	0	0.01458	45.19	5.6E-05	3	1625.8969	-0.09629	26.5822	1
Protein Group	Modificati	ΔCn	q-Value	PEP	IonScore	Exp Value	Charge	MH+ [Da]	ΔM [ppm]	RT [min]	Missed Cleavages
15610526	M11(Oxid	0	0	0.1629	82.92	6.13E-09	2	2793.3353	-0.30559	27.99399	1
15610526		0	0	0.1386	70.26	9.4E-08	2	2621.2356	-1.68928	32.7612	0
15610526		0	0	0.0358	70	2.55E-07	2	1298.7217	0.089308	29.70408	0
15610526	M10(Oxid	0	0	0.2951	58.3	1.48E-06	2	2637.2332	-0.67662	30.73744	0
15610526		0	0	0.3889	51.94	1.63E-05	2	987.5258	-0.05758	27.44552	0
15610526	N-Term(A	0	0	0.1227	46.43	5.69E-05	2	1340.7314	-0.60076	36.26636	0
15610526		0	0	0.1666	43.42	7.96E-05	3	1891.0131	-0.74861	27.05699	1
15610526		0	0	0.4434	30.26	0.001978	3	1533.8379	-0.37002	28.66216	0
Protein Group	Modificati	ΔCn	q-Value	PEP	IonScore	Exp Value	Charge	MH+ [Da]	ΔM [ppm]	RT [min]	Missed Cleavages
15610628		0	0	3.92E-08	72.94	8.13E-08	2	1608.8633	0.060632	35.79819	0
15610628		0	0	2.36E-05	63.99	8.18E-07	2	975.48503	-0.42886	11.19708	0
15610628		0	0	1.16E-08	58.35	1.75E-06	3	2287.1056	-0.37924	36.57931	0
15610628	N-Term(A	0	0	0.000925	52.75	8.49E-06	2	1650.8741	0.166419	40.37867	0
15610628		0	0	7.06E-05	48.31	4.65E-05	2	2269.1832	-1.42264	34.16858	1
15610628	M3(Oxida	0	0	3.71E-06	44.88	3.9E-05	3	2303.1004	-0.42132	33.16096	0
15610628		0	0	0.00079	40.91	0.000199	2	1141.6214	0.143821	20.73377	0
15610628	N-Term(A	0	0	0.03467	32.3	0.001119	3	2867.4518	-4.58803	39.27962	1
Protein Group	Modificati	ΔCn	q-Value	PEP	IonScore	Exp Value	Charge	MH+ [Da]	ΔM [ppm]	RT [min]	Missed Cleavages
15610586		0	0	5.68E-08	104.13	4.64E-11	2	1799.846	0.718708	49.50438	0

15610586		0	0	2.15E-05	91.61	8.63E-10	2	1594.8975	1.415286	39.64271	0
15610586		0	0	7.83E-05	86.37	2.77E-09	2	1325.7217	0.33641	28.88325	0
15610586		0	0	4.64E-08	85.37	6.1E-09	2	2333.221	0.040101	21.30353	1
15610586		0	0	6.03E-12	83.47	6.07E-09	2	1897.0627	-1.49264	31.62262	0
15610586		0	0	4.17E-07	82.93	5.08E-09	2	1427.8373	0.228718	49.08605	0
15610586	N-Term(A	0	0	1.88E-09	81.52	9.51E-09	2	1469.8489	0.924076	41.69639	0
15610586		0	0	1.76E-07	79.13	3.73E-08	2	2177.1207	0.400422	22.57025	0
15610586	C6(Carban	0	0	1.48E-08	78.09	3.65E-08	2	1899.9773	1.42487	29.81496	0
15610586		0	0	1.29E-10	75.82	3.27E-08	3	2446.3462	0.30381	37.36198	0
15610586		0	0	1.16E-12	75.14	3.05E-08	3	2351.3293	-0.63093	30.87692	1
15610586		0	0	0.01995	72.38	1.47E-07	2	1691.8876	0.685353	31.09715	0
15610586		0	0	0.000269	69.07	1.98E-07	2	1055.6194	-1.32523	27.4549	0
15610586		0	0	5.65E-07	60.41	9.1E-07	3	1750.9971	0.418713	36.01324	1
15610586		0	0	4.44E-06	56.57	4.41E-06	2	1957.9927	-2.63429	49.56457	0
15610586		0	0	3.6E-07	56	4.4E-06	3	2567.3369	0.131231	34.51873	1
15610586		0	0	4.33E-07	52.7	9.94E-06	3	2170.0765	-0.51663	28.94113	1
15610586		0	0	0.000571	51.94	7.04E-06	2	1023.5469	-0.04758	22.04702	0
15610586		0	0	0.0181	42.75	5.3E-05	4	3753.0561	1.915687	39.97249	1
15610586		0	0	0.001211	41.93	0.000176	3	1896.9767	-0.42363	23.91108	1
15610586		0	0	0.01756	15.02	0.073972	3	2756.4724	-0.25215	48.14734	0
15610586	N-Term(A	0	0	0.009845	13.55	0.066236	3	2488.3579	0.762339	44.11629	0
15610586		0	0.004	0.06748	55.52	7.01E-06	2	1610.8248	0.107603	18.02401	1
15610586		0	0.004	0.02581	41.44	8.97E-05	2	894.46733	-0.73624	18.25013	0
15610586	N-Term(A	0	0.004	0.05355	34.04	0.000434	2	1636.9065	0.443177	45.48535	0
15610586		0	0.004	0.03183	19.23	0.011912	3	1798.073	1.723992	38.2686	1

Protein Group	Modificati	ΔCn	q-Value	PEP	IonScore	Exp Value	Charge	MH+ [Da]	ΔM [ppm]	RT [min]	Missed Cleavages
57117132	M13(Oxid	0	0	6.21E-16	108.22	2.64E-11	2	1546.7536	0.330588	26.89217	0
57117132		0	0	6.21E-16	97.06	1.96E-10	2	1701.7721	0.341424	27.63737	0
57117132		0	0	1.44E-15	89.46	1.76E-09	2	1530.7576	-0.35626	31.62952	0
57117132		0	0	6.21E-16	76.76	2.42E-08	3	2989.4165	-0.78754	31.18452	1
57117132		0	0	6.21E-16	73.21	4.76E-08	2	2762.2837	1.13953	32.07409	0
57117132	M20(Oxid	0	0	6.21E-16	69.99	1E-07	3	2964.39	-0.21877	22.7696	0
57117132		0	0	2.53E-08	61.4	1.81E-06	3	2791.3886	0.997243	32.88339	1
57117132	M26(Oxid	0	0	1.26E-13	59.11	1.22E-06	4	3681.723	0.466322	20.39136	1
57117132		0	0	1.04E-06	57.45	3.33E-06	2	2000.9864	-0.09774	27.57074	0
57117132		0	0	6.21E-16	55.97	2.53E-06	5	2918.3876	2.011111	30.69346	1

57117132		0	0	2.04E-11	54.08	8.01E-06	4	2191.1256	0.63883	33.65463	0
57117132		0	0	2.92E-06	53.64	8E-06	2	973.5421	-0.48326	17.58908	1
57117132		0	0	3.49E-05	49.49	2.19E-05	2	2454.2845	-1.27527	35.71847	0
57117132		0	0	8.77E-16	47.55	3.16E-05	5	3145.5211	0.353865	29.59298	2
57117132	M20(Oxid	0	0	6.21E-16	43.13	7.78E-05	4	3120.4931	0.418651	21.92896	1
57117132	M18(Oxid	0	0	0.0034	33.37	0.000713	3	2274.0838	0.782347	28.52223	1
57117132	M26(Oxid	0	0	1.65E-06	24.9	0.003228	5	3837.8187	-0.9676	20.0733	2

2	1025.485	-0.0009	0	29.25	0.0012	R	SYMEDLIR
2	1174.566	0.0008	0	60.17	9.60E-07	K	GSLFQYFADK
2	1379.804	-0.0027	0	28.69	0.0014	R	HYLDVLRPLVR
2	1481.751	-0.0018	0	45.98	2.50E-05	R	DLYAFIADIASQR
2	1552.785	-0.0019	0	94.83	4.10E-10	R	ALHAAATLEVDTDAR

Protein Group	Modificati	ΔCn	q-Value	PEP	IonScore	Exp Value	Charge	MH+ [Da]	ΔM [ppm]	RT [min]	Missed Cleavages
15608836		0	0	2.96E-13	103.02	9.48E-11	2	1492.7387	-0.28554	34.41907	0
15608836		0	0	4.65E-16	95.63	6.56E-10	2	2586.3482	-0.04994	35.40654	0
15608836		0	0	3.41E-14	89.7	2.36E-09	2	1749.876	-0.38125	27.98114	1
15608836		0	0	5.18E-12	88.21	1.51E-09	2	1454.6751	-0.51893	19.04716	0
15608836		0	0	4.65E-16	87.21	4.66E-09	3	2857.4731	-1.16701	35.02559	1
15608836		0	0	2.4E-06	77.59	4.01E-08	2	2051.0909	-0.60133	37.0105	1
15608836		0	0	1.23E-14	69.47	1.86E-07	2	1687.9235	0.561763	28.51091	0
15608836		0	0	7.96E-10	69.08	2.16E-07	3	1384.7281	-0.80071	16.10278	0
15608836		0	0	1.01E-09	67.18	4.79E-07	3	2677.367	-0.37826	38.6042	0
15608836		0	0	4.37E-15	66.59	6.58E-07	2	816.47301	0.376895	22.09373	0
15608836		0	0	0.002966	59.97	1.31E-06	3	3218.6667	0.431814	39.90472	0
15608836		0	0	4.65E-16	55.72	3.75E-06	2	1211.606	0.374156	24.10176	0
15608836		0	0	2.91E-13	55.14	4.13E-06	2	1628.9284	3.186773	27.7251	1
15608836	N-Term(A	0	0	4.65E-16	54.41	7.24E-06	2	1791.8989	6.470795	27.9955	1
15608836		0	0	0.007602	50.01	2.19E-05	2	1636.8282	-0.49407	25.73234	1
15608836		0	0	1.48E-07	39.02	0.000163	2	1023.5099	-0.63117	26.87124	0
15608836		0	0	0.003236	17.62	0.019028	4	4887.5358	-7.01048	43.03783	1
15608836		0	0.004	0.05616	57.4	4.09E-06	2	831.46807	-0.33209	18.12526	0
15608836		0	0.004	0.05616	54.29	1.01E-05	2	858.50505	0.770155	28.09748	0
15608836		0	0.004	0.0819	16.37	0.043828	3	2197.1837	1.829011	21.53947	1

Protein Group	Modificati	ΔCn	q-Value	PEP	IonScore	Exp Value	Charge	MH+ [Da]	ΔM [ppm]	RT [min]	# Missed Cleavages
15610082		0	0	1.94E-08	126.07	3.46E-13	2	2026.9712	-0.23301	38.13983	0
15610082		0	0	6.51E-07	116.88	5.95E-12	2	1604.8494	0.219044	29.56465	0
15610082		0	0	0.000124	108.78	2.71E-11	2	1947.0401	0.346866	32.10741	1

15610082		0	0	6.38E-06	82.28	1.54E-08	2	1559.827	-0.42274	34.22151	0
15610082		0	0	0.09387	80.35	1.48E-08	3	2967.477	2.497598	41.61825	1
15610082		0	0	5.14E-08	66.82	2.39E-07	3	3296.763	1.359922	47.6757	0
15610082		0	0	0.2062	65.57	5.13E-07	3	2101.1037	-0.63701	24.34963	0
15610082		0	0	0.01229	60.72	8.45E-07	2	1038.6052	-0.33811	21.89315	0
15610082	C3(Carban	0	0	0.002099	58.33	1.47E-06	2	1641.7316	-0.62758	26.1532	0
15610082		0	0	0.00206	53.66	9.26E-06	3	2558.2752	0.71938	34.25133	1
15610082		0	0	0.000198	50.92	1.21E-05	3	1781.9926	3.225377	27.92476	1
15610082	N-Term(A	0	0	0.4224	17.22	0.021812	3	3009.457	-7.67977	42.45839	1

Protein Group	Modificati	ΔCn	q-Value	PEP	IonScore	Exp Value	Charge	MH+ [Da]	ΔM [ppm]	RT [min]	# Missed Cleavages
57116716	M9(Oxidat	0	0	0.006548	123.01	4.99E-13	3	3079.2132	0.023919	30.08111	0
57116716	M13(Oxid	0	0	0.008038	101.91	6.43E-11	3	3564.4669	-1.70691	28.7685	1
57116716		0	0	0.04166	100.68	8.53E-11	3	3063.2184	0.057665	32.49559	0
57116716	M1(Oxidat	0	0	0.000921	77.69	1.7E-08	3	2461.0398	0.088489	24.47484	0
57116716	M14(Oxid	0	0	0.02982	71.06	7.82E-08	3	3720.5786	1.206828	28.54067	2
57116716		0	0	0.2538	63.78	7.75E-07	2	1151.6053	-0.2684	22.88452	0
57116716		0	0	0.08427	54.39	6.55E-06	2	1380.7591	-0.29006	22.87961	0
57116716		0	0	0.1587	48.56	2.51E-05	2	1644.8359	1.064987	31.43107	0
57116716	M1(Oxidat	0	0	0.1137	47.62	1.73E-05	3	2445.0478	1.254513	26.51631	0
57116716		0	0	0.2452	42.29	8.56E-05	2	1735.9795	-1.10688	24.80881	1

Rv1032c,193-1524	1332	52.1	193	1524	0.02	-	-	0.02	-	0.00%	-	-	0.00%	-
Rv1352,37-372	336	15.6	37	372	0.20	-	-	0.20	-	3.40%	-	-	3.40%	-
Rv1418,106-381	276	13.4	106	381	0.00	-	-	0.00	-	2.00%	-	-	2.00%	-
Rv2261c,1-309	309	14.6	1	309	0.00	-	-	0.00	-	4.10%	-	-	4.10%	-
Rv2318,64-1287	1224	48.2	64	1287	0.27	-	-	0.27	-	5.60%	-	-	5.60%	-
Rv2914c,253-1692	1440	56.1	253	1692	0.86	-	-	0.86	-	4.50%	-	-	4.50%	-
Rv3390,226-711	486	21.1	226	711	0.35	-	-	0.35	-	0.90%	-	-	0.90%	-
Rv3492c,67-327	261	12.9	67	327	0.13	-	-	0.13	-	0.60%	-	-	0.60%	-
Rv3604c,289-1152	864	35.0	289	1152	0.24	-	-	0.24	-	3.80%	-	-	3.80%	-
Rv3450c,223-1299	1077	42.8	223	1299	0.49	-	-	0.49	-	13.60%	-	-	13.60%	-
Rv0232,136-585	450	18.2	136	585	0.00	-	-	0.00	-	54.92%	-	55.00%	52.69%	0.00
Rv1698,58-828	771	31.57	58	828	0.13	-	-	0.13	-	1.00%	-	-	1.00%	-
Rv2945c,73-699	624	26.18	4	627	0.58	-	-	0.58	-	3.57%	30.12%	1.24%	11.64%	0.16

Supplemental file 1–Manually validated by MS/MS spectra

The files present all MS/MS spectra of peptides matching to seven proteins that had a single peptide hit in our study. Each entry is represented by peptide sequence, ions score, and MS/MS spectra of matching peptides.

Rv0402c, 118-573

Peptide sequence: RPSPLPTPIVPGLAPmPPGSVVPSSR

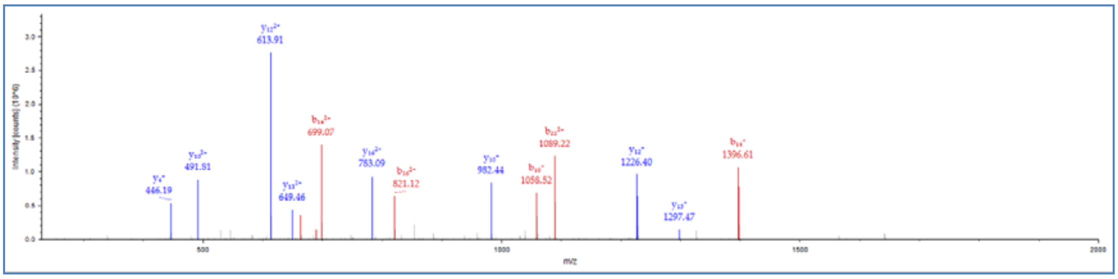

Ion Score: 66.07 Exp Value: 2.6E-07

#1	b^+	b^{2+}	b^{3+}	Seq.	y^+	y^{2+}	y^{3+}	#2
1	157.10840	79.05784	53.04098	R				26
2	254.16117	127.58422	85.39191	P	2466.34264	1233.67496	822.78573	25
3	341.19320	171.10024	114.40258	S	2369.28987	1185.14857	790.43481	24
4	438.24597	219.62662	146.75351	P	2282.25784	1141.63256	761.42413	23
5	551.33004	276.16866	184.44820	L	2185.20507	1093.10617	729.07321	22
6	648.38281	324.69504	216.79912	P	2072.12100	1036.56414	691.37852	21
7	749.43049	375.21888	250.48168	T	1975.06823	988.03775	659.02759	20
8	846.48326	423.74527	282.83260	P	1874.02055	937.51391	625.34503	19
9	959.56733	480.28730	320.52729	I	1776.96778	888.98753	592.99411	18
10	1058.63575	529.82151	353.55010	V	1663.88371	832.44549	555.29942	17
11	1155.68852	578.34790	385.90102	P	1564.81529	782.91128	522.27661	16
12	1212.70999	606.85863	404.90818	G	1467.76252	734.38490	489.92569	15
13	1325.79406	663.40067	442.60287	L	1410.74105	705.87416	470.91853	14
14	1396.83118	698.91923	466.28191	A	1297.65698	649.33213	433.22384	13
15	1493.88395	747.44561	498.63283	P	1226.61986	613.81357	409.54480	12
16	1640.91936	820.96332	547.64464	M-Oxidation	1129.56709	565.28718	377.19388	11
17	1737.97213	869.48970	579.99556	P	982.53167	491.76947	328.18207	10
18	1835.02490	918.01609	612.34648	P	885.47890	443.24309	295.83115	9
19	1892.04637	946.52682	631.35364	G	788.42613	394.71670	263.48023	8
20	1979.07840	990.04284	660.36432	S	731.40466	366.20597	244.47307	7
21	2078.14682	1039.57705	693.38712	V	644.37263	322.68995	215.46239	6
22	2177.21524	1089.11126	726.40993	V	545.30421	273.15574	182.43959	5
23	2274.26801	1137.63764	758.76085	P	446.23579	223.62153	149.41678	4
24	2361.30004	1181.15366	787.77153	S	349.18302	175.09515	117.06586	3
25	2448.33207	1224.66967	816.78221	S	262.15099	131.57913	88.05518	2
26				R	175.11896	88.06312	59.04450	1

Rv0996, 79-705

Peptide sequence: WGYLPPEGQGDDPDWKPEEDWR

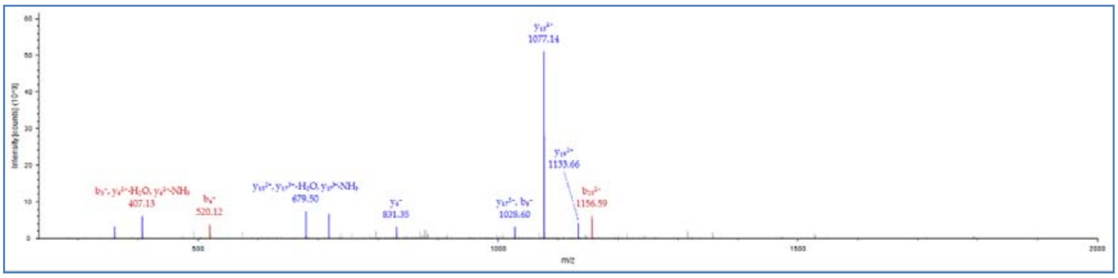

Ion Score: 33.04 Exp Value: 4.95E-04

#1	b^+	b^{2+}	b^{3+}	Seq.	y^+	y^{2+}	y^{3+}	#2
1	187.08660	94.04694	63.03372	W				22
2	244.10807	122.55767	82.04087	G	2486.08963	1243.54845	829.36806	21
3	407.17139	204.08933	136.39531	Y	2429.06816	1215.03772	810.36090	20
4	520.25546	260.63137	174.09000	L	2266.00484	1133.50606	756.00646	19
5	617.30823	309.15775	206.44093	P	2152.92077	1076.96402	718.31177	18
6	714.36100	357.68414	238.79185	P	2055.86800	1028.43764	685.96085	17
7	843.40360	422.20544	281.80605	E	1958.81523	979.91125	653.60993	16
8	900.42507	450.71617	300.81321	G	1829.77263	915.38995	610.59573	15
9	1028.48365	514.74546	343.49940	Q	1772.75116	886.87922	591.58857	14
10	1085.50512	543.25620	362.50656	G	1644.69258	822.84993	548.90238	13
11	1200.53207	600.76967	400.84887	D	1587.67111	794.33919	529.89522	12
12	1315.55902	658.28315	439.19119	D	1472.64416	736.82572	491.55290	11
13	1412.61179	706.80953	471.54211	P	1357.61721	679.31224	453.21059	10
14	1527.63874	764.32301	509.88443	D	1260.56444	630.78586	420.85966	9
15	1713.71806	857.36267	571.91087	W	1145.53749	573.27238	382.51735	8
16	1841.81303	921.41015	614.60919	K	959.45817	480.23272	320.49091	7
17	1938.86580	969.93654	646.96012	P	831.36320	416.18524	277.79258	6
18	2067.90840	1034.45784	689.97432	E	734.31043	367.65885	245.44166	5
19	2196.95100	1098.97914	732.98852	E	605.26783	303.13755	202.42746	4
20	2311.97795	1156.49261	771.33083	D	476.22523	238.61625	159.41326	3
21	2498.05727	1249.53227	833.35727	W	361.19828	181.10278	121.07094	2
22				R	175.11896	88.06312	59.04450	1

Supplemental Figures 1. The Western Blot result of 219 protein candidates in pulmonary TB patients.

1

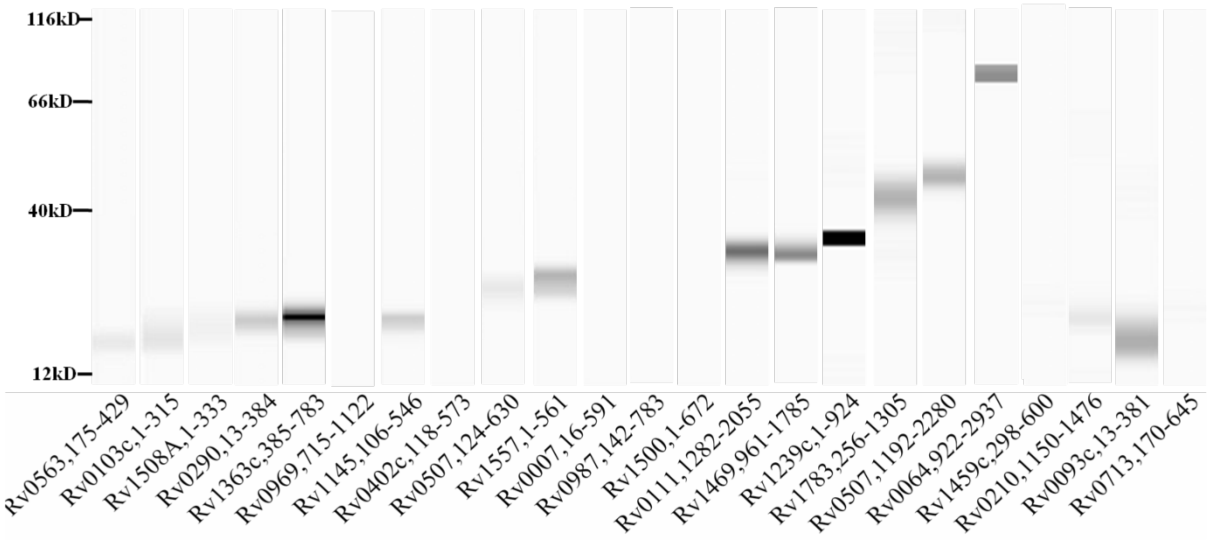

2

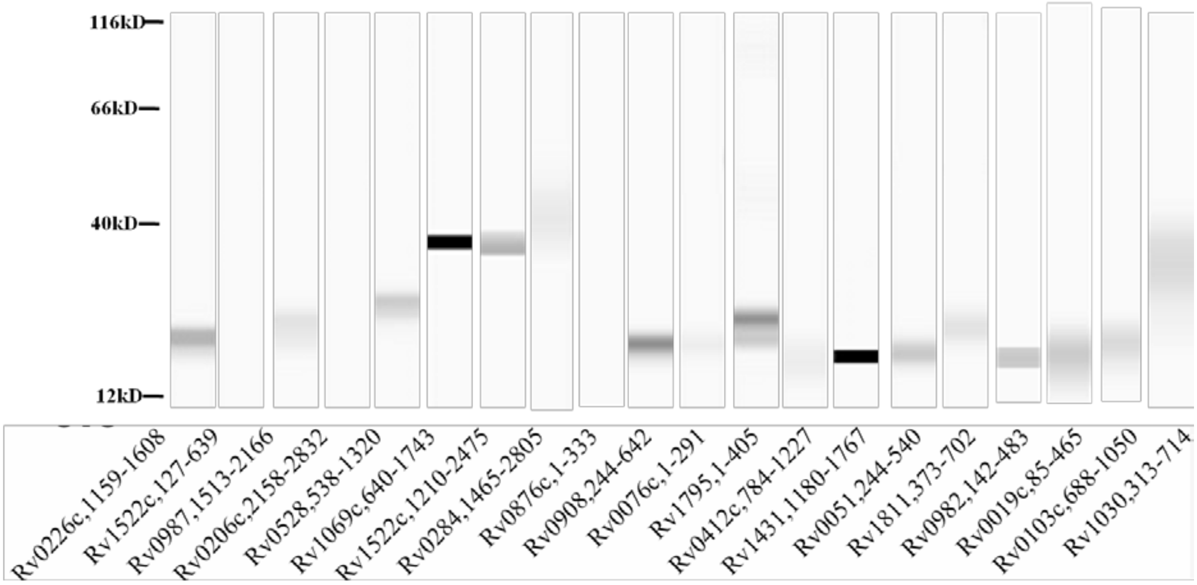

3

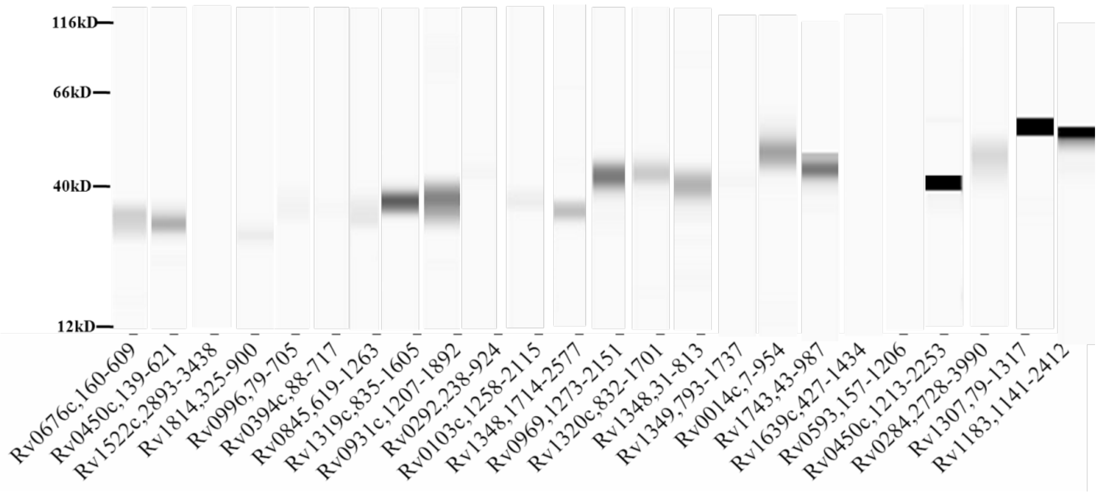

4

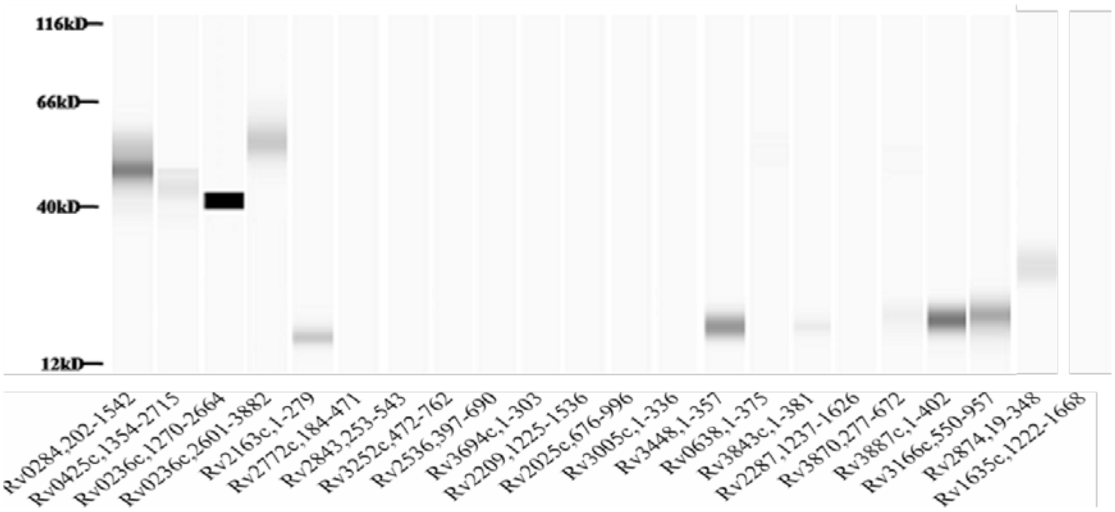
116kD
66kD
40kD
12kD
Rv0284,202-1542
Rv0425c,1354-2715
Rv0236c,1270-2664
Rv0236c,2601-3882
Rv2163c,1-279
Rv2772c,184-471
Rv2843,253-543
Rv3252c,472-762
Rv2536,397-690
Rv3694c,1-303
Rv2209,1225-1536
Rv2025c,676-996
Rv3005c,1-336
Rv3448,1-357
Rv0638,1-375
Rv3843c,1-381
Rv2287,1237-1626
Rv3870,277-672
Rv3887c,1-402
Rv3166c,550-957
Rv2874,19-348
Rv1635c,1222-1668

5

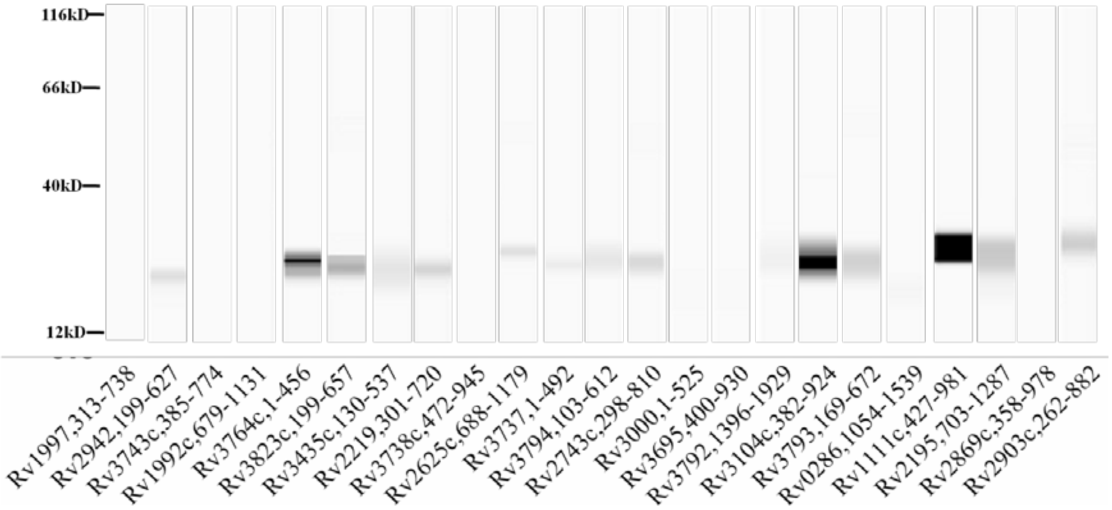
116kD
66kD
40kD
12kD
Rv1997,313-738
Rv2942,199-627
Rv3743c,385-774
Rv1992c,679-1131
Rv3764c,1-456
Rv3823c,199-657
Rv3435c,130-537
Rv2219,301-720
Rv3738c,472-945
Rv2625c,688-1179
Rv3737,1-492
Rv3794,103-612
Rv2743c,298-810
Rv3000,1-525
Rv3695,400-930
Rv3792,1396-1929
Rv3104c,382-924
Rv3793,169-672
Rv0286,1054-1539
Rv1111c,427-981
Rv2195,703-1287
Rv2869c,358-978
Rv2903c,262-882

6

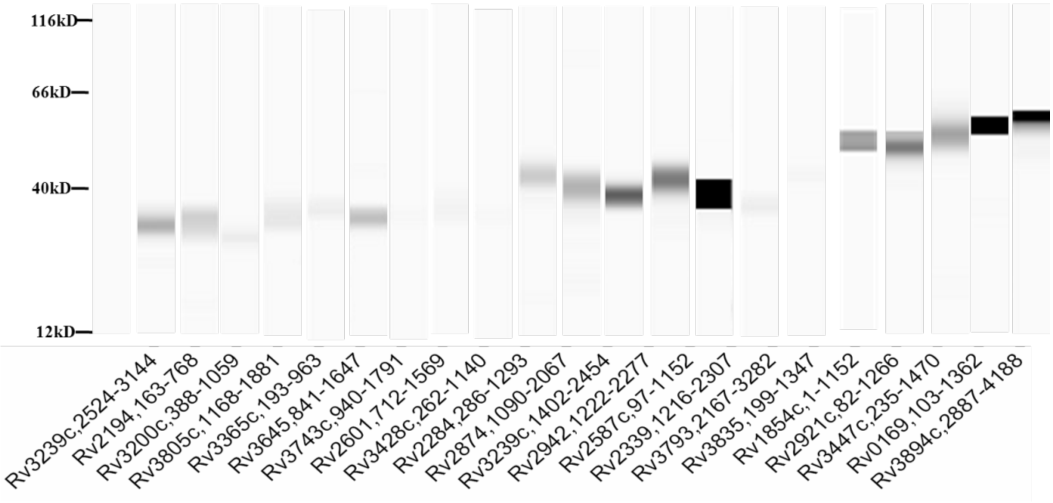
116kD
66kD
40kD
12kD
Rv3239c,2524-3144
Rv2194,163-768
Rv3200c,388-1059
Rv3805c,1168-1881
Rv3365c,193-963
Rv3645,841-1647
Rv3743c,940-1791
Rv2601,712-1569
Rv3428c,262-1140
Rv2284,286-1293
Rv2874,1090-2067
Rv3239c,1402-2454
Rv2942,1222-2277
Rv2587c,97-1152
Rv2339,1216-2307
Rv3793,2167-3282
Rv3835,199-1347
Rv1854c,1-1152
Rv2921c,82-1266
Rv3447c,235-1470
Rv0169,103-1362
Rv3894c,2887-4188

7

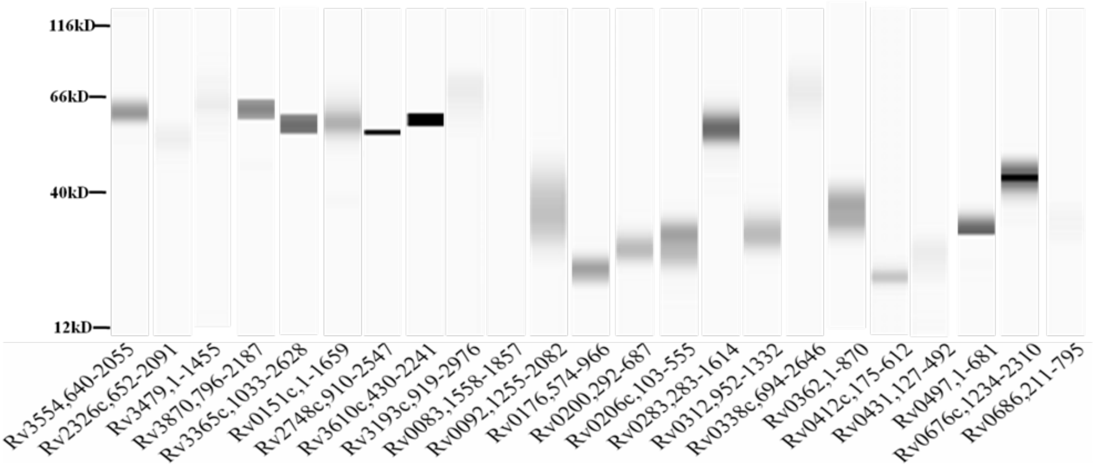
116kD
66kD
40kD
12kD
Rv3554,640-2055
Rv2326c,652-2091
Rv3479,1-1455
Rv3870,796-2187
Rv3365c,1033-2628
Rv0151c,1-1659
Rv2748c,910-2547
Rv3610c,430-2241
Rv3193c,919-2976
Rv0083,1558-1857
Rv0092,1255-2082
Rv0176,574-966
Rv0200,292-687
Rv0206c,103-555
Rv0283,283-1614
Rv0312,952-1332
Rv0338c,694-2646
Rv0362,1-870
Rv0412c,175-612
Rv0431,127-492
Rv0497,1-681
Rv0676c,1234-2310
Rv0686,211-795

8

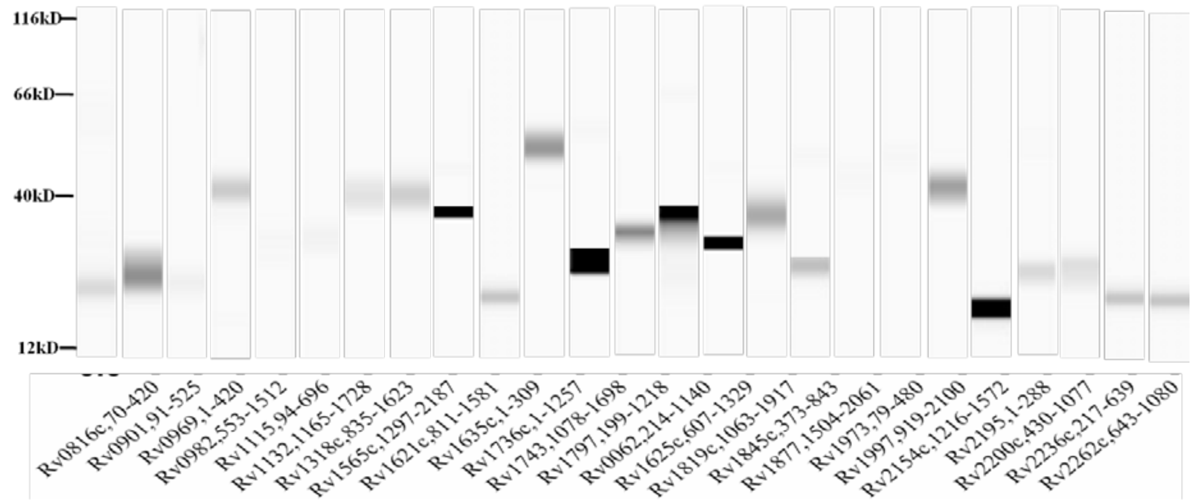
116kD
66kD
40kD
12kD
Rv0816c,70-420
Rv0901,91-525
Rv0969,1-420
Rv0982,553-1512
Rv1115,94-696
Rv1132,1165-1728
Rv1318c,835-1623
Rv1565c,1297-2187
Rv1621c,811-1581
Rv1635c,1-309
Rv1736c,1-1257
Rv1743,1078-1698
Rv1797,199-1218
Rv0062,214-1140
Rv1625c,607-1329
Rv1819c,1063-1917
Rv1845c,373-843
Rv1877,1504-2061
Rv1973,79-480
Rv1997,919-2100
Rv2154c,1216-1572
Rv2195,1-288
Rv2200c,430-1077
Rv2236c,217-639
Rv2262c,643-1080

9

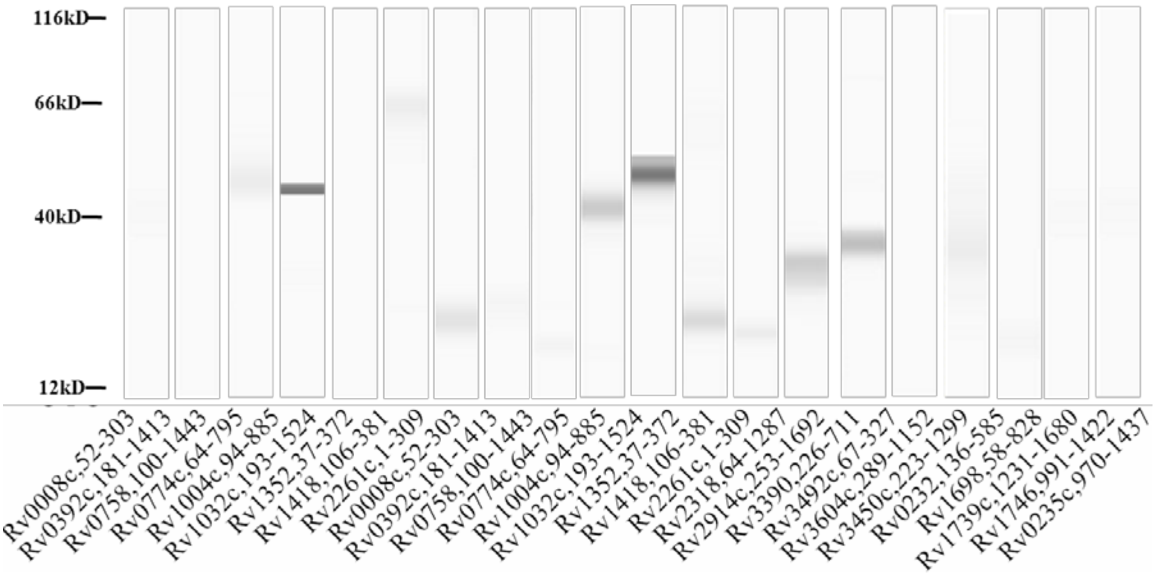
116kD
66kD
40kD
12kD
Rv0008c,52-303
Rv0392c,181-1413
Rv0758,100-1443
Rv0774c,64-795
Rv1004c,94-885
Rv1032c,193-1524
Rv1352,37-372
Rv1418,106-381
Rv2261c,1-309
Rv0008c,52-303
Rv0392c,181-1413
Rv0758,100-1443
Rv0774c,64-795
Rv1004c,94-885
Rv1032c,193-1524
Rv1352,37-372
Rv1418,106-381
Rv2261c,1-309
Rv2318,64-1287
Rv2914c,253-1692
Rv3390,226-711
Rv3492c,67-327
Rv3604c,289-1152
Rv3450c,223-1299
Rv0232,136-585
Rv1698,58-828
Rv1739c,1231-1680
Rv1746,991-1422
Rv0235c,970-1437

10

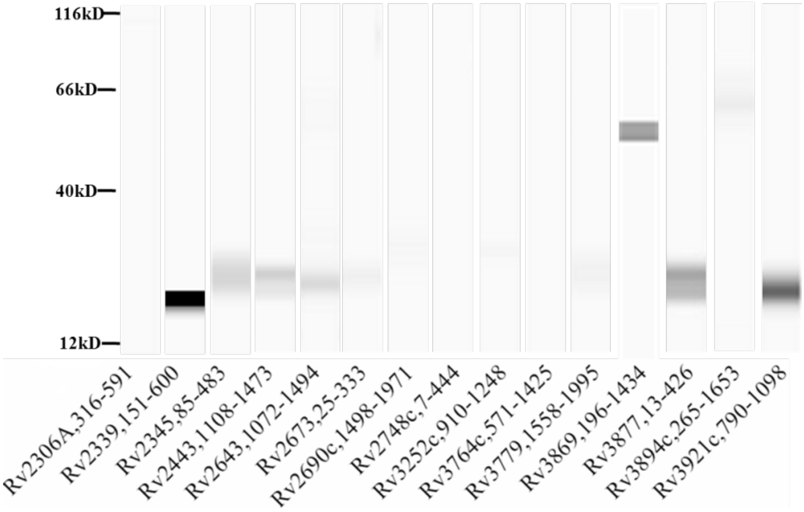

Second round

2. The Western Blot result of 52 protein candidates in pulmonary TB patients.

1

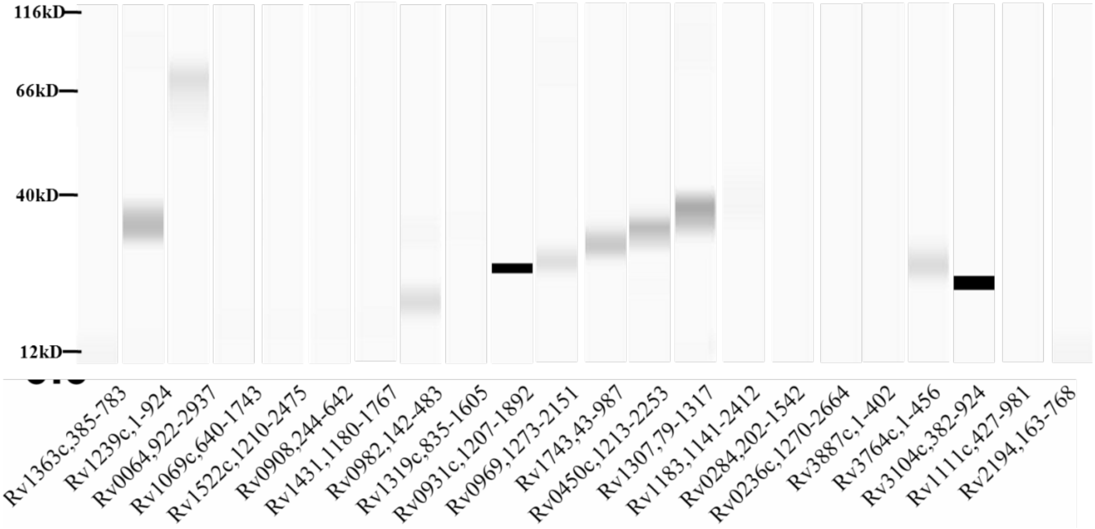

2

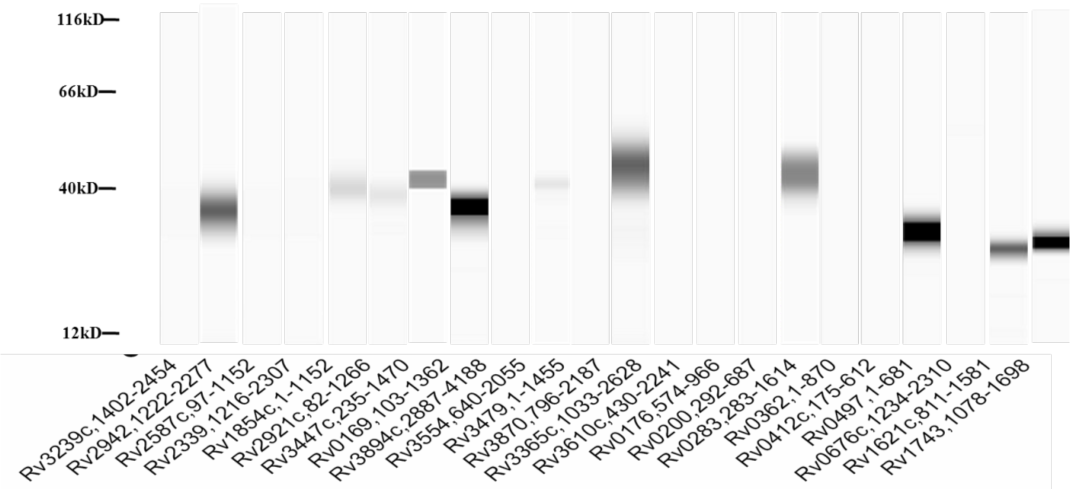

3

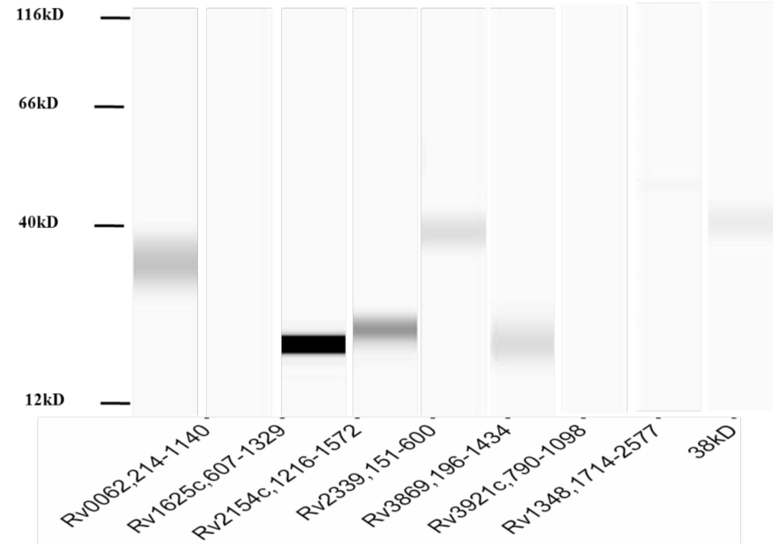

Third round

3. The Western Blot result of 12 protein candidates in pulmonary TB patients.

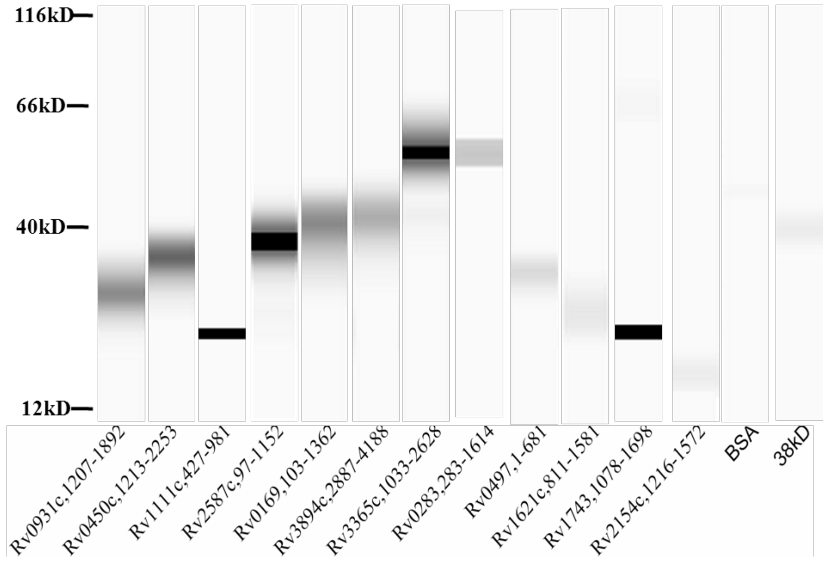

ELISPOT Confirmation file				
ORF	Positive rate	average Intensity of reactivity	spot of cell format	Product
Rv0232	53.33%	42.15%	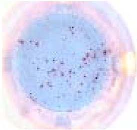	TETR/ACRR FAMILY TRANSCRIPTIONAL REGULATOR
Rv1115	46.67%	43.62%	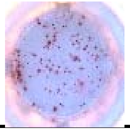	POSSIBLE CONSERVED LIPOPROTEIN LPPH
Rv0284	40.00%	26.55%	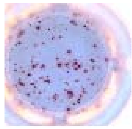	POSSIBLE CONSERVED MEMBRANE PROTEIN
Rv1145	46.67%	22.20%	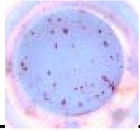	PROBABLE CONSERVED TRANSMEMBRANE TRANSPORT PROTEIN MMPL13A
Rv0987	20.0%	23.38%	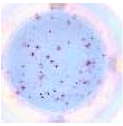	PROBABLE ADHESION COMPONENT TRANSPORT TRANSMEMBRANE PROTEIN ABC TRANSPORTER
Rv1783	26.67%	24.34%	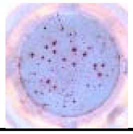	PROBABLE CONSERVED MEMBRANE PROTEIN
Rv3447c	20.0%	23.84%	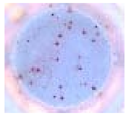	PROBABLE CONSERVED MEMBRANE PROTEIN
Rv1500	26.67%	14.52%	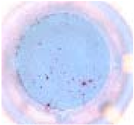	PROBABLE CATION TRANSPORTER P-TYPE ATPASE CTPJ

8 Proteins SFC with ESAT-6 positive patient															
ORF	P*-1	P-2	P-3	P-4	P-5	P-6	P-7	P-8	P-9	P-10	P-11	P-12	P-13	P-14	P-15
Rv0232	20	11	9	35	10	8	83	7	57	67	44	28	0	11	48
Rv0284	16	10	28	10	7	113	28	19	16	44	5	22	33	18	43
Rv0987	7	9	19	2	4	80	18	4	14	42	5	11	34	15	27
Rv1115	18	7	13	10	23	78	37	6	24	27	0	27	29	15	25
Rv1145	8	9	26	7	10	74	22	6	7	33	6	23	37	19	31
Rv1500	15	5	4	5	14	47	23	9	5	24	8	15	37	11	23
Rv1783	7	9	25	2	15	66	16	12	10	48	10	16	21	17	27
Rv3447c	7	7	18	2	8	65	19	16	18	31	15	5	26	16	45
ESTA-6 KIT	138	115	109	121	272	197	62	42	185	122	182	192	288	244	117
ESTA-6 EXP	67	66	136	109	176	153	97	22	145	44	185	219	234	212	226

*:ESAT-6 positive patient

8 Proteins SFC with ESAT-6 negative patient								
ORF	N*-1	N-2	N-3	N-4	N-5	N-6	N-7	N-8
Rv0232	29	4	7	20	13	6	39	3
Rv0284	0	0	9	63	15	25	7	0
Rv0987	0	0	8	56	7	20	7	0
Rv1115	11	0	4	52	24	18	3	0
Rv1145	7	0	4	56	14	24	11	1
Rv1500	0	0	3	40	6	23	4	0
Rv1783	2	0	4	53	16	25	7	0
Rv3447c	0	0	1	46	14	28	15	1
ESTA-6 KIT	10	17	30	5	27	15	6	3
ESTA-6 EXP	15	19	1	71	114	23	2	0

*:ESAT-6nagetive patients

8 Proteins SFC with ESAT-6 negative patient					
ORF	candidate 1	candidate 2	candidate 3	candidate 4	candidate 5
Rv0232	2	6	5	9	6
Rv0284	4	0	1	5	4
Rv0987	6	1	0	1	1
Rv1115	2	2	0	0	1
Rv1145	5	0	0	2	1
Rv1500	3	0	0	1	4
Rv1783	6	0	0	1	3
Rv3447c	5	0	0	0	3
ESTA-6 KIT	23	7	6	7	0
ESTA-6 EXP	11	6	24	0	2

The data of IFN-γ were stimulation by proteins.			
	CD4+ , IFN-r+	CD4+ , IFN-r+	CD4+ , IFN-r+
PBS-N-γ.fcs	0	0	0
PBS-P-γ.fcs	0.092	0.056	0.044
PBS-Ag-γ.fcs	0.051	0.012	0.766
OVA-N-γ.fcs	0	0	0
OVA-P-γ.fcs	0.572	0.035	0.713
OVA-Ag-γ.fcs	0.617	0.077	0.303
Rv3875-N-γ.fcs	0	0	0
Rv3875-P-γ.fcs	2.05	1.664	1.478
Rv3875-Ag-γ.fcs	0.324	0.188	0.118
Rv0232-N-γ.fcs	0	0	0
Rv0232-P-γ.fcs	1.809	0.242	1.741
Rv0232-Ag-γ.fcs	0.129	0.482	0.04
Rv1811N-γ.fcs	0	0	0
Rv1811P-γ.fcs	1.685	0.018	1.7
Rv1811Ag-γ.fcs	0.108	0.159	0.19
Rv1115N-γ.fcs	0	0	0
Rv1115P-γ.fcs	1.35	0.398	0.15
Rv1115Ag-γ.fcs	0.28	0.181	1.64

N：PBS stimulate

P：PHA stimulate

Ag：Protein stimulate

The data of IFN-γ were stimulation by proteins.			
	CD8+ , IFN-r+	CD8+ , IFN-r+	CD8+ , IFN-r+
PBS-N-γ.fcs	0	0	0
PBS-P-γ.fcs	0.195	1.343	0.73
PBS-Ag-γ.fcs	0.071	0.084	0.066
OVA-N-γ.fcs	0	0	0
OVA-P-γ.fcs	0.251	1.217	0.045
OVA-Ag-γ.fcs	0.126	0.535	0.045
Rv3875-N-γ.fcs	0	0	0
Rv3875-P-γ.fcs	1.302	2.347	2.328
Rv3875-Ag-γ.fcs	0.237	1.217	0.308
Rv0232-N-γ.fcs	0	0	0
Rv0232-P-γ.fcs	0.776	3.629	0.5
Rv0232-Ag-γ.fcs	0.456	0.177	0.05
Rv1811N-γ.fcs	0	0	0
Rv1811P-γ.fcs	0.161	3.12	0.897
Rv1811Ag-γ.fcs	0.367	0.054	0.031
Rv1115N-γ.fcs	0	0	0
Rv1115P-γ.fcs	0.318	1.404	0.47
Rv1115Ag-γ.fcs	0.337	0.006	0.029

N：PBS stimulate

P：PHA stimulate

Ag：Protein stimulate

The data of IL-2 were stimulation by proteins.			
	CD4+ , IL-2+	CD4+ , IL-2+	CD4+ , IL-2+
PBS-N-2.fcs	0	0	0
PBS-P-2.fcs	0.05	0.059	0.013
PBS-Ag-2.fcs	0.001	0.033	0.028
OVA-N-2.fcs	0	0	0
OVA-P-2.fcs	0.045	0.081	0.028
OVA-Ag-2.fcs	0.087	0.076	0.026
Rv3875-N-2.fcs	0	0	0
Rv3875-P-2.fcs	0.487	1.279	0.256
Rv3875-Ag-2.fcs	0.14	0.121	0.122
Rv0232-N-2.fcs	0	0	0
Rv0232-P-2.fcs	0.152	0.093	0.115
Rv0232-Ag-2.fcs	0.12	0.075	0.122
Rv1811N-2.fcs	0	0	0
Rv1811P-2.fcs	0.286	0.169	0.036
Rv1811Ag-2.fcs	0.069	0.175	0.074
Rv1115-N-2.fcs	0	0	0
Rv1115-P-2.fcs	0.192	0.095	0.233
Rv1115-Ag-2.fcs	0.079	0.077	0.085

N：PBS stimulate

P：PHA stimulate

Ag：Protein stimulate

The data of IL-2 were stimulation by proteins.			
	CD8+ , IL-2+	CD8+ , IL-2+	CD8+ , IL-2+
PBS-N-2.fcs	0	0	0
PBS-P-2.fcs	0.06	0.076	0.013
PBS-Ag-2.fcs	0.037	0.031	0.049
OVA-N-2.fcs	0	0	0
OVA-P-2.fcs	0.059	0.094	0.058
OVA-Ag-2.fcs	0.023	0.071	0.021
Rv3875-N-2.fcs	0	0	0
Rv3875-P-2.fcs	0.55	0.21	0.165
Rv3875-Ag-2.fcs	0.19	0.135	0.179
Rv0232-N-2.fcs	0	0	0
Rv0232-P-2.fcs	0.218	0.13	0.11
Rv0232-Ag-2.fcs	0.06	0.036	0.177
Rv1811N-2.fcs	0	0	0
Rv1811P-2.fcs	0.253	0.054	0.345
Rv1811Ag-2.fcs	0.083	0.18	0.104
Rv1115-N-2.fcs	0	0	0
Rv1115-P-2.fcs	0.087	0.107	0.244
Rv1115-Ag-2.fcs	0.069	0.175	0.185

N：PBS stimulate

P：PHA stimulate

Ag：Protein stimulate

The fig results of IFN-γ and IL-2 were stimulation by proteins.

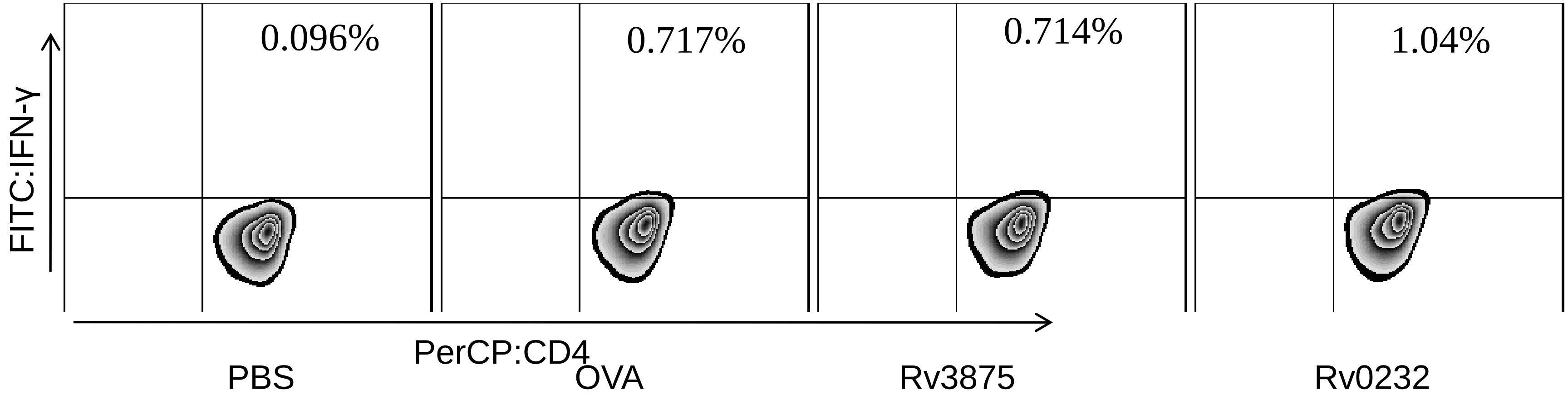

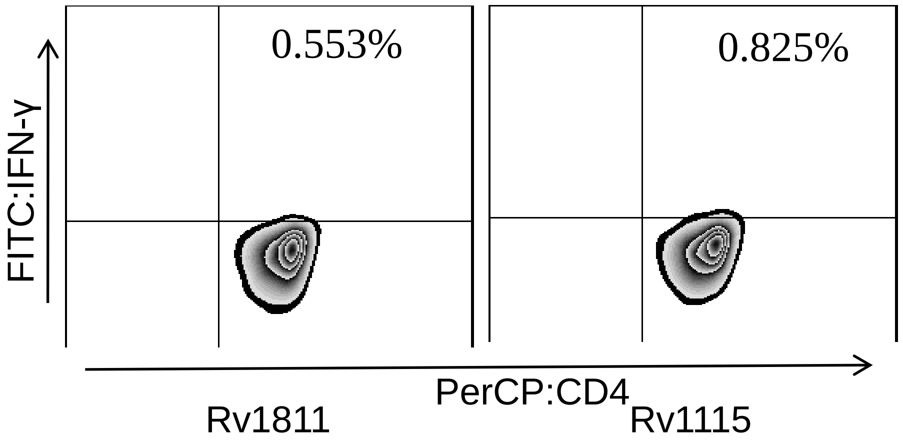

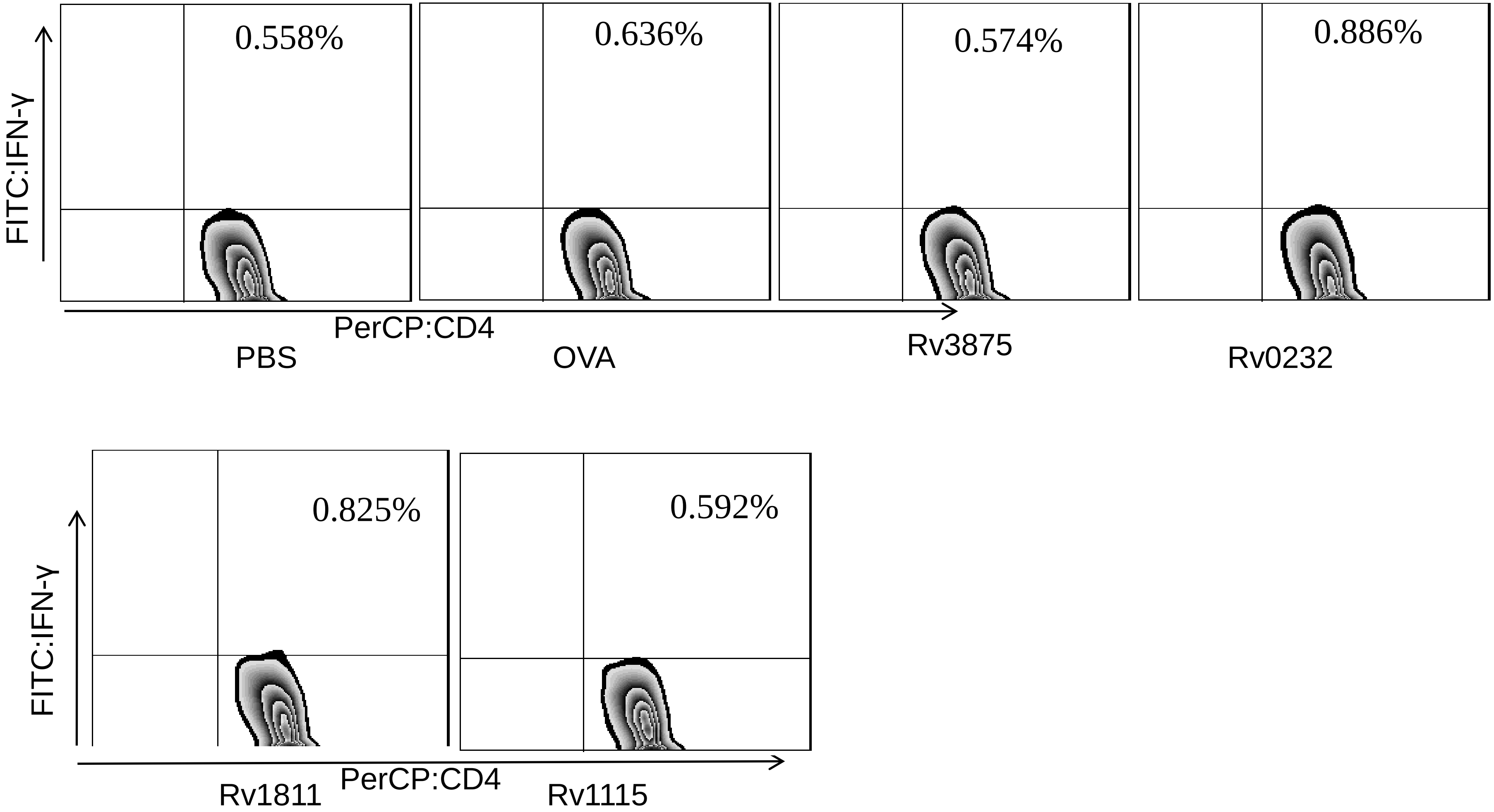

FITC:IFN-γ
0.558%
0.636%
0.574%
0.886%
PerCP:CD4
PBS
OVA
Rv3875
Rv0232
FITC:IFN-γ
0.825%
0.592%
PerCP:CD4
Rv1811
Rv1115

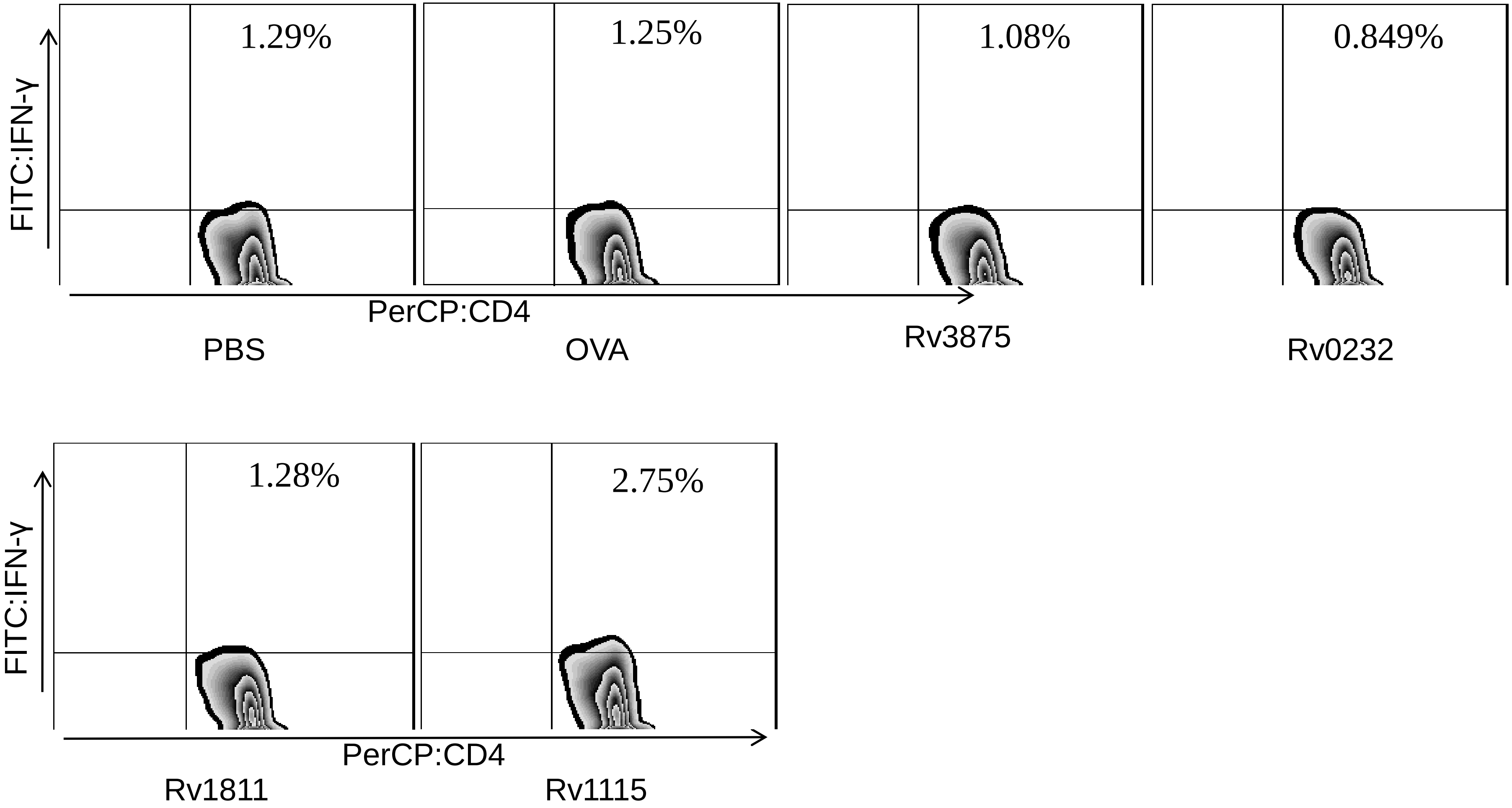
1.29%
1.25%
1.08%
0.849%
FITC:IFN-γ
PerCP:CD4
PBS
OVA
Rv3875
Rv0232
1.28%
2.75%
FITC:IFN-γ
PerCP:CD4
Rv1811
Rv1115

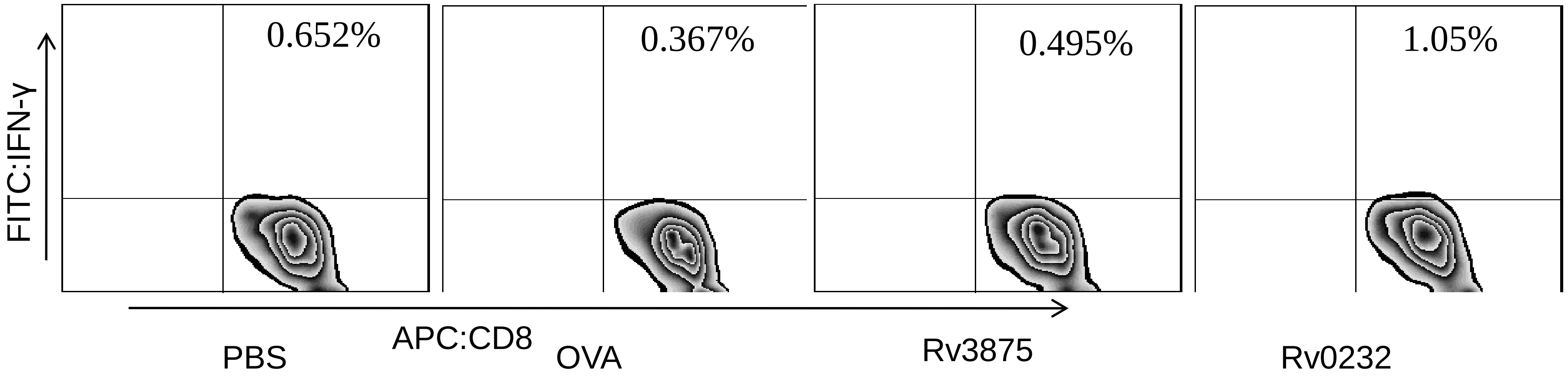
0.652%
0.367%
0.495%
1.05%
FITC:IFN-γ
APC:CD8
PBS
OVA
Rv3875
Rv0232

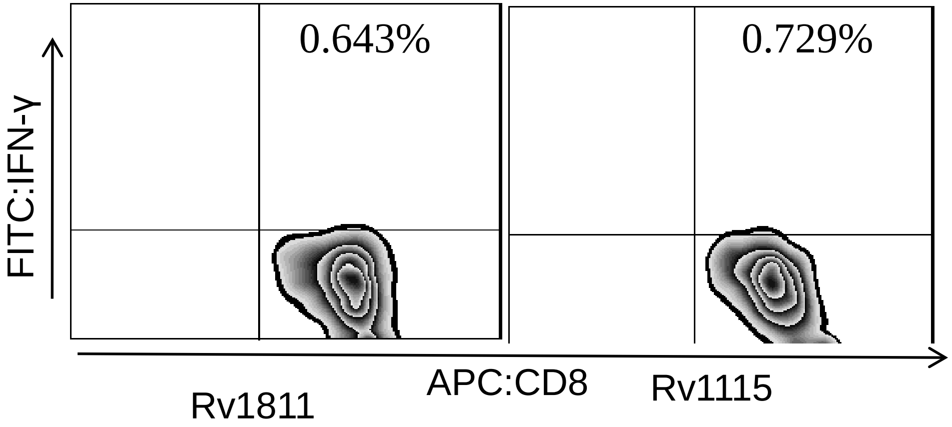
0.643%
0.729%
FITC:IFN-γ
APC:CD8
Rv1811
Rv1115

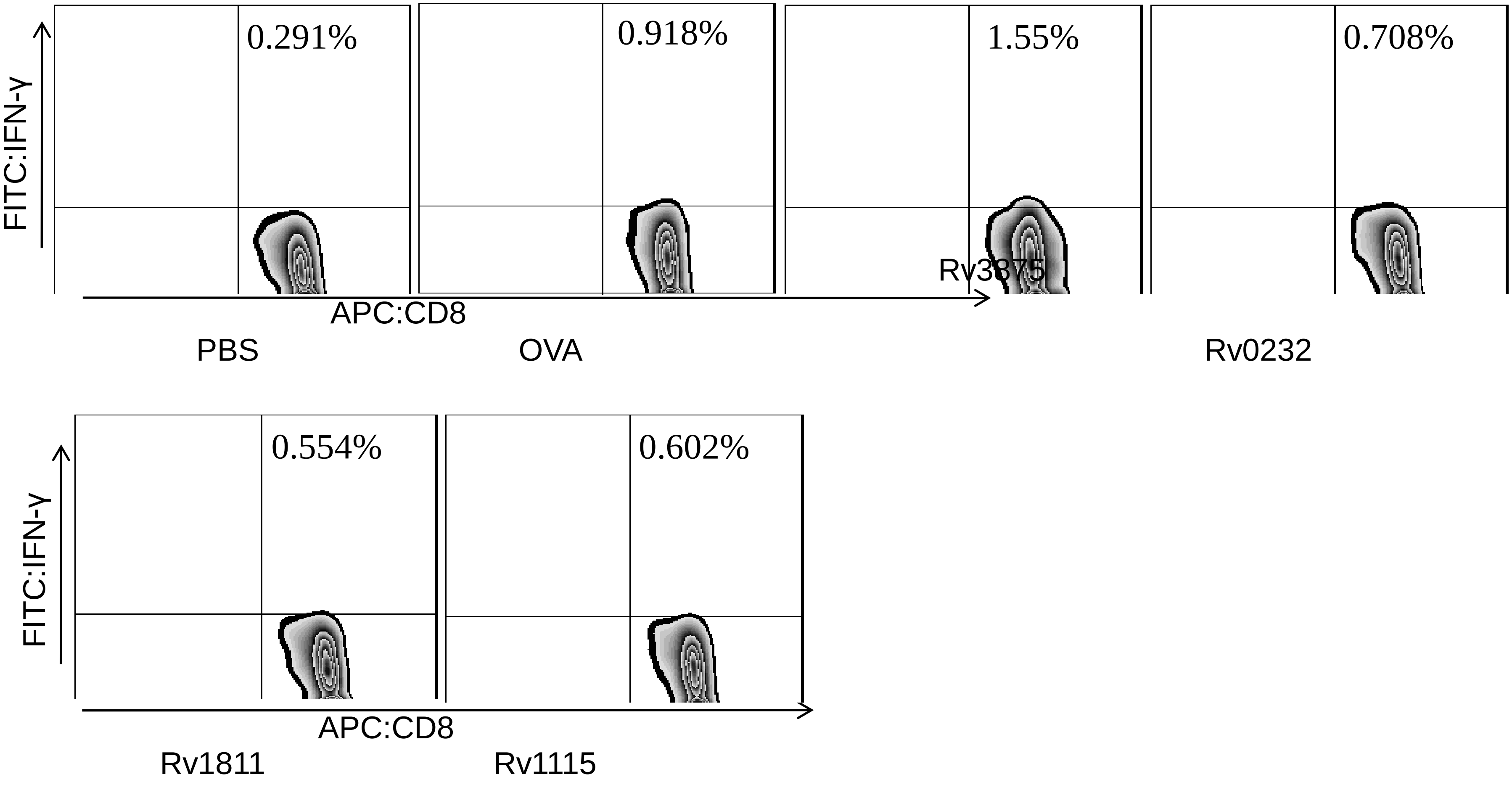
FITC:IFN-γ
0.291%
0.918%
1.55%
0.708%
Rv3875
APC:CD8
PBS
OVA
Rv0232
FITC:IFN-γ
0.554%
0.602%
APC:CD8
Rv1811
Rv1115

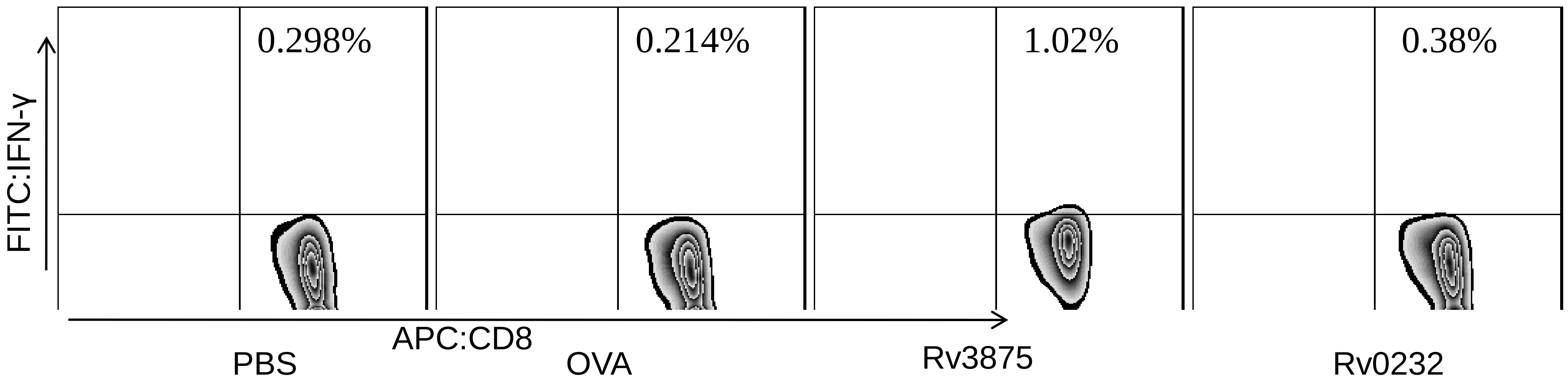
0.298%
0.214%
1.02%
0.38%
FITC:IFN-γ
APC:CD8
PBS
OVA
Rv3875
Rv0232

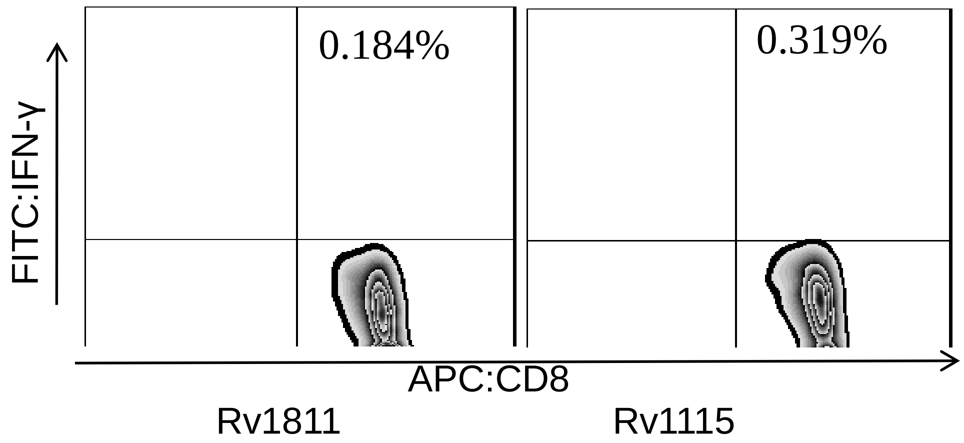
0.184%
0.319%
FITC:IFN-γ
APC:CD8
Rv1811
Rv1115

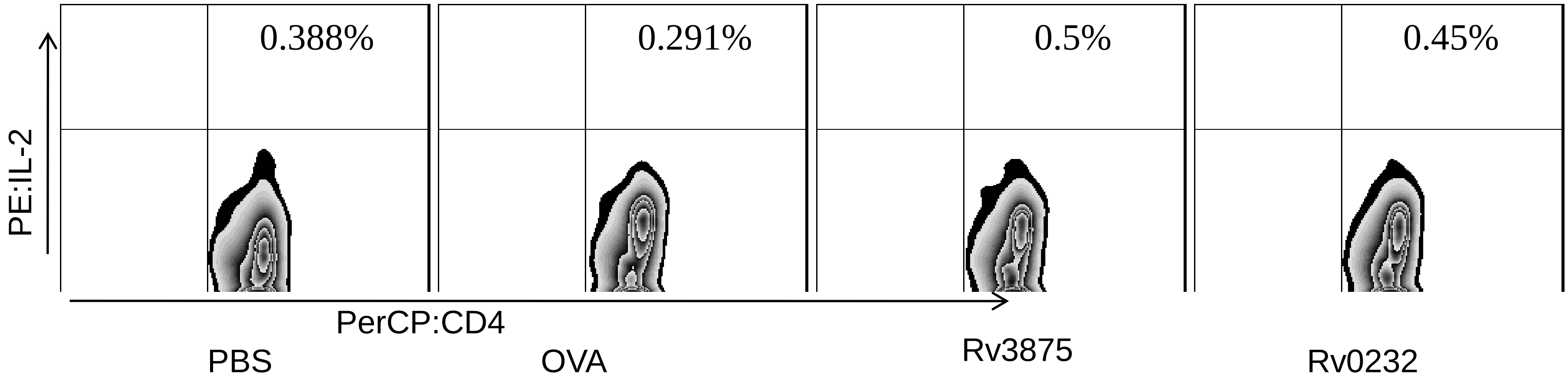

0.388%
0.291%
0.5%
0.45%
PE:IL-2
PerCP:CD4
PBS
OVA
Rv3875
Rv0232

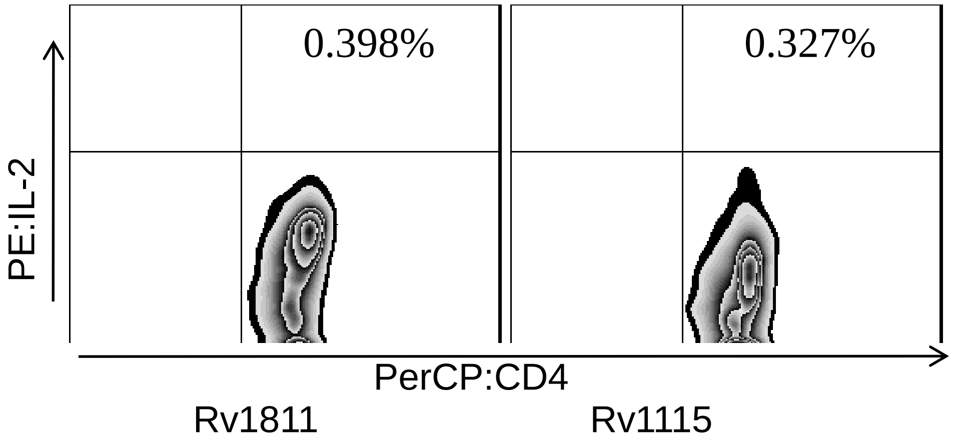

0.398%
0.327%
PE:IL-2
PerCP:CD4
Rv1811
Rv1115

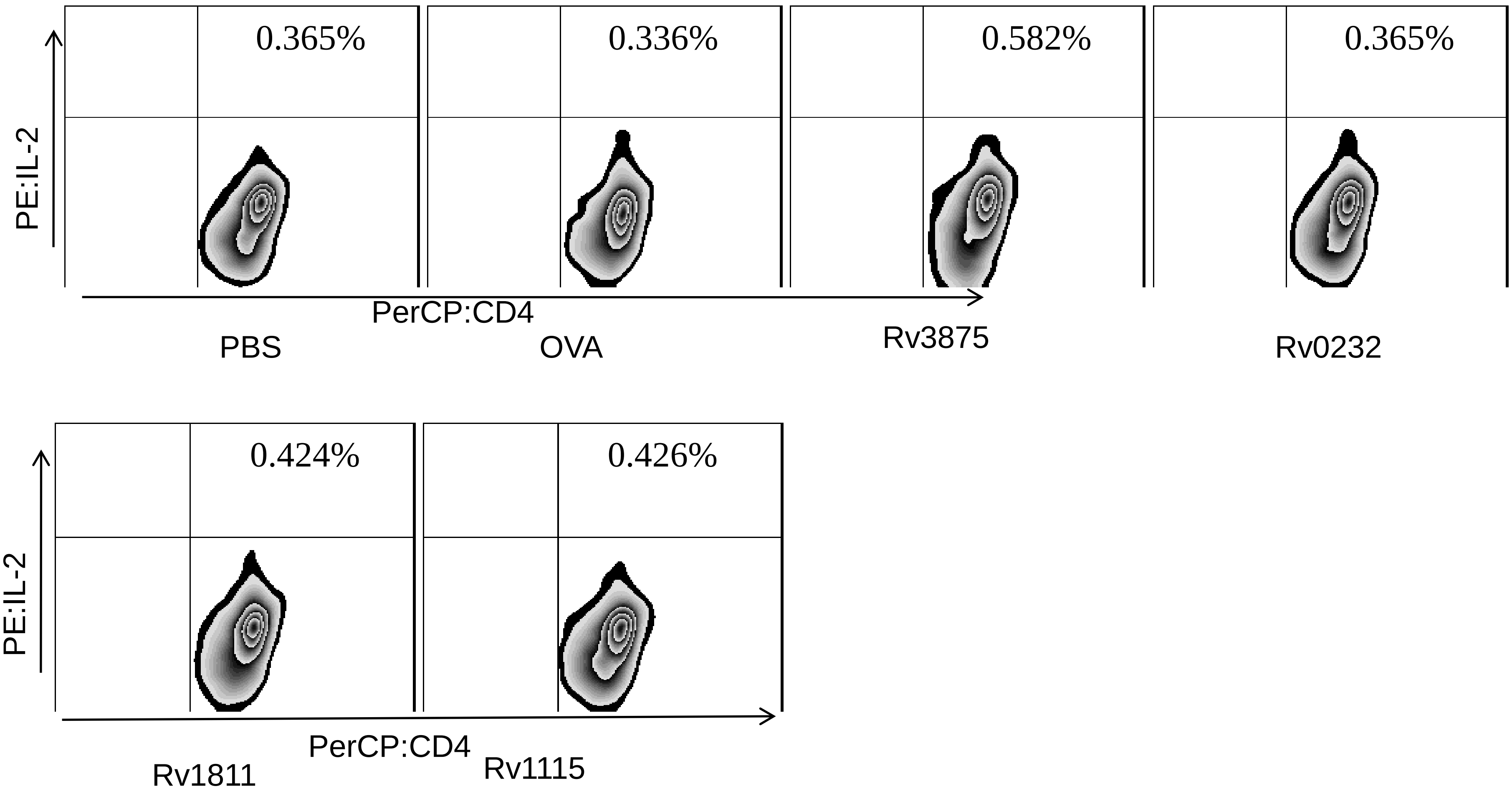
0.365%
0.336%
0.582%
0.365%
PE:IL-2
PerCP:CD4
PBS
OVA
Rv3875
Rv0232
0.424%
0.426%
PE:IL-2
PerCP:CD4
Rv1811
Rv1115

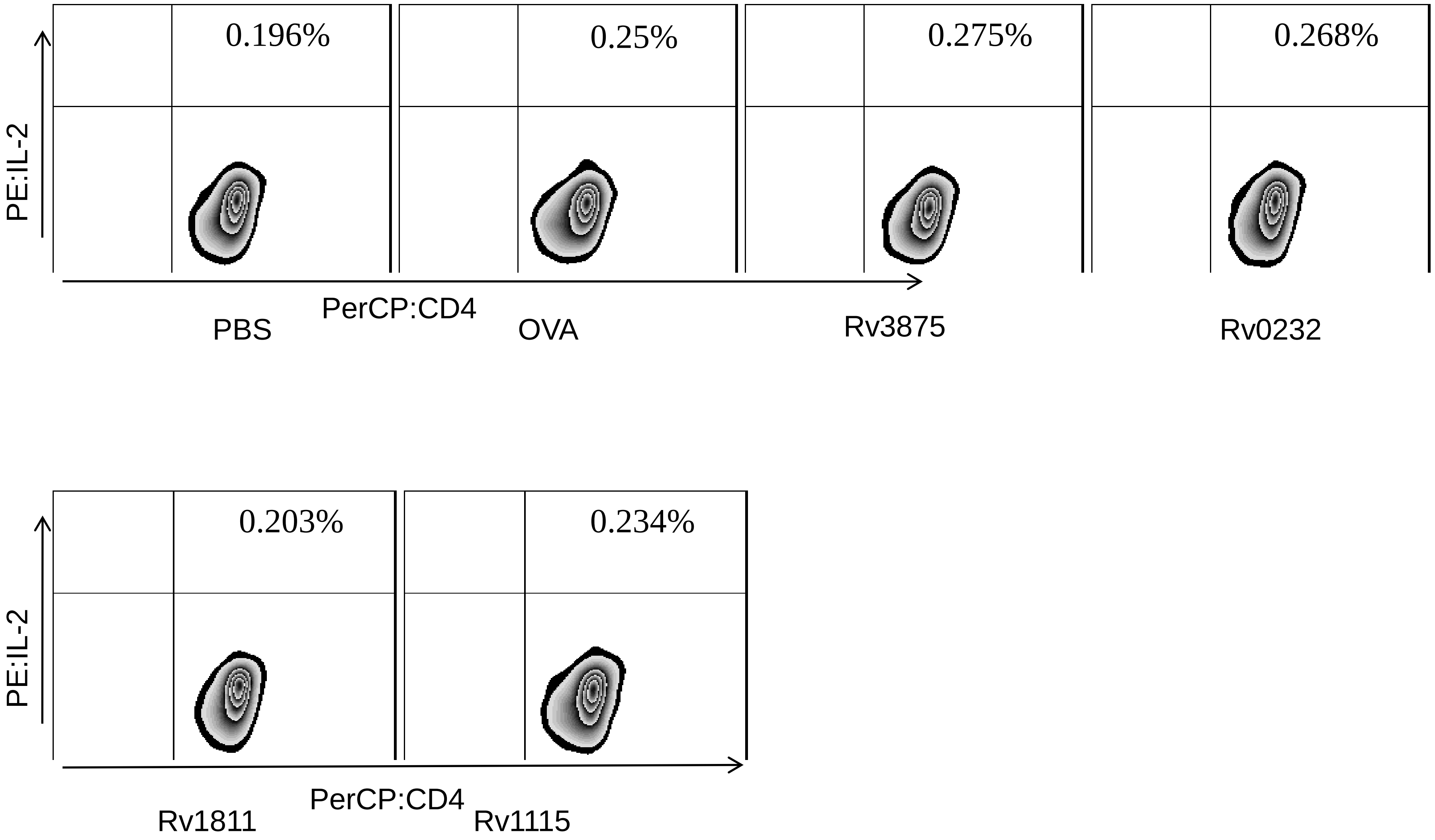
0.196%
0.25%
0.275%
0.268%
PE:IL-2
PerCP:CD4
PBS
OVA
Rv3875
Rv0232
0.203%
0.234%
PE:IL-2
PerCP:CD4
Rv1811
Rv1115

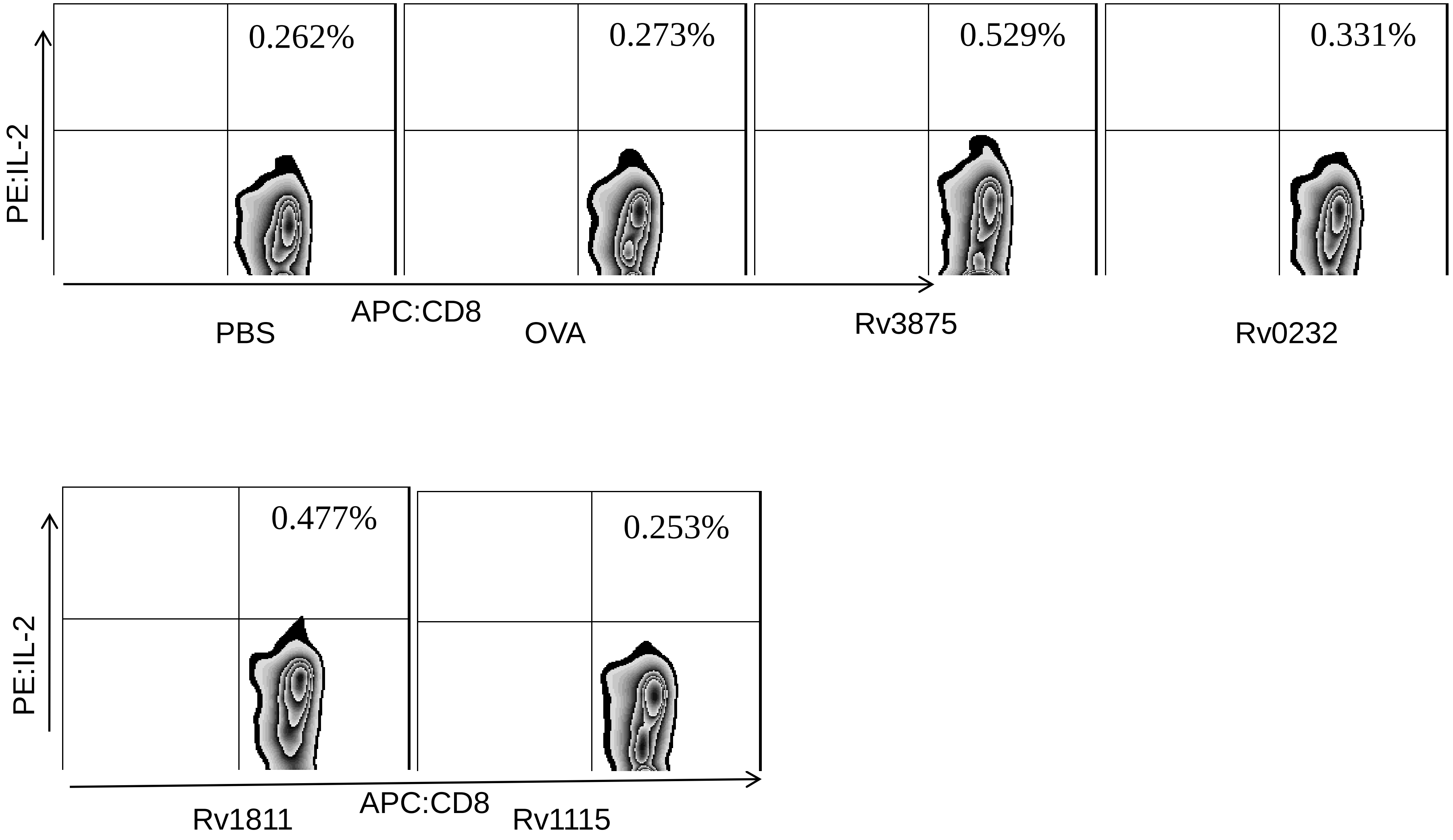

0.262%
0.273%
0.529%
0.331%
PE:IL-2
APC:CD8
PBS
OVA
Rv3875
Rv0232
0.477%
0.253%
PE:IL-2
APC:CD8
Rv1811
Rv1115

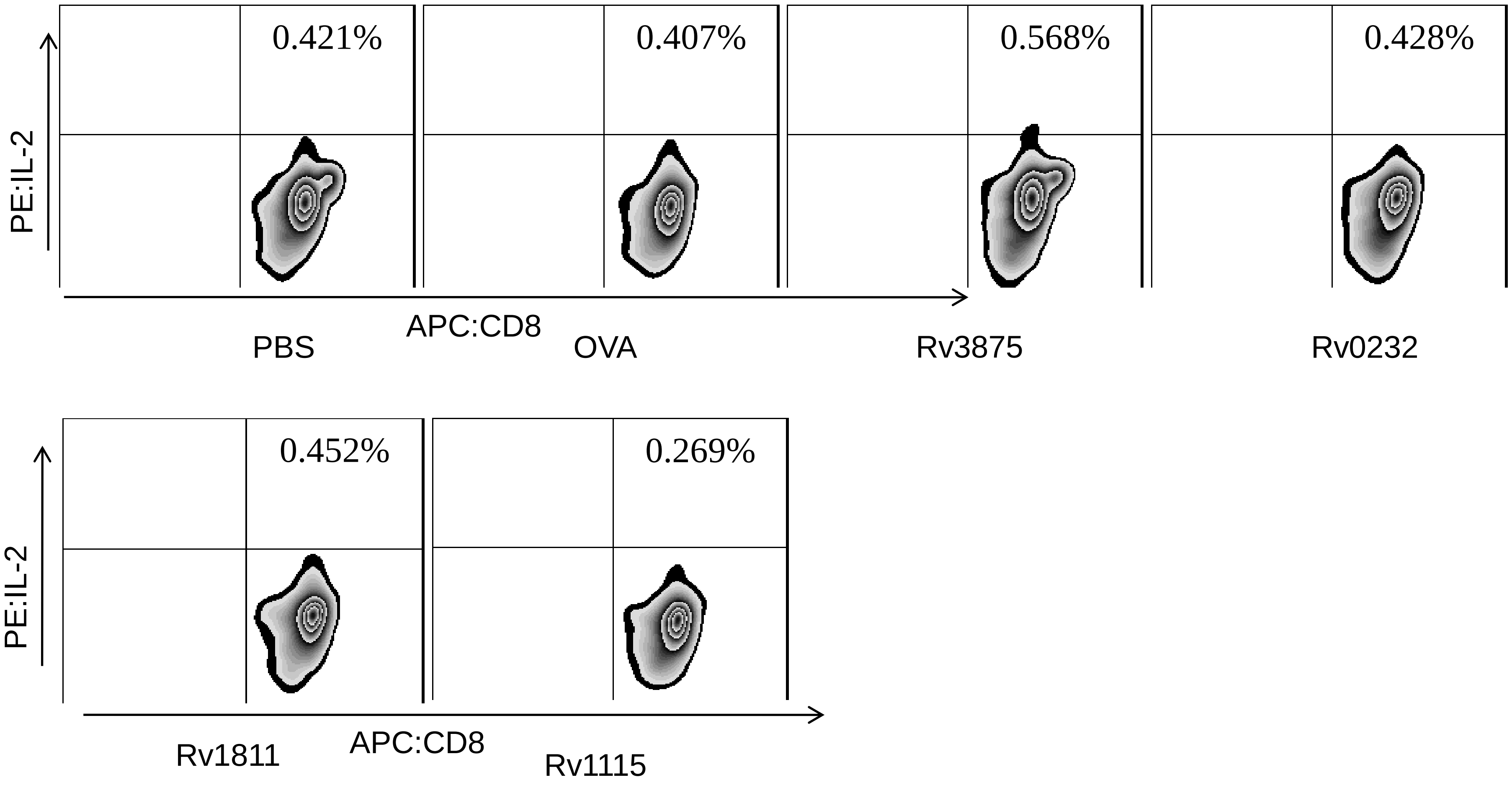
0.421%
0.407%
0.568%
0.428%
PE:IL-2
APC:CD8
PBS
OVA
Rv3875
Rv0232
0.452%
0.269%
PE:IL-2
APC:CD8
Rv1811
Rv1115

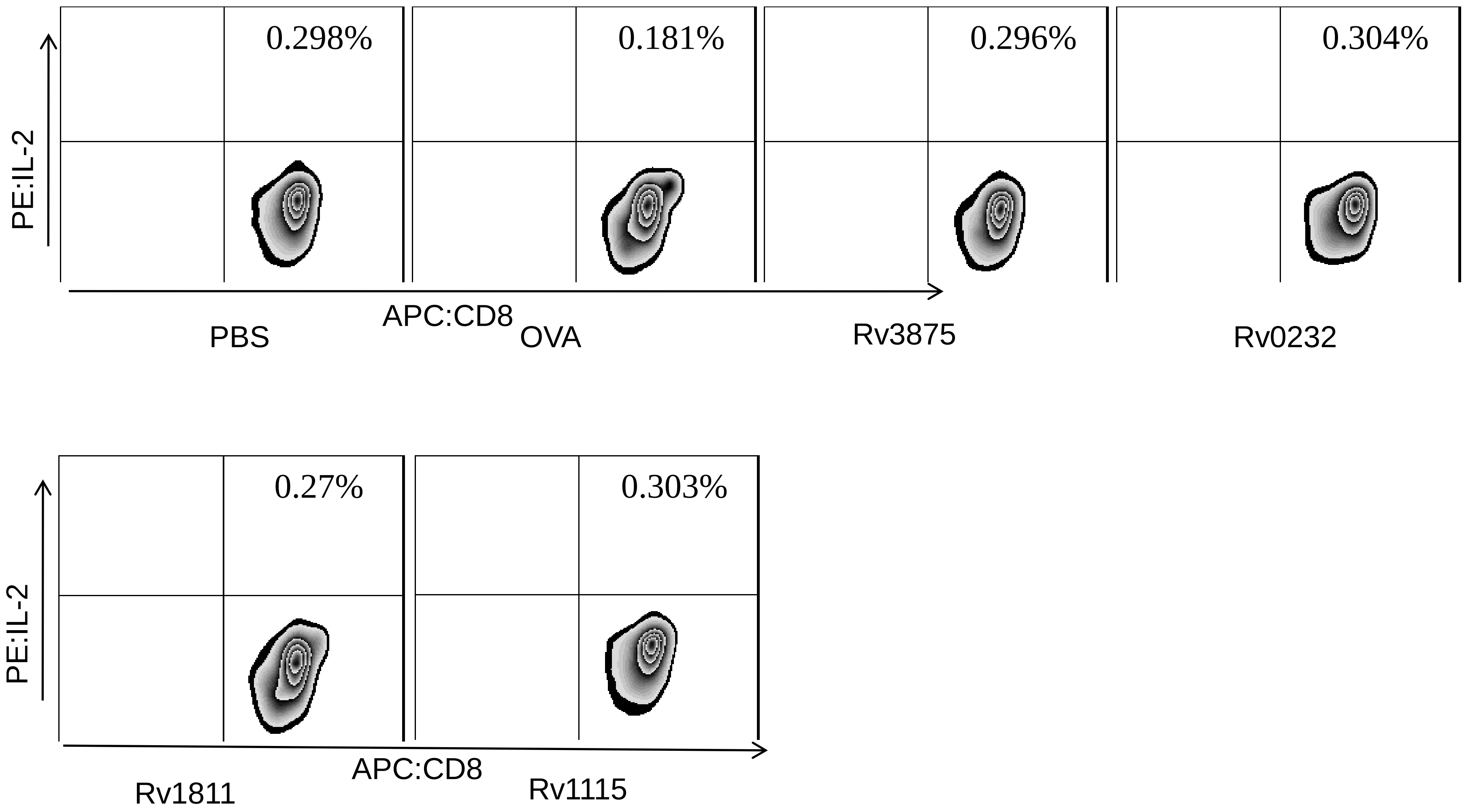
PE:IL-2
0.298%
0.181%
0.296%
0.304%
APC:CD8
PBS
OVA
Rv3875
Rv0232
PE:IL-2
0.27%
0.303%
APC:CD8
Rv1811
Rv1115

The data of T cell proliferation were stimulation by proteins.

CD4+			
PBS	0	0.16	0
OVA	0	0	0
Rv3875	0	1.27	0.21
Rv0232	0	3.36	0.383
Rv1811	0	2.85	0
Rv1115	0	0.74	0

CD8+			
PBS	0	0.17	0
OVA	0	0	0
Rv3875	0	0	0.107
Rv0232	0	0.47	0.37
Rv1811	0	0	0
Rv1115	0	0.36	0

The data of T cell proliferation were stimulation by PHA.

CD4-PHA			
PBS	39.32	1.03	1.78
OVA	37.38	0	0
Rv3875	18.9	0.96	1.32
Rv0232	56.63	0.58	0.245
Rv1811	56.61	0.62	0.99
Rv1115	59.49	1.47	1.77

CD8-PHA			
PBS	90.96	31.65	38.903
OVA	80.98	25.29	12
Rv3875	79.72	13.49	15.484
Rv0232	85.76	21.61	19.84
Rv1811	79.17	13.36	5.9
Rv1115	88.02	20.18	19.8

The results of T cell proliferation were stimulation by proteins.

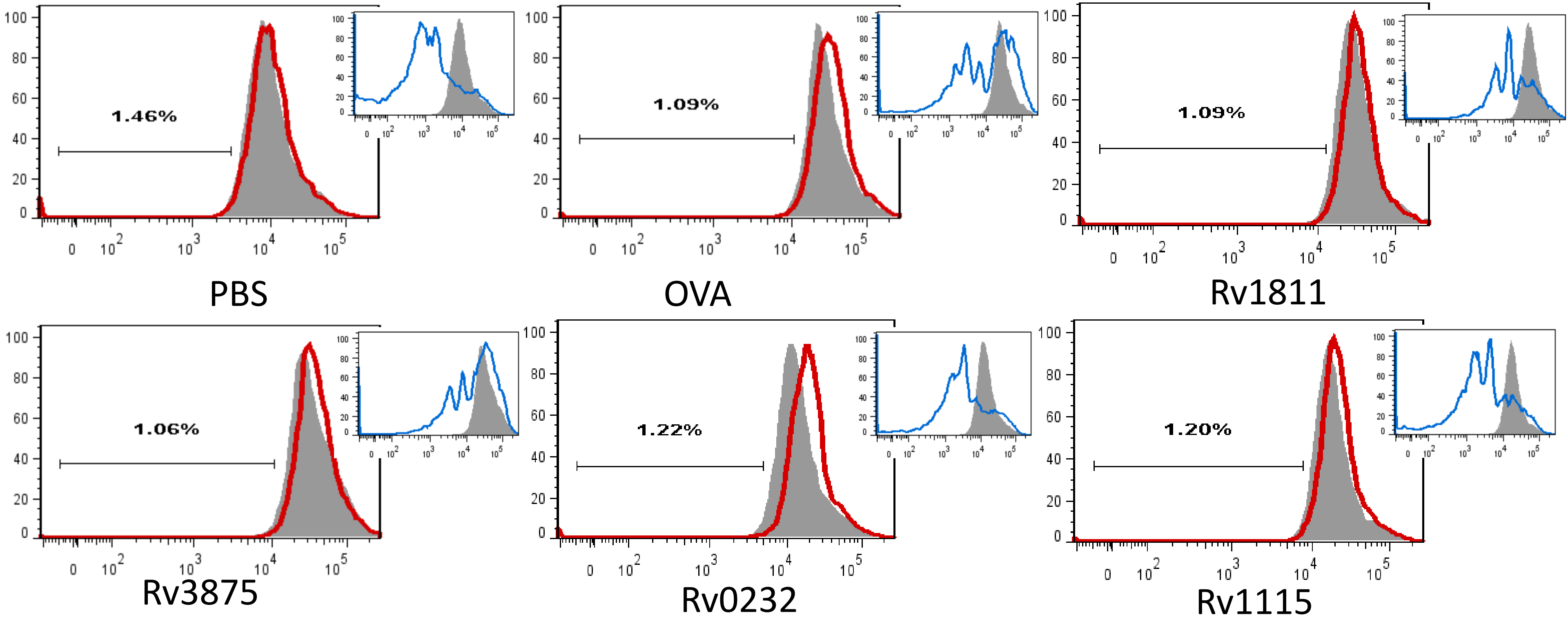

First CD4+ proliferation

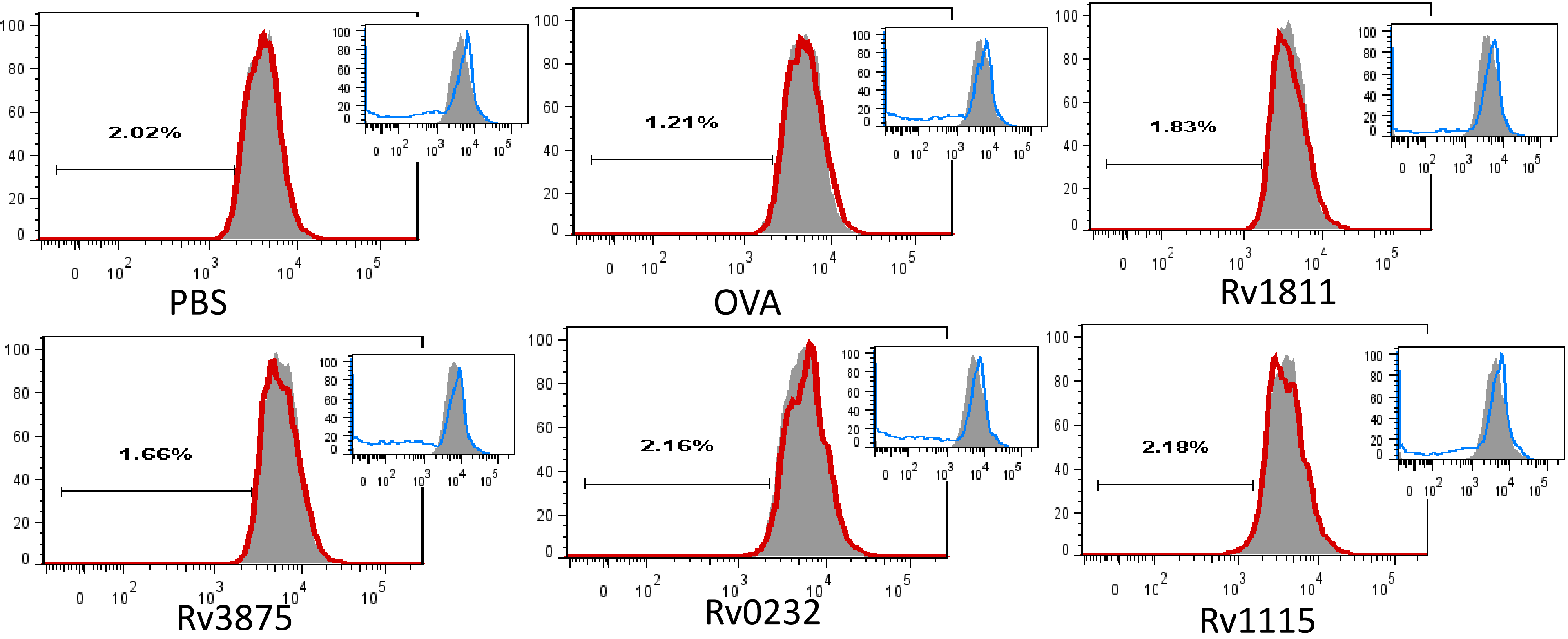

Second CD4+proliferation

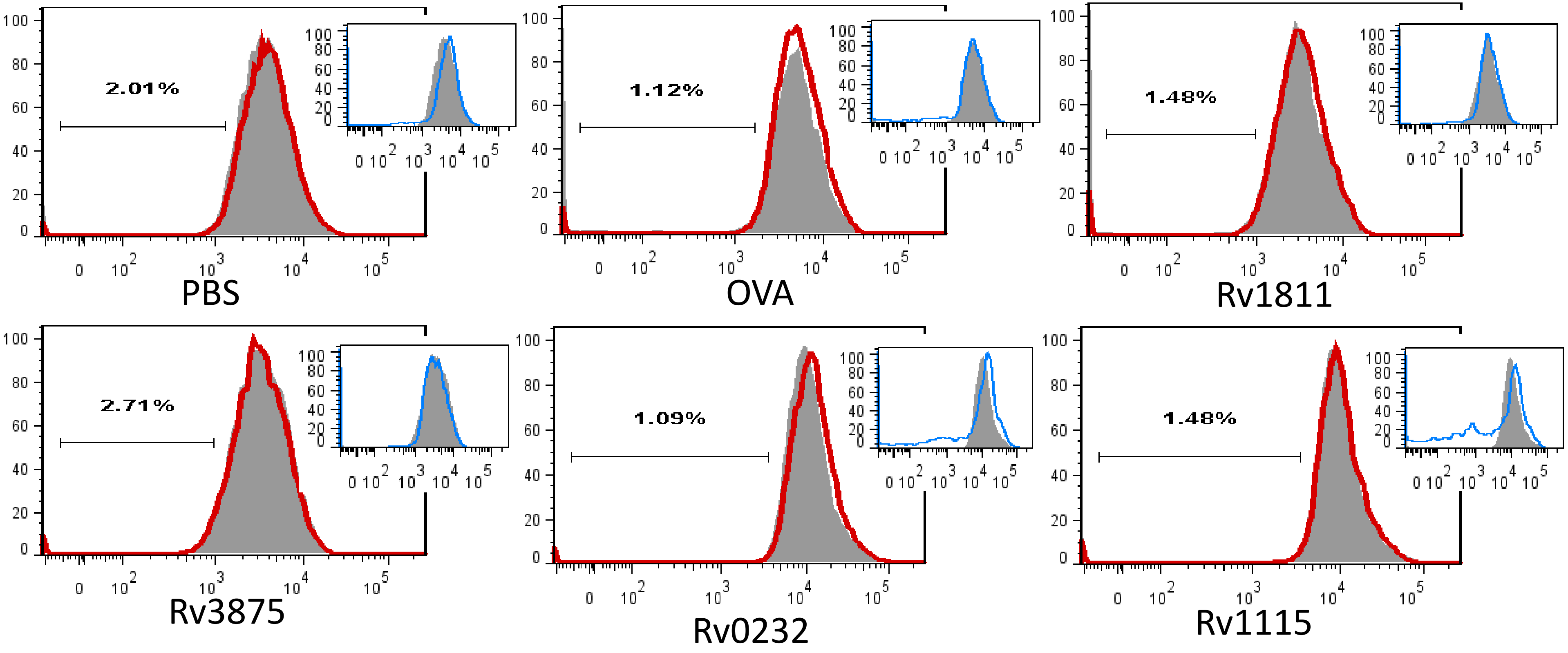

Third CD4+ proliferation

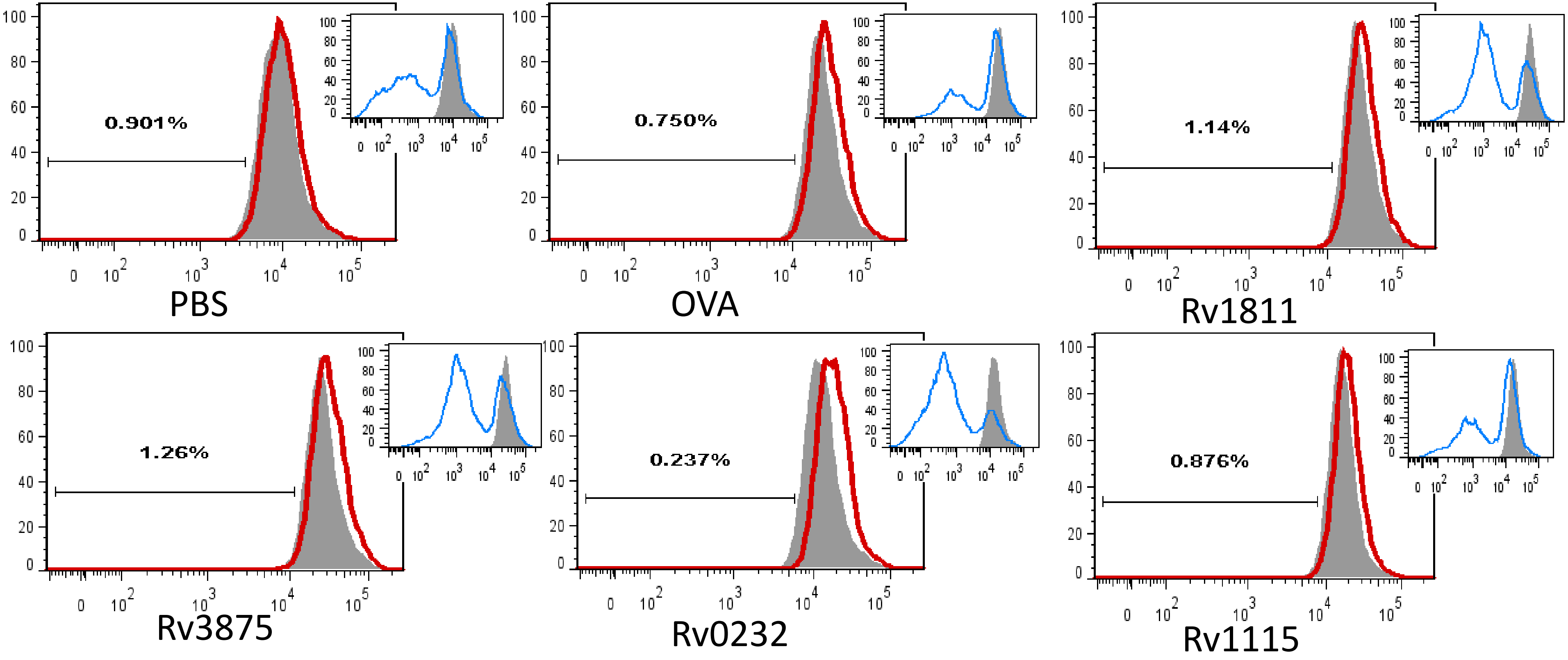

First CD8+prolifertion

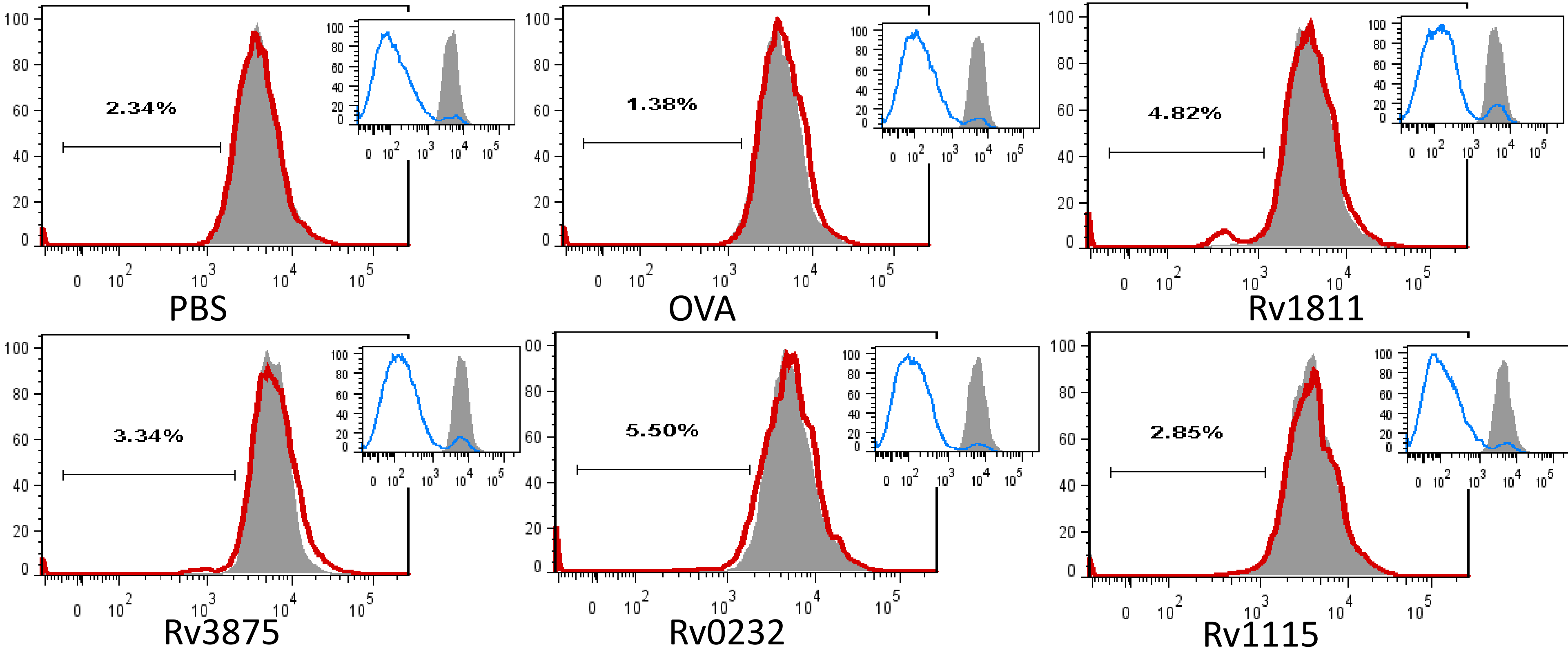

Second CD8+prolifertion

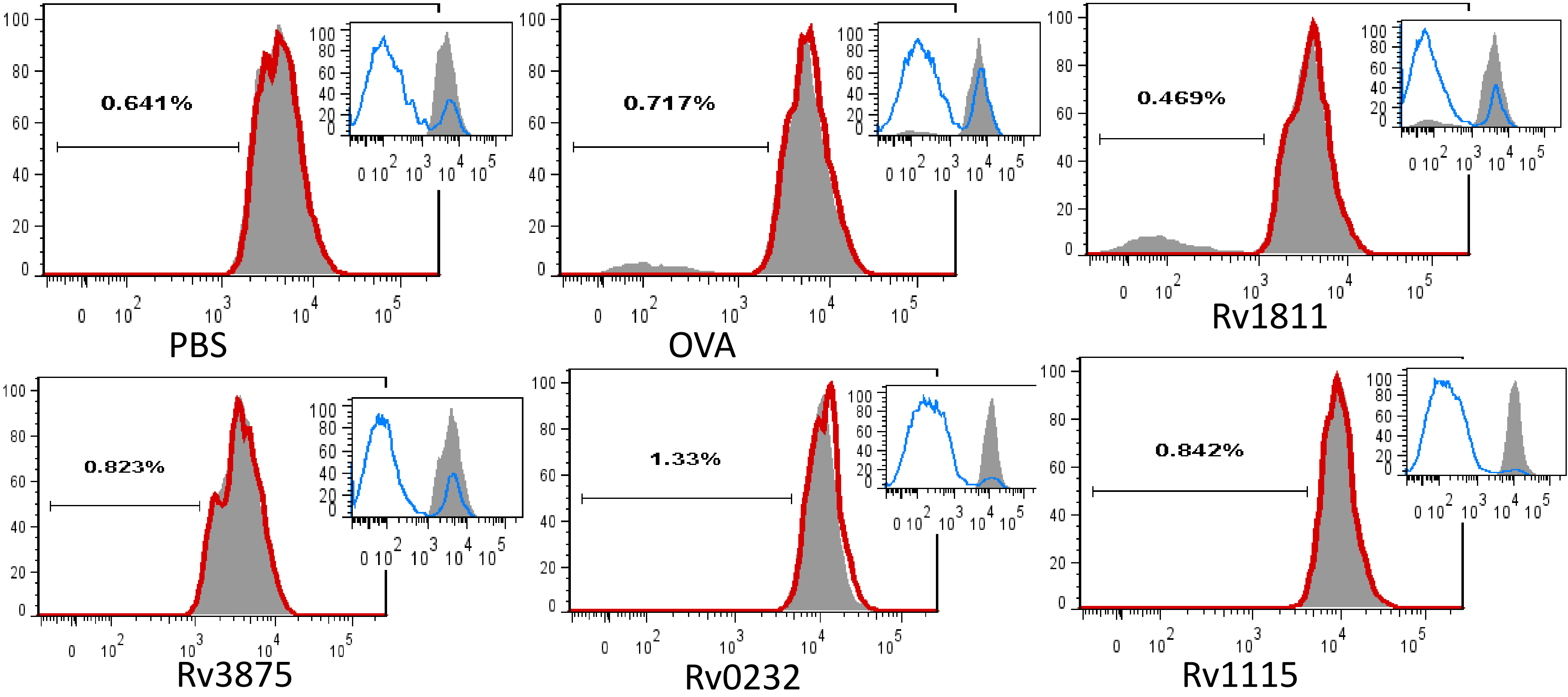

Third CD8+profertion

The data of cytokines were stimulation by PHA.

GM-CSF			
PBS	147.2	154.87	165
OVA	122.99	155.17	136.68
Rv3875	245.15	222.62	212.22
Rv0232	532.73	552.66	500.82
Rv1811	272.48	263.04	290.3
Rv1115	305.62	341	329.12

IFN-γ			
PBS	3001	2885.7	2783
OVA	3039	2985.7	2745
Rv3875	4627	4883.7	4886
Rv0232	6671	6385.7	6023
Rv1811	3354	3685.7	3067
Rv1115	6258	6085.7	6281

IL-2			
PBS	97.86	80.76	90
OVA	87.49	88.94	91.95
Rv3875	166.17	187.32	171.3
Rv0232	40.17	47.26	41.59
Rv1811	37.96	36.43	32.31
Rv1115	44.87	42.22	49.56

IL-4			
PBS	6.99	8.75	7
OVA	8.65	7.64	6.04
Rv3875	11.04	11.1	10.19
Rv0232	18.74	17.73	17.05
Rv1811	22.51	21.76	21.53
Rv1115	17.74	16.82	15.63

IL-6			
PBS	35.18	37.9	40
OVA	51.47	52.94	49.73
Rv3875	81.2	77.87	94.74
Rv0232	112.3	147.83	113.14
Rv1811	42.83	43.62	31.34
Rv1115	410.5	501.1	432.66

IL-12P40			
PBS	0	0	0
OVA	0	0	0
Rv3875	0	0	0
Rv0232	0	0	0
Rv1811	0	0	0
Rv1115	0	0	0

IL-17			
PBS	391.11	321	400
OVA	428.86	404.17	379.9
Rv3875	793.56	863.94	767.96
Rv0232	1566	1800	1848.2
Rv1811	851.28	613.97	692.7
Rv1115	421.9	417.95	422.41

IP-10			
PBS	540.83	595.01	490
OVA	561.22	562.13	635.16
Rv3875	1199.9	1006.1	1123.5
Rv0232	794.29	818.56	742.38
Rv1811	552.05	477.76	465.89
Rv1115	898.67	879.49	919.57

TNF-α			
PBS	52.62	47.05	50
OVA	46.04	47.53	52.14
Rv3875	84.51	101.72	99.01
Rv0232	97.83	85.74	67.73
Rv1811	62.78	87.26	75.61
Rv1115	64.37	81.85	72.17

The data of cytokines were stimulation by proteins.

GM-CSF			
PBS	0	0	0
OVA	2.1	1	1.73
Rv3875	7.73	17.04	10
Rv0232	36	52.77	43.1
Rv1811	6.5	4	2.57
Rv1115	6.07	7	9.27

IFN-γ			
PBS	0	0	0
OVA	0	0	0
Rv3875	1.83	0	0.7
Rv0232	388	414.81	406.49
Rv1811	0.3	0.94	0.7
Rv1115	20	28	36.43

IL-2			
PBS	0	0	0
OVA	2	1.42	1
Rv3875	5.56	6.95	6
Rv0232	24.19	20.91	25.78
Rv1811	0.7	0.6	0.75
Rv1115	5	6.05	5.59

IL-4			
PBS	0	0	0
OVA	0	0	0
Rv3875	0.6	1.17	0.7
Rv0232	2	3.03	3
Rv1811	0	0	0
Rv1115	1.8	2.2	2.25

IL-6			
PBS	0	0	0
OVA	2.3	2.8	2.2
Rv3875	41.2	35.6	42.17
Rv0232	642.37	722.43	674.98
Rv1811	13.46	13.19	10
Rv1115	45.99	38.43	47.83

IL-12P40			
PBS	0	0	0
OVA	0	0	0
Rv3875	0.74	0.5	0.6
Rv0232	1.6	1.2	1.53
Rv1811	0	0	0
Rv1115	2.7	3.16	3.03

IL-17			
PBS	0	0	0
OVA	0	0	0
Rv3875	0	0	0
Rv0232	1200	1343	1254.8
Rv1811	0	0	0
Rv1115	20	18.5	17.54

IP-10			
PBS	0	0	0
OVA	0	0	0
Rv3875	10.12	12.99	12.9
Rv0232	103.68	101.77	83.43
Rv1811	6.31	5.03	6.85
Rv1115	46.29	50.9	64.01

TNF-α			
PBS	0	0	0
OVA	0	0	3.2
Rv3875	18.22	35.55	28.66
Rv0232	27.01	32.46	38.12
Rv1811	78.62	89.95	59.02
Rv1115	35.28	34.28	25.61
